# Photochemical Transformations of Peptides Containing the *N*-(2-Selenoethyl)glycine Moiety

**DOI:** 10.1021/acsomega.4c01015

**Published:** 2024-03-29

**Authors:** Özge Pehlivan, Kamil Wojtkowiak, Aneta Jezierska, Mateusz Waliczek, Piotr Stefanowicz

**Affiliations:** Faculty of Chemistry, University of Wrocław, F. Joliot-Curie str. 14, 50-383 Wrocław, Poland

## Abstract

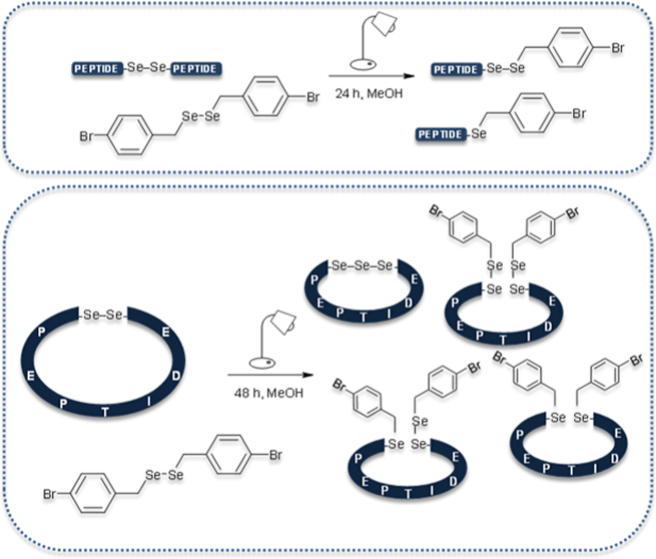

The diselenide bond has attracted considerable attention due to its ability to undergo the metathesis reaction in response to visible light. In our previous study, we demonstrated visible-light-induced diselenide metathesis of selenocysteine-containing linear peptides, allowing for the convenient generation of peptide libraries. Here, we investigated the transformation of linear and cyclic peptides containing the *N*-(2-selenoethyl)glycine moiety. The linear peptides were highly susceptible to the metathesis reaction, whereas the cyclic systems gave only limited conversion yields of the metathesis product. In both cases, side reactions leading to the formation of mono-, di-, and polyselenides were observed upon prolonged irradiation. To confirm the radical mechanism of the reaction, the radical initiator 2,2′-azobis[2-(2-imidazolin-2-yl)propane] dihydrochloride (VA-044) was tested, and it was found to induce diselenide metathesis without photochemical activation. The data were interpreted in the light of quantum-chemical simulations based on density functional theory (DFT). The simulations were performed at the B3LYP-D3BJ/def2-TZVP level of theory using a continuum solvation model (IEF-PCM) and methanol as a solvent.

## Introduction

Dynamic combinatorial chemistry (DCC) is a method that generates thermodynamically controlled libraries of chemical compounds by interconverting the building blocks through reversible covalent or noncovalent interactions.^1^ Various dynamic covalent bonds (DCBs), including boronate ester linkages, imine linkages, and disulfide linkages, have been widely used for biomedical applications^2^ such as wound dressing,^3^ antibacterial activity,^4^ and gene delivery.^5^ In recent years, diselenide bonds have emerged as a new class of DCBs capable of undergoing metathesis in response to many stimuli, including pH, heat, and light.^6−17^ In addition, osmotic pressure has been shown to be an external force to induce the diselenide exchange reaction that can be modulated by varying the concentration of NaCl.^18^ More recently, pulse sonication has been demonstrated to cleave the Se–Se bond in polymers more rapidly, but not as effectively for small molecules, thus providing a more selective method to induce diselenide metathesis.^19^ Visible light can be employed for a wide range of compounds, including small diselenides and polymers.^20,21^ Due to the low bond energy of the Se–Se bond,^22^ visible light can easily break the diselenide bond without the need for a catalyst, making diselenide metathesis compatible with biological systems. This property of Se–Se bonds has paved the way for the development of dynamic systems with properties such as self-healing,^23−26^ shape memory,^27^ and so on.^28^

Selenium (Se) is a micronutrient that is essential for life. However, depending on the chemical form, dosage, and route of exposure, certain Se compounds exhibit toxic properties.^29^ Se is incorporated into proteins primarily in the form of selenocysteine (Sec), a naturally occurring amino acid present in eukaryotes, archaea, and eubacteria.^30^ The selenol group of Sec is susceptible to rapid oxidation, leading to the formation of diselenide bonds, which have been experimentally identified in the SelL (selenoprotein L) protein family.^31^ Besides their natural occurrence, diselenide bonds have been extensively utilized to enhance protein folding by replacing cysteine (Cys) with the Sec analogue.^32−34^ In our previous study, we demonstrated that the diselenide metathesis of Sec-containing linear peptides could be induced by visible light, enabling the construction of peptide libraries.^35^ This led to the idea of testing the applicability of the same approach to the chemistry of cyclic peptides. Herein, we present the synthesis of peptides containing the *N*-(2-selenoethyl)glycine moiety and investigate the diselenide metathesis of both linear and cyclic systems. This amino acid moiety is similar to Sec but is not chiral. Therefore, peptides containing *N*-(2-selenoethyl)glycine are not susceptible to epimerization in contrast to peptides containing Cys and Sec. Furthermore, this new amino acid could be useful for peptide stapling and cyclization. The example of using its sulfur analogue, *N*-(2-thioethyl)glycine, for the cyclization of leu-enkephalin has been given in our recent paper.^36^ The potential synthetic application of *N*-(2-selenoethyl)glycine was an additional motivation to study diselenide metathesis in peptides containing this residue. Therefore, we performed studies between low-molecular-weight diselenide and both linear and cyclic peptides under visible-light irradiation, where we observed decomposition of the metathesis products that led to the formation of mono-, di-, and polyselenides. To elucidate the underlying formation of the resulting species, we carried out density functional theory (DFT) calculations.^37,38^ In addition, electronic structure analyses based on the quantum theory of atoms in molecules (QTAIM)^39^ as well as the noncovalent interactions (NCI) index^40^ were performed to provide a deeper insight into the interatomic interactions present in the studied possible products. Finally, we introduce the thermal radical initiator, 2,2′-azobis[2-(2-imidazolin-2-yl)propane] dihydrochloride (VA-044), which has been shown to facilitate diselenide metathesis without photochemical activation.

## Results and Discussion

### Synthesis of Building Blocks and Incorporation into Peptide Sequences

**Figure 1:**
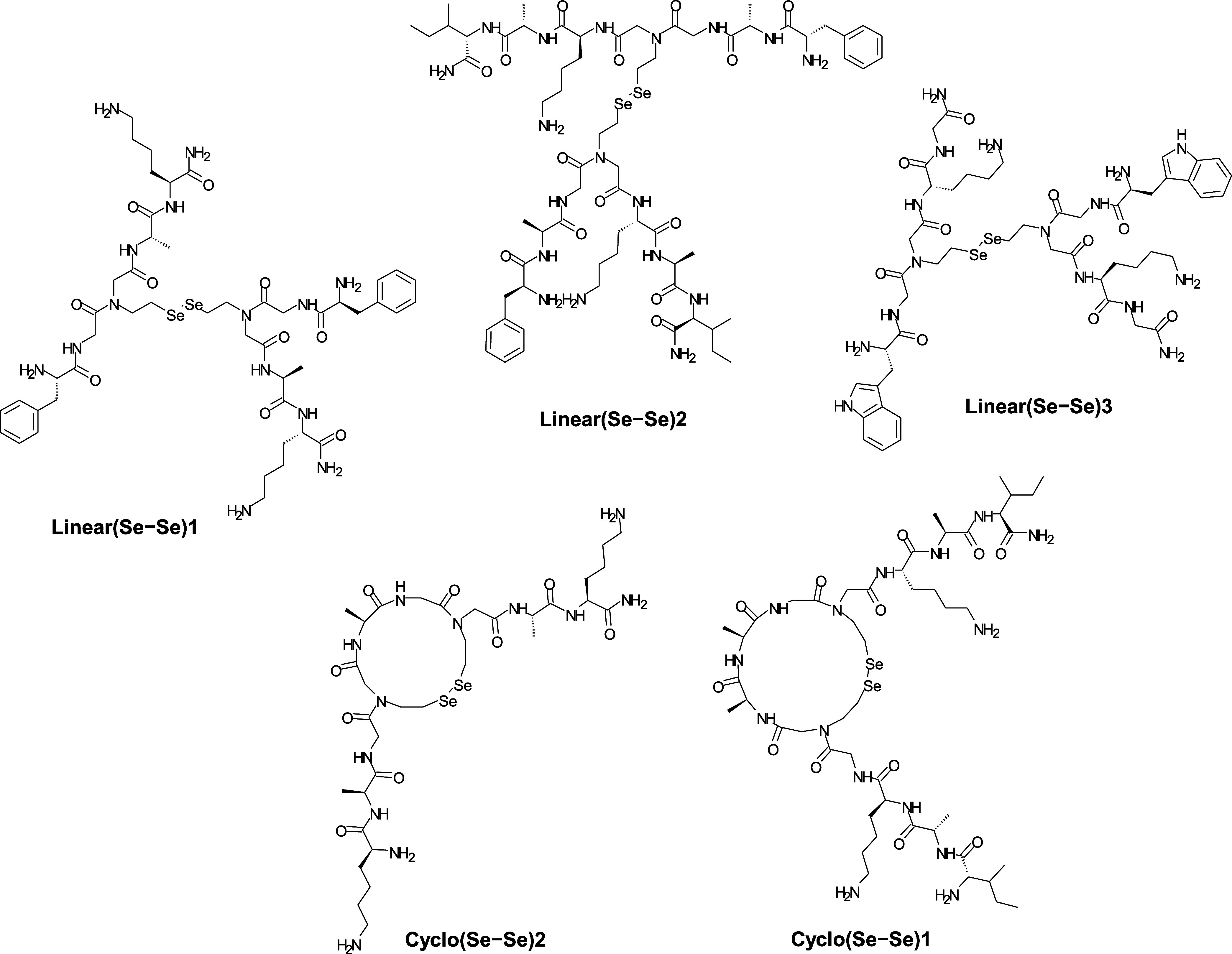
Structures of linear and cyclic peptides synthesized by combining the standard Fmoc-SPPS with the solid-phase submonomer method.

In order to study the diselenide metathesis of peptides, we synthesized a series of linear and cyclic peptides that contain the *N*-(2-selenoethyl)glycine moiety (Figure 1 and Table 2) using a combination of the standard Fmoc-SPPS and the solid-phase submonomer method.

**Scheme 1:**
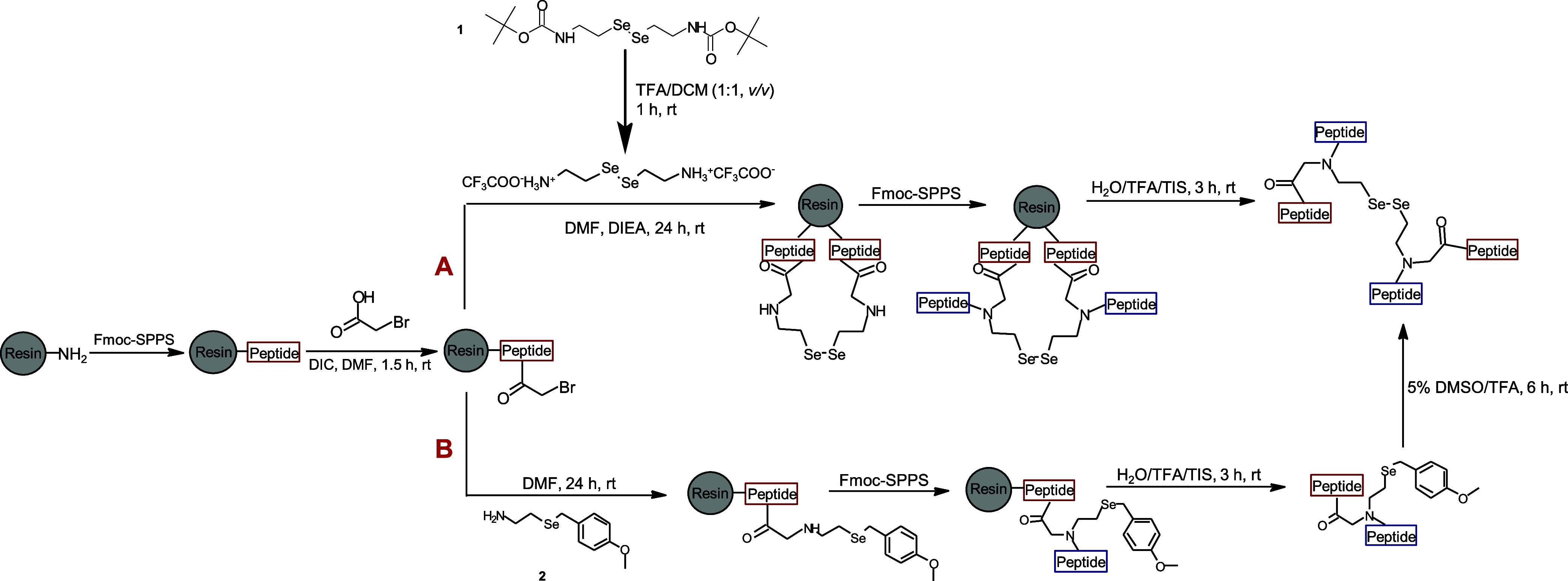
Synthesis of Linear Peptides by Incorporation of **1** and **2** via Pathways A and B, respectively

**Scheme 2:**
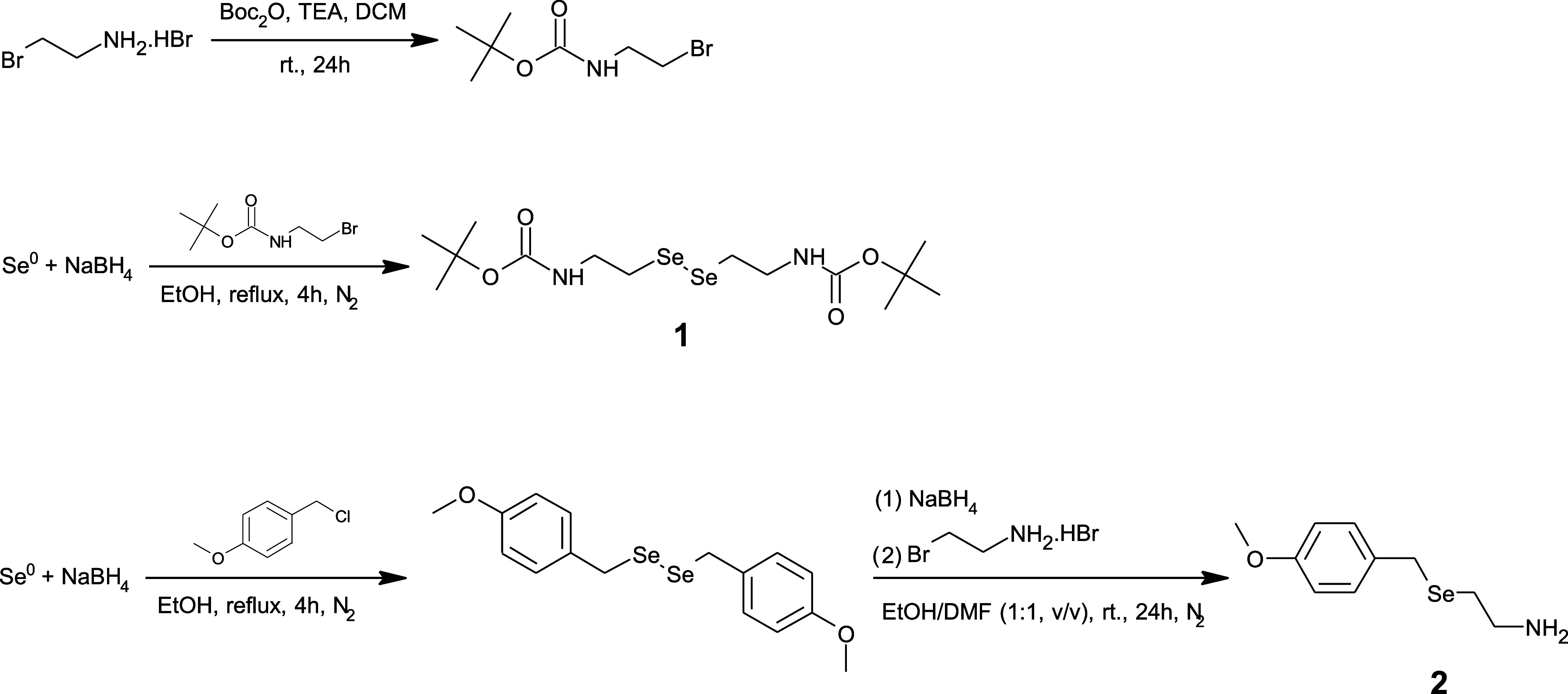
Synthetic Pathway of **1** and **2**

In particular, the main chain of the peptide precursors was extended by coupling amino acids according to the standard Fmoc-SPPS procedure,^41^ and the *N*-(2-selenoethyl)glycine moiety was sequentially assembled by bromoacetylation of the resin-bound secondary amine and bromine displacement according to the solid-phase submonomer method (Scheme 1).^42^ To enable the formation of the *N*-(2-selenoethyl)glycine, two Se-containing building blocks, **1** and **2**, were synthesized (Scheme 2) and subsequently employed in the bromine displacement step (Scheme 1). Although the designed diselenide-containing peptides were synthesized by different pathways, both building blocks contributed to the formation of the same peptide sequences. Building block **1** offered a simpler predeprotection step, while building block **2** required a longer postdeprotection treatment.

To incorporate **1** into the peptide sequence, the Boc-protecting group was removed for 1 h in a solution of trifluoroacetic acid (TFA)/dichloromethane (DCM) (1:1, v/v) to activate the amino functional group prior to the S_N_2 reaction (Scheme 1, pathway A). After the cleavage from the solid support, the peptides were obtained in a diselenide form.

As shown in pathway B (Scheme 1), after the cleavage step, the peptides containing **2** were treated with 5% dimethyl sulfoxide (DMSO)/TFA solution for 6 h to remove the *p-*methoxybenzyl protecting group, thereby resulting in the formation of a diselenide bridge. The building blocks and designed peptides were characterized by ^1^H NMR, ^13^C NMR, ^77^Se NMR, electrospray ionization mass spectrometry (ESI-MS), ESI-MS/MS, high-performance liquid chromatography (HPLC), matrix-assisted laser desorption ionization mass spectrometry (MALDI-MS), and liquid chromatography–mass spectrometry (LC–MS).

### Metathesis Reaction of Linear and Cyclic Peptides Induced by Visible Light

Our study involves a comparative analysis of the reactivity of linear and cyclic peptides in visible-light-induced diselenide metathesis. Accordingly, we began our investigation by examining the metathesis reaction between **Linear(Se–Se)1** and **BBSe**_**2**_. In our previous study, we observed that the presence or absence of oxygen did not significantly affect the results of photochemical reactions.^43^ Therefore, we did not perform the reactions in deaerated solutions. However, the reactions were performed in capped vials that were additionally sealed with parafilm. This limited the access of atmospheric oxygen to the sample.

For experimental details, a sample containing an equimolar concentration (5 mM) of **Linear(Se–Se)1** and **BBSe**_**2**_ in methanol was prepared and subsequently subjected to irradiation under an LED lamp (the characterization of the lamp is presented in the Supporting Information (SI)). The reaction progress was monitored by HPLC and ESI-MS analyses.

**Figure 2:**
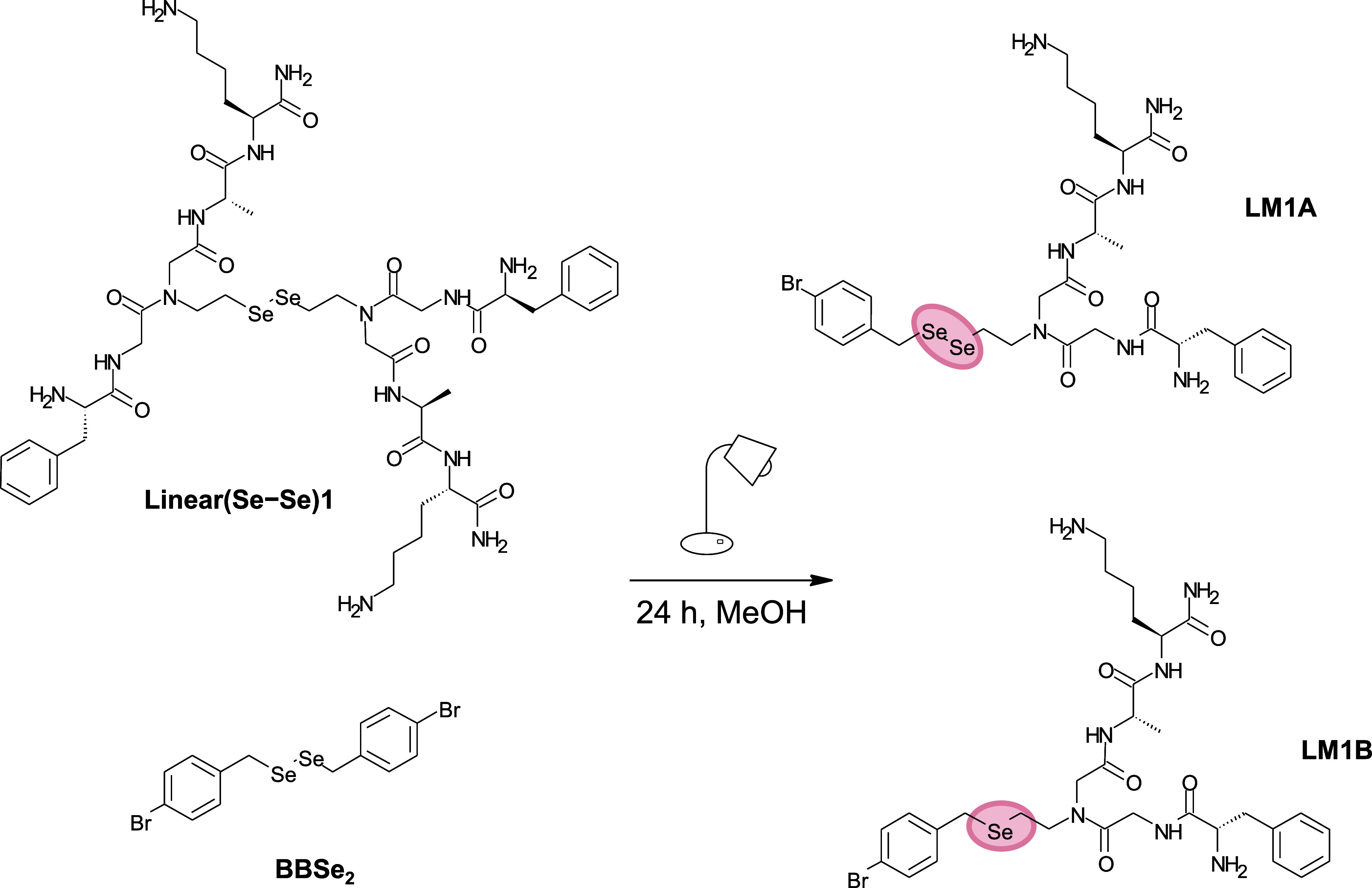
Metathesis reaction between **Linear(Se–Se)1** and **BBSe**_**2**_, resulting in the formation of **LM1A** and **LM1B** in 24 h under visible light.

**Figure 3:**
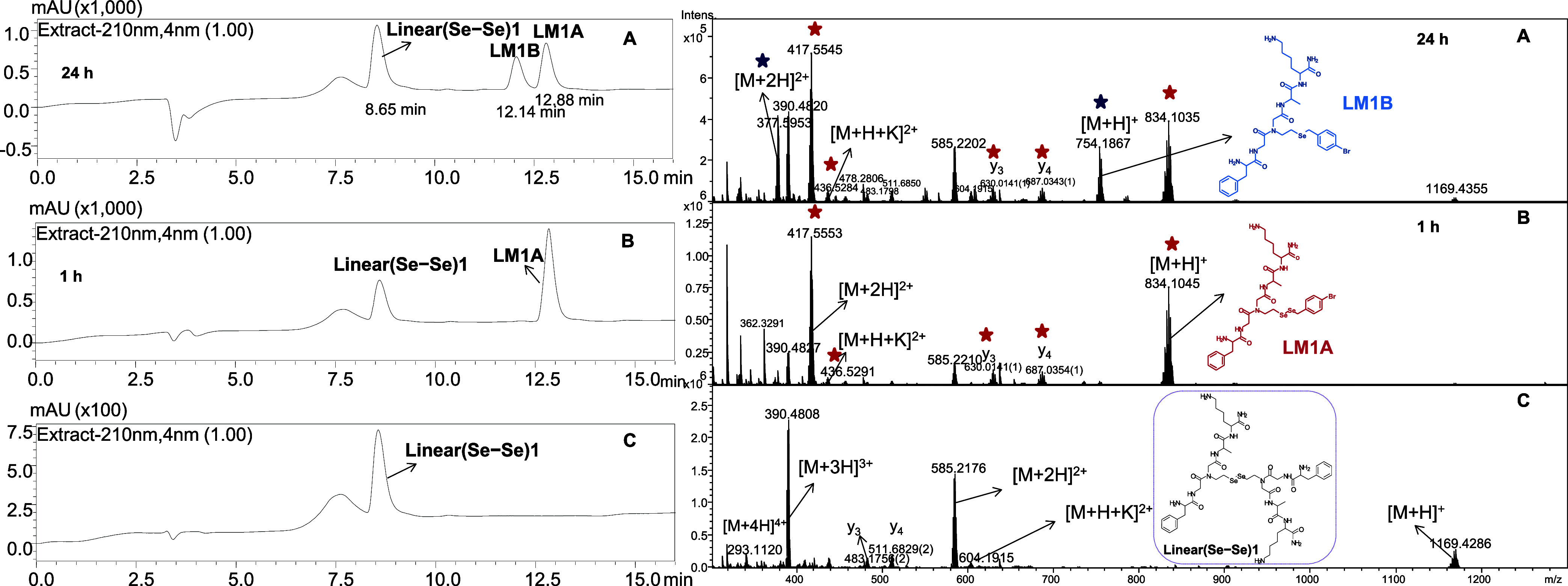
HPLC chromatograms (left) and ESI-qTOF-MS spectra (right) illustrating the progress of the metathesis reaction between **Linear(Se–Se)1** and **BBSe**_**2**_. Chromatograms (B) and (A) were acquired after 1 and 24 h of irradiation of the sample under visible light, respectively. (A broad signal adjacent to the main peak is present when only HPLC-grade solvents and no-column injection were also recorded (Figure S19)). Chromatogram (C) corresponds to the purified **Linear(Se–Se)1**. Retention times (r.t.) of **Linear(Se–Se)1**, **LM1B**, and **LM1A** are 8.65, 12.14, and 12.88 min, respectively. Spectra (B) and (A) were acquired after 1 and 24 h of irradiation of the sample under visible light, respectively. The spectrum (C) corresponds to the purified **Linear(Se–Se)1**. Red and dark-blue stars indicate peaks of **LM1A** and **LM1B**, respectively. Conditions: (5 mM) **Linear(Se–Se)1**, (5 mM) **BBSe**_**2**_, methanol, LED lamp 400–700 nm.

After 1 h of irradiation, the HPLC chromatogram (Figure 3) revealed two prominent peaks eluted with retention times of 8.65 and 12.88 min. By comparing the chromatogram of purified **Linear(Se–Se)1** with that after the metathesis reaction, the peak eluted at 8.65 min was attributed to **Linear(Se–Se)1**. According to the ESI-MS spectrum (Figure 3) of the analyzed sample, the predominant forms of singly protonated and doubly protonated ions were detected at *m*/*z* 834.1045 and *m*/*z* 417.5553, respectively. These ions were assigned to the metathesis product, **LM1A** (Figure 2), obtained by the exchange of the Se–Se bond between **Linear(Se–Se)1** and **BBSe**_**2**_, and the structure was confirmed by ESI-MS/MS analysis (Figure S24). Therefore, the newly formed peptide that was eluted at 12.88 min was identified as **LM1A**, obtained with a conversion yield of 70%. Upon the irradiation of the sample for 24 h, we observed a new peak with a retention time of 12.14 min in the HPLC chromatogram (Figure 3) along with those of **Linear(Se–Se)1** and **LM1A**. When the ESI-MS spectrum was analyzed (Figure 3), we detected a newly formed peptide ion at *m*/*z* 754.1867 (*z* = +), which was found to be 79.92 Da smaller than the singly protonated ion of **LM1A**, indicating the absence of one Se atom. Consequently, prolonged irradiation resulted in the formation of a metathesis product containing the selenoether bond, **LM1B** (Figure 2). In addition, the identified fragmentation ions in the MS/MS spectrum further confirmed the structure of **LM1B** (Figure S26).

To determine whether the obtained result was consistent among the peptides with different sequences, we conducted the same experiment with two alternative peptides, **Linear(Se–Se)2** and **Linear(Se–Se)3**. As supported by HPLC and ESI-MS/MS experiments, in both cases, the selenoether bond was formed within 24 h and the reaction resulted in a mixture containing mono- and diselenides (Figures S29–S38 and S43–S52). Our group previously demonstrated the extrusion of Se from cyclic peptides under UV light (254 nm), where selenolanthionines were derived from their corresponding selenocystines.^43^ Payne et al. reported the formation of selenoethers from selenocystine-containing homodimers when treated in the presence of phosphine and an iridium photocatalyst under LED_450_.^44^ It is worth noting that, in our case, neither HPLC nor ESI-MS analysis detected the degraded species of peptide homodimers, **Linear(Se–Se)1–3**, resulting from Se extrusion under visible-light irradiation. In addition, we performed a diselenide metathesis between **Linear(Se–Se)1** and **Linear(Se–Se)2** to determine whether the result would be similar to that observed between a linear peptide and **BBSe**_**2**_. However, the reaction resulted in the formation of an exclusive metathesis product, **LM12**, within 24 h of irradiation of the sample (Figures S39–S42) without inducing further decomposition of **LM12** into monoselenide. Importantly, the inability to ionize **BBSe**_**2**_ precludes the identification of this compound by ESI-MS as we did not use the additional ionization source during the analysis. To understand whether the extrusion of Se could be associated with the decomposition of **BBSe**_**2**_, we exposed a 5 mM methanolic solution of **BBSe**_**2**_ to visible light for 24 h and analyzed the resulting sample by gas chromatography–mass spectrometry (GC–MS). According to the spectrum, we detected a peak at *m*/*z* 169 that was ascribed to the fragment of 1,2-bis(4-bromophenyl)ethane, a compound devoid of Se atoms (Figures S97–S98). This observation is similar to studies on the thermolysis of neat dibenzyl diselenide^45^ and bis(diphenylmethyl) diselenide^46^ at elevated temperatures, which induced the formation of 1,2-diphenylethane and 1,1,2,2-tetraphenylethane, respectively.

The photodecomposition of dibenzyl diselenide to monoselenide at 350 nm was documented early,^47^ the mechanism of which has been proposed to involve the cleavage of the C–Se bond.^48^ Therefore, we presume that the weaker C–Se bond in **BBSe**_**2**_ compared to that in linear peptides may account for the decomposition of the metathesis product under prolonged irradiation since the energy is sufficient to break the C–Se bond of **BBSe**_**2**_ unlike the C–Se bond of linear peptides. To support this hypothesis, we calculated the bond dissociation enthalpies (BDEs) of the benzylic and peptide C–Se bonds of the **LM1A**, confirming that the benzylic C–Se bond was found to be less stable than the peptide C–Se bond.

In light of the above findings, we were encouraged to investigate the metathesis reaction on cyclic peptides and ascertain the possibility of subsequent decomposition of the metathesis products. Therefore, two model peptides, **Cyclo(Se–Se)1** and **Cyclo(Se–Se)2**, were designed in such a way that the submonomer method was iterated twice at different positions in the peptide chain to achieve the formation of the desired monomers. The metathesis reaction between the cyclic peptide and **BBSe**_**2**_ was carried out under the same reaction conditions as for the linear diselenides, and the reaction progress was monitored by HPLC and ESI-MS analyses.

**Figure 4:**
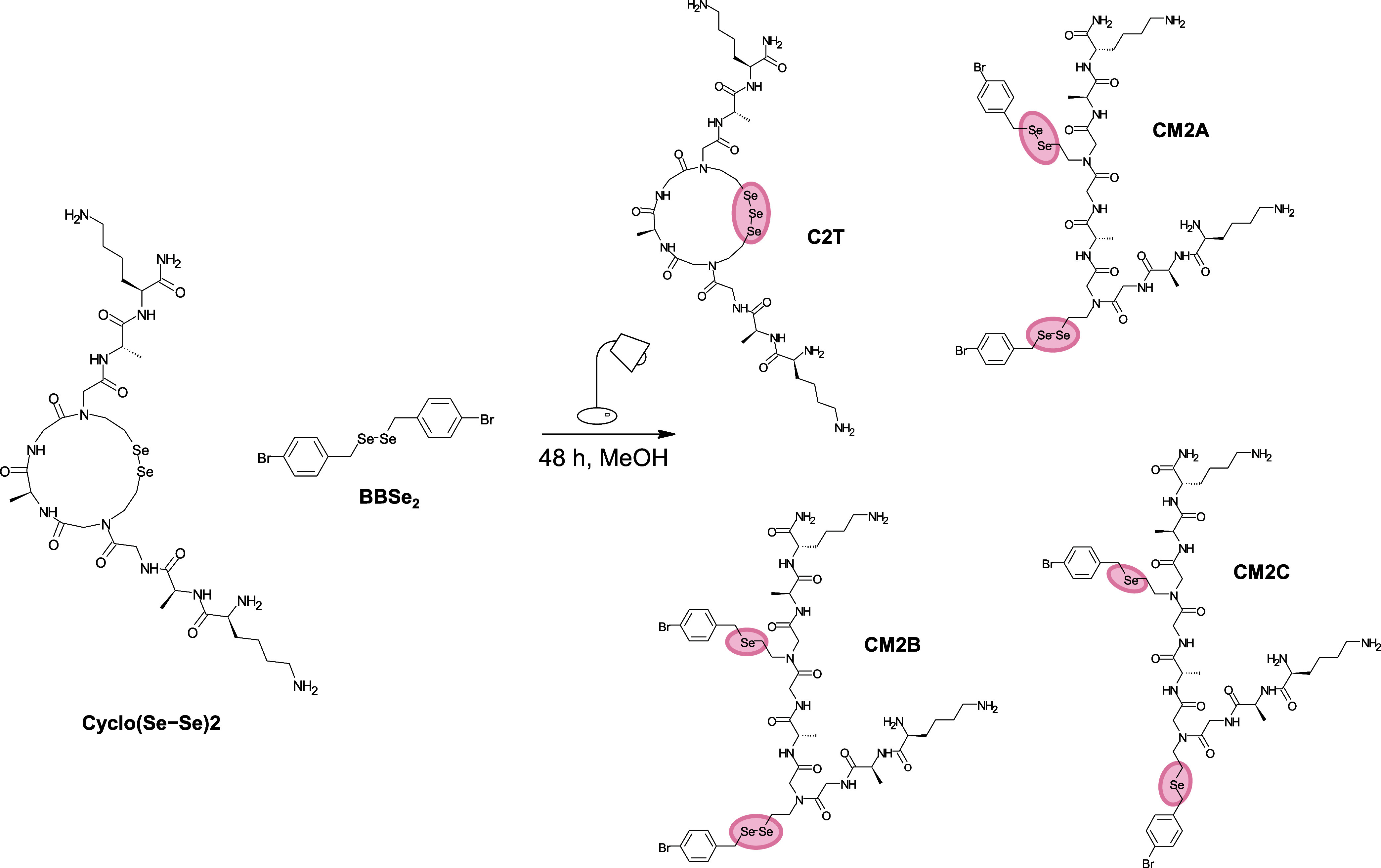
Metathesis reaction between **Cyclo(Se–Se)2** and **BBSe**_**2**_, resulting in the formation of **C2T**, **CM2A**, **CM2B**, and **CM2C** in 48 h under visible light.

In addition to the formation of metathesis products, **CM1A**/**CM2A**, and their degraded forms, **CM1B**/**CM2B** and **CM1C**/**CM2C**, we observed the formation of cyclic triselenides, **C1T** and **C2T** (Figure 4). To the best of our knowledge, peptides and proteins containing triselenide have not yet been discovered, although proteins with trisulfide bonds have been identified^49−51^ and characterized by mass spectrometry analysis.^52^

**Figure 5:**
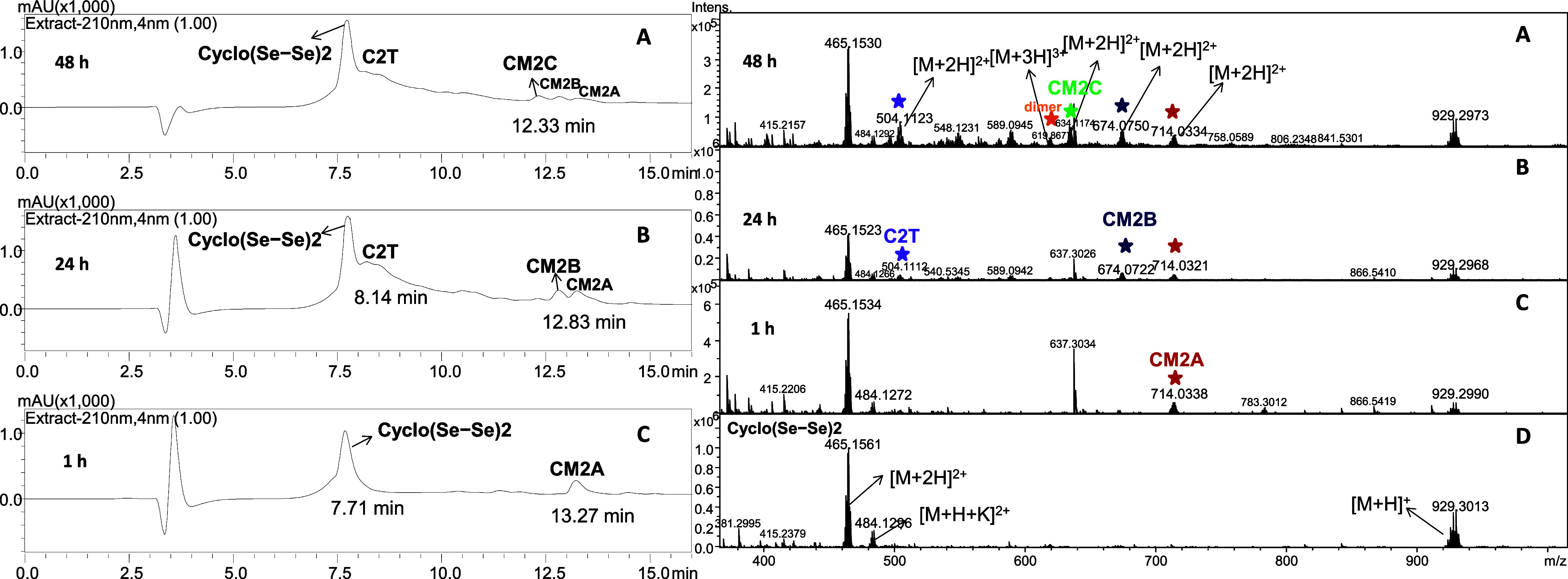
HPLC chromatograms (left) and ESI-qTOF-MS spectra (right) illustrating the progress of the metathesis reaction between **Cyclo(Se–Se)2** and **BBSe**_**2**_. Chromatograms (C), (B), and (A) were acquired after 1, 24, and 48 h of irradiation of the sample under visible light, respectively. Retention times (r.t.) of **Cyclo(Se–Se)2**, **C2T**, **CM2C**, **CM2B**, and **CM2A** are 7.71, 8.14, 12.33, 12.83, and 13.27 min, respectively. (A broad signal adjacent to the main peak is present when only HPLC-grade solvents and no-column injection were also recorded (Figure S19)). Spectra (C), (B), and (A) were acquired after 1, 24, and 48 h of irradiation of the sample under visible light, respectively. Spectrum (D) corresponds to the purified **Cyclo(Se–Se)2**. Violet, orange, green, dark-blue, and red stars indicate peaks of **C2T**, dimer, **CM2C**, **CM2B**, and **CM2A**, respectively. Conditions: (5 mM) **Cyclo(Se–Se)2**, (5 mM) **BBSe**_**2**_, methanol, and LED lamp 400–700 nm.

Considering our experimental results, **Cyclo(Se–Se)1** and **Cyclo(Se–Se)2** followed a slightly different reaction process (Table 1) that could be attributed to the different sequences of the peptides as well as to the size of the diselenide loop. In particular, **Cyclo(Se–Se)1** transformed into **C1T** (r.t. = 9.51 min) and **CM1A** (r.t. = 13.54 min) within 1 h of irradiation (Figures S59–S61 and S63), whereas **Cyclo(Se–Se)2** only yielded **CM2A** (r.t. = 13.27 min, 40%) within the same irradiation period (Figures 5, S87). Nevertheless, the formation of **C2T** (r.t. = 8.14 min) was obtained within 24 h (Figures 5 and S93). In both cases, we observed the degraded species of **CM1A** (Figures S67 and S70) and **CM2A** (Figures 5, S89, and S91) as well as the dimerization of **Cyclo(Se–Se)1** (Figure S65) and **Cyclo(Se–Se)2** (Figure S95) within 48 h. All of the resulting photoproducts, including **CM1A–C**, **CM2A–C**, **C1T**, and **C2T**, were further subjected to MS/MS experiments to characterize their structures and to support the results obtained in the MS experiments (Figures S62, S64, S66, S69, S72, S88, S90, S92, S94, and S96).

**Table 1:** List of Peptides Formed at Different Time Intervals when a Sample Containing a Peptide and **BBSe**_**2**_ Was Irradiated with Visible Light^a^

	peptides obtained after		
peptides subjected to irradiation	1 h irradiation	24 h irradiation	48 h irradiation
Linear(Se–Se)1	Linear(Se–Se)1	Linear(Se–Se)1	X
	LM1A	LM1A	
		LM1B	
Linear(Se–Se)2	Linear(Se–Se)2	Linear(Se–Se)2	X
	LM2A	LM2A	
		LM2B	
Linear(Se–Se)3	Linear(Se–Se)3	Linear(Se–Se)3	X
	LM3A	LM3A	
		LM3B	
Cyclo(Se–Se)1	Cyclo(Se–Se)1	Cyclo(Se–Se)1	Cyclo(Se–Se)1
	CM1A	CM1A	CM1A
	C1T	C1T	C1T
			CM1B
			CM1C
Cyclo(Se–Se)2	Cyclo(Se–Se)2	Cyclo(Se–Se)2	Cyclo(Se–Se)2
	CM2A	CM2A	CM2A
		CM2B	CM2B
		C2T	C2T
			CM2C

a X, **Linear(Se–Se)1–3** not irradiated for 48 h.

To ascertain the peak assignment in the HPLC chromatogram, we additionally performed LC-MS analysis for the reaction of **Cyclo(Se–Se)1**/**BBSe**_**2**_. As shown in Figures S73–S74, **C1T** was eluted with a retention time of 8.83 min, which is longer than that (8.14 min) of **Cyclo(Se–Se)1**. This can be explained by the increased hydrophobicity of the peptide due to the higher number of Se atoms. Regarding the degraded forms of **CM1A**, peptides with a reduced number of Se atoms were eluted with shorter retention times, as the hydrophobicity of each peptide decreased with the extrusion of Se (Figures S75–S76). Therefore, the information provided by the LC-MS analysis reinforces the accuracy of the identification of the peaks in our HPLC chromatogram (Figure 5).

Based on our results from the photochemical reactions, cyclic peptides were found to be less susceptible to diselenide metathesis compared to linear peptides, resulting in lower conversion yields of metathesis products. Therefore, we hypothesized that noncovalent interactions could play an important role in maintaining the cyclic form of the peptides and thus reduce the ability of the peptides to undergo diselenide metathesis. Similarly, Qi et al. reported on the metathesis reaction between the low-molecular-weight compound, BnSe_2_, and the diselenide-containing crown ether, BC7Se_2_, under visible light, which was found to be weaker than the reaction between noncyclic compounds due to the cyclic topology of BC7Se_2_.^15^

In order to determine whether there are any interactions in the studied systems, we performed noncovalent interaction (NCI) calculations. Accordingly, four products, including **CM2A**, **CM2B**, **CM2C**, and **C2T**, formed by the reaction between **Cyclo(Se–Se)2** and **BBSe**_**2**_ were taken into consideration to study the possible interactions. As discussed in the theoretical part, the molecules were found to be stabilized by a network of noncovalent interactions, mainly hydrogen bonds.

**Figure 6:**
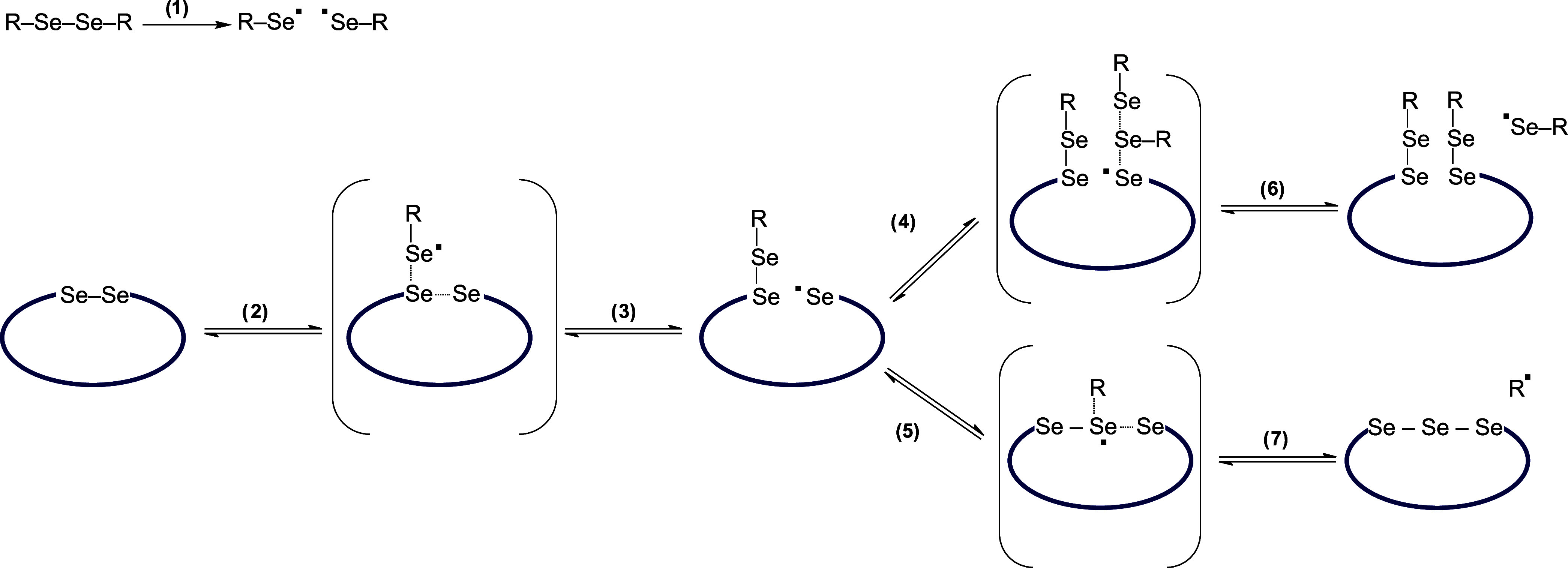
Hypothetical mechanism for the formation of **CM1A**/**CM2A** (**6**) and **C1T**/**C2T** (**7**).

To gain a better understanding of the formation of cyclic triselenides (**C1T** and **C2T**) and the metathesis products (**CM1A** and **CM2A**), we followed a [2 + 1] radical mechanism that has been theoretically supported for the exchange of aromatic diselenides.^53^ According to our hypothetical mechanism (Figure 6), we propose a process that proceeds through the cleavage of the Se–Se bond of the aromatic compound. This generates selenyl radicals (1) that attack the diselenide bond of the cyclic peptide (2), forming new selenyl radicals (3) through a three-membered transition state. This radical can attack either the Se–Se bond of the aromatic compound (4), forming **CM1A**/**CM2A** and a selenyl radical (6), or the Se–Se bond of the cyclic compound (5), resulting in the formation of **C1T**/**C2T** and a benzyl radical (7). A similar mechanism has been postulated for the radical metathesis of disulfides.^54,55^ Our experimental data remain in good agreement with the scheme shown in Figure 6. It explains the formation of **C1T** and **C2T** as byproducts. This mechanism also remains in good agreement with the much lower susceptibility of cyclic systems to metathesis than linear systems.

In the case of linear systems, the attack of the selenyl radical on the Se–Se bond leads to a system that can decompose with the reconstitution of either the original homodimeric system or the heterodimeric metathesis product. On the other hand, the cyclic system resulting from reaction 3 is transformed into a molecule containing both a diselenide bond and a selenyl radical. Therefore, the reverse reaction of reaction 3, or intramolecular reaction 5, is much more likely to occur than reaction 4, which requires a collision with another molecule containing a diselenide bond. The presence of noncovalent interactions, mainly hydrogen bonds, in our systems is likely a key factor in explaining the lower yield formation of the products. These hydrogen bonds help maintain the close proximity of the systems, further increasing the likelihood of the occurrence of a reverse reaction to reaction 3.

### Metathesis Reaction of Linear and Cyclic Peptides in the Presence of VA-044

Finally, we aimed to investigate the feasibility of the metathesis reaction using the thermal radical initiator, VA-044, which has been shown to be effective in reducing Sec to alanine (Ala) in the presence of TCEP.^56^

Taking advantage of the relatively low decomposition temperature of VA-044 (44 °C, in water), we opted to examine both linear and cyclic peptides to compare their ability to undergo the reaction at 45 °C, using a substoichiometric amount of VA-044. Accordingly, we separately incubated methanolic solutions of (5 mM) **Linear(Se–Se)1**/**BBSe**_**2**_ and (5 mM) **Cyclo(Se–Se)1**/**BBSe**_**2**_ in the presence of (0.5 mM) VA-044 at 45 °C. To ensure optimal experimental conditions, the samples were protected from light and the reactions were performed in capped vials that were sealed with parafilm. In addition, we performed control experiments in the absence of VA-044 to determine whether temperature alone had an impact on the possible conversion of peptides to their corresponding metathesis products.

**Figure 7:**
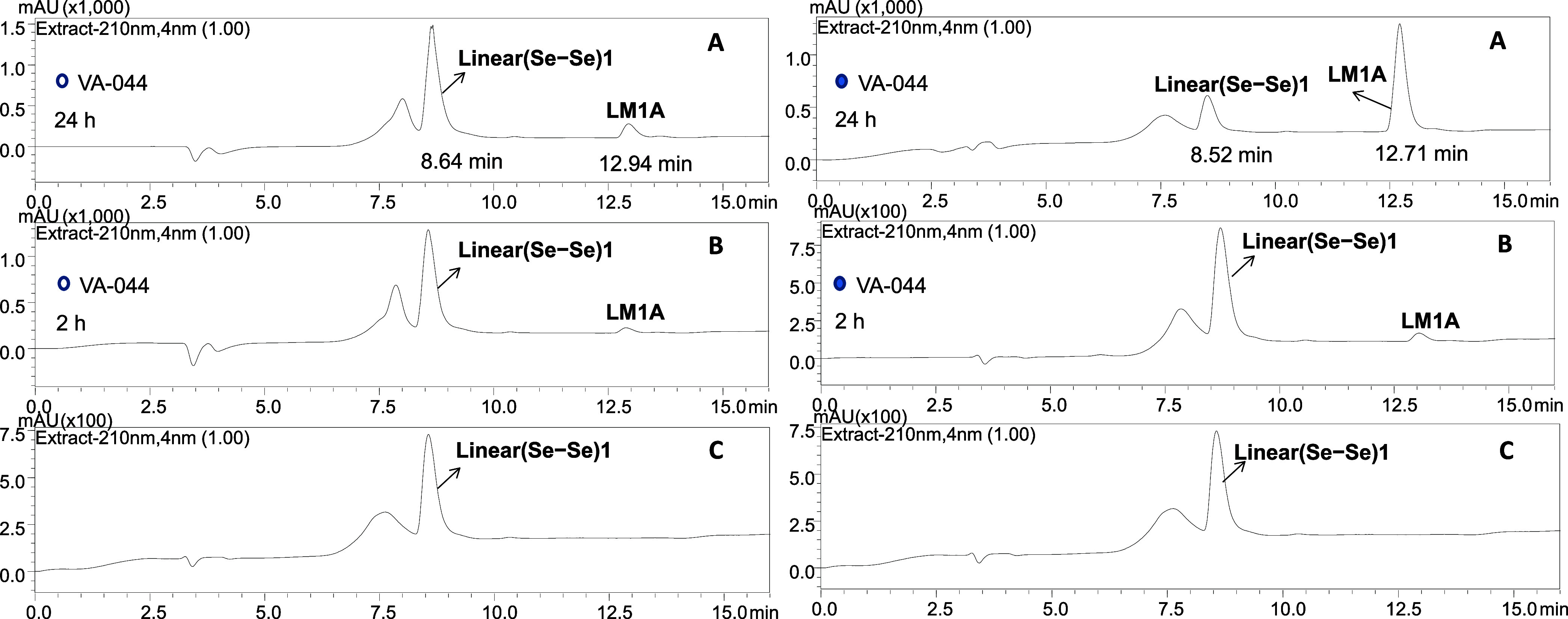
HPLC chromatograms illustrating the progress of the metathesis reaction between **Linear(Se–Se)1** and **BBSe**_**2**_. Chromatograms (B) and (A) were acquired after 2 and 24 h of incubation of the sample in the dark at 45 °C, respectively. Chromatogram (C) corresponds to the purified **Linear(Se–Se)1**. (A broad signal adjacent to the main peak is present when only HPLC-grade solvents and no-column injection were also recorded (Figure S19)). Retention times (r.t.) of **Linear(Se–Se)1** are 8.52 min (right) and 8.64 min (left). Retention times (r.t.) of **LM1A** are 12.71 min (right) and 12.94 min (left). Conditions: (5 mM) **Linear(Se–Se)1**, (5 mM) **BBSe**_**2**_, methanol, dark, 45 °C in the presence of (0.5 mM) VA-044 (right, filled circle), and in the absence of VA-044 (left, unfilled circle).

When the sample of **Linear(Se–Se)1**/**BBSe**_**2**_/VA-044 was incubated for 24 h, we observed a significant increase in the intensity of **LM1A** (r.t. = 12.71 min) that eventually reached the predominant form (70%) (Figures 7 and S28). In contrast, the reaction in the absence of VA-044 was slower but still favored the formation of **LM1A**, albeit to a lesser extent (r.t. = 12.94 min, 27%) (Figures 7 and S27). This demonstrates that even though VA-044 is present at much lower concentrations (0.5 mM) than the other components (5 mM) in the metathesis mixture, it is effective in promoting diselenide metathesis due to its ability to initiate a chain reaction through the involvement of free radicals.

Regarding the conversion of **Cyclo(Se–Se)1** to **CM1A**, we observed significant differences when compared to **Linear(Se–Se)1**. Specifically, we detected a trace amount of **CM1A** in the absence of VA-044 (Figures S77–S78). However, when the thermal radical initiator was introduced, it promoted the formation of 36% **CM1A** (r.t. = 13.79 min) within 24 h (Figures S79–S80). It is worth noting that the low conversion yield of **CM1A** compared to **LM1A** indicates the low propensity of cyclic peptides to undergo diselenide metathesis, supporting the findings observed in the photochemical reactions. Interestingly, in contrast to the visible-light experiments, we did not observe decomposition of **LM1A** under heat and in the presence of the thermal radical initiator within 24 h of incubation of the samples. On the other hand, a trace amount of **C1T** was present when the sample containing **Cyclo(Se–Se)1** and **BBSe**_**2**_ was incubated with VA-044 within the same 24 h period. This is likely due to the more efficient excitation of the molecule caused by visible light compared to the other stimuli, resulting in the dissociation of the C–Se bond.

### Quantum-Chemical-Based Density Functional Theory (DFT) Results

Theoretical calculations were performed using DFT theory, which has been used in numerous studies to reliably evaluate bond and conformational energies and noncovalent interactions of medium-sized molecules, including systems containing heavy atoms.^57−59^ In our theoretical study, we took into consideration the linear model of **LM1A** (Figure S99) to investigate the bond dissociation enthalpies (BDEs) at the temperature of 298.15 K, electron density, and its Laplacian at the bond critical points (BCPs) as well as compliance constants of benzylic C–Se, Se–Se, and peptide C–Se bonds. Evaluation of the compliance constants can serve as a gauge of the bond stiffness and measures the extent by which the bond will be elongated by acting on it with a stretching force of 1 N magnitude. The results are presented in Table S1. Analyses of the quantities related to the above-mentioned covalent bonds provide the following conclusions. (1) The BDE value strongly depends on the chemical environment—it varies from −41.5989 obtained for benzylic C–Se up to −51.9277 for peptide C–Se and −56.5072 kcal/mol for Se–Se. (2) The quantum theory of atoms in molecules (QTAIM) analysis shows that the presence of both C–Se is manifested by the larger values of the electron density and the concomitant lower values of the Laplacian and the energy density at BCP when compared to their counterparts of Se–Se—it allows one to conclude that the covalency component is substantially larger for the C–Se bond than for the Se–Se bond. In the case of both C–Se bonds, electron density is more orthogonally contracted toward the bond path of C–Se (which is reflected by the negative values of the Laplacian and the energy density and thus the excess of the potential energy density at the BCP). (3) The increase in compliance constants is associated with the decreasing stiffness of the examined bonds. In this case, the rigidness of the bonds decreases in the following order: peptide C–Se, Se–Se, benzylic C–Se.

**Figure 8:**
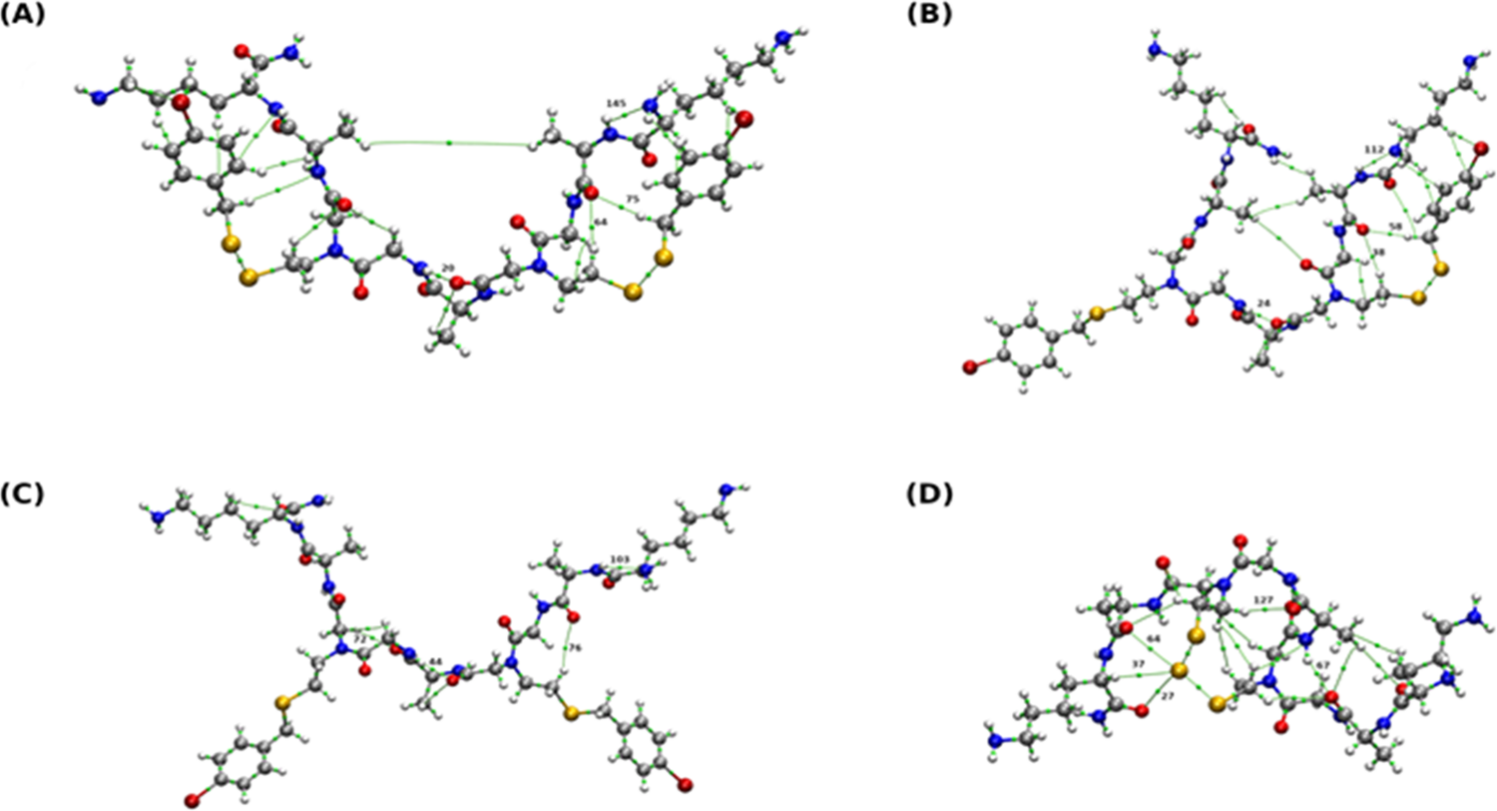
QTAIM graphs of the possible products obtained at the B3LYP-D3BJ/def2-TZVP level of theory and with the solvent reaction field reproduced by the IEF-PCM model and methanol as a solvent. Green dots indicate covalent and noncovalent interactions detected based on QTAIM. Selected bond critical points (BCPs) are designated with numbers. Color coding: gray, carbon; red, oxygen; blue, nitrogen; white, hydrogen; dark yellow, selenium; dark red, bromine. (A) – **CM2A**, (B) – **CM2B**, (C) – **CM2C**, and (D) – **C2T**.

Next, we focused on the possible four products obtained by the reaction carried out between **Cyclo(Se–Se)2** and **BBSe**_**2**_. The electronic structure analyses on the basis of QTAIM and NCI are presented in Figure 8, Table S2, and Figure S100. In Figure 8 are presented graphs obtained as a result of QTAIM analysis. Green dots indicate the presence of bond critical points (BCPs). Using the QTAIM theory, we can confirm the composition of the structure detected by physicochemical methods as well as reveal noncovalent interactions present in the studied molecules. Based on QTAIM graphs, we can postulate the presence of a network of noncovalent interactions (mainly hydrogen bonds) stabilizing the obtained structures. Selected quantitative parameters are presented in Table S2. The geometric parameters confirmed the presence of intramolecular hydrogen bonds: N–H···O and C–H···O. From the obtained numbers, we can conclude that they are middle-strength hydrogen bonds.

In the case of **CM2A**, we can see that one oxygen atom serves as an acceptor in two C–H···O hydrogen bonds. The presence of BCPs denoted as 64 and 75 revealed such a type of interaction. A similar situation was observed in **CM2B** (BCPs denoted as 38 and 58). Concerning **CM2C**, we did not observe such a kind of interaction. In **C2T**, where three Se atoms are making the −Se–Se–Se– moiety, BCPs were detected between selenium atoms and oxygen and hydrogen from the neighboring part of the molecule. As presented in Table S2, the electron density value at BCP is rather low.

The last part of the theoretical study is devoted to the search for secondary bonds of more delocalized characteristics than hydrogen bonds. In Figure S100, the three-dimensional (3D) plots of reduced density gradient (RDG) are presented for the discussed products. The visual representation arises from the isosurfaces of the reduced density gradient (RDG) colored by a scale of strength. Isosurfaces with the blue color indicate a strong attraction, green represents van der Waals interaction, and the red ones are associated with strong repulsion. In Figure S100, van der Waals interactions seem to play a significant role because they dominate the drawings. We can see red surfaces in the aromatic rings, which are responsible for steric effects. However, as shown in the plots, steric effects are also present in other parts of the investigated molecules. The presence of blue surfaces confirmed the presence of hydrogen bonds.

## Conclusions

In conclusion, we have successfully synthesized peptides containing the nonproteinogenic amino acid, *N*-(2-selenoethyl)glycine, using a combination of Fmoc-SPPS and the solid-phase submonomer method. This amino acid has structure and chemical properties similar to those of Sec, but it is achiral and therefore not susceptible to epimerization. We have studied the diselenide metathesis between the model peptides and low-molecular-weight diselenide under visible light and in the presence of the thermal radical initiator, VA-044. In particular, the latter was found to enhance the metathesis reaction at relatively low temperatures when used in a substoichiometric amount. Regardless of the external stimulus, we observed that linear peptides exhibited higher conversion rates to the metathesis products compared to cyclic peptides. The difference could be attributed to the stability of the cyclic structure, presumably due to noncovalent interactions. A similar effect of cyclic structures on diselenide metathesis was previously observed for diselenide-containing analogues of crown ethers. The linear peptides based on *N*-(2-selenoethyl)glycine are promising systems for dynamic covalent chemistry, whereas the reactivity of cyclic peptides is limited.

Electronic structure analyses based on QTAIM and NCI enabled us to see qualitative and quantitative electron density changes depending on the possible product structure. It was found that intramolecular hydrogen bonds and other present noncovalent interactions stabilize the structure in the methanol reaction field. Furthermore, the reactivity of the peptides under irradiation with visible light provided further insight, revealing that the benzylic C–Se bond was less stable than the peptide C–Se bond upon prolonged irradiation, leading to the extrusion of the Se atom from the metathesis product. Quantum-chemical simulations were carried out to estimate the bond dissociation enthalpy (BDE) for the linear model of the studied structure, confirming the lower stability of the benzylic C–Se bond compared to the peptide C–Se bond. Our research can pave the way for exploring new opportunities in the design and development of peptide-based systems with unique reactivity and promising applications in biological chemistry.

## Experimental Section

### NMR Spectroscopy

^1^H NMR and ^13^C NMR spectra were recorded on a Bruker Avance III 500 MHz equipped with a broadband inverse gradient probe head. ^77^Se NMR spectra were recorded on a Bruker Avance III 600 MHz equipped with a broadband inverse gradient probe head. ^1^H spectra were referenced to the residual solvent signals (CDCl_3_: δ = 7.26 ppm; DMSO-*d*_6_: δ = 2.50 ppm; MeOD: δ = 3.31 ppm).

### Purification of Peptides

Peptide purification was carried out by preparative RP-HPLC (Vydac C18 column, 22 mm × 250 mm) using a linear gradient of 5–95% S2 (S1 0.1% TFA in H_2_O, S2 80% MeCN + 0.1% TFA) within 50 min with UV absorbance at 210 nm. The flow rate was 7.0 mL/min.

### ESI-MS Analysis

Analysis was carried out on both the LCMS-9030 qTOF (Shimadzu) equipped with the electrospray ion source (ESI) (mobile phase; A: 0.1% HCOOH in H_2_O and B: 0.1% HCOOH in MeCN) and the Bruker qTOF compact equipped with the electrospray ion source (ESI) (solvent: MeCN/H_2_O/HCOOH (50:50:0.1)). ESI-MS/MS analysis was carried out on the Bruker qTOF compact equipped with the electrospray ion source (ESI). The collision-induced dissociation (CID) technique was used with argon as the collision gas, and the collision energy was optimized in the range of 15–30 eV for each peptide. A solution of MeCN/H_2_O/HCOOH (50:50:0.1) was used as the solvent.

### MALDI-MS Analysis

Analysis was carried out on a JEOL JMS-S3000 SpiralTOF-plus Ultra-High Mass Resolution MALDI-TOFMS. 2,5-Dihyroxybenzoic acid (DHB) was used as the matrix.

### LC-MS Analysis

Analysis was carried out on a hybrid LCMS-IT-TOF (Shimadzu) equipped with the electrospray ion source (ESI). The separation was performed on an Aeris Peptide XB-C18 column (100 mm × 21 mm), 3.6 μm bead diameter, with a gradient elution (B%) of 0–60% B (eluent A: 0.1% HCOOH in H_2_O; eluent B: 0.1% HCOOH in MeCN) within 15 min. The flow rate was 0.2 mL/min.

### HPLC Analysis

Analysis was carried out on a Nexera X2 UHPLC (Shimadzu) with UV control using a PDA detector (190–380 nm). The separation was performed on an Aeris Peptide XB-C18 column (100 mm × 21 mm), 3.6 μm bead diameter, with a gradient elution (B%) of 0–60% B (eluent A: 0.1% HCOOH in H_2_O; eluent B: 0.1% HCOOH in MeCN) within 15 min. The flow rate was 0.2 mL/min.

### GC-MS Analysis

Analysis was carried out on a GC-MS Shimadzu QP 2010 Ultra.

### UV–vis Analysis

Analysis was carried out on an Agilent Technologies Cary 5000 UV–vis–NIR spectrophotometer. The spectrum was recorded in methanol.

### Computational Methodology

The geometry optimization and the subsequent frequency calculations were performed at the B3LYP-D3BJ/def2-TZVP level of theory^60−63^ using the IEF-PCM implementation of the polarizable continuum solvation model^64^ with methanol taken as a solvent. Absence of imaginary frequencies indicated that the correct local minima at the potential energy surface (PES) were found. Frequency calculations allowed for estimation of the bond dissociation enthalpies (BDEs) and the compliance constants of the bonds of interest associated with particular normal modes. Subsequently, the generated wave functions and checkpoint files were used to perform the electronic structure analyses on the basis of the quantum theory of atoms in molecules (QTAIM)^39^ and the reduced density gradient (RDG).^65^ For the first part of the study, the Gaussian 16 C.01 suite of programs^66^ served as a software of choice, whereas the subsequent QTAIM and RDG analyses were conducted with Multiwfn 3.8 dev. software.^67^ Compliance constants were calculated using Compliance 3.0.2 software.^68,69^ The graphical presentation of the obtained results was prepared using VMD 1.9.3 visualization software.^70^

### Reagents

Triethylamine, triisopropylsilane (TIS), 2-bromoethylamine hydrobromide, chloroform-*d*_1_, sodium borohydride, dimethyl sulfoxide-*d*_6_, bromoacetic acid, *N,N′*-diisopropylcarbodiimide (DIC), 4-bromobenzyl bromide, methanol-*d*_4_, and H-Rink Amide ChemMatrix were purchased from Sigma-Aldrich. Di*tert*-butyl dicarbonate and (benzotriazol-1-yloxy)tripyrrolidinophosphonium hexafluorophosphate (PyBOP) were purchased from Novabiochem. Solvents for peptide synthesis (analytical grade) were purchased from Sigma-Aldrich (dimethylformamide) and J. T. Baker (diethyl ether). Trifluoroacetic acid (TFA) and *N*,*N*-diisopropylethylamine (DIEA) were purchased from Iris Biotech. Ethyl acetate and acetic acid were purchased from J. T. Baker. Chloroform and dimethyl sulfoxide were purchased from Chempur. Se powder and ethyl alcohol were purchased from POCH. Amino acids were purchased from PeptideWeb. 4-Methoxybenzyl chloride was purchased from TCI. Solvents for LC-MS and HPLC measurements were as follows: acetonitrile (MeCN), formic acid (HCOOH), and water were purchased from chemsolve and J. T. Baker. The solvent (HPLC grade) for the irradiation experiments and UV–vis measurement was methanol (MeOH), which was purchased from J. T. Baker. The solvent for GC-MS measurement was dichloromethane (DCM), which was purchased from Chemsolve.

#### Synthesis of 2-((*tert*-Butoxycarbonyl)amino)ethyl Bromide^71^

2-Bromoethylamine hydrobromide (2.2 g, 10.7 mmol) was added to a solution of di*tert*-butyl dicarbonate (2.3 g, 10.7 mmol) in DCM (50 mL), followed by the dropwise addition of triethylamine (2.0 mL) at 0 °C. The solution was then warmed to room temperature and stirred overnight. Water was added, and the collected organic layer was washed with water, 1.0 M acetic acid solution, and brine, sequentially. The organic layer was dried over anhydrous MgSO_4_, and the solvent was removed on a rotary evaporator to give a colorless oily product. Yield 1.63 g (68%). ^1^H NMR (500 MHz, CDCl_3_): δ = 4.94 (brs, H), 3.54–3.52 (m, 2H), 3.45 (t, *J* = 5.5 Hz, 2H), 1.45 (s, 9H).

#### Synthesis of Di*tert*-butyl (Diselanediylbis(ethane-2,1-diyl))dicarbamate, **1**(35)

(*The reaction was carried out under a nitrogen atmosphere*.) Ethanol (30 mL) was added to selenium powder (0.6 g, 7.6 mmol) and sodium borohydride (0.2 g, 5.3 mmol), and the mixture was stirred in an ice bath until a vigorous reaction was completed. The reaction mixture was then heated under reflux for 1.5 h by using a heating mantle. 2-((*tert*-Butoxycarbonyl)amino)ethyl bromide (1.1 g, 5.0 mmol) was added, and the solution was allowed to reflux for 4 h. The obtained solution was cooled down to room temperature, water was added, and the aqueous layer was extracted with chloroform (2×). The organic layer was dried over anhydrous MgSO_4_, and the solvent was removed on a rotary evaporator to give a reddish solid product. Yield 0.84 g (75%). ^1^H NMR (500 MHz, DMSO-*d*_6_): δ = 6.96 (brs, 2H), 3.25–3.21 (m, 4H), 2.94 (t, *J* = 7.1 Hz, 4H), 1.37 (s, 18H); ^13^C{^1^H} NMR (126 MHz, DMSO-*d*_6_): δ = 155.9, 78.2, 41.5, 29.4, 28.7; ^77^Se NMR (114 MHz, CDCl_3_): δ = 278.3; calculated *m*/*z* [M + H]^+^: 248.9405; found *m*/*z* [M + H]^+^: 248.9315 for C_4_H_12_N_2_Se_2_.

#### Synthesis of 1,2-Bis(4-methoxybenzyl)diselane^35^

(*The reaction was carried out under a nitrogen atmosphere*.) Ethanol (30 mL) was added to selenium powder (0.6 g, 7.6 mmol) and sodium borohydride (0.2 g, 5.3 mmol), and the mixture was stirred in an ice bath until a vigorous reaction was completed. The reaction mixture was then heated under reflux for 1.5 h by using a heating mantle. 4-Methoxybenzyl chloride (0.68 mL, 5.0 mmol) was added, and the solution was allowed to reflux for 4 h. The obtained solution was cooled down to room temperature, water was added, and the aqueous layer was extracted with chloroform (2×). The organic layer was dried over anhydrous MgSO_4_, and the solvent was removed on a rotary evaporator to give an orange solid product. Yield 0.81 g (81%). ^1^H NMR (500 MHz, DMSO-*d*_6_): δ = 7.19–7.16 (m, 4H), 6.89–6.86 (m, 4H), 3.89 (s, 4H), 3.73 (s, 6H); ^13^C{^1^H} NMR (126 MHz, DMSO-*d*_6_): δ = 158.9, 131.4, 130.6, 114.3, 55.6, 31.8; ^77^Se NMR (114 MHz, CDCl_3_): δ = 396.8.

#### Synthesis of 2-(4-Methoxybenzylseleno)ethylamine, **2**(72)

(*The reaction was carried out under a nitrogen atmosphere*.) Sodium borohydride (0.4 g, 10.6 mmol) was added portionwise to a solution of 1,2-bis(4-methoxybenzyl)diselane (1.0 g, 2.5 mmol) in EtOH/DMF (30 mL, 1:1, v/v), and the reaction mixture was stirred for 2 h at room temperature. 2-Bromoethylamine hydrobromide (1.3 g, 6.3 mmol) was then dissolved in EtOH (5 mL) and added dropwise at 0 °C. The solution was warmed to room temperature and stirred overnight. The solvent was removed to dryness under a stream of nitrogen. The obtained product was dissolved in a saturated aqueous solution of NaHCO_3_, and the aqueous layer was extracted with ethyl acetate (3×). The organic layer was washed with brine and dried over anhydrous MgSO_4_. The solvent was removed on a rotary evaporator to give a white solid product. Yield 0.55 g (90%). ^1^H NMR (500 MHz, MeOD): δ = 7.24–7.22 (m, 2H), 6.85–6.83 (m, 2H), 3.78 (s, 2H), 3.77 (s, 3H), 2.80 (t, *J* = 6.9 Hz, 2H), 2.60 (t, *J* = 6.9 Hz, 2H); ^13^C{^1^H} NMR (126 MHz, MeOD): δ = 158.7, 131.3, 129.6, 113.5, 54.3, 40.6, 25.5, 25.0; ^77^Se NMR (114 MHz, MeOD): δ = 217.8; calculated *m*/*z* [M + H]^+^: 246.0392; found *m*/*z* [M + H]^+^: 246.0344 for C_10_H_15_NOSe.

#### Synthesis of 1,2-Bis(4-bromobenzyl)diselane, **BBSe_2_**(35)

(*The reaction was carried out under a nitrogen atmosphere*.) Ethanol (30 mL) was added to selenium powder (0.6 g, 7.6 mmol) and sodium borohydride (0.2 g, 5.3 mmol), and the mixture was stirred in an ice bath until a vigorous reaction was completed. The reaction mixture was then heated under reflux for 1.5 h by using a heating mantle. 4-Bromobenzyl bromide (1.2 g, 5.0 mmol) was added, and the solution was allowed to reflux for 4 h. The obtained solution was cooled down to room temperature, water was added, and the aqueous layer was extracted with chloroform (2×). The organic layer was dried over anhydrous MgSO_4_, and the solvent was removed on a rotary evaporator to give a yellowish-green solid product. Yield 0.96 g (77%). ^1^H NMR (500 MHz, DMSO-*d*_6_): δ = 7.52–7.49 (m, 4H), 7.20–7.17 (m, 4H), 3.94 (s, 4H); ^13^C{^1^H} NMR (126 MHz, DMSO-*d*_6_): δ = 139.2, 131.7, 131.5, 120.5, 31.0; ^77^Se NMR (114 MHz, CDCl_3_): δ = 409.7.

### General Procedure for Peptide Synthesis

Synthesis of peptides was carried out on H-Rink Amide ChemMatrix (0.40–0.60 mmol/g) according to the standard Fmoc protocol. 100 mg of the resin was placed in a syringe and swollen in DMF for 30 min on a rotary mixer at room temperature. The amino acid coupling reaction was then performed with Fmoc-Xaa (3 equiv), PyBOP (3 equiv), and DIEA (6 equiv) in DMF for 20 min in an ultrasonic bath. After the coupling step, the peptidyl resin was washed with DMF (6 × 1 min), and Fmoc removal was carried out with 25% piperidine in DMF (v/v) for 4 min in an ultrasonic bath.^73^ After Fmoc removal, the peptidyl resin was washed with DMF (7 × 1 min) and incubated with bromoacetic acid (5 equiv) and DIC (5 equiv) in DMF for 30 min (3×) on a rotary mixer at room temperature. Upon completion of the bromoacetylation step, **1** or **2** was incorporated and the peptidyl resin was then washed with DMF (10 × 1 min). After incorporation of the building blocks, the first incoming amino acid was coupled twice and amino acid coupling was continued until the desired sequence was obtained. After the peptide synthesis was completed, the peptidyl resin was washed with DCM (3 × 1 min), THF (3 × 1 min), and Et_2_O (3 × 1 min) and dried overnight in a vacuum desiccator. Cleavage of the peptide from the resin was performed with TFA/H_2_O/TIS (95:2.5:2.5, v/v/v) for 3 h at room temperature. After evaporating the solvent under a stream of nitrogen, the obtained product was subjected to lyophilization and then purified by HPLC. The bromoacetylation step was performed twice at different positions of the peptide chain to synthesize cyclic peptides.

**Synthesis of linear and cyclic peptides via incorporation of1and/or2** For the incorporation of **1**, di*tert*-butyl (diselanediylbis(ethane-2,1-diyl))dicarbamate was treated with TFA/DCM (1:1, v/v) for 1 h at room temperature to remove the Boc-protecting group, and the solvent was removed under a stream of nitrogen. The bromoacetylated peptidyl resin was then incubated with the deprotected compound (3 equiv) and DIEA (12 equiv) in DMF on a rotary mixer overnight at room temperature. For the incorporation of **2**, the bromoacetylated peptidyl resin was incubated with 2-(4-methoxybenzylseleno)ethylamine (3 equiv) in DMF on a rotary mixer overnight at room temperature. After the cleavage process, the obtained product was treated with 5% DMSO in TFA (v/v) for 6 h to remove the *p*-methoxybenzyl protecting group and then precipitated in Et_2_O (Table 2).

**Table 2:** List of Synthesized Peptides

sequence	abbreviation
[H-FG-*N*-(2-selenoethyl)glycine-AK-NH_2_]_2_	**Linear(Se**–**Se)1**
[H-FAG-*N*-(2-selenoethyl)glycine-KAI-NH_2_]_2_	**Linear(Se**–**Se)2**
[H-WG-*N*-(2-selenoethyl)glycine-KG-NH_2_]_2_	**Linear(Se**–**Se)3**
cycloIAKG-*N*-(2-selenoethyl)glycine-AAG-*N*-(2-selenoethyl)glycine-KAI	**Cyclo(Se**–**Se)1**
cycloKAG-*N*-(2-selenoethyl)glycine-AG-*N*-(2-selenoethyl)glycine-AK	**Cyclo(Se**–**Se)2**

### Synthesis of **Linear(Se–Se)1**

The synthesis of the peptide was performed using **2** as the building block. The peptide was obtained as a lyophilized white powder. Yield: 46.92 mg (67%); HPLC (retention time): 8.56 min; ESI-qTOF-MS: *m*/*z* [M + H]^+^: 1169.4286; *m*/*z* [M+H+K]^2+^: 604.1915; *m*/*z* [M+2H]^2+^: 585.2176; *m*/*z* y_4_: 511.6830, *z* = 2+; *m*/*z* y_3_: 483.1756, *z* = 2+; *m*/*z* [M+3H]^3+^: 390.4809; calculated *m*/*z* [M + H]^+^: 1169.4285; *m*/*z* [M+H+K]^2+^: 604.1958; *m*/*z* [M+2H]^2+^: 585.2179; *m*/*z* y_4_: 511.6835, *z* = 2+; *m*/*z* y_3_: 483.1727, *z* = 2+; *m*/*z* [M+3H]^3+^: 390.4810; ESI-qTOF-MS/MS: precursor ion at *m*/*z* [M+2H]^2+^: 585.2360 (calculated for M: 585.2179, *z* = 2+); collision energy 20 eV; *m*/*z* [M + H]^+^: 1024.3436 (calculated for b_4_: 1024.3067, *z* = +); *m*/*z* [M + H]^+^: 948.3393 (calculated for b_5_y_3_: 948.3116, *z* = +); *m*/*z* [M + H]^+^: 877.2637 (calculated for b_4_y_4_: 877.2380, *z* = +); *m*/*z* [M + H]^+^: 820.2404 (calculated for b_4_y_3_: 820.2165, *z* = +); *m*/*z* [M+2H]^2+^: 576.7233 (calculated for M-NH_3_: 576.7046, *z* = 2+); *m*/*z* [M+2H]^2+^: 511.7004 (calculated for y_4_: 511.6835, *z* = 2+); *m*/*z* [M+2H]^2+^: 503.1865 (calculated for y_4_-NH_3_: 503.1702, *z* = 2+); *m*/*z* [M+2H]^2+^: 483.1892 (calculated for y_3_: 483.1727, *z* = 2+); *m*/*z* [M+2H]^2+^: 474.6754 (calculated for y_3_-NH_3_: 474.6595, *z* = 2+).

### **LM1A**

ESI-qTOF-MS: *m*/*z* found [M + H]^+^: 834.1035; *m*/*z* calculated [M + H]^+^: 834.0992; ESI-qTOF-MS/MS: precursor ion at *m*/*z* [M + H]^+^: 834.1153 (calculated for M: 834.0992, *z* = +); collision energy 30 eV; *m*/*z* [M + H]^+^: 817.0905 (calculated for M-NH_3_: 817.0726, *z* = +); *m*/*z* [M + H]^+^: 665.1482 (calculated for CSe: 665.1342, *z* = +); *m*/*z* [M + H]^+^: 630.0220 (calculated for y_3_: 630.0090, *z* = +); *m*/*z* [M + H]^+^: 617.9543 (calculated for b_3_: 617.9403, *z* = +).

### **LM1B**

ESI-qTOF-MS: *m*/*z* found [M + H]^+^: 754.1867; *m*/*z* calculated [M + H]^+^: 754.1825; ESI-qTOF-MS/MS: precursor ion at *m*/*z* [M + H]^+^: 754.2008 (calculated for M: 754.1825, *z* = +); collision energy 30 eV; *m*/*z* [M + H]^+^: 592.1119 (calculated for b_4_-NH_3_: 592.0343, *z* = +).

### Synthesis of **Linear(Se–Se)2**

The synthesis of the peptide was performed using **1** as the building block. The peptide was obtained as a lyophilized white powder. Yield: 67.26 mg (73%); HPLC (retention time): 8.99 min; ESI-qTOF-MS: *m*/*z* [M + H]^+^: 1537.6871; *m*/*z* [M+H+K]^2+^: 788.3203; *m*/*z* [M+2H]^2+^: 769.3466; *m*/*z* [M+2H+K]^3+^: 525.8826; *m*/*z* [M+3H]^3+^: 513.2335; calculated *m*/*z* [M + H]^+^: 1537.6716; *m*/*z* [M+H+K]^2+^: 788.3173; *m*/*z* [M+2H]^2+^: 769.3394; *m*/*z* [M+2H+K]^3+^: 525.8806; *m*/*z* [M+3H]^3+^: 513.2287; ESI-qTOF-MS/MS: precursor ion at *m*/*z* [M+3H]^3+^: 512.5659 (calculated for M: 512.5618, *z* = 3+); collision energy 15 eV; *m*/*z* [M+2H]^2+^: 704.2841 (calculated for b_6_: 704.2840, *z* = 2+); *m*/*z* [M+2H]^2+^: 695.8007 (calculated for y_6_: 695.8050, *z* = 2+); *m*/*z* [M+2H]^2+^: 687.2906 (calculated for y_6_-NH_3_: 687.2918, *z* = 2+); *m*/*z* [M+2H]^2+^: 659.2865 (calculated for y_5_: 659.2862, *z* = 2+); *m*/*z* [M+2H]^2+^: 651.7752 (calculated for y_5_-NH_3_: 651.7731, *z* = 2+); *m*/*z* [M+2H]^2+^: 595.2343 (calculated for b_5_y_6_: 595.2387, *z* = 2+); *m*/*z* [M+3H]^3+^: 506.8912 (calculated for M-NH_3_: 506.8862, *z* = 3+); *m*/*z* [M+3H]^3+^: 497.5602 (calculated for M-NH_3_–CO: 497.5546, *z* = 3+); *m*/*z* [M+3H]^3+^: 491.8839 (calculated for M-2NH_3_–CO: 491.8791, *z* = 3+); *m*/*z* [M+3H]^3+^: 439.8678 (calculated for y_5_: 439.8599, *z* = 3+).

### **LM2A**

ESI-qTOF-MS: *m*/*z* found [M + H]^+^: 1018.2230; *m*/*z* calculated [M + H]^+^: 1018.2207; ESI-qTOF-MS/MS: precursor ion at *m*/*z* [M+2H]^2+^: 510.6138 (calculated for M: 510.6129, *z* = 2+); collision energy 20 eV; *m*/*z* [M + H]^+^: 888.1130 (calculated for b_6_: 888.1099, *z* = +); *m*/*z* [M + H]^+^: 871.1542 (calculated for b_6_-NH_3_: 871.0833, *z* = +); *m*/*z* [M + H]^+^: 800.1178 (calculated for b_5_-NH_3_: 800.0461, *z* = +); *m*/*z* [M+2H]^2+^: 502.1016 (calculated for M-NH_3_: 502.0996, *z* = 2+); *m*/*z* [M+2H]^2+^: 488.1037 (calculated for M-NH_3_–CO: 488.1022, *z* = 2+).

### **LM2B**

ESI-qTOF-MS: *m*/*z* found [M + H]^+^: 938.3363; *m*/*z* calculated [M + H]^+^: 938.3039; ESI-qTOF-MS/MS: precursor ion at *m*/*z* [M + H]^+^: 940.3073 (calculated for M: 940.3019, *z* = +); collision energy 20 eV; *m*/*z* [M + H]^+^: 923.2812 (calculated for M-NH_3_: 923.2754, *z* = +); *m*/*z* [M + H]^+^: 895.2818 (calculated for M-NH_3_–CO: 895.2804, *z* = +); *m*/*z* [M + H]^+^: 810.1961 (calculated for b_6_: 810.1913, *z* = +); *m*/*z* [M + H]^+^: 792.1891 (calculated for b_6_-H_2_O: 792.1807, *z* = +); *m*/*z* [M + H]^+^: 782.1957 (calculated for b_6_-CO: 782.1963, *z* = +); *m*/*z* [M + H]^+^: 739.1591 (calculated for b_5_: 739.1541, *z* = +); *m*/*z* [M + H]^+^: 722.1991 (calculated for b_5_-NH_3_: 722.1276, *z* = +); *m*/*z* [M + H]^+^: 705.1738 (calculated for b_5_-2NH_3_: 705.1010, *z* = +); *m*/*z* [M + H]^+^: 665.1774 (calculated for y_4_: 665.1748, *z* = +); *m*/*z* [M + H]^+^: 648.1519 (calculated for y_4_-NH_3_: 648.1483, *z* = +); *m*/*z* [M + H]^+^: 611.0632 (calculated for b_4_: 611.0591, *z* = +); *m*/*z* [M + H]^+^: 330.2520 (calculated for y_3_: 330.2500, *z* = +).

### LM12

ESI-qTOF-MS: *m*/*z* found [M+3H]^3+^: 451.8551; *m*/*z* calculated [M+3H]^3+^: 451.8549; ESI-qTOF-MS/MS: precursor ion at *m*/*z* [M+2H]^2+^: 677.2999 (calculated for M: 677.2786, *z* = 2+); collision energy 25 eV; *m*/*z* [M+2H]^2+^: 668.7863 (calculated for M-NH_3_: 668.7654, *z* = 2+); *m*/*z* [M+2H]^2+^: 612.2436 (calculated for b_6_: 612.2232, *z* = 2+); *m*/*z* [M+2H]^2+^: 603.7571 (calculated for y_4_* and y_6_: 603.7443, *z* = 2+); *m*/*z* [M+2H]^2+^: 575.2466 (calculated for y_3_*: 575.2335, *z* = 2+); *m*/*z* [M+2H]^2+^: 568.2416 (calculated for y_5_: 568.2256, *z* = 2+); *m*/*z* [M+3H]^3+^: 451.8688 (calculated for M: 451.8549, *z* = 3+).

### Synthesis of **Linear(Se–Se)3**

The synthesis of the peptide was performed using **1** as the building block. The peptide was obtained as a lyophilized white powder. Yield: 47.47 mg (65%); LC-MS (retention time): 8.60 min; LC-ESI-IT-TOF-MS: *m*/*z* [M+2H]^2+^: 610.1874; *m*/*z* [M+3H]^3+^: 407.1247; *m*/*z* [M+4H]^4+^: 305.0945; calculated *m*/*z* [M+2H]^2+^: 610.2132; *m*/*z* [M+3H]^3+^: 407.1445; *m*/*z* [M+4H]^4+^: 305.1102; ESI-qTOF-MS/MS: precursor ion at *m*/*z* [M+2H]^2+^: 609.2061 (calculated for M: 609.2130, *z* = 2+); collision energy 20 eV; *m*/*z* [M + H]^+^: 1033.3253 (calculated for y_4_: 1033.3393, *z* = 1+); *m*/*z* [M + H]^+^: 1016.2966 (calculated for y_4_-NH_3_: 1016.3128, *z* = 1+); *m*/*z* [M + H]^+^: 976.3045 (calculated for y_3_: 976.3178, *z* = 1+); *m*/*z* [M + H]^+^: 959.2781 (calculated for y_3_-NH_3_: 959.2912, *z* = 1+); *m*/*z* [M+2H]^2+^: 600.6932 (calculated for M-NH_3_: 600.6998, *z* = 2+); *m*/*z* [M+2H]^2+^: 487.6575 (calculated for y_3_: 487.6626, *z* = 2+); *m*/*z* [M+2H]^2+^: 479.1444 (calculated for y_3_-NH_3_: 479.1493, *z* = 2+).

### **LM3A**

ESI-qTOF-MS: *m*/*z* found [M + H]^+^: 859.0939; *m*/*z* calculated [M + H]^+^: 859.0945; ESI-qTOF-MS/MS: precursor ion at *m*/*z* [M + H]^+^: 859.0841 (calculated for M: 859.0945, *z* = +); collision energy 20 eV; *m*/*z* [M + H]^+^: 842.0597 (calculated for M-NH_3_: 842.0679, *z* = 1+); *m*/*z* [M + H]^+^: 690.1219 (calculated for CSe: 690.1295, *z* = 1+); *m*/*z* [M + H]^+^: 673.0134 (calculated for y_4_: 673.0149, *z* = 1+); *m*/*z* [M + H]^+^: 656.9455 (calculated for b_3_: 656.9513, *z* = 1+); *m*/*z* [M + H]^+^: 615.9870 (calculated for y_3_: 615.9933, *z* = 1+); *m*/*z* [M + H]^+^: 541.9404 (calculated for b_4_y_3_: 541.9453, *z* = 1+).

### **LM3B**

ESI-qTOF-MS: *m*/*z* found [M + H]^+^: 779.1658; *m*/*z* calculated [M + H]^+^: 779.1778; ESI-qTOF-MS/MS: precursor ion at *m*/*z* [M+2H]^2+^: 390.0951 (calculated for M: 390.0925, *z* = 2+); collision energy 20 eV; *m*/*z* [M + H]^+^: 611.2241 (calculated for M-C_7_H_5_Br: 611.2205, *z* = 1+); *m*/*z* [M + H]^+^: 594.1991 (calculated for M-C_7_H_5_Br-NH_3_: 594.1939, *z* = 1+); *m*/*z* [M + H]^+^: 536.0820 (calculated for y_3_: 536.0768, *z* = 1+); *m*/*z* [M+2H]^2+^: 381.5822 (calculated for M-NH_3_: 381.5792, *z* = 2+).

### Synthesis of **Cyclo(Se–Se)1**

The synthesis of the peptide was performed using **2** as the building block. The peptide was obtained as a lyophilized white powder. Yield: 55.09 mg (75%); HPLC (retention time): 8.77 min; LC-ESI-IT-TOF-MS: *m*/*z* [M+2H]^2+^: 613.7562; *m*/*z* [M-NH_3_+2H]^2+^: 605.2439; *m*/*z* y_11:_ 557.2146, *z* = 2+; *m*/*z* y_11_-NH_3_ and b_11:_ 548.7027, *z* = 2+; *m*/*z* y_11_-NH_3_–CO and b_11_-CO: 534.6987, *z* = 2+; *m*/*z* y_10:_ 521.6978, *z* = 2+; *m*/*z* [M+3H]^3+^: 409.5077; *m*/*z* [M-NH_3_+3H]^3+^: 403.8327; *m*/*z* [M-NH_3_–CO+3H]^3+^: 394.5023; calculated *m*/*z* [M+2H]^2+^: 613.7574; *m*/*z* [M-NH_3_+2H]^2+^: 605.2441; *m*/*z* y_11_: 557.2152, *z* = 2+; *m*/*z* y_11_-NH_3_ and b_11:_ 548.7020, *z* = 2+; *m*/*z* y_11_-NH_3_–CO and b_11_-CO: 534.7045, *z* = 2+; *m*/*z* y_10_: 521.6966, *z* = 2+; *m*/*z* [M+3H]^3+^: 409.5073; *m*/*z* [M-NH_3_+3H]^3+^: 403.8318; *m*/*z* [M-NH_3_–CO+3H]^3+^: 394.5002; ESI-qTOF-MS: *m*/*z* [M+3H]^3+^: 409.5066; calculated *m*/*z* [M+3H]^3+^: 409.5073; MALDI-MS: *m*/*z* [M+K]^+^: 1264.444; *m*/*z* [M + Na]^+^: 1248.470; *m*/*z* [M + H]^+^: 1226.489; calculated *m*/*z* [M+K]^+^: 1264.463; *m*/*z* [M + Na]^+^: 1248.489; *m*/*z* [M + H]^+^: 1226.507; ESI-qTOF-MS/MS: precursor ion at *m*/*z* [M+3H]^3+^: 409.5174 (calculated for M: 409.5073, *z* = 3+); collision energy 15 eV; *m*/*z* [M+2H]^2+^: 605.2468 (calculated for M-NH_3_: 605.2441, *z* = 2+); *m*/*z* [M+2H]^2+^: 557.2171 (calculated for y_11_: 557.2152, *z* = 2+); *m*/*z* [M+2H]^2+^: 548.7048 (calculated for y_11_-NH_3_ and b_11_: 548.7020, *z* = 2+); *m*/*z* [M+2H]^2+^: 534.7068 (calculated for y_11_-NH_3_–CO and b_11_-CO: 534.7045, *z* = 2+); *m*/*z* [M+2H]^2+^: 521.7005 (calculated for y_10_: 521.6966, *z* = 2+); *m*/*z* [M+2H]^2+^: 513.1910 (calculated for y_10_-NH_3_: 513.1833, *z* = 2+); *m*/*z* [M+2H]^2+^: 499.1900 (calculated for y_10_-NH_3_–CO: 499.1859, *z* = 2+); *m*/*z* [M+3H]^3+^: 403.8431 (calculated for M-NH_3_: 403.8318, *z* = 3+); *m*/*z* [M+3H]^3+^: 394.5119 (calculated for M-NH_3_–CO: 394.5002, *z* = 3+).

### **C1T**

ESI-qTOF-MS: *m*/*z* found [M+3H]^3+^: 436.1475; *m*/*z* calculated [M+3H]^3+^: 436.1458; ESI-qTOF-MS/MS: precursor ion at *m*/*z* [M+3H]^3+^: 435.4863 (calculated for M: 435.4800, *z* = 3+); collision energy 15 eV; *m*/*z* [M+2H]^2+^: 644.2015 (calculated for M-NH_3_: 644.2030, *z* = 2+); *m*/*z* [M+2H]^2+^: 596.1734 (calculated for y_11_: 596.1741, *z* = 2+); *m*/*z* [M+2H]^2+^: 587.6604 (calculated for y_11_-NH_3_ and b_11_: 587.6609, *z* = 2+); *m*/*z* [M+2H]^2+^: 573.6632 (calculated for y_11_-NH_3_–CO and b_11_-CO: 573.6634, *z* = 2+); *m*/*z* [M+2H]^2+^: 560.6554 (calculated for y_10_: 560.6555, *z* = 2+); *m*/*z* [M+3H]^3+^: 429.8119 (calculated for M-NH_3_: 429.8044, *z* = 3+); *m*/*z* [M+3H]^3+^: 420.4804 (calculated for M-NH_3_–CO: 420.4728, *z* = 3+).

### Dimer Form of **Cyclo(Se–Se)1**

ESI-qTOF-MS: *m*/*z* found [M+5H]^5+^: 491.2072; *m*/*z* calculated [M+5H]^5+^: 491.2079; ESI-qTOF-MS/MS: precursor ion at *m*/*z* [M+5H]^5+^: 491.2143 (calculated for M: 491.2079, *z* = 5+); collision energy 15 eV; *m*/*z* [M+4H]^4+^: 584.9939 (calculated for y_11_: 584.9869, *z* = 4+); *m*/*z* [M+5H]^5+^: 487.8086 (calculated for M-NH_3_: 487.8025, *z* = 5+); *m*/*z* [M+5H]^5+^: 482.2094 (calculated for M-NH_3_–CO: 482.2035, *z* = 5+); *m*/*z* [M+3H]^3+^: 409.5125 (calculated for SeSe_1_SeSe_2_ (monomer): 409.5073, *z* = 3+).

### **CM1A**

ESI-qTOF-MS: *m*/*z* found [M+3H]^3+^: 575.4293; *m*/*z* calculated [M+3H]^3+^: 575.4286; ESI-qTOF-MS/MS: precursor ion at *m*/*z* [M+3H]^3+^: 575.4255 (calculated for M: 575.4286, *z* = 3+); collision energy 15 eV; *m*/*z* [M + H]^+^: 1412.0526 (calculated for y_9_: 1412.0544, *z* = +); *m*/*z* [M + H]^+^: 942.1924 (calculated for y_7_: 942.1892, *z* = +); *m*/*z* [M + H]^+^: 925.1619 (calculated for y_7_-NH_3_ and b_7_: 925.1626, *z* = +); *m*/*z* [M+2H]^2+^: 806.0954 (calculated for y_11_: 806.0971, *z* = 2+); *m*/*z* [M+2H]^2+^: 797.5821 (calculated for y_11_-NH_3_ and b_11_: 797.5838, *z* = 2+); *m*/*z* [M+2H]^2+^: 783.5845 (calculated for y_11_-NH_3_–CO and b_11_-CO: 783.5863, *z* = 2+); *m*/*z* [M+2H]^2+^: 770.5773 (calculated for y_10_: 770.5785, *z* = 2+); *m*/*z* [M+3H]^3+^: 435.4781 (calculated for CSe_1_SeSe_2_ and CSe_2_SeSe_1_ (cyclic triselenide): 435.4800, *z* = 3+); *m*/*z* [M+3H]^3+^: 409.5066 (calculated for SeSe_1_SeSe_2_ (monomer): 409.5073, *z* = 3+); *m*/*z* [M+3H]^3+^: 394.4996 (calculated for monomer-NH_3_–CO: 394.5002, *z* = 3+).

### **CM1B**

ESI-qTOF-MS: *m*/*z* found [M+3H]^3+^: 548.7868; *m*/*z* calculated [M+3H]^3+^: 548.7896; ESI-qTOF-MS/MS: precursor ion at *m*/*z* [M+3H]^3+^: 549.4602 (calculated for M: 549.4560, *z* = 3+); collision energy 15 eV; *m*/*z* [M+2H]^2+^: 766.1436 (calculated for y_11_: 766.1387, *z* = 2+); *m*/*z* [M+2H]^2+^: 757.6248 (calculated for y_11_-NH_3_ and b_11_: 757.6254, *z* = 2+); *m*/*z* [M+2H]^2+^: 653.7135 (calculated for CSe_1_CSe_2_ (cyclic triselenide): 653.7151, *z* = 2+); *m*/*z* [M+2H]^2+^: 613.7588 (calculated for CSe_1_SeSe_2_ (monomer): 613.7574, *z* = 2+); *m*/*z* [M+3H]^3+^: 543.7865 (calculated for M-NH_3_: 543.7805, *z* = 3+); *m*/*z* [M+3H]^3+^: 534.4525 (calculated for M-NH_3_–CO: 534.4489, *z* = 3+); *m*/*z* [M+3H]^3+^: 436.1545 (calculated for CSe_1_CSe_2_ (cyclic triselenide): 436.1458, *z* = 3+); *m*/*z* [M+3H]^3+^: 409.5181 (calculated for CSe_1_SeSe_2_ (monomer): 409.5073, *z* = 3+).

### **CM1C**

ESI-qTOF-MS: *m*/*z* found [M+3H]^3+^: 522.1500; *m*/*z* calculated [M+3H]^3+^: 522.1507; ESI-qTOF-MS/MS: precursor ion at *m*/*z* [M+3H]^3+^: 522.1575 (calculated for M: 522.1507, *z* = 3+); collision energy 15 eV; *m*/*z* [M+2H]^2+^: 612.7599 (calculated for CSe_1_CSe_2_ (monomer): 612.7573, *z* = 2+); *m*/*z* [M+3H]^3+^: 409.5192 (calculated for CSe_1_CSe_2_ (monomer): 409.5073, *z* = 3+).

### Synthesis of **Cyclo(Se–Se)2**

The synthesis of the peptide was performed using **2** as the building block. The peptide was obtained as a lyophilized white powder. Yield: 38.37 mg (69%); HPLC (retention time): 6.65 min; LC-ESI-IT-TOF-MS: *m*/*z* [M + Na]^+^: 951.2844; *m*/*z* [M + H]^+^: 929.3024; *m*/*z* [M+2H]^2+^: 465.1544; *m*/*z* [M+3H]^3+^: 310.4383; calculated *m*/*z* [M + Na]^+^: 951.2836; *m*/*z* [M + H]^+^: 929.3017; *m*/*z* [M+2H]^2+^: 465.1545; *m*/*z* [M+3H]^3+^: 310.4387; ESI-qTOF-MS: *m*/*z* [M + H]^+^: 929.3034; *m*/*z* [M+H+K]^2+^: 484.1307; *m*/*z* [M+2H]^2+^: 465.1573; calculated *m*/*z* [M + H]^+^: 929.3017; *m*/*z* [M+H+K]^2+^:484.1324; *m*/*z* [M+2H]^2+^:465.1545; MALDI-MS: *m*/*z* [M+K]^+^: 967.248; *m*/*z* [M + Na]^+^: 951.271; *m*/*z* [M + H]^+^: 929.281; calculated *m*/*z* [M+K]^+^: 967.258; *m*/*z* [M + Na]^+^: 951.284; *m*/*z* [M + H]^+^: 929.302; ESI-qTOF-MS/MS: precursor ion at *m*/*z* [M+2H]^2+^: 464.1586 (calculated for M: 464.1546, *z* = 2+); collision energy 15 eV; *m*/*z* [M + H]^+^: 801.1989 (calculated for y_8_: 801.2066, *z* = +); *m*/*z* [M + H]^+^: 784.1728 (calculated for y_8_-NH_3_ and b_8_: 784.1800, *z* = +); *m*/*z* [M + H]^+^: 756.1741 (calculated for y_8_-NH_3_–CO and b_8_-CO: 756.1851, *z* = +); *m*/*z* [M + H]^+^: 730.1634 (calculated for y_7_: 730.1694, *z* = +); *m*/*z* [M + H]^+^: 713.1369 (calculated for y_7_-NH_3_ and b_7_: 713.1428, *z* = +); *m*/*z* [M + H]^+^: 673.1411 (calculated for y_6_: 673.1478, *z* = +); *m*/*z* [M + H]^+^: 656.0986 (calculated for y_6_-NH_3_: 656.1213, *z* = +); *m*/*z* [M + H]^+^: 585.0482 (calculated for b_8_y_7_: 585.0477, *z* = +); *m*/*z* [M+2H]^2+^: 455.6461 (calculated for M-NH_3_: 455.6413, *z* = 2+); *m*/*z* [M+2H]^2+^: 446.6387 (calculated for M-NH_3_–H_2_O: 446.6360, *z* = 2+); *m*/*z* [M+2H]^2+^: 357.0886 (calculated for b_7_: 357.0750, *z* = 2+); *m*/*z* [M+2H]^2+^: 343.0804 (calculated for b_7_-CO: 343.0776, *z* = 2+).

### **C2T**

ESI-qTOF-MS: *m*/*z* found [M+2H]^2+^: 505.1133; *m*/*z* calculated [M+2H]^2+^: 505.1125; ESI-qTOF-MS/MS: precursor ion at *m*/*z* [M+2H]^2+^: 505.1113 (calculated for M: 505.1125, *z* = 2+); collision energy 15 eV; *m*/*z* [M + H]^+^: 881.1274 (calculated for y_8_: 881.1227, *z* = +); *m*/*z* [M + H]^+^: 864.0955 (calculated for y_8_-NH_3_ and b_8_: 864.0961, *z* = +); *m*/*z* [M+2H]^2+^: 465.1521 (calculated for monomer: 465.1545, *z* = 2+).

### Dimer Form of **Cyclo(Se–Se)2**

ESI-qTOF-MS: *m*/*z* found [M+3H]^3+^: 619.8677; *m*/*z* calculated [M+3H]^3+^: 619.8709; ESI-qTOF-MS/MS: precursor ion at *m*/*z* [M+3H]^3+^: 619.2038 (calculated for M: 619.2043, *z* = 3+); collision energy 15 eV; *m*/*z* [M+2H]^2+^: 464.1594 (calculated for SeSe_1_SeSe_2_ (monomer): 464.1546, *z* = 2+).

### **CM2A**

ESI-qTOF-MS: *m*/*z* found [M+2H]^2+^: 714.0343; *m*/*z* calculated [M+2H]^2+^: 714.0363; ESI-qTOF-MS/MS: precursor ion at *m*/*z* [M+2H]^2+^: 714.0400 (calculated for M: 714.0363, *z* = 2+); collision energy 20 eV; *m*/*z* [M + H]^+^: 1298.9787 (calculated for y_8_: 1298.9701, *z* = +); *m*/*z* [M + H]^+^: 1281.9593 (calculated for y_8_-NH_3_ and b_8_: 1281.9436, *z* = +); *m*/*z* [M + H]^+^: 1227.9423 (calculated for y_7_: 1227.9329, *z* = +); *m*/*z* [M + H]^+^: 879.1350 (calculated for cyclic triselenide (y_8_): 879.1243, *z* = +); *m*/*z* [M + H]^+^: 862.1069 (calculated for cyclic triselenide (y_8_-NH_3_) and (b_8_): 862.0978, *z* = +); *m*/*z* [M + H]^+^: 808.0954 (calculated for cyclic triselenide (y_7_): 808.0871, *z* = +); *m*/*z* [M + H]^+^: 801.2141 (calculated for monomer (y_8_): 801.2066, *z* = +); *m*/*z* [M + H]^+^: 784.1875 (calculated for monomer (y_8_-NH_3_) and (b_8_): 784.1800, *z* = +); *m*/*z* [M + H]^+^: 730.1759 (calculated for monomer (y_7_): 730.1694, *z* = +); *m*/*z* [M+2H]^2+^: 504.1160 (calculated for SeSe_1_CSe_2_ and SeSe_2_CSe_1_ (cyclic triselenide): 504.1134, *z* = 2+); *m*/*z* [M+2H]^2+^: 495.6025 (calculated for cyclic triselenide-NH_3_: 495.6001, *z* = 2+); *m*/*z* [M+2H]^2+^: 465.1574 (calculated for SeSe_1_SeSe_2_ (monomer): 465.1545, *z* = 2+); *m*/*z* [M+2H]^2+^: 456.6440 (calculated for monomer-NH_3_: 456.6412, *z* = 2+).

### **CM2B**

ESI-qTOF-MS: *m*/*z* found [M+2H]^2+^: 674.0750; *m*/*z* calculated [M+2H]^2+^: 674.0779; ESI-qTOF-MS/MS: precursor ion at *m*/*z* [M+2H]^2+^: 674.0803 (calculated for M: 674.0779, *z* = 2+); collision energy 15 eV; *m*/*z* [M + H]^+^: 1007.2219 (calculated for CSe_1_CSe_2_ (cyclic triselenide): 1007.2195, *z* = +); *m*/*z* [M + H]^+^: 927.3036 (calculated for SeSe_1_CSe_2_ (monomer): 927.3019, *z* = +); *m*/*z* [M+2H]^2+^: 504.1189 (calculated for CSe_1_CSe_2_ (cyclic triselenide): 504.1134, *z* = 2+); *m*/*z* [M+2H]^2+^: 465.1623 (calculated for SeSe_1_CSe_2_ (monomer): 465.1545, *z* = 2+).

### **CM2C**

ESI-qTOF-MS: *m*/*z* found [M+2H]^2+^: 634.1174; *m*/*z* calculated [M+2H]^2+^: 634.1196; ESI-qTOF-MS/MS: precursor ion at *m*/*z* [M+2H]^2+^: 635.1164 (calculated for M: 635.1190, *z* = 2+); collision energy 20 eV; *m*/*z* [M + H]^+^: 929.2958 (calculated for CSe_1_CSe_2_ (monomer): 929.3017, *z* = +); *m*/*z* [M+2H]^2+^: 465.1548 (calculated for CSe_1_CSe_2_ (monomer): 465.1545, *z* = 2+); *m*/*z* [M+2H]^2+^: 456.6404 (calculated for monomer-NH_3_: 456.6412, *z* = 2+).

### Irradiation Experiments

A solution (equimolar, 5 mM) of peptide/peptide or **BBSe**_**2**_/peptide in MeOH (400 μL) was placed in an HPLC vial, and the HPLC vial caps were wrapped in parafilm. The LED lamp (13W, 4000K, LEDVANCE GmbH) was placed at a distance of 1.3 cm from the surface of the HPLC vial. The room temperature was 20 °C. After 1, 24, and 48 h of irradiation, an aliquot was taken and concentrated under a stream of nitrogen and subjected to HPLC, LC-MS, ESI-MS, and ESI-MS/MS analyses. For LC-MS and HPLC analyses, samples were prepared in MeCN/H_2_O (5:95). For ESI-MS and ESI-MS/MS analyses, samples were prepared in MeCN/H_2_O/HCOOH (50:50:0.1). For the irradiation experiment of **BBSe**_**2**_, the above procedure was followed. A methanolic solution of **BBSe**_**2**_ (5 mM, 400 μL) was prepared. After 24 h of irradiation, the solvent was removed under a stream of nitrogen and subjected to GC-MS analysis (the sample was prepared in DCM for the analysis).

### Heating Experiments in the Presence of VA-044

A solution (equimolar, 5 mM) of peptide and **BBSe**_**2**_ in the presence of VA-044 (0.5 mM) in MeOH (400 μL) was placed in an HPLC vial. The HPLC vial caps were wrapped in parafilm, and the vials were wrapped in aluminum foil to protect them from light. The vials were placed in a heating block of the Thermo shaker LLG-uniTHERMIX 1 pro with shaking and heating functions. The heating block was 15 mL. The speed was set to 450 rpm, and the temperature was set to 45 °C. After 2 and 24 h, an aliquot was taken and concentrated under a stream of nitrogen and subjected to HPLC, ESI-MS, and ESI-MS/MS analyses. For HPLC analysis, samples were prepared in MeCN/H_2_O (5:95). For ESI-MS and ESI-MS/MS analyses, samples were prepared in MeCN/H_2_O/HCOOH (50:50:0.1).

### Heating Experiments in the Absence of VA-044

The same experimental conditions were followed as for the heating experiments in the presence of VA-044, except that VA-044 was not added to the solution.

## Data Availability

The data underlying this study are available in the published article and its Supporting Information.

## Supporting Information Available

NMR and MS spectra, chromatograms, DFT results (PDF)
