# Supplementary material for: Photochemical Transformations of Peptides Containing the N-(2-Selenoethyl)glycine Moiety
Source: ACS Omega. 2024 Mar 29;9(14):16775–91. doi: 10.1021/acsomega.4c01015 (PMC11007844; doi:10.1021/acsomega.4c01015)
Supplement: Supplementary file 1 — ao4c01015_si_001.pdf [file ao4c01015_si_001.pdf]

# Photochemical Transformations of Peptides Containing the *N*-(2-Selenoethyl)glycine Moiety

Özge Pehlivan,* Kamil Wojtkowiak, Aneta Jezierska, Mateusz Waliczek, and Piotr Stefanowicz

Faculty of Chemistry, University of Wrocław, F. Joliot-Curie str. 14, 50-383 Wrocław, Poland

## Table of Contents

1. NMR spectra ........ 2
2. ESI-MS, ESI-MS/MS, LC-MS, MALDI-MS, GC-MS and HPLC analyses ........ 8
   2.1. Linear(Se−Se)1 ........ 10
   2.2. Linear(Se−Se)2 ........ 18
   2.4. Linear(Se−Se)3 ........ 28
   2.5. Cyclo(Se−Se)1 ........ 35
   2.6. Cyclo(Se−Se)2 ........ 53
3. Theoretical analyses ........ 65

# 1. NMR spectra

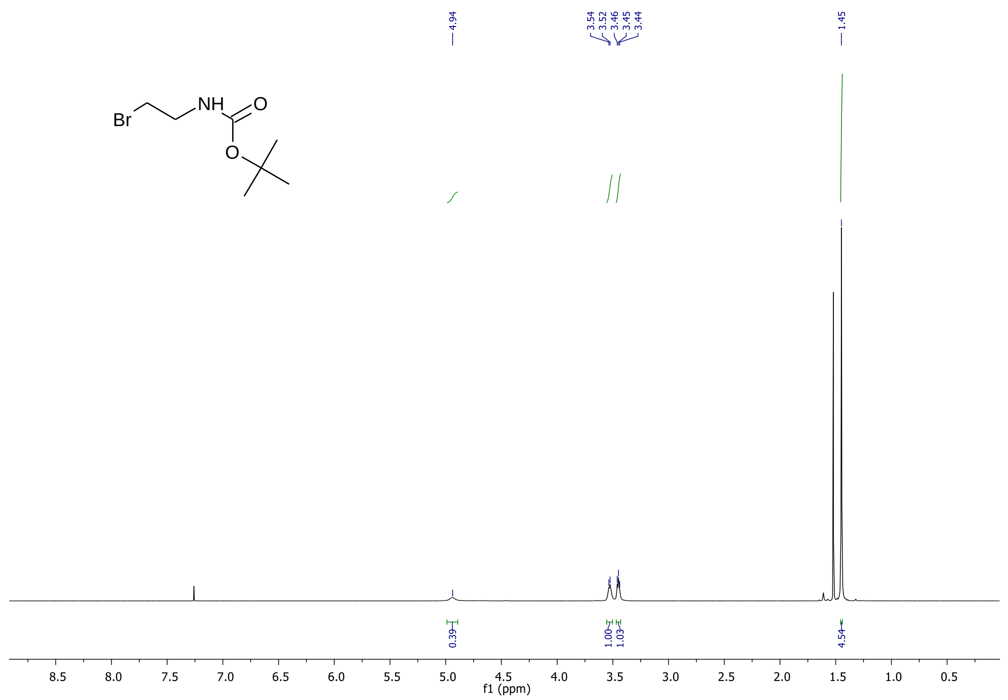


*Figure S1. $^{1}$H NMR ($CDCl_3$, 500 MHz, 300 K) spectrum of 2-((tert-butoxycarbonyl)amino)ethyl bromide*

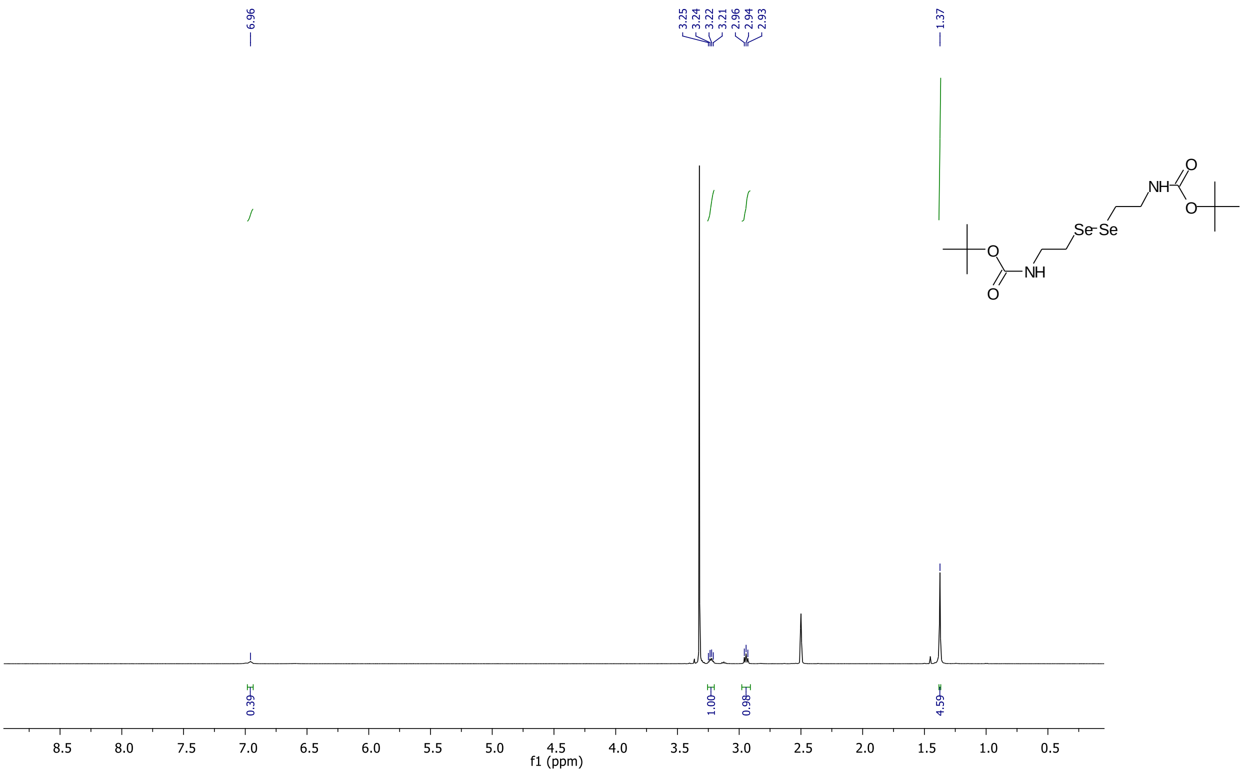


*Figure S2. $^{1}$H NMR (DMSO-$d_6$, 500 MHz, 300 K) spectrum of di-tert-butyl (diselanediylbis(ethane-2, 1-diyl))dicarbamate*

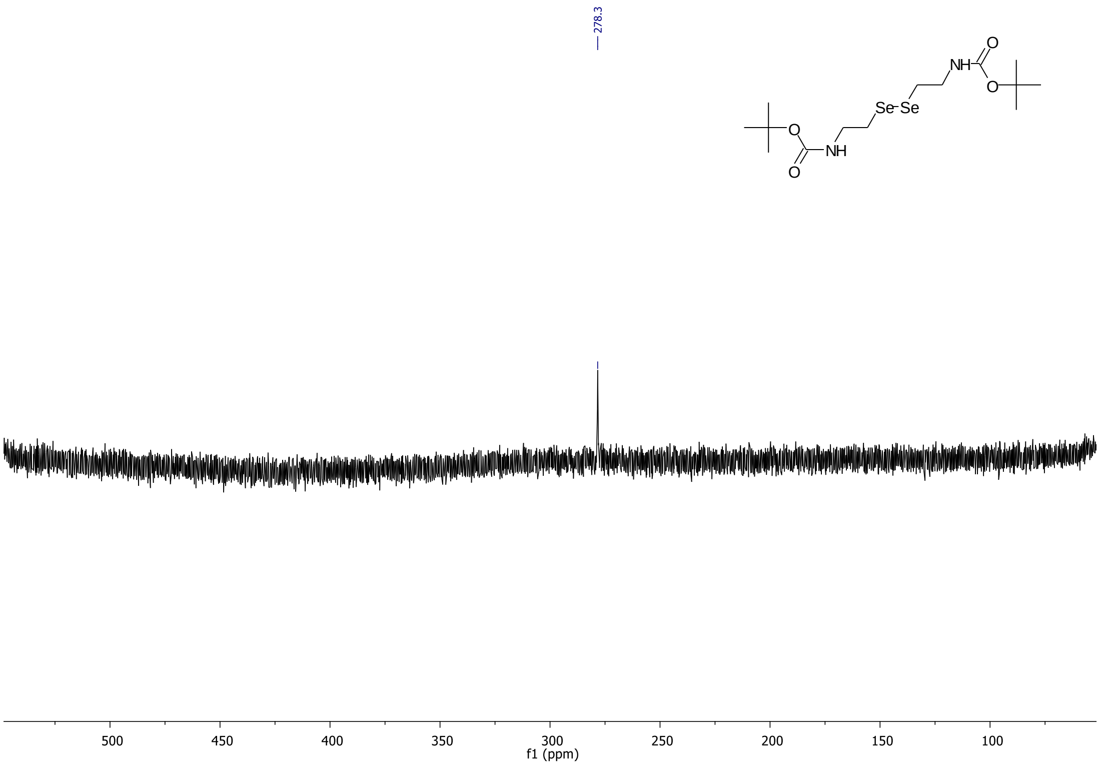


*Figure S3. $^{77}$Se NMR (CDCl$_3$, 114 MHz, 300 K) spectrum of di-tert-butyl (diselanediylbis(ethane-2, 1-diyl))dicarbamate*

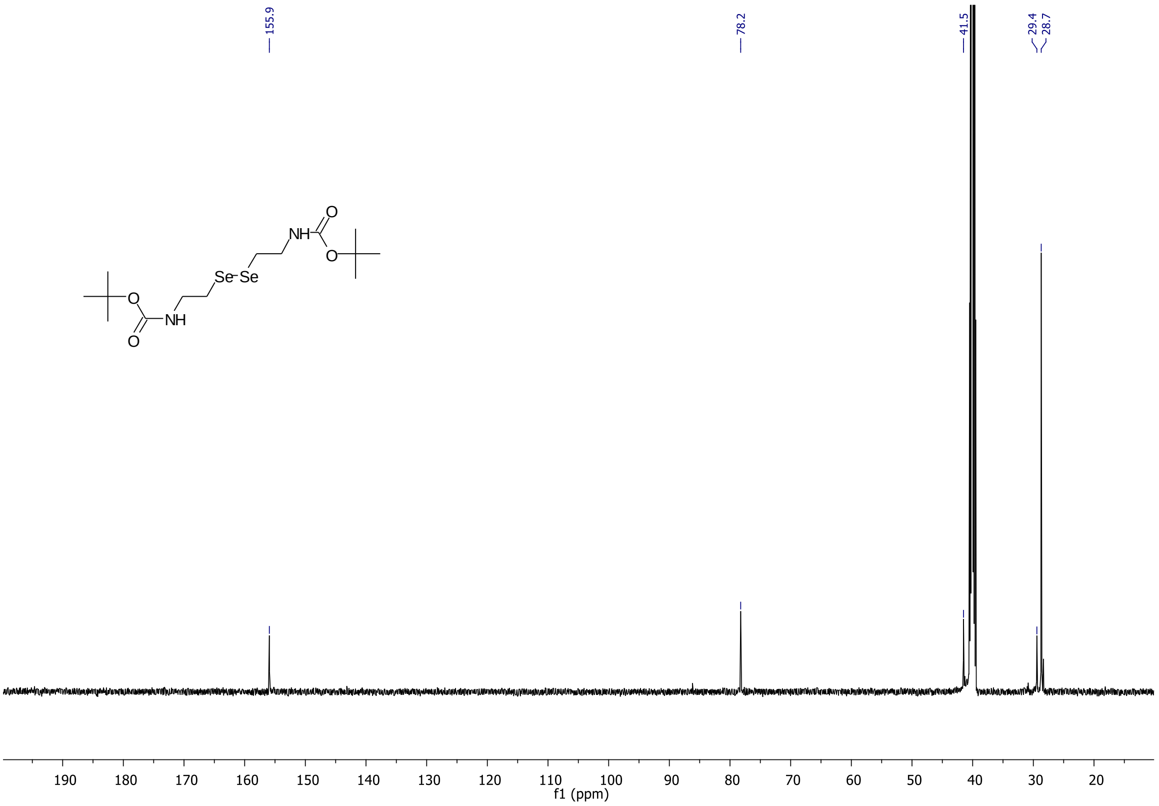


*Figure S4. $^{13}$C{$^1$H} NMR (DMSO-$d_6$, 126 MHz, 300 K) spectrum of di-tert-butyl (diselanediylbis(ethane-2, 1-diyl))dicarbamate*

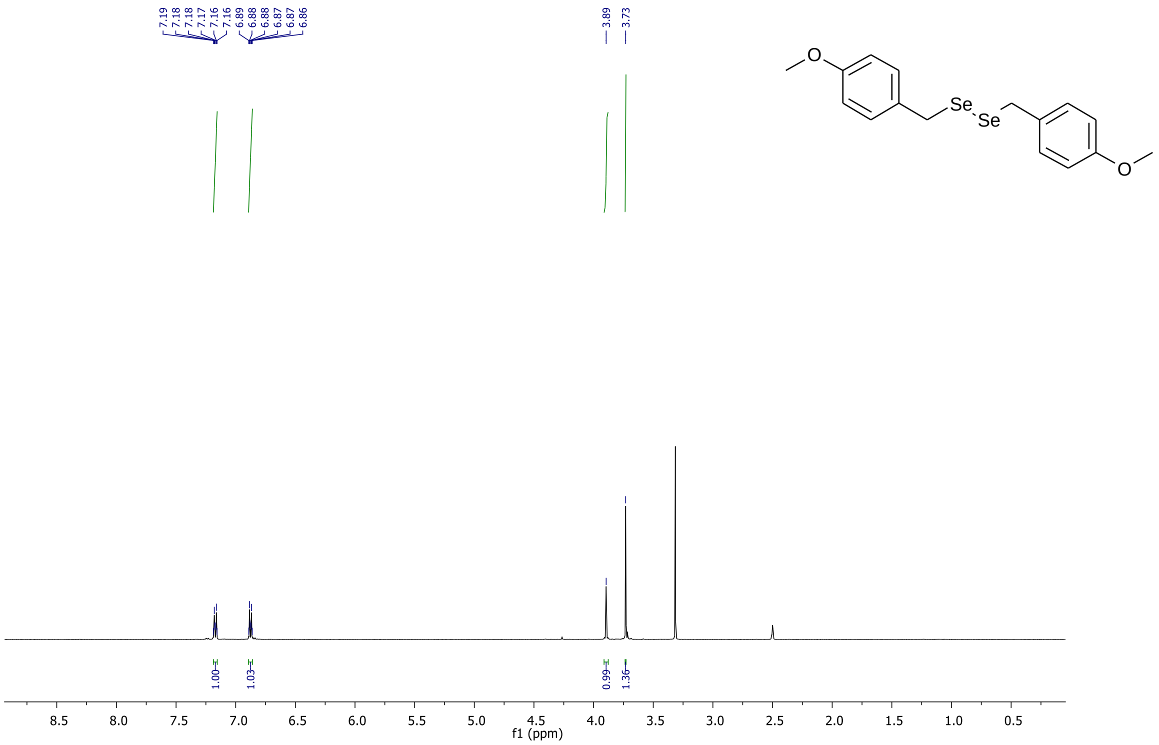


*Figure S5. $^{1}$H NMR (DMSO-d$_6$, 500 MHz, 300 K) spectrum of 1,2-bis(4-methoxybenzyl)diselane*

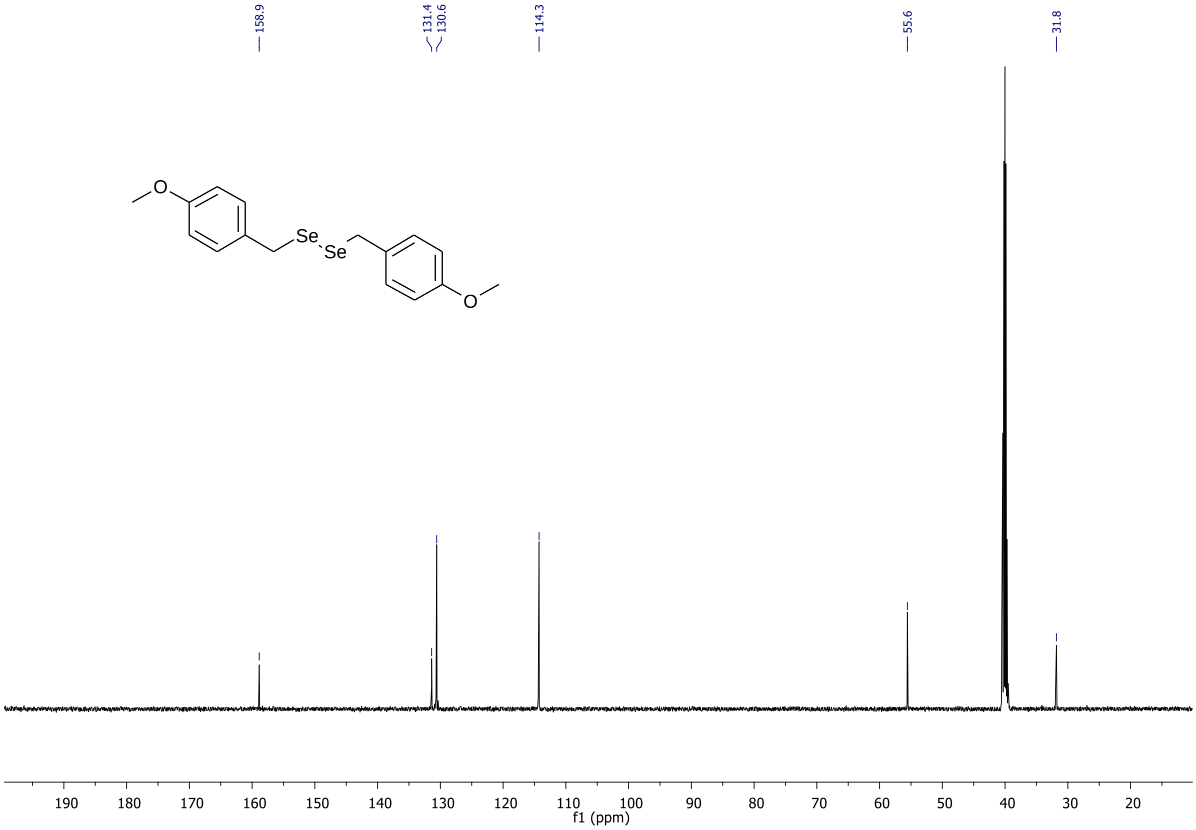


*Figure S6. $^{13}$C{$^{1}$H} NMR (DMSO-d$_6$, 126 MHz, 300 K) spectrum of 1,2-bis(4-methoxybenzyl)diselane*

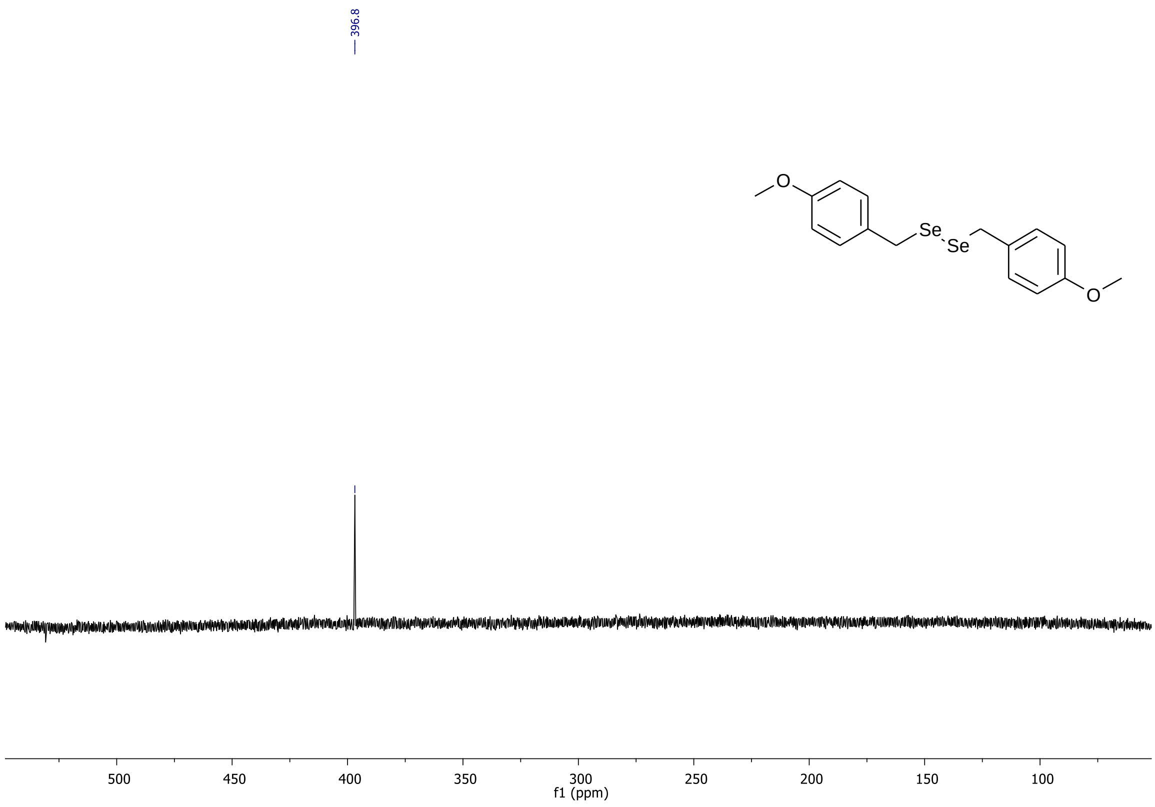

*Figure S7. $^{77}$Se NMR ($CDCl_3$, 114 MHz, 300 K) spectrum of 1,2-bis(4-methoxybenzyl)diselane*

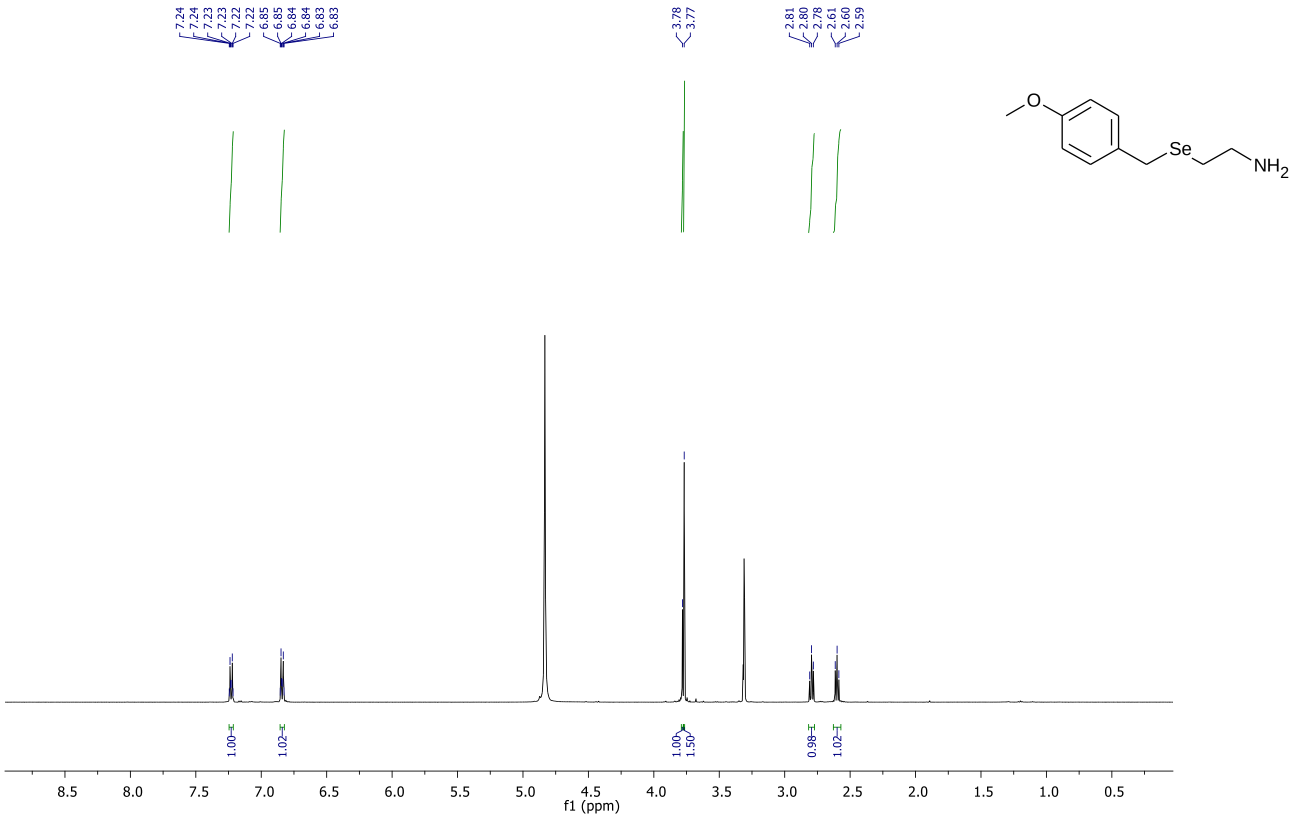

*Figure S8. $^{1}$H NMR (MeOD, 500 MHz, 300 K) spectrum of 2-(4-methoxybenzylseleno)ethylamine*

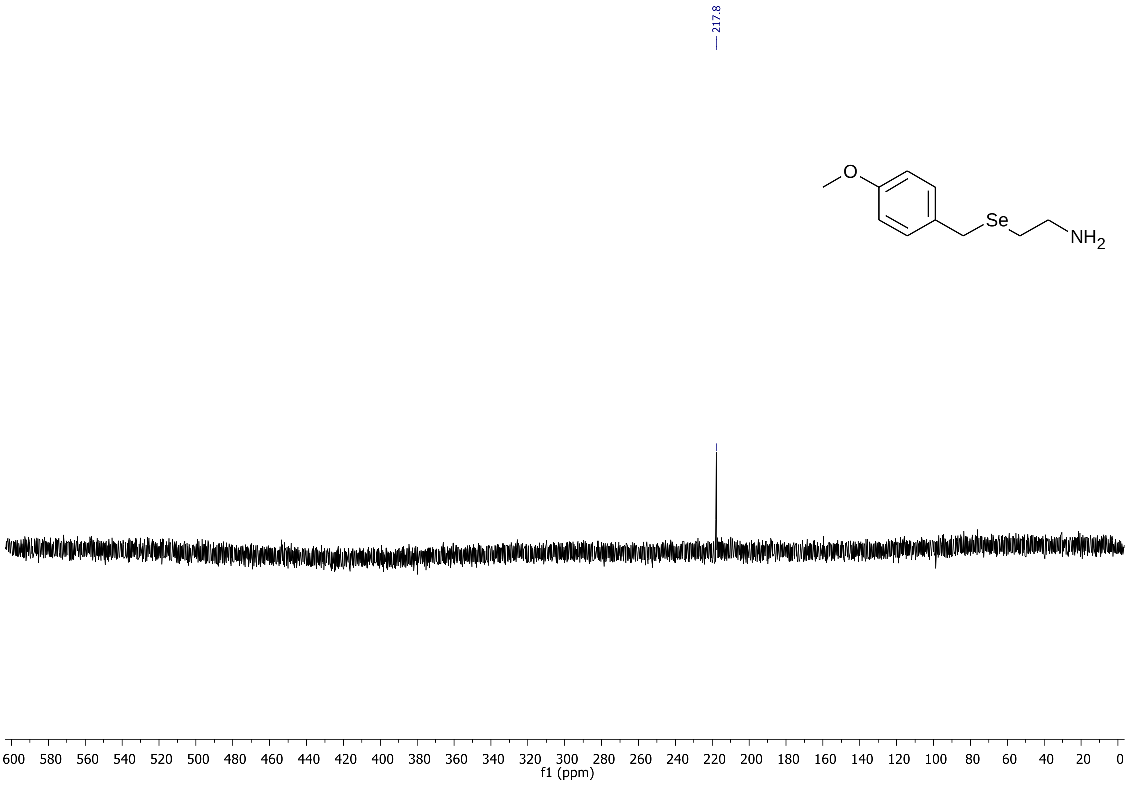


*Figure S9.* $^{77}$*Se NMR (MeOD, 114 MHz, 300 K) spectrum of 2-(4-methoxybenzylseleno)ethylamine*

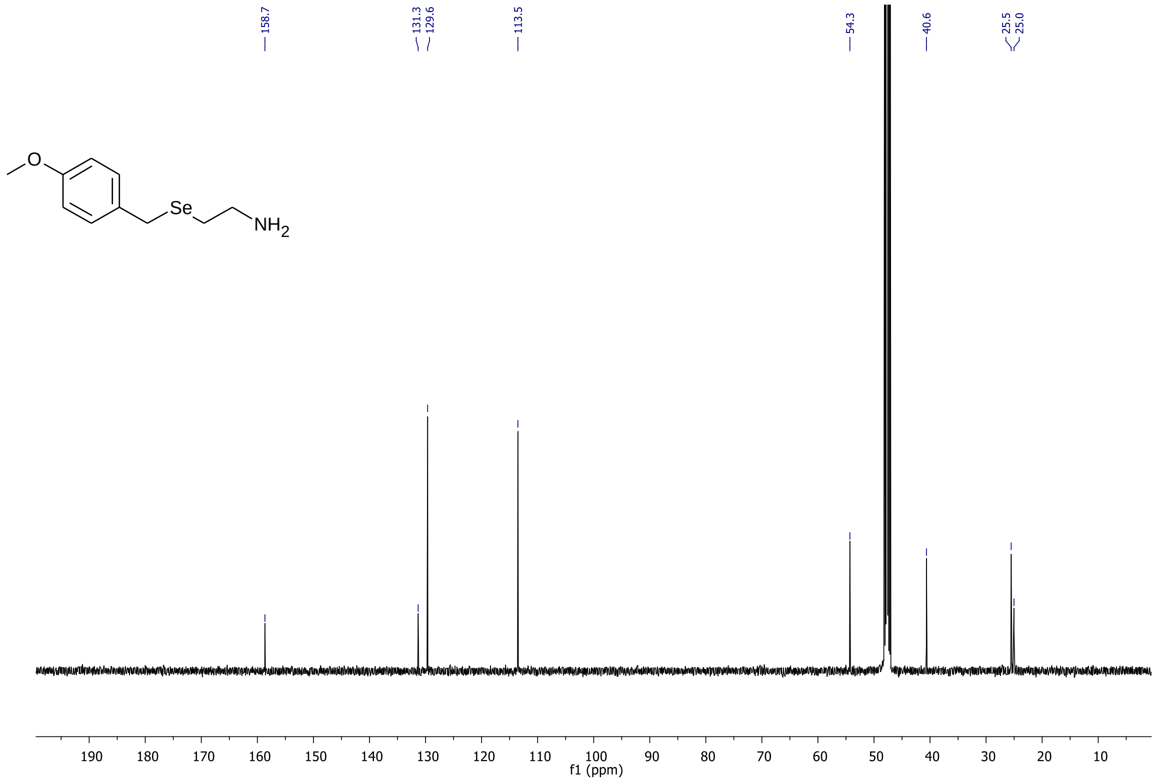


*Figure S10.* $^{13}C\{^{1}H\}$ *NMR (MeOD, 126 MHz, 300 K) spectrum of 2-(4-methoxybenzylseleno)ethylamine*

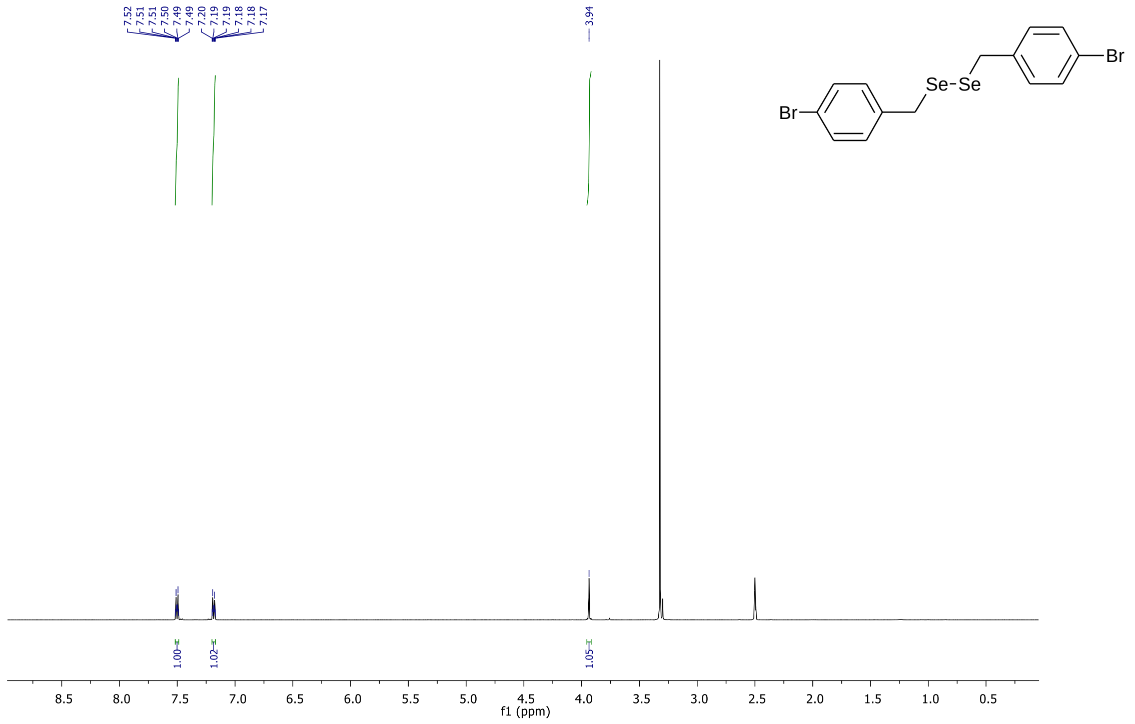


*Figure S11. $^{1}$H NMR (DMSO-$d_6$, 500 MHz, 300 K) spectrum of 1,2-bis(4-bromobenzyl)diselane*

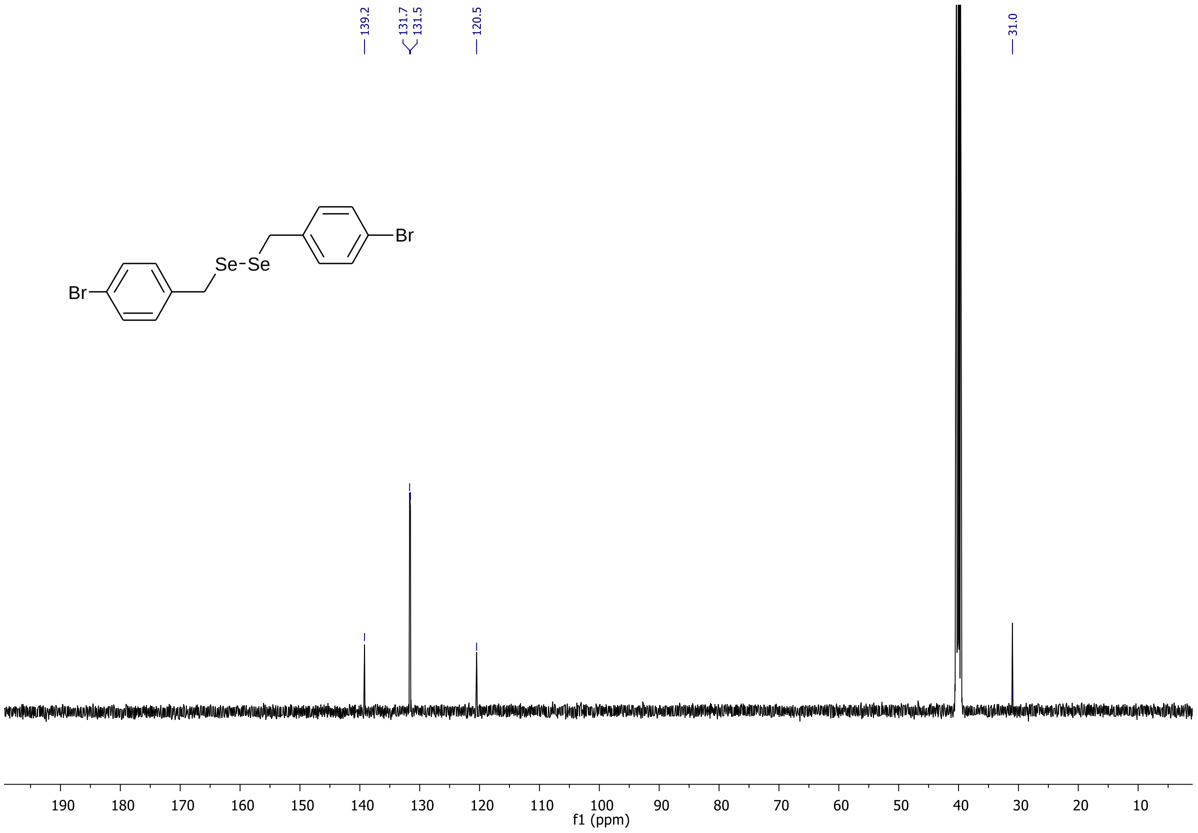


*Figure S12. $^{13}C\{^1H\}$ NMR (DMSO-$d_6$, 126 MHz, 300 K) spectrum of 1,2-bis(4-bromobenzyl)diselane*

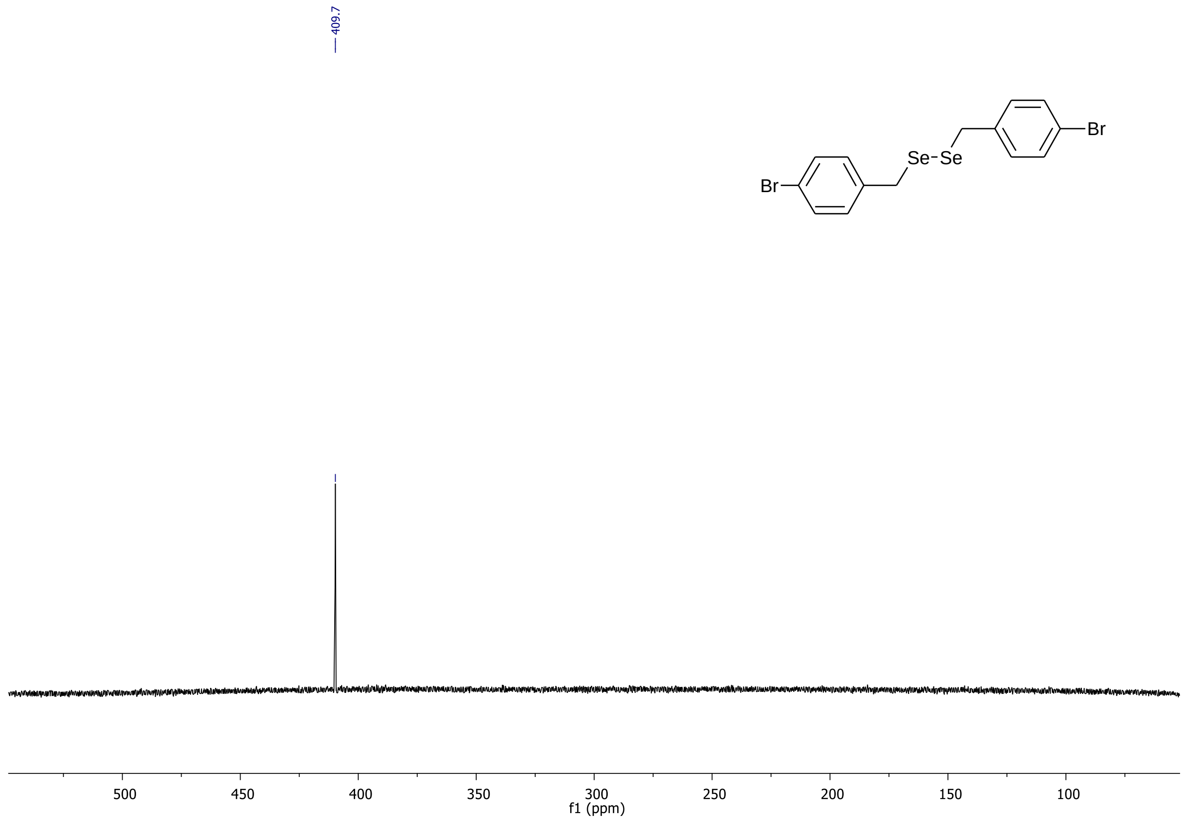


*Figure S13.* $^{77}$*Se NMR (CDCl$_3$, 114 MHz, 300 K) spectrum of 1,2-bis(4-bromobenzyl)diselane*

## 2. ESI-MS, ESI-MS/MS, LC-MS, MALDI-MS, GC-MS and HPLC analyses

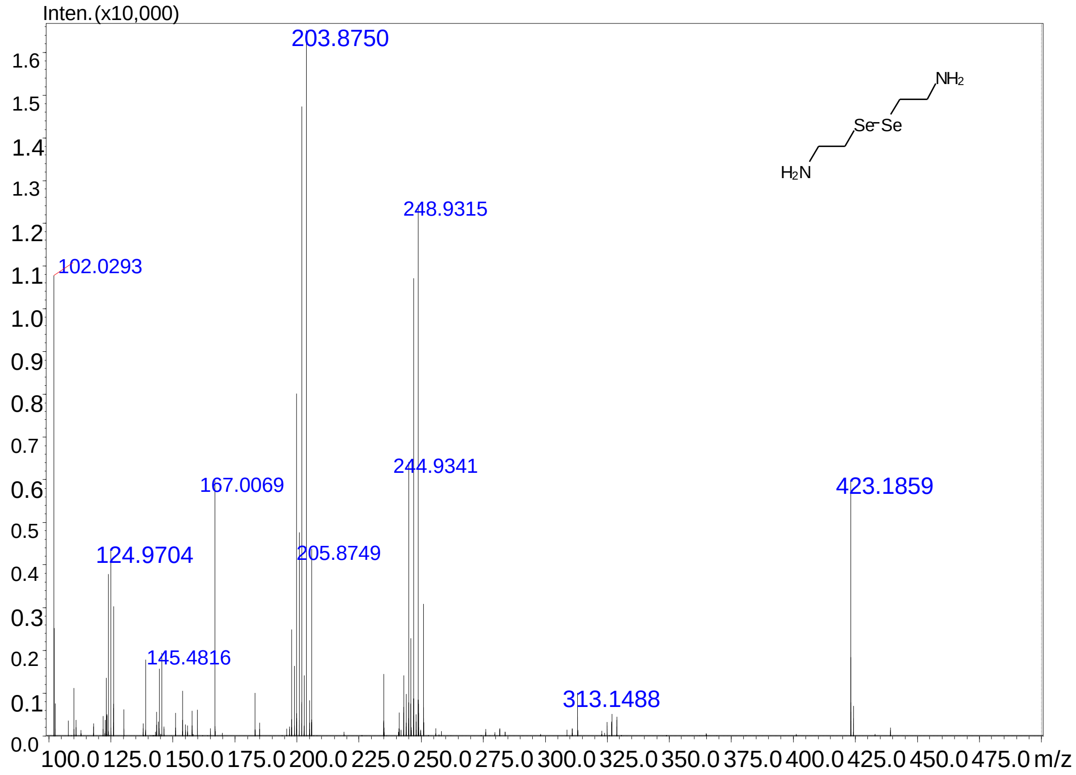


*Figure S14. ESI-qTOF-MS (Shimadzu) spectrum of selenocystamine ($C_4H_{12}N_2Se_2$)*

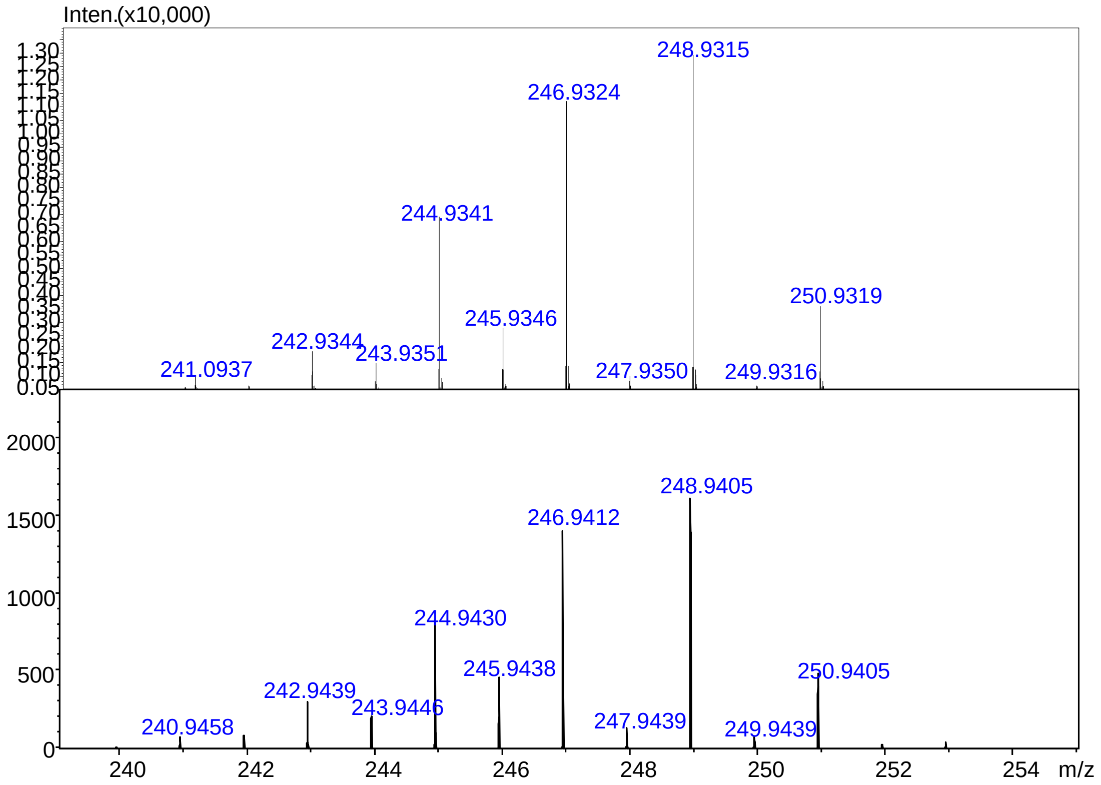


*Figure S15. Isotopic distribution of the relative peak, m/z found $[M+H]^+$: 248.9315 (top) and the simulated peak, m/z calculated $[M+H]^+$: 248.9405 (bottom)*

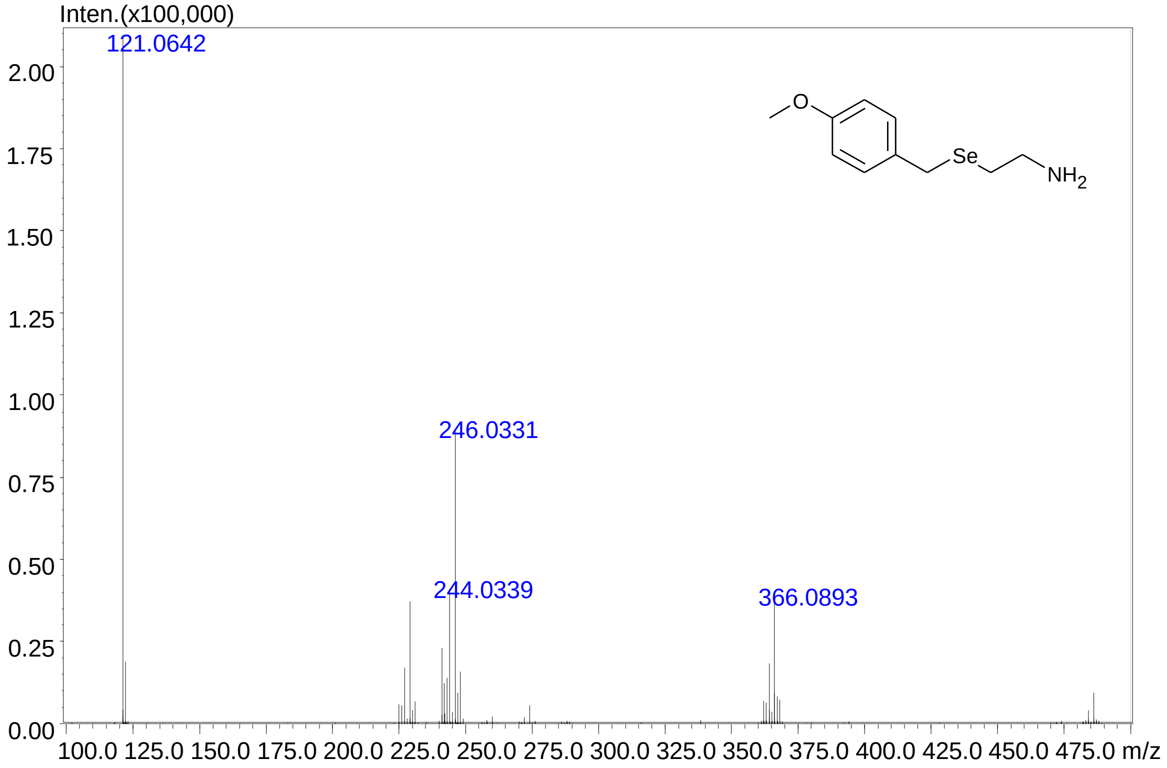


*Figure S16. ESI-qTOF-MS (Shimadzu) spectrum of 2-(4-methoxybenzylseleno)ethylamine ($C_{10}H_{15}NOSe$)*

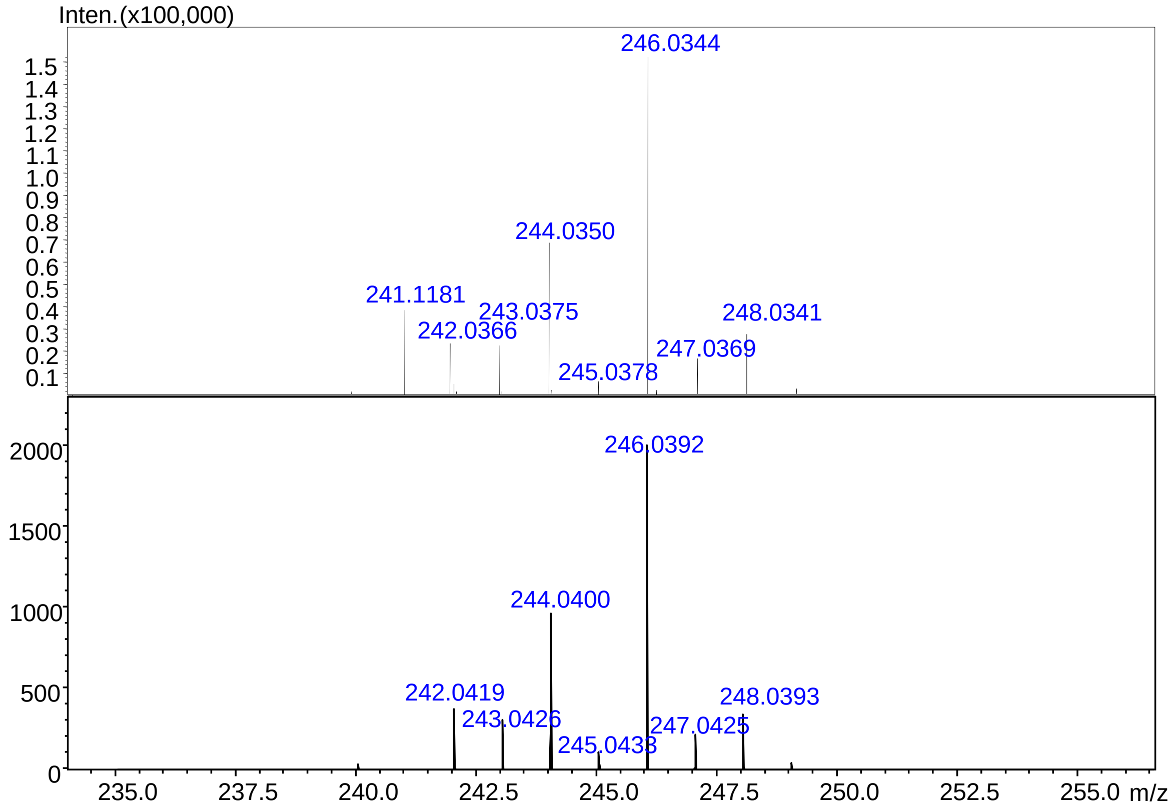


*Figure S17. Isotopic distribution of the relative peak, m/z found $[M+H]^+$: 246.0344 (top) and the simulated peak, m/z calculated $[M+H]^+$: 246.0392 (bottom)*

## 2.1. Linear(Se−Se)1

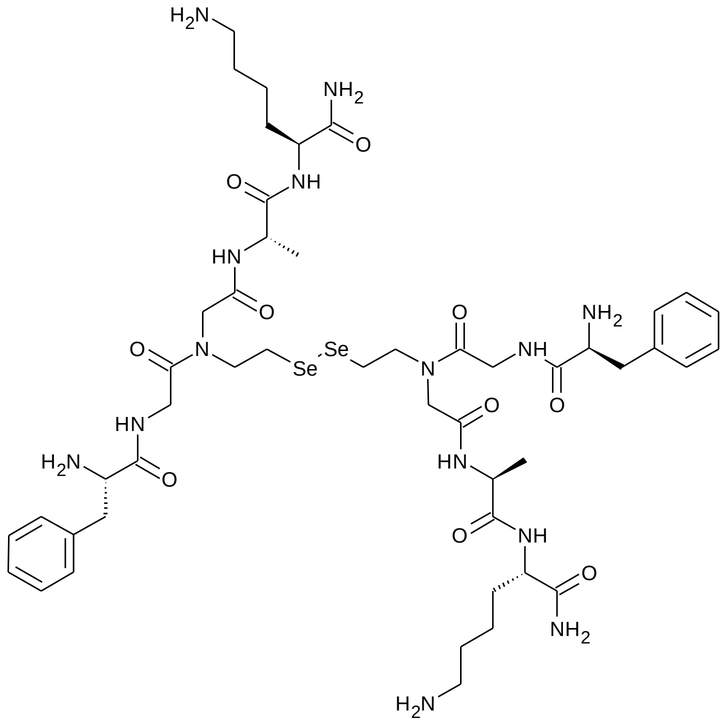

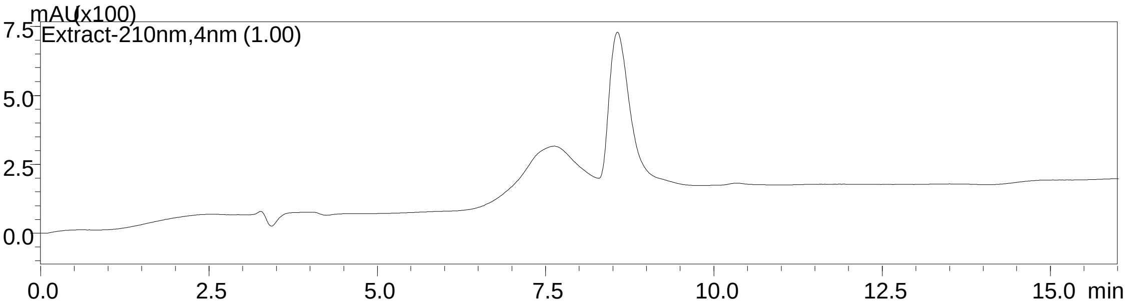


*Figure S18. HPLC chromatogram of **Linear(Se−Se)1** (retention time: 8.56 min)*

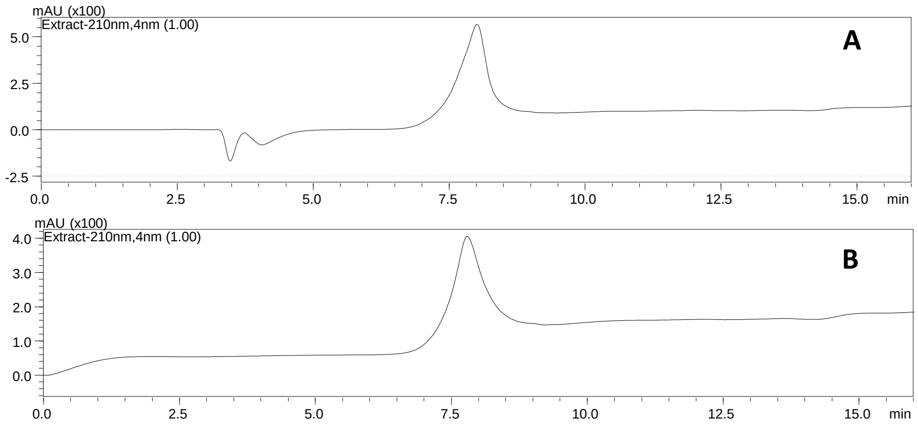


*Figure S19. HPLC chromatograms showing a broad signal when HPLC-grade solvents (A) and no-column injection (B) were recorded.*

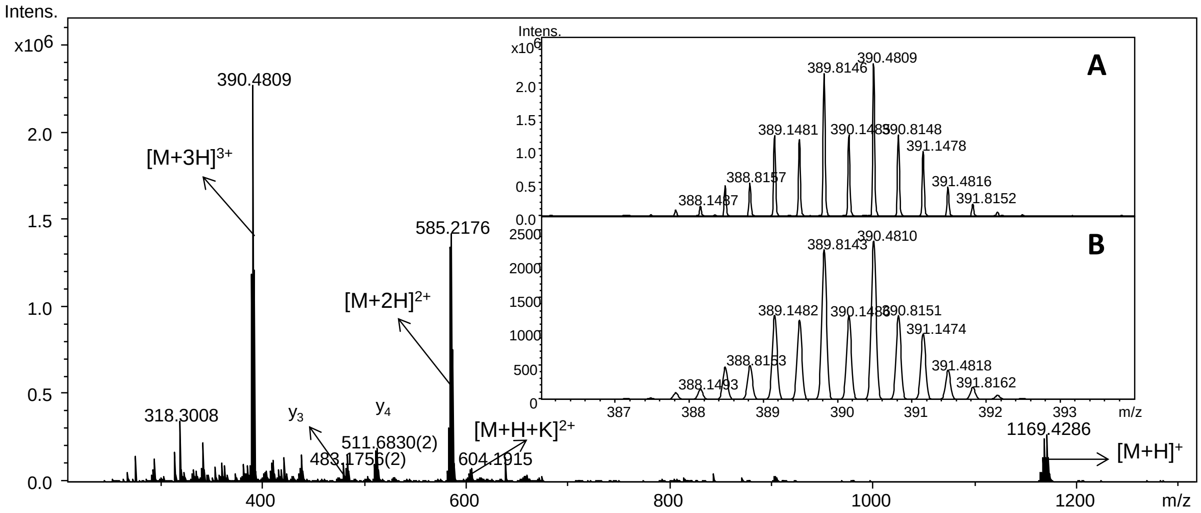


*Figure S20. ESI-qTOF-MS spectrum of **Linear(Se−Se)1** ($C_{48}H_{76}N_{14}O_{10}Se_2$) (A) Isotopic distribution of the relative peak, m/z found $[M+3H]^{3+}$: 390.4809 (B) Isotopic distribution of the simulated peak, m/z calculated $[M+3H]^{3+}$: 390.4810 (Fragmentation was observed in MS spectrum. $y_3$ and $y_4$ correspond to the fragments.*

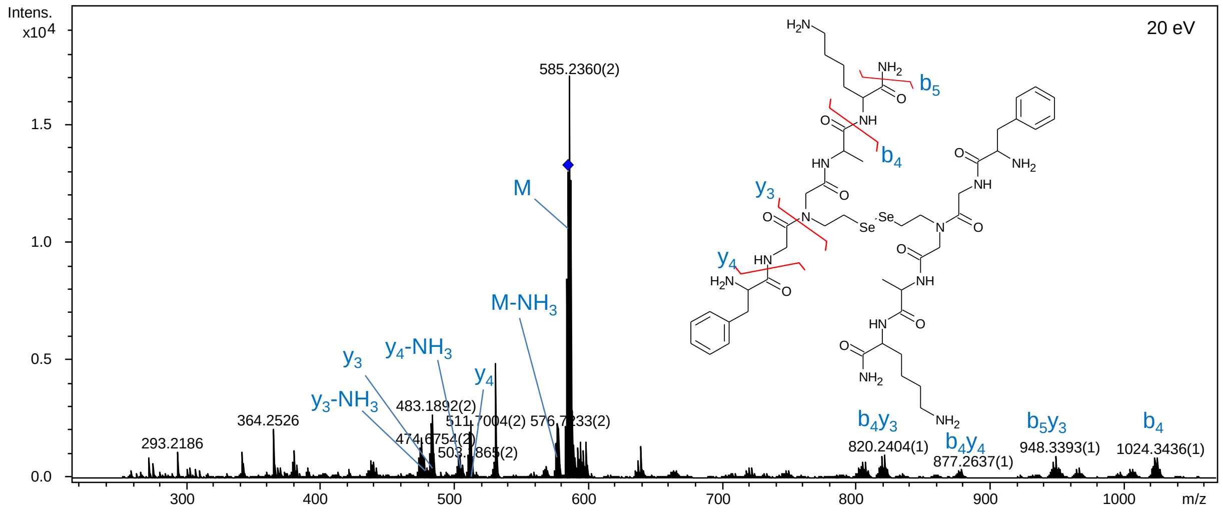


*Figure S21. ESI-qTOF-MS/MS (CE 20 eV) spectrum of **Linear(Se−Se)1** ($C_{48}H_{76}N_{14}O_{10}Se_2$). Precursor ion: m/z $[M+2H]^{2+}$: 585.2360 (calculated for M: 585.2179, z=2+)*

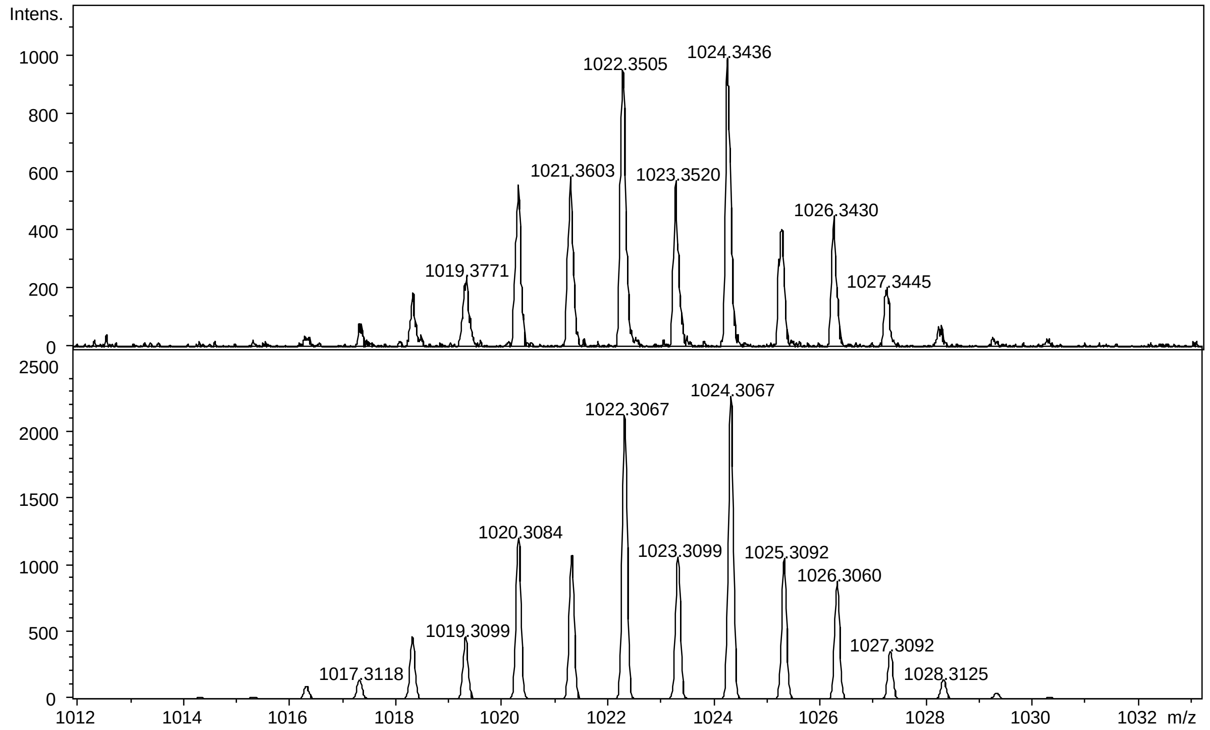


*Figure S22. ESI-qTOF-MS/MS (CE 20 eV) spectrum of **Linear(Se−Se)1** ($C_{48}H_{76}N_{14}O_{10}Se_2$). Isotopic distribution of the relative peak that corresponds to the fragment $b_4$, m/z found $[M+H]^+$: 1024.3436 (top) and isotopic distribution of the simulated peak, m/z calculated $[M+H]^+$: 1024.3067 (bottom)*

## 2.1.1. Metathesis reaction between Linear(Se−Se)1 and $BBSe_2$ under visible light

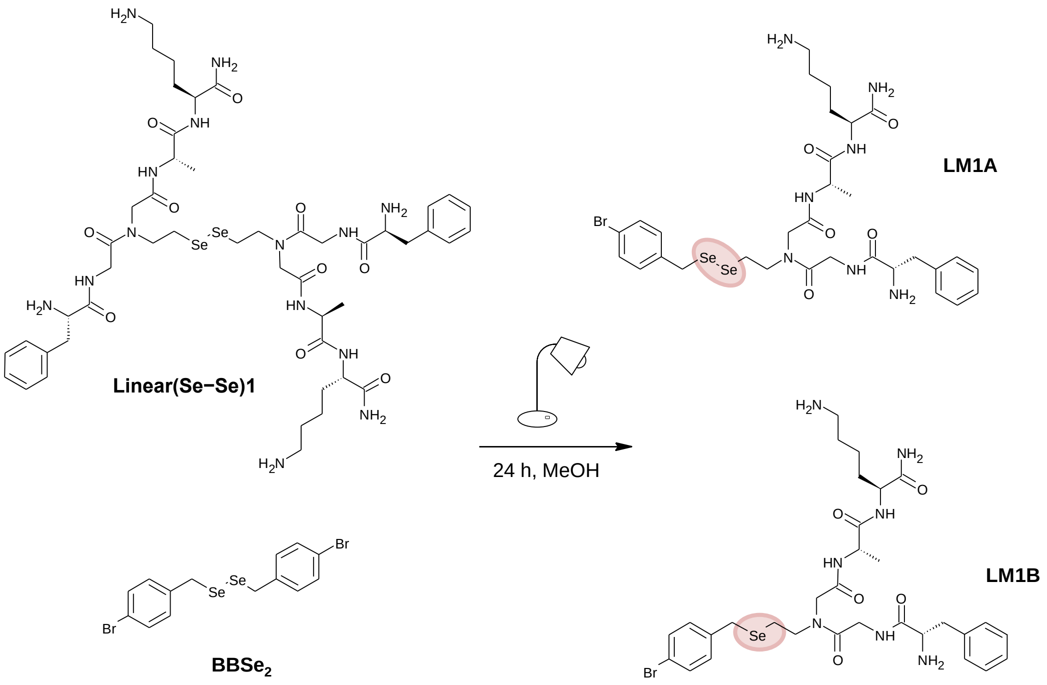


### *2.1.1.1. LM1A*

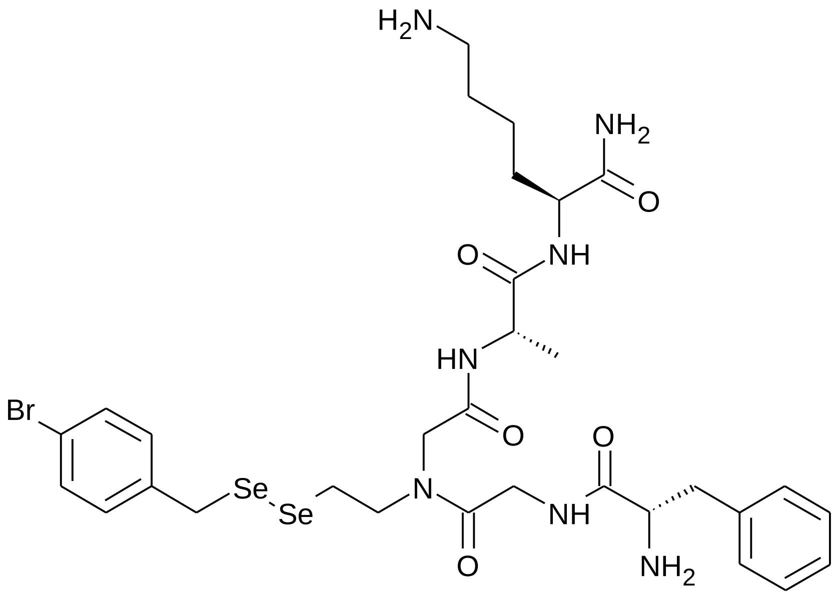

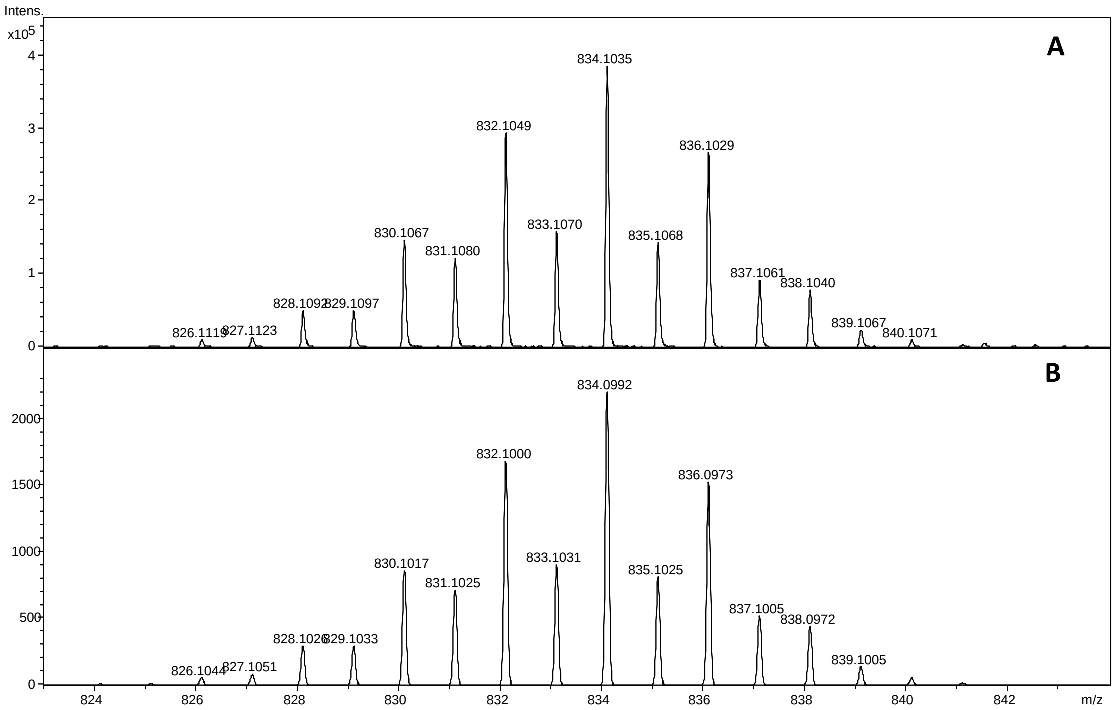


*Figure S23. ESI-qTOF-MS spectrum of* ***LM1A*** *($C_{31}H_{44}BrN_7O_5Se_2$) (A) Isotopic distribution of the relative peak, m/z found $[M+H]^+$: 834.1035 (B) Isotopic distribution of the simulated peak, m/z calculated $[M+H]^+$: 834.0992*

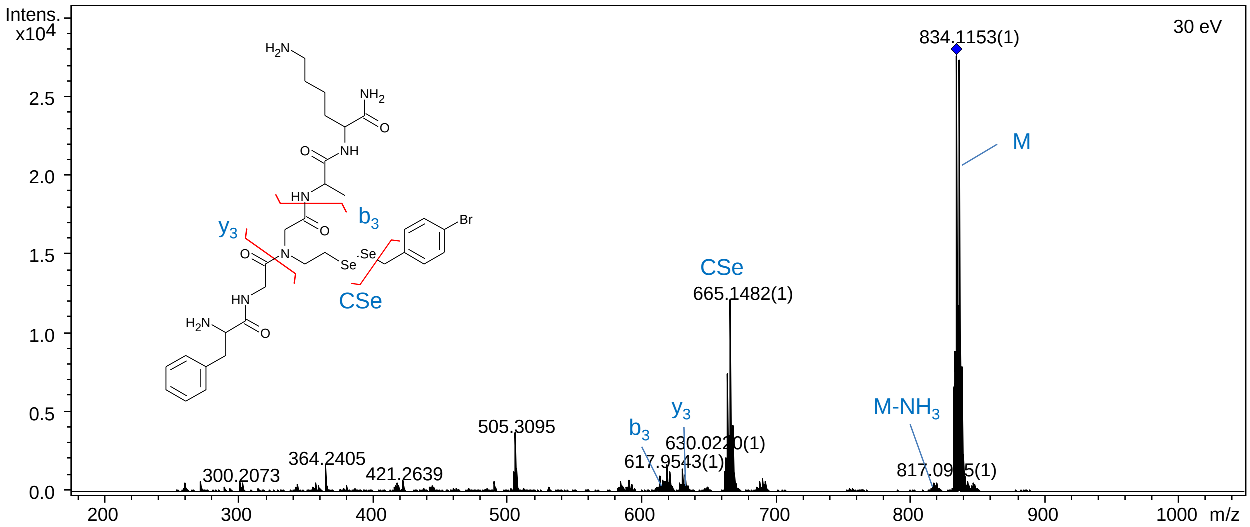


*Figure S24. ESI-qTOF-MS/MS (CE 30 eV) spectrum of* ***LM1A*** *($C_{31}H_{44}BrN_7O_5Se_2$). Precursor ion: m/z $[M+H]^+$: 834.1153 (calculated for M: 834.0992, z=+) The notation "CSe" was used to indicate cleavage of the C−Se bond.*

## *2.1.1.2. LM1B*

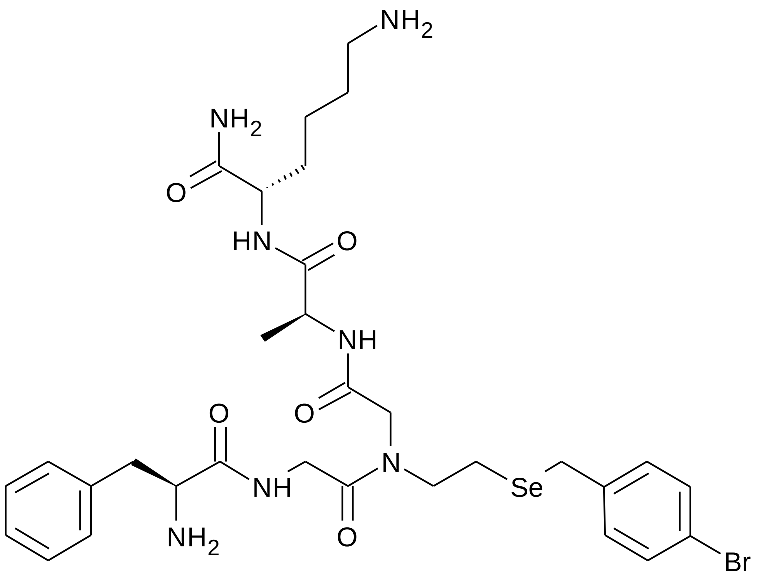


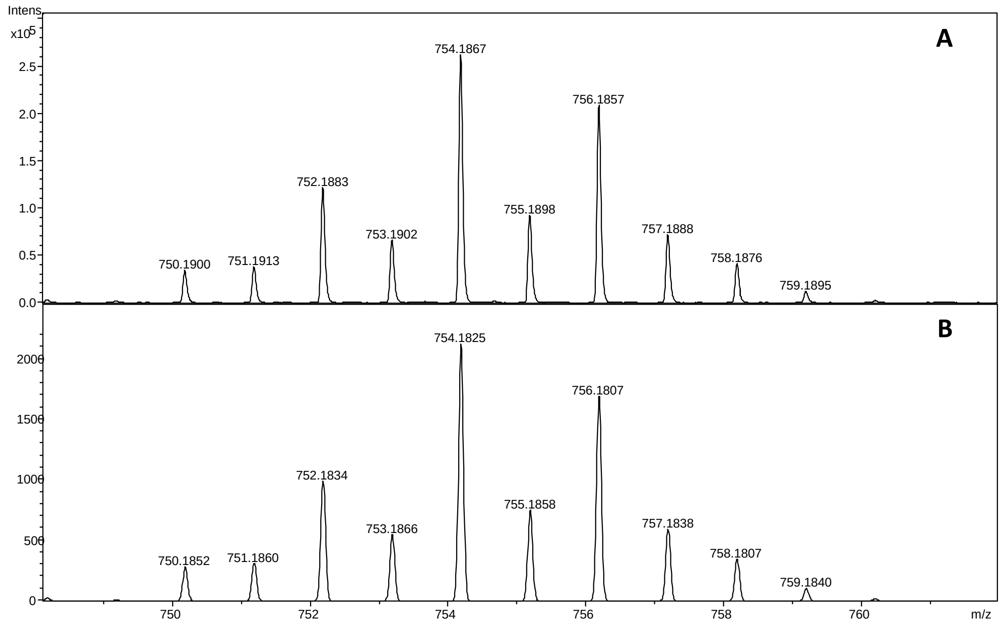


*Figure S25. ESI-qTOF-MS spectrum of **LM1B** ($C_{31}H_{44}BrN_7O_5Se$) (A) Isotopic distribution of the relative peak, m/z found $[M+H]^+$: 754.1867 (B) Isotopic distribution of the simulated peak, m/z calculated $[M+H]^+$: 754.1825*

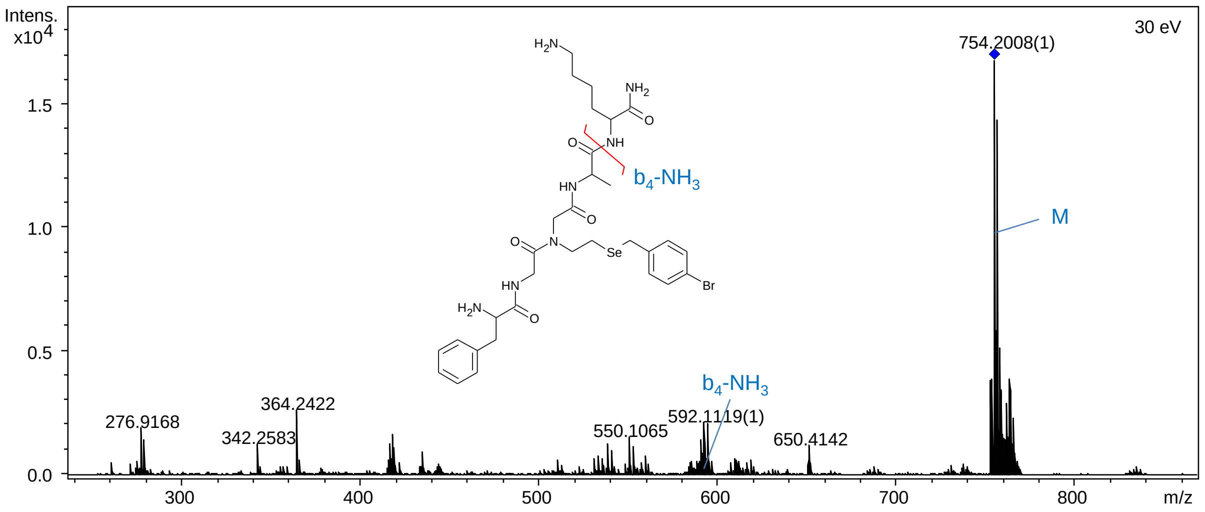


*Figure S26. ESI-qTOF-MS/MS (CE 30 eV) spectrum of **LM1B** ($C_{31}H_{44}BrN_7O_5Se$). Precursor ion: m/z $[M+H]^+$: 754.2008 (calculated for M: 754.1825, z=+)*

## 2.1.2. Metathesis reaction between Linear(Se−Se)1 and $BBSe_2$ under heat

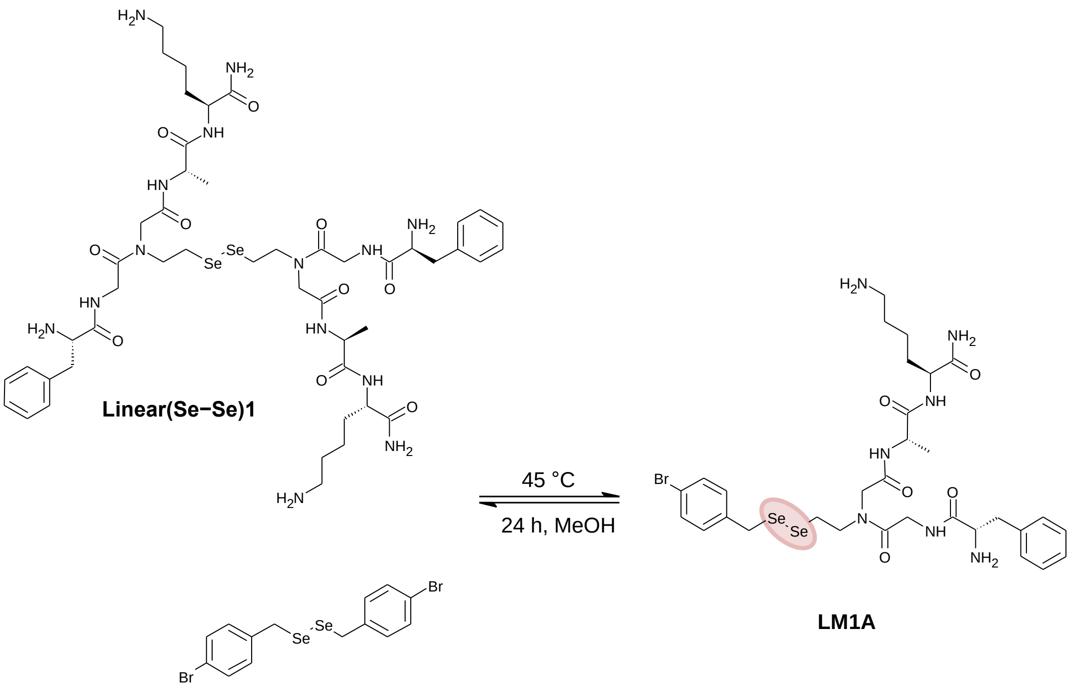

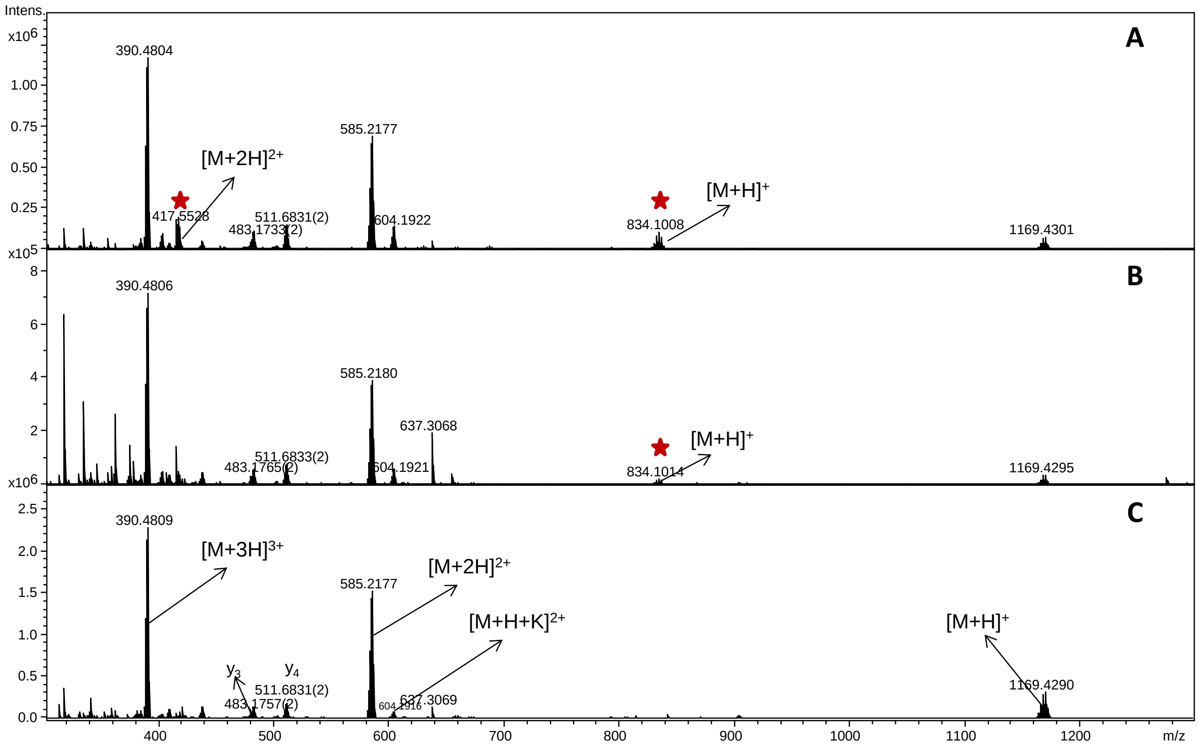


*Figure S27. ESI-qTOF-MS spectra illustrating the progress of the metathesis reaction between **Linear(Se−Se)1** and **BBSe₂**. Spectra (B) and (A) were acquired after 2 h and 24 h of incubation of the sample in the dark at 45 °C, respectively. The spectrum (C) corresponds to the purified **Linear(Se−Se)1**. Conditions: (5 mM) **Linear(Se−Se)1**, (5 mM) **BBSe₂**, methanol, dark and 45 °C. The red star indicates the peak of **LM1A**.*

## 2.1.3. Metathesis reaction between Linear(Se−Se)1 and BBSe$_2$ in the presence of VA-044 under heat

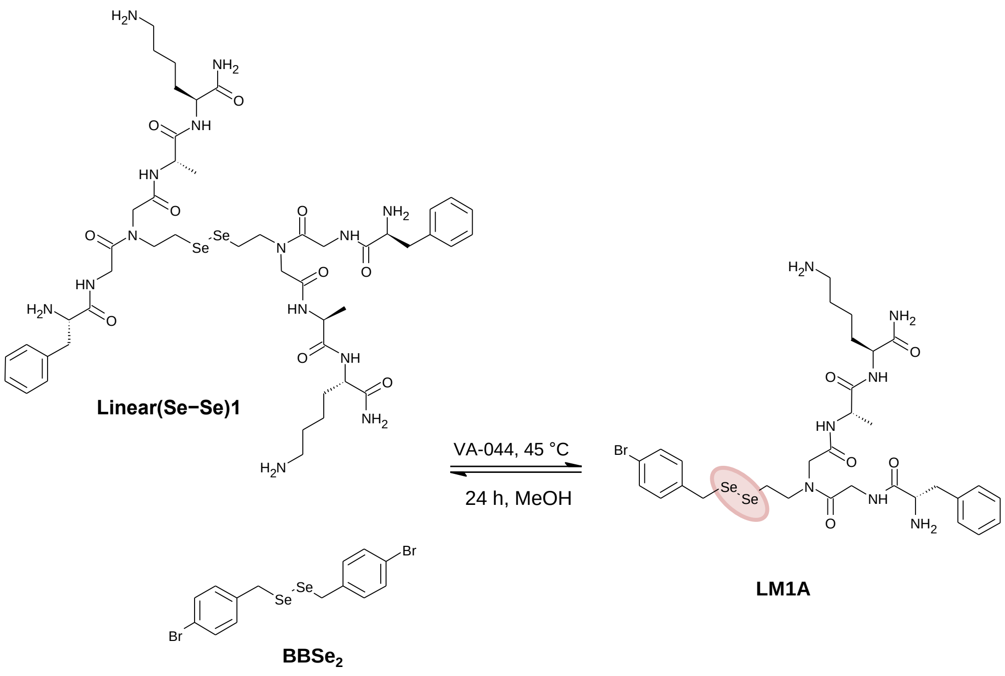

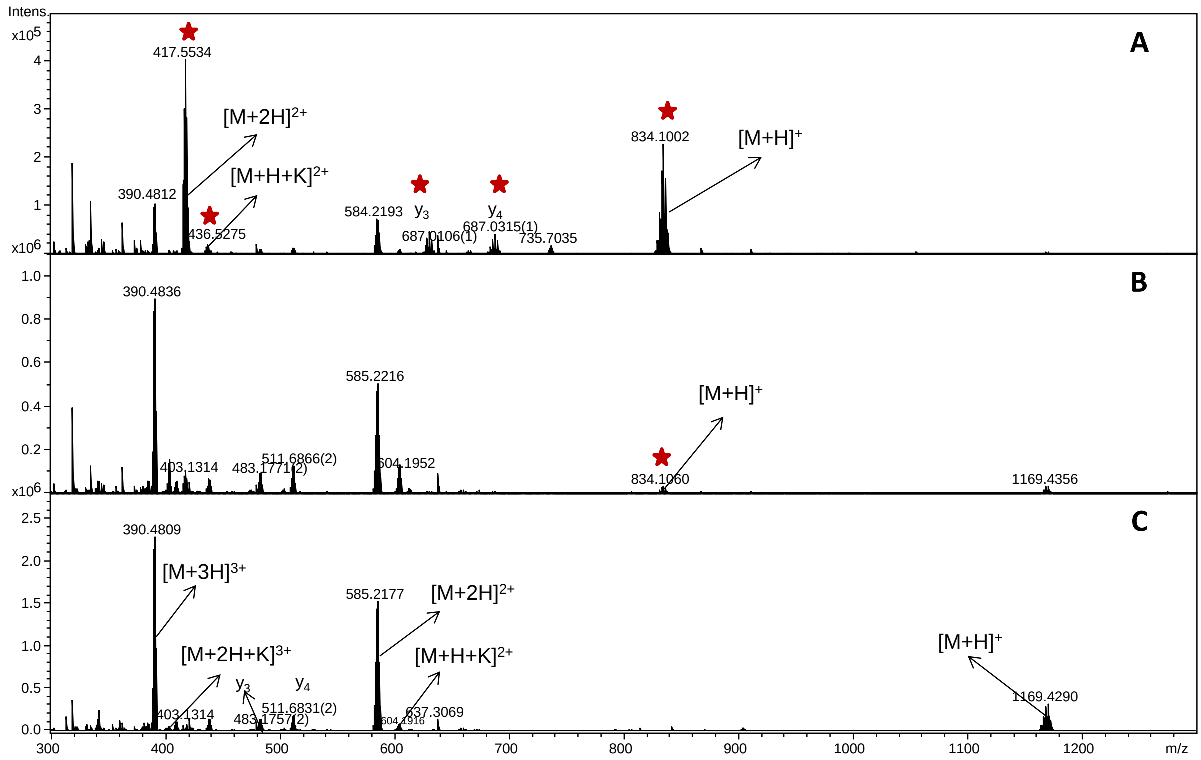


*Figure S28. ESI-qTOF-MS spectra illustrating the progress of the metathesis reaction between **Linear(Se−Se)1** and **BBSe$_2$**. Spectra (B) and (A) were acquired after 2 h and 24 h of incubation of the sample in the dark at 45 °C, respectively. The spectrum (C) corresponds to the purified **Linear(Se−Se)1**. Conditions: (5 mM) **Linear(Se−Se)1**, (5 mM) **BBSe$_2$**, (0.5 mM) VA-044, methanol, dark and 45 °C. The red star indicates the peak of **LM1A**. After incubation of the sample in the presence of VA-044 at 45 °C, fragmentation of **LM1A** was observed in the MS spectrum. $y_3$ and $y_4$ correspond to the fragments of **LM1A** (see spectrum A).*

## 2.2. Linear(Se−Se)2

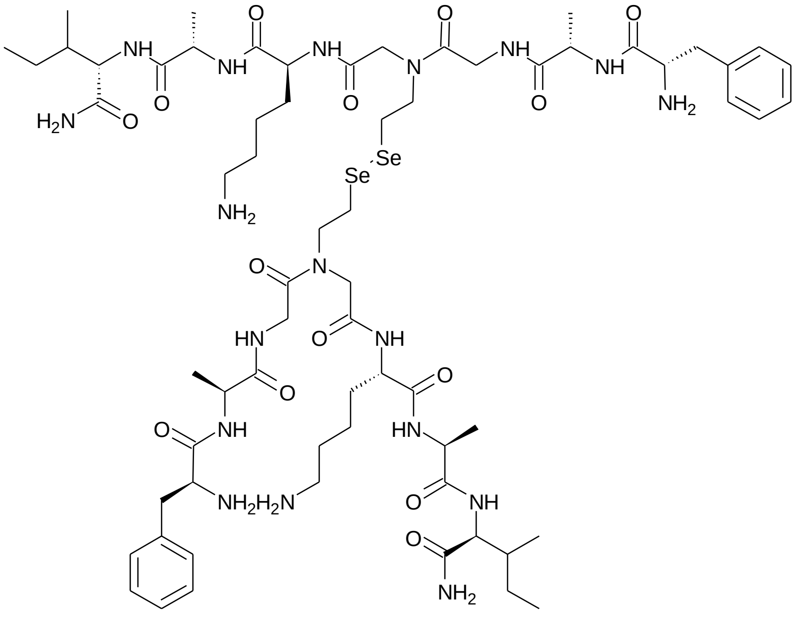

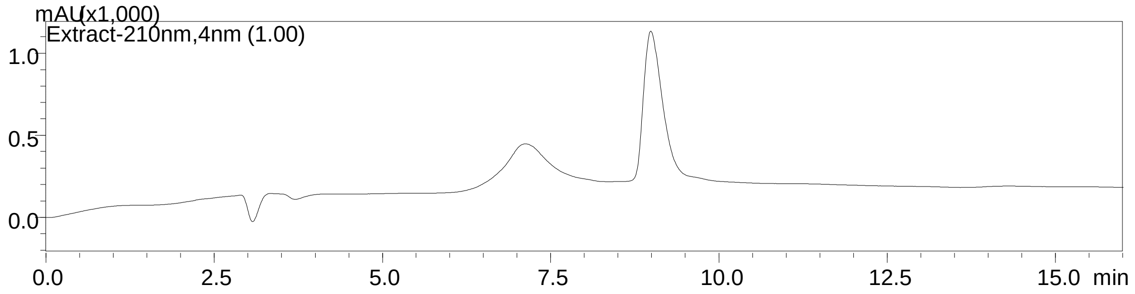


*Figure S29. HPLC chromatogram of **Linear(Se−Se)2** (retention time: 8.99 min)*

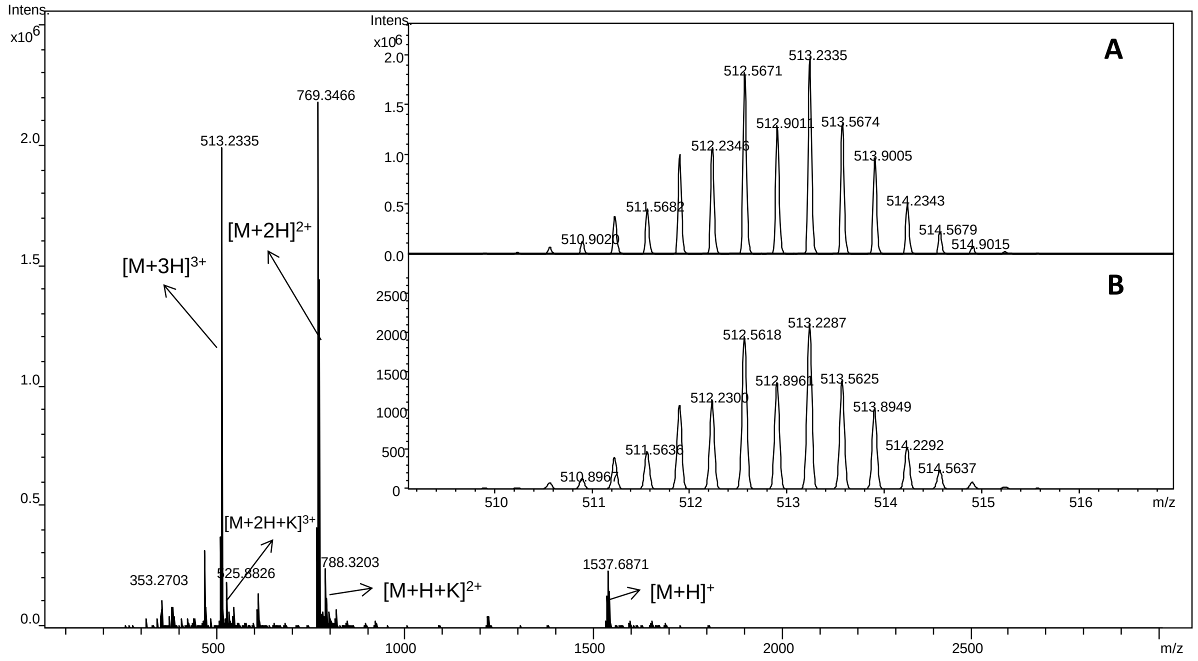


*Figure S30. ESI-qTOF-MS spectrum of **Linear(Se−Se)2** ($C_{66}H_{108}N_{18}O_{14}Se_2$) (A) Isotopic distribution of the relative peak, m/z found $[M+3H]^{3+}$: 513.2335 (B) Isotopic distribution of the simulated peak, m/z calculated $[M+3H]^{3+}$: 513.2287*

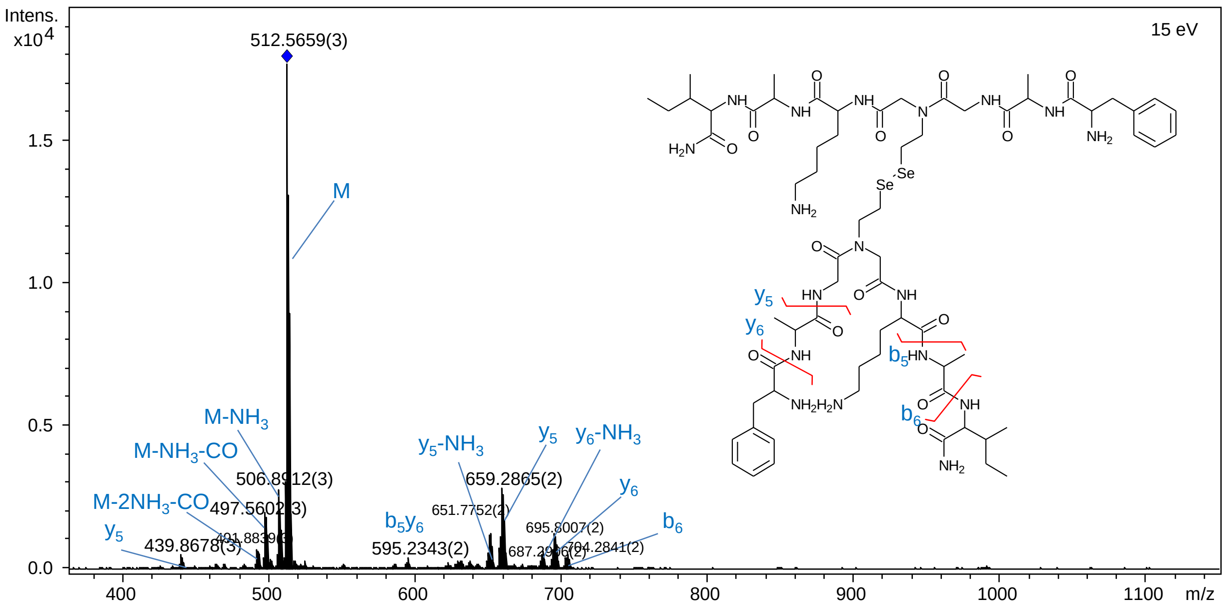


*Figure S31. ESI-qTOF-MS/MS (CE 15 eV) spectrum of* ***Linear(Se−Se)2*** *($C_{66}H_{108}N_{18}O_{14}Se_2$). Precursor ion: m/z $[M+3H]^{3+}$: 512.5659 (calculated for M: 512.5618, z=3+)*

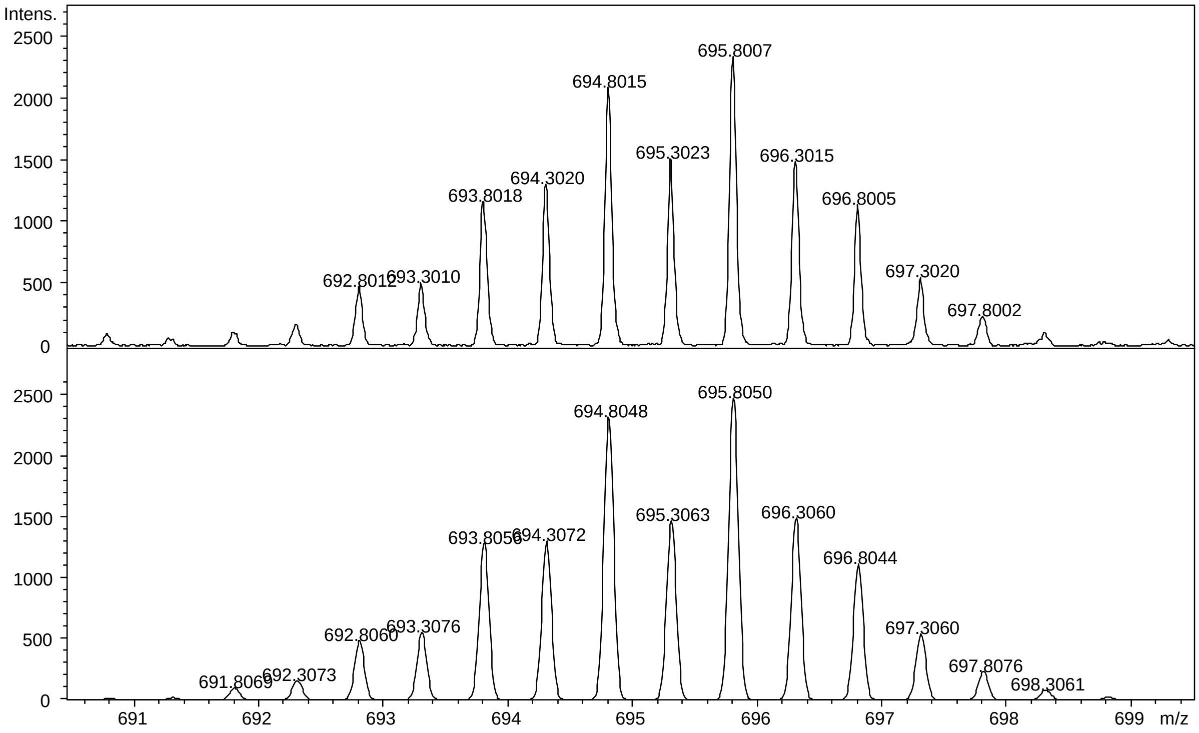


*Figure S32. ESI-qTOF-MS/MS (CE 15 eV) spectrum of* ***Linear(Se−Se)2*** *($C_{66}H_{108}N_{18}O_{14}Se_2$). Isotopic distribution of the relative peak that corresponds to the fragment $y_6$, m/z found $[M+2H]^{2+}$: 695.8007 (top) and isotopic distribution of the simulated peak, m/z calculated $[M+2H]^{2+}$: 695.8050 (bottom)*

## 2.2.1. Metathesis reaction between Linear(Se−Se)2 and $BBSe_2$ under visible light

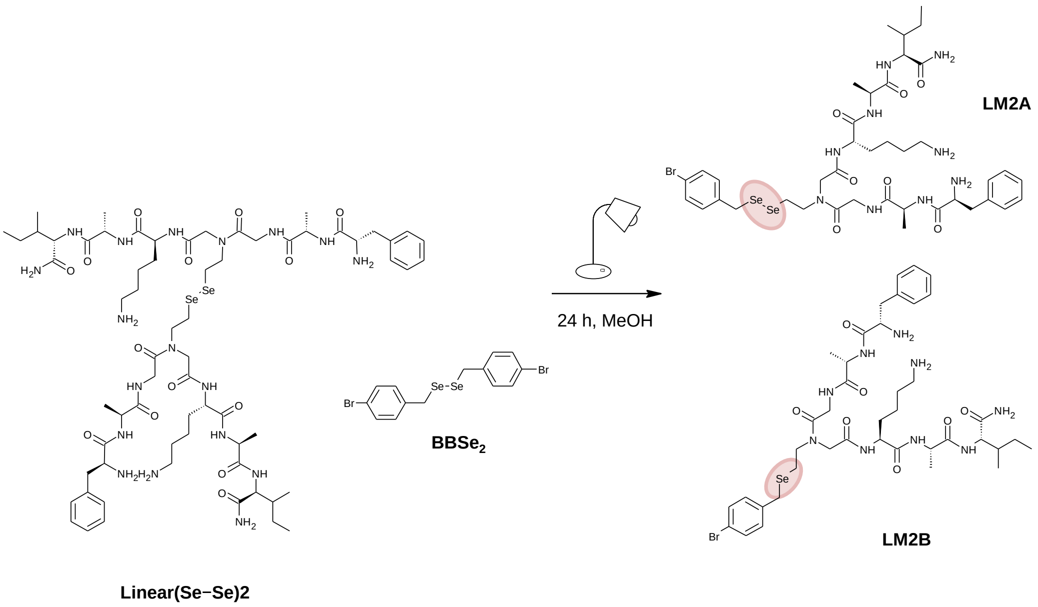


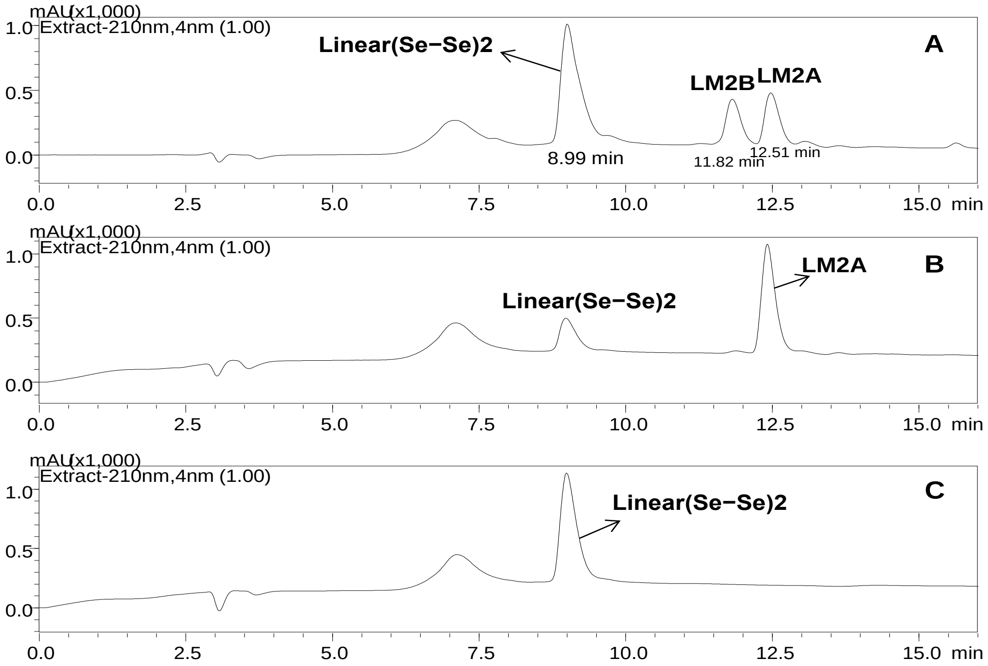


*Figure S33. HPLC chromatograms illustrating the progress of the metathesis reaction between **Linear(Se−Se)2** and **BBSe₂**. Chromatograms (B) and (A) were acquired after 1 h and 24 h of irradiation of the sample under visible light, respectively. The spectrum (C) corresponds to the purified **Linear(Se−Se)2**. Conditions: (5mM) **Linear(Se−Se)2**, (5 mM) **BBSe₂**, methanol and LED lamp 400–700 nm. Retention times (r.t.) of **Linear(Se−Se)2**, **LM2B** and **LM2A** are 8.99 min, 11.82 min and 12.51 min, respectively.*

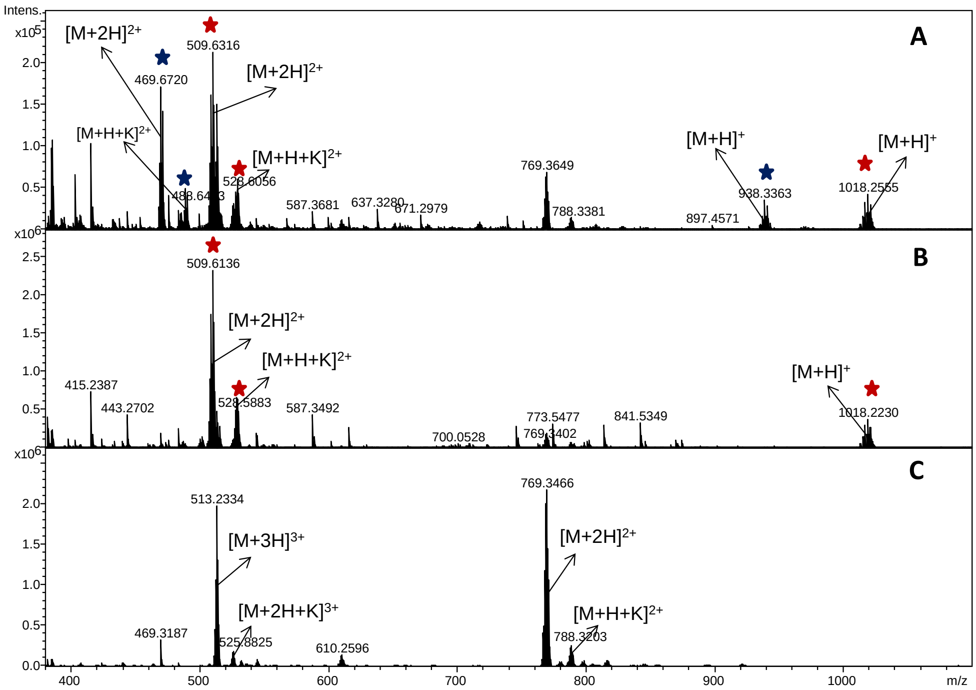


*Figure S34. ESI-qTOF-MS spectra illustrating the progress of the metathesis reaction between **Linear(Se−Se)2** and **BBSe$_2$**. Spectra (B) and (A) were acquired after 1 h and 24 h of irradiation of the sample under visible light, respectively. The spectrum (C) corresponds to the purified **Linear(Se−Se)2**. Conditions: (5mM) **Linear(Se−Se)2**, (5 mM) **BBSe$_2$**, methanol and LED lamp 400–700 nm. Red and dark-blue stars indicate peaks of **LM2A** and **LM2B**, respectively.*

### *2.2.1.1. LM2A*

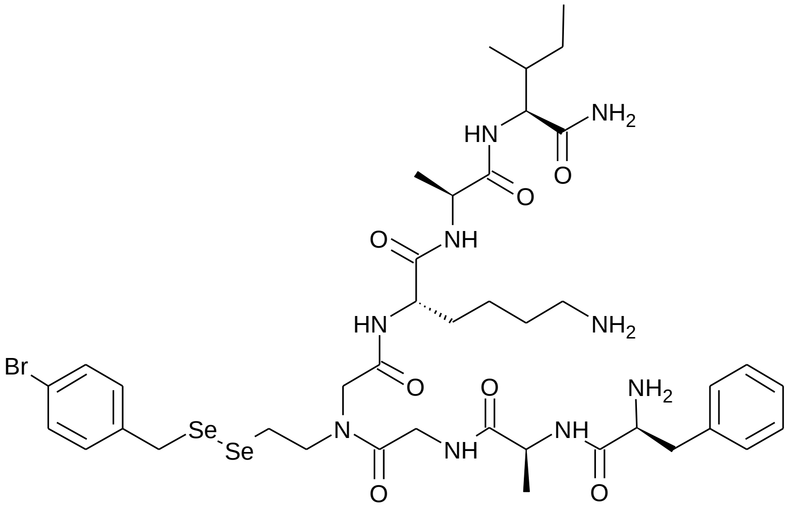

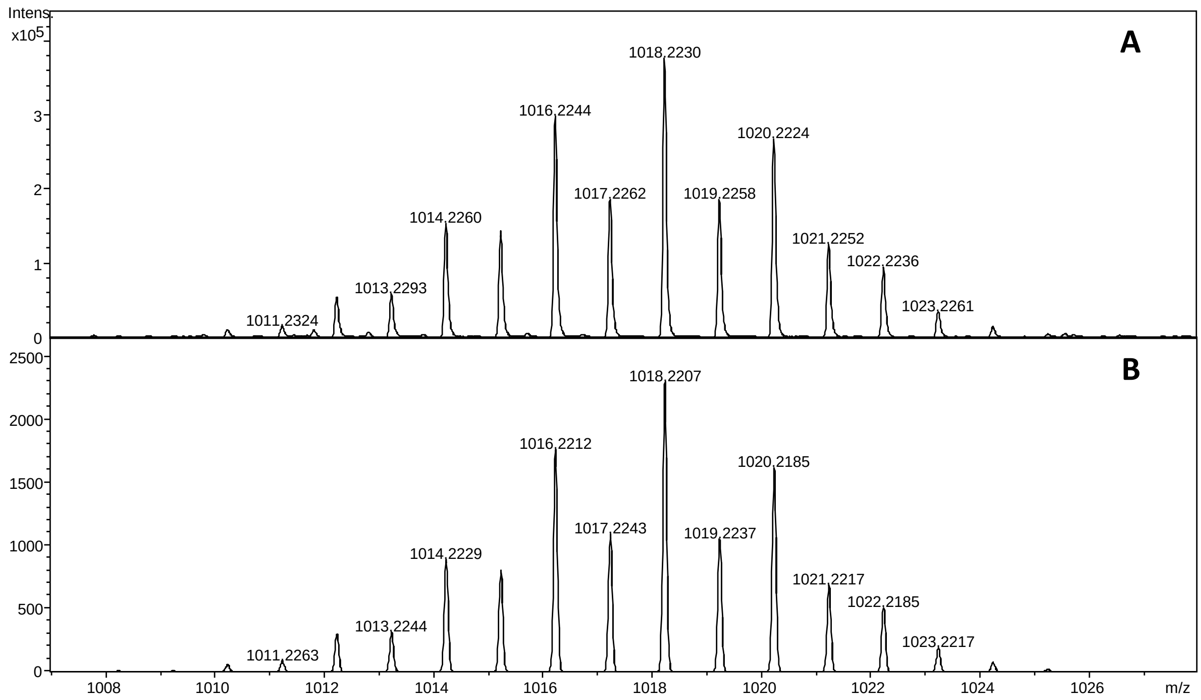


*Figure S35. ESI-qTOF-MS spectrum of **LM2A** ($C_{40}H_{60}BrN_9O_7Se_2$) (A) Isotopic distribution of the relative peak, m/z found $[M+H]^+$: 1018.2230 (B) Isotopic distribution of the simulated peak, m/z calculated $[M+H]^+$: 1018.2207*

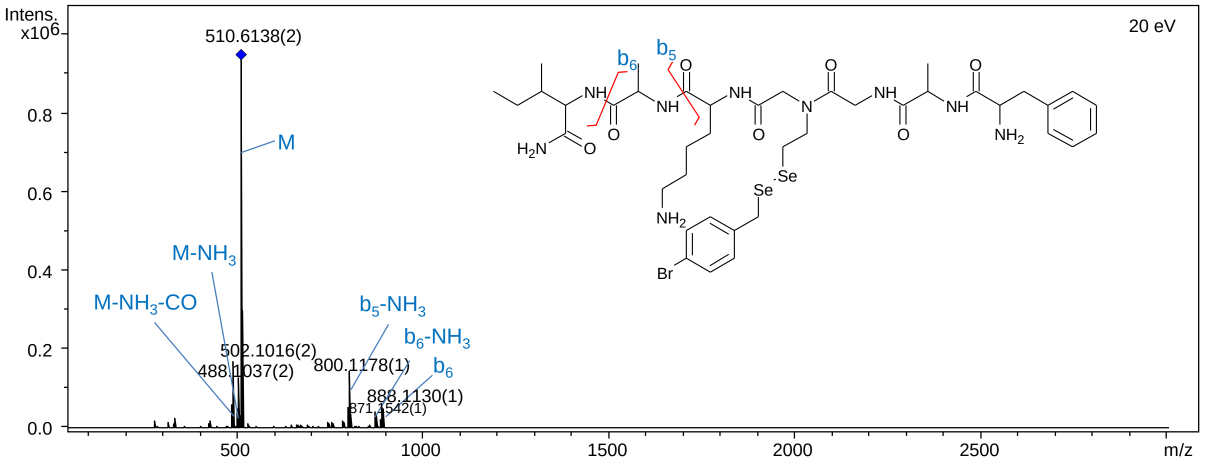


*Figure S36. ESI-qTOF-MS/MS (CE 20 eV) spectrum of **LM2A** ($C_{40}H_{60}BrN_9O_7Se_2$). Precursor ion: m/z $[M+2H]^{2+}$: 510.6138 (calculated for M: 510.6129, z=2+)*

### *2.2.1.2. LM2B*

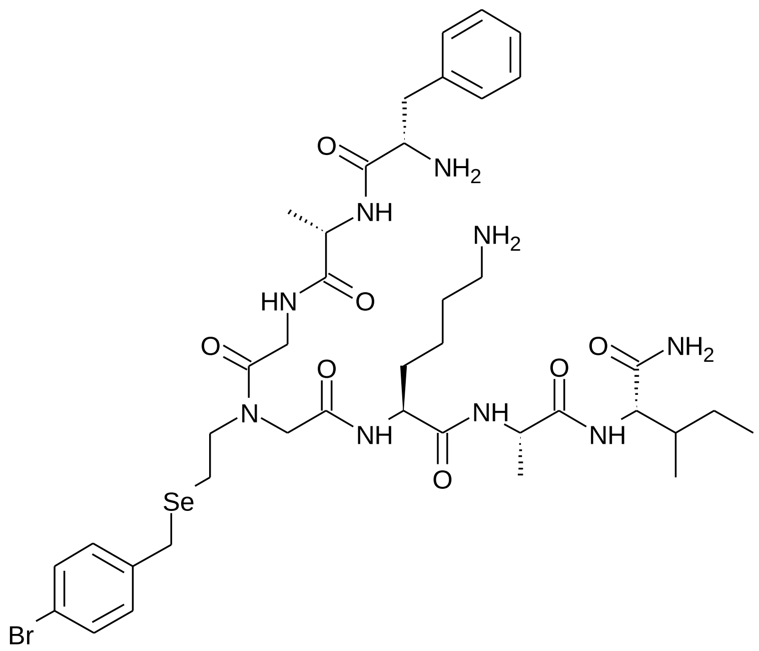


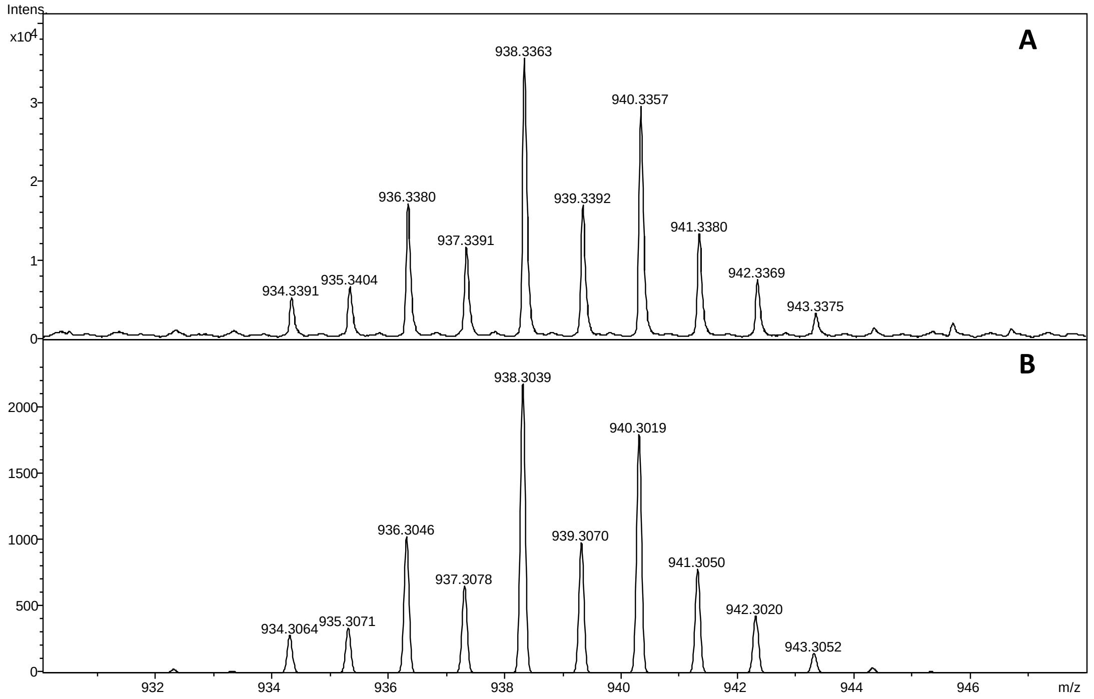


*Figure S37. ESI-qTOF-MS spectrum of **LM2B** ($C_{40}H_{60}BrN_9O_7Se$) (A) Isotopic distribution of the relative peak, m/z found $[M+H]^+$: 938.3363 (B) Isotopic distribution of the simulated peak, m/z calculated $[M+H]^+$: 938.3039*

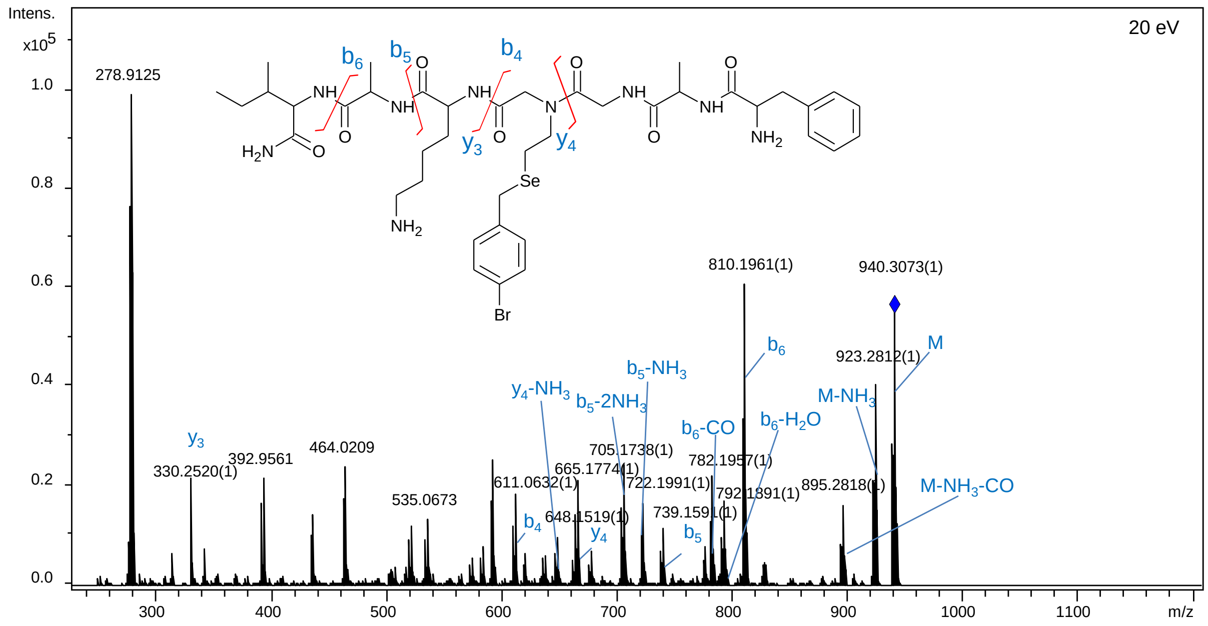


*Figure S38. ESI-qTOF-MS/MS (CE 20 eV) spectrum of **LM2B** ($C_{40}H_{60}BrN_9O_7Se$). Precursor ion: m/z $[M+H]^+$: 940.3073 (calculated for M: 940.3019, z=+)*

## 2.3. Metathesis reaction between Linear(Se−Se)1 and Linear(Se−Se)2 under visible light

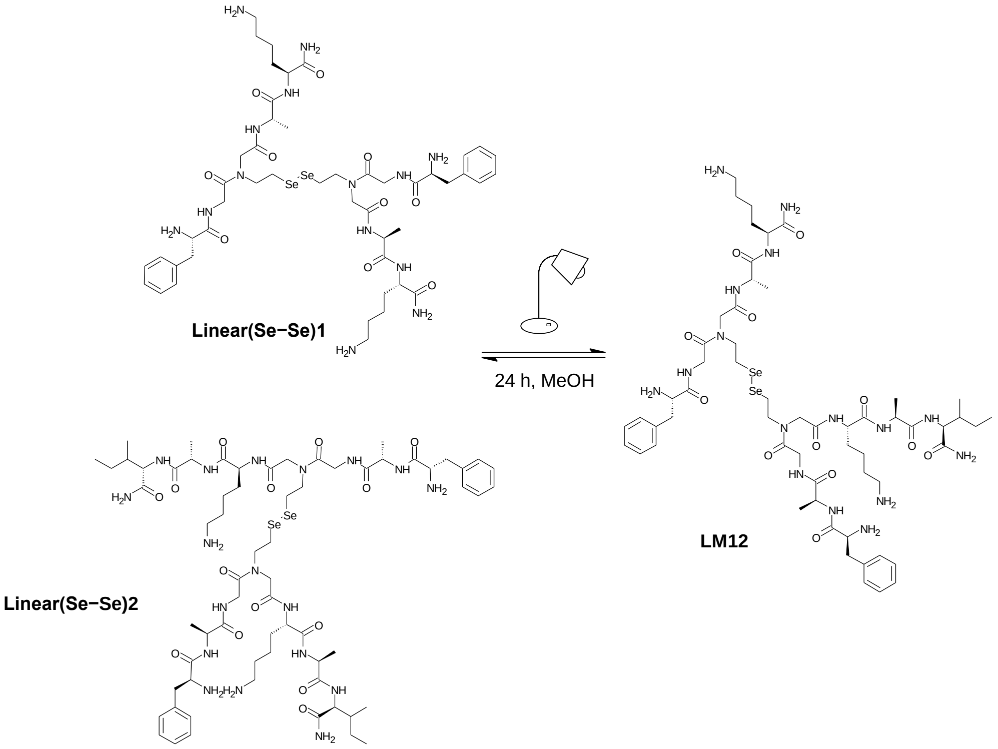

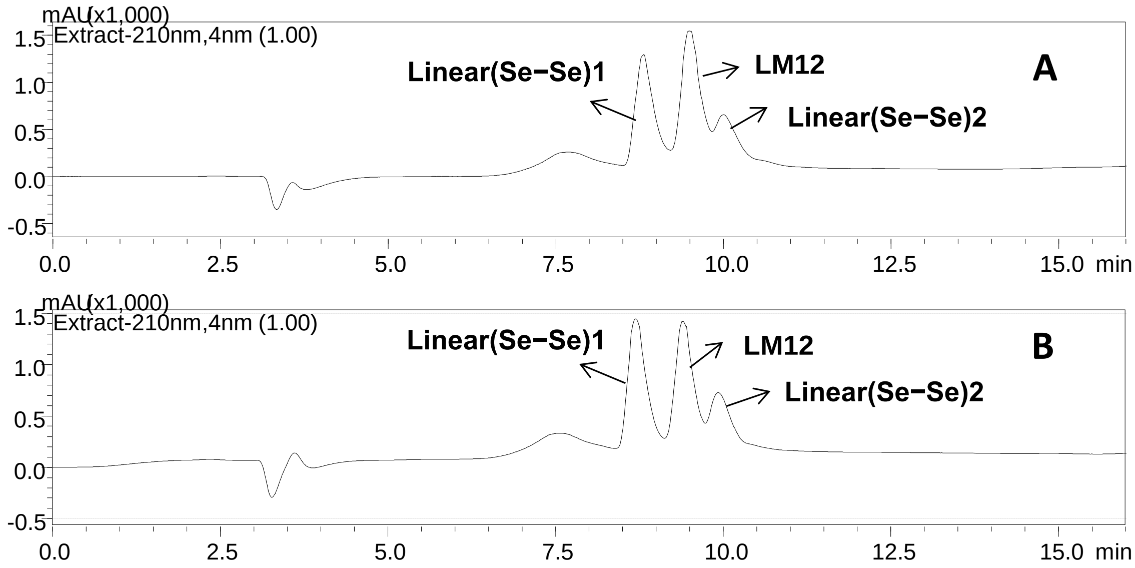


*Figure S39. HPLC chromatograms illustrating the progress of the metathesis reaction between **Linear(Se−Se)1** and **Linear(Se−Se)2**. Chromatograms (B) and (A) were acquired after 1 h and 24 h of irradiation of the sample under visible light, respectively. Conditions: (5mM) **Linear(Se−Se)1**, (5 mM) **Linear(Se−Se)2**, methanol and LED lamp 400–700 nm. Retention times (r.t.) of **Linear(Se−Se)1**, **LM12** and **Linear(Se−Se)2** are 8.81 min, 9.49 min and 10.03 min., respectively.*

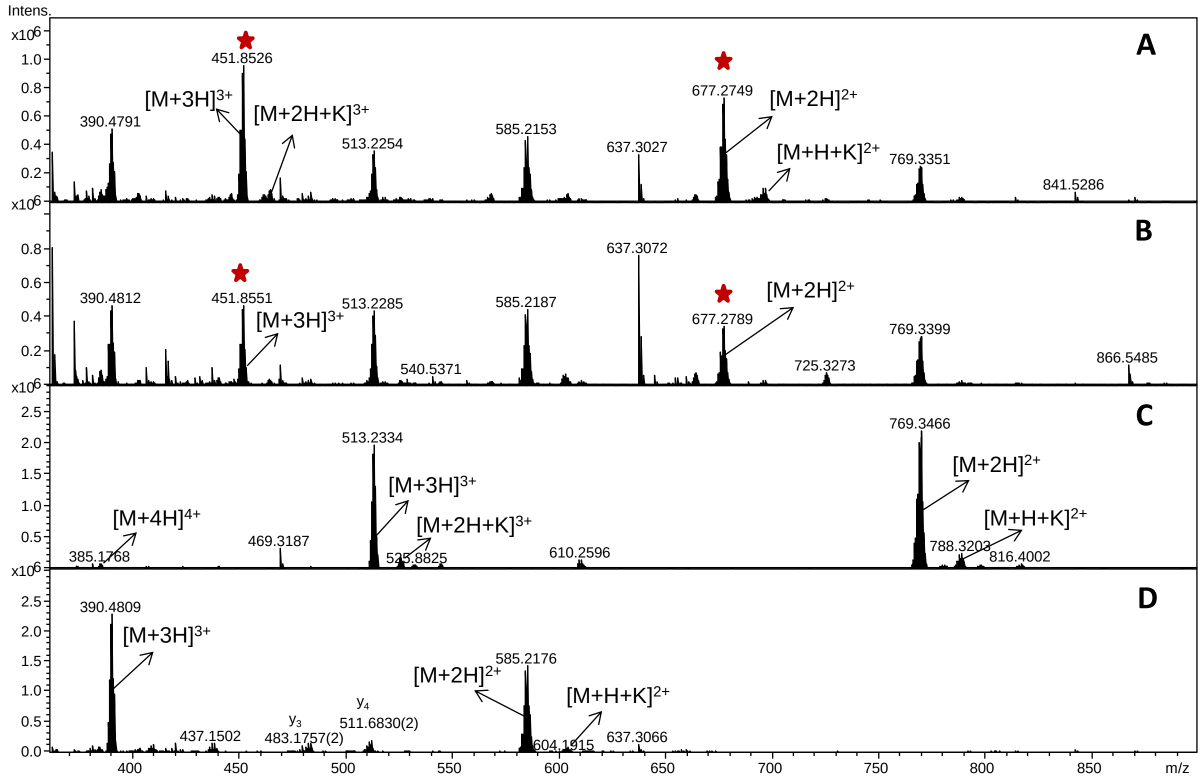


*Figure S40. ESI-qTOF-MS spectra illustrating the progress of the metathesis reaction between **Linear(Se−Se)1** and **Linear(Se−Se)2**. Spectra (B) and (A) were acquired after 1 h and 24 h of irradiation of the sample under visible light, respectively. Spectra (C) and (D) correspond to the purified **Linear(Se−Se)2** and **Linear(Se−Se)1**, respectively. Conditions: (5mM) **Linear(Se−Se)1**, (5 mM) **Linear(Se−Se)2**, methanol and LED lamp 400–700 nm. The red star indicates the peak of **LM12**.*

## 2.3.1. LM12

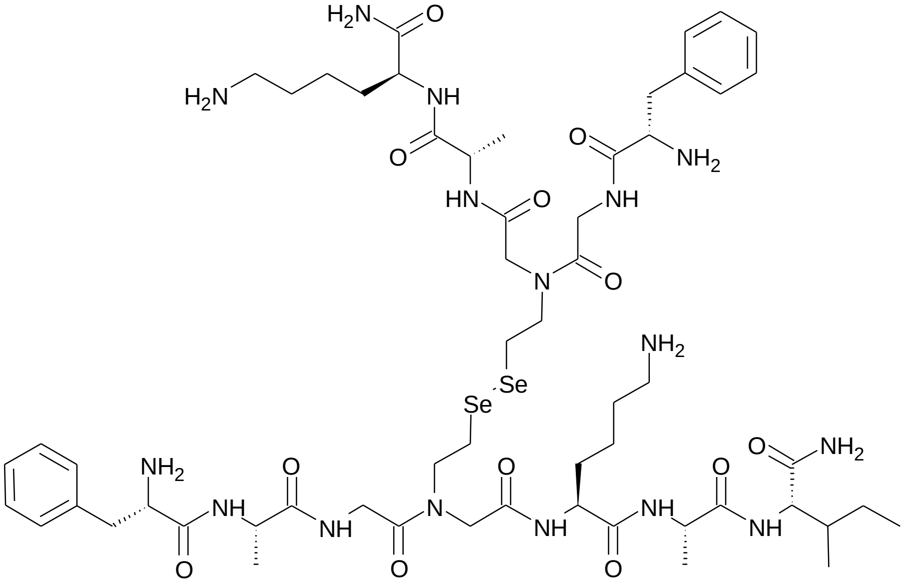

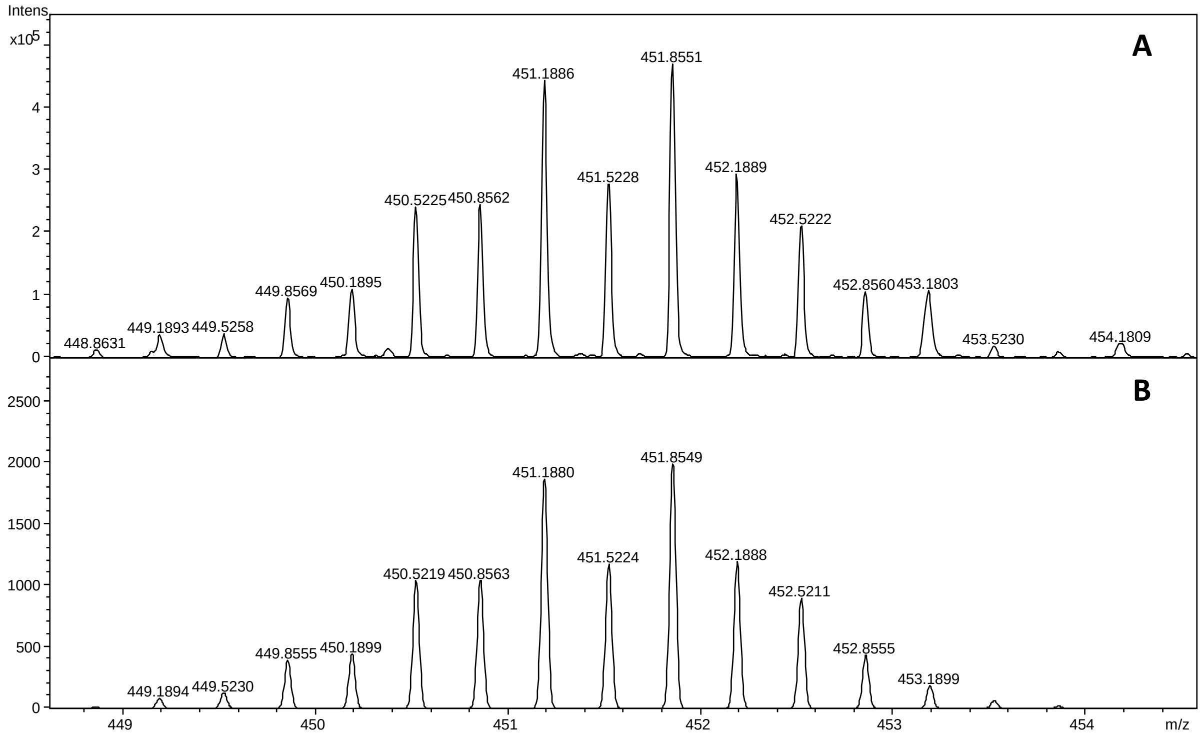


*Figure S41. ESI-qTOF-MS spectrum of **LM12** ($C_{57}H_{92}N_{16}O_{12}Se_2$) (A) Isotopic distribution of the relative peak, m/z found $[M+3H]^{3+}$: 451.8551 (B) Isotopic distribution of the simulated peak, m/z calculated $[M+3H]^{3+}$: 451.8549*

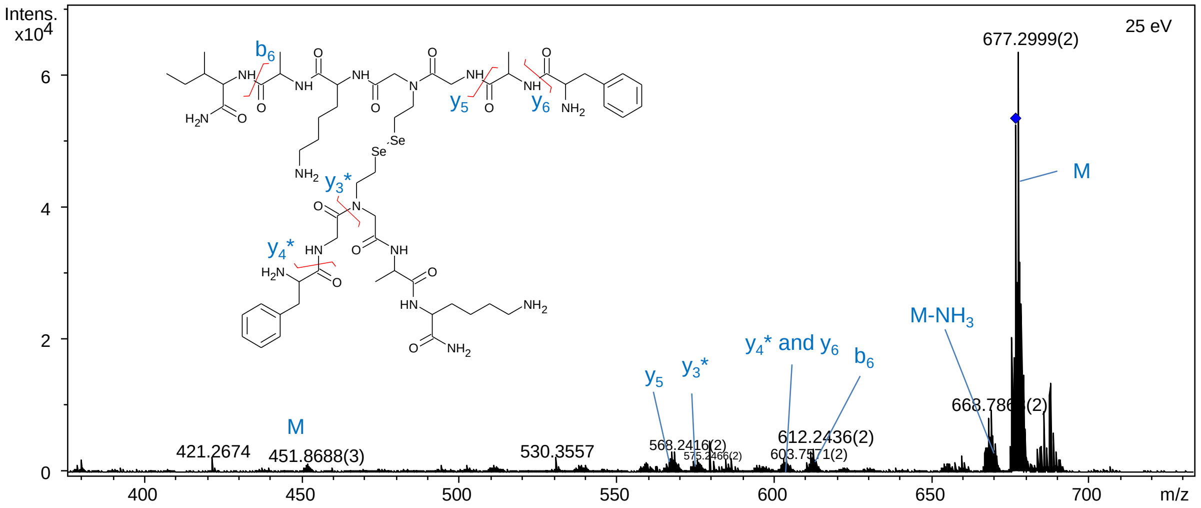


*Figure S42. ESI-qTOF-MS/MS (CE 25 eV) spectrum of **LM12** ($C_{57}H_{92}N_{16}O_{12}Se_2$). Precursor ion: m/z $[M+2H]^{2+}$: 677.2999 (calculated for M: 677.2786, z=2+)*

## 2.4. Linear(Se−Se)3

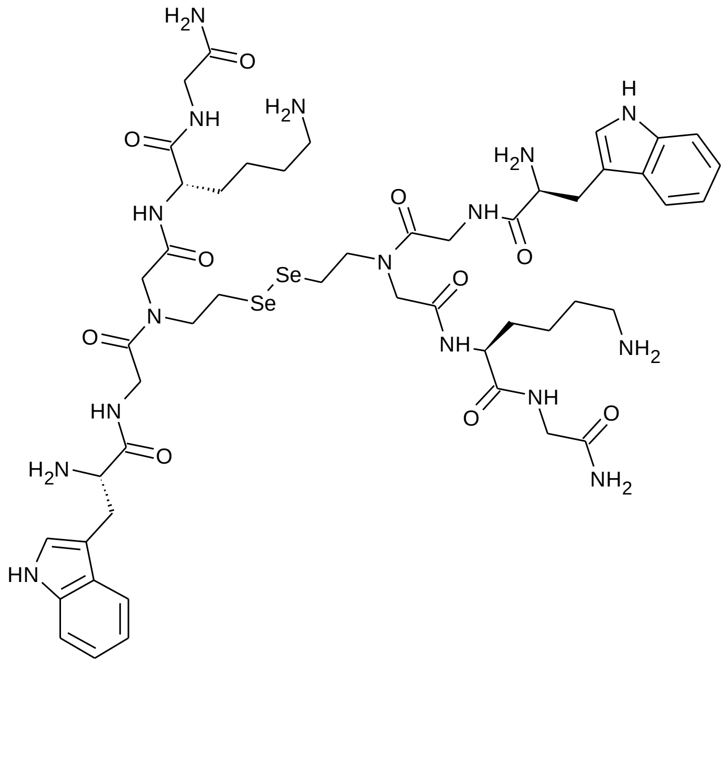

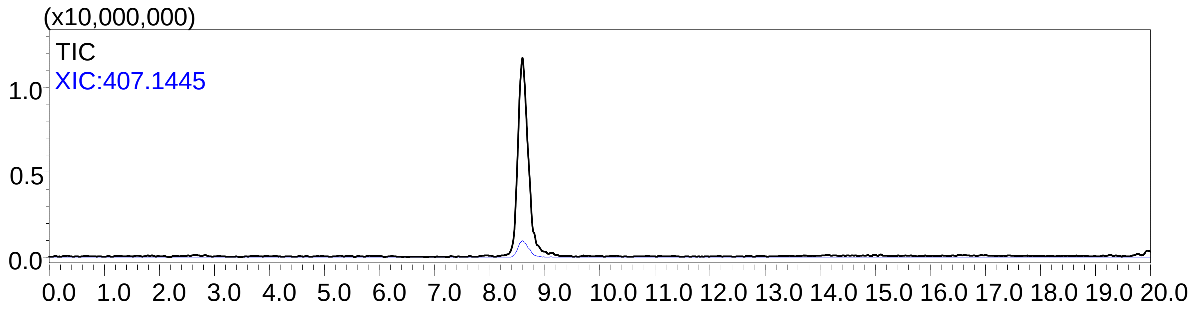


*Figure S43. LC-MS chromatogram of **Linear(Se−Se)3** (retention time: 8.60 min)*

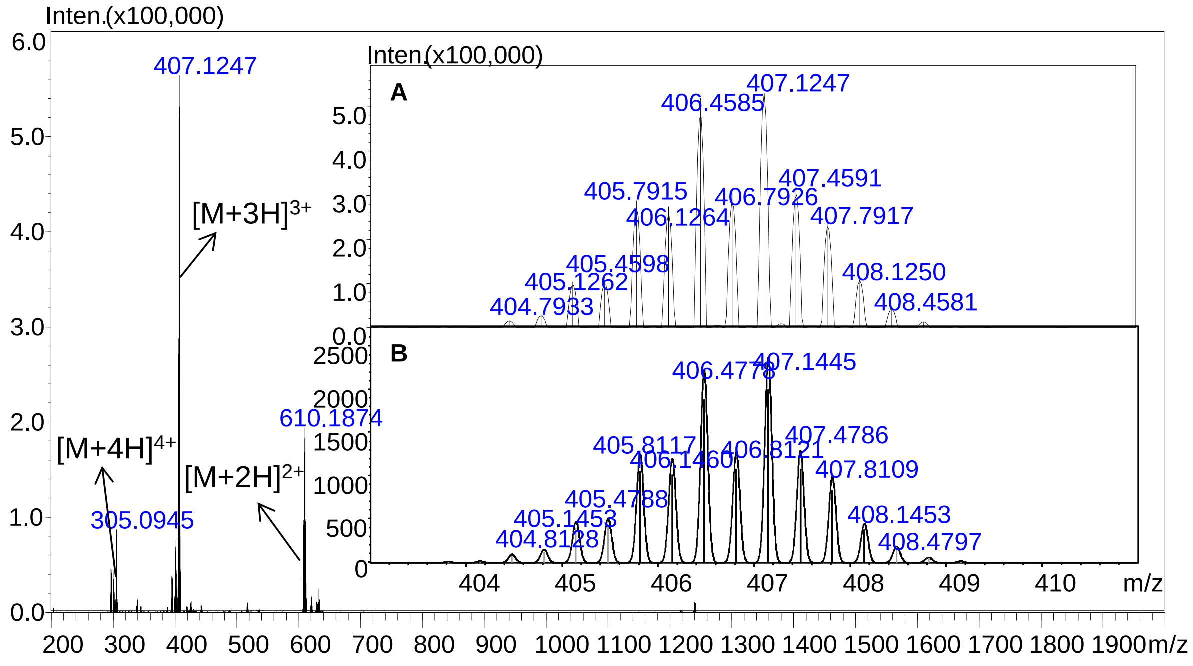


*Figure S44. LC-ESI-IT-TOF-MS spectrum of **Linear(Se−Se)3** ($C_{50}H_{74}N_{16}O_{10}Se_2$) (A) Isotopic distribution of the relative peak, m/z found $[M+3H]^{3+}$: 407.1247 (B) Isotopic distribution of the simulated peak, m/z calculated $[M+3H]^{3+}$: 407.1445*

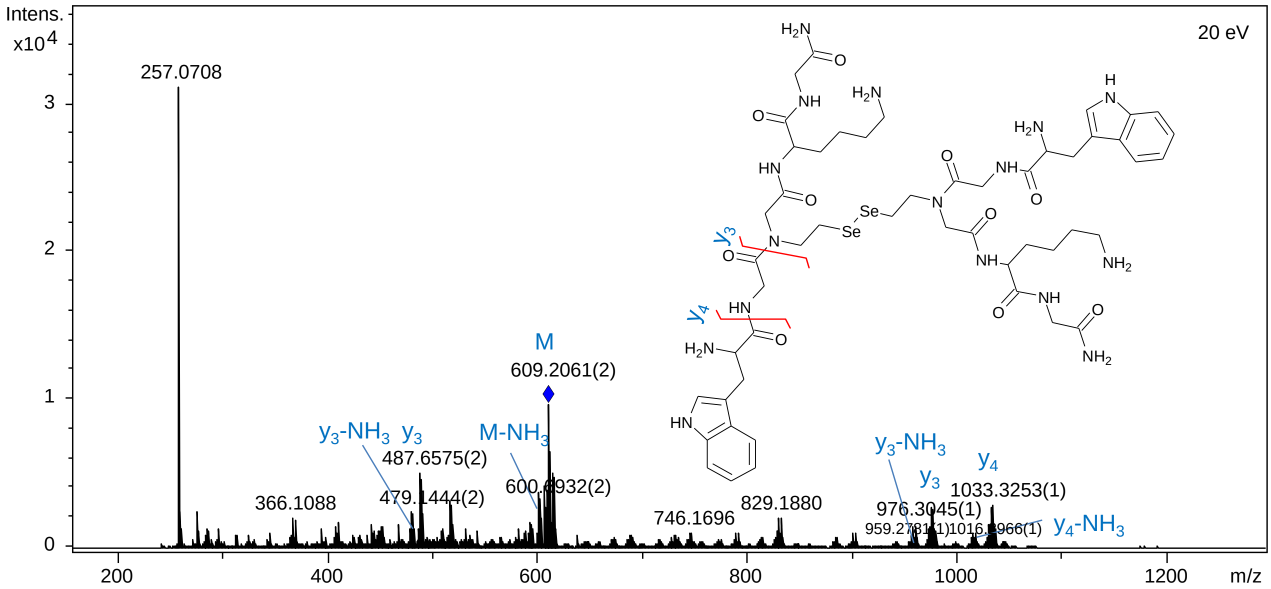


*Figure S45. ESI-qTOF-MS/MS (CE 20 eV) spectrum of **Linear(Se−Se)3** ($C_{50}H_{74}N_{16}O_{10}Se_2$). Precursor ion: m/z $[M+2H]^{2+}$: 609.2061 (calculated for M: 609.2130, z=2+)*

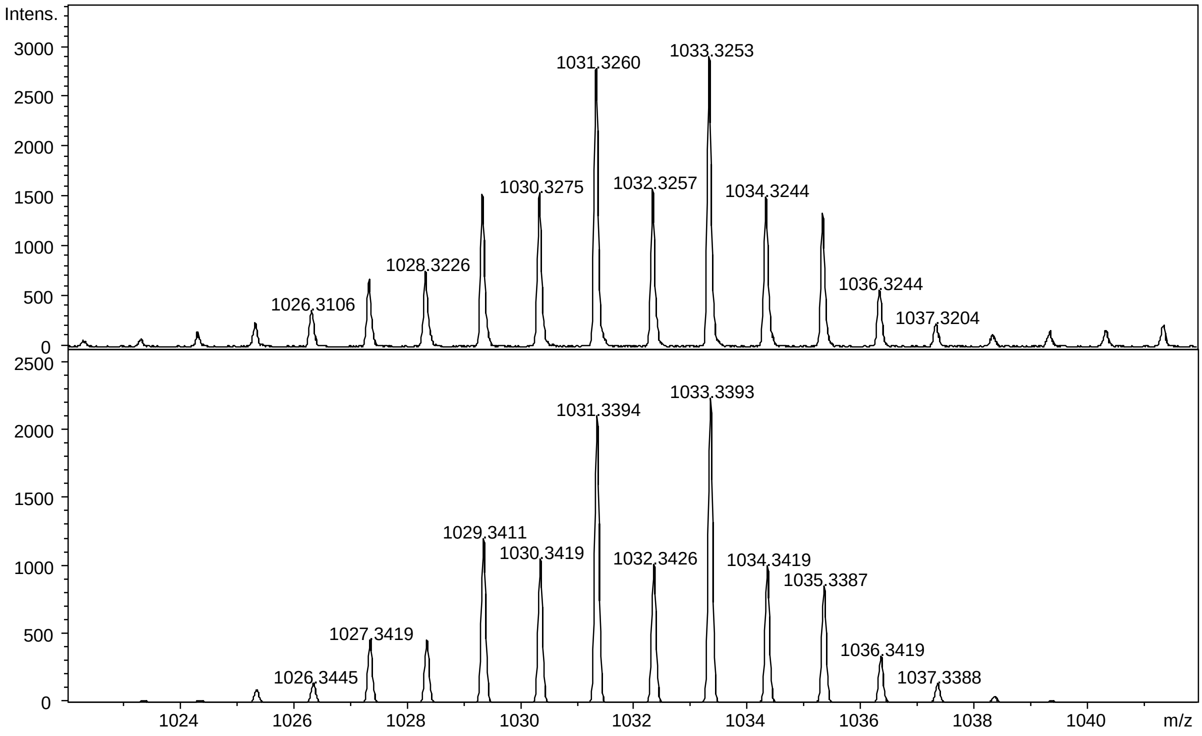


*Figure S46. ESI-qTOF-MS/MS (CE 20 eV) spectrum of **Linear(Se−Se)3** ($C_{50}H_{74}N_{16}O_{10}Se_2$). Isotopic distribution of the relative peak that corresponds to the fragment $y_4$, m/z found $[M+H]^+$: 1033.3253 (top) and isotopic distribution of the simulated peak, m/z calculated $[M+H]^+$: 1033.3393 (bottom)*

## 2.4.1. Metathesis reaction between Linear(Se−Se)3 and $BBSe_2$ under visible light

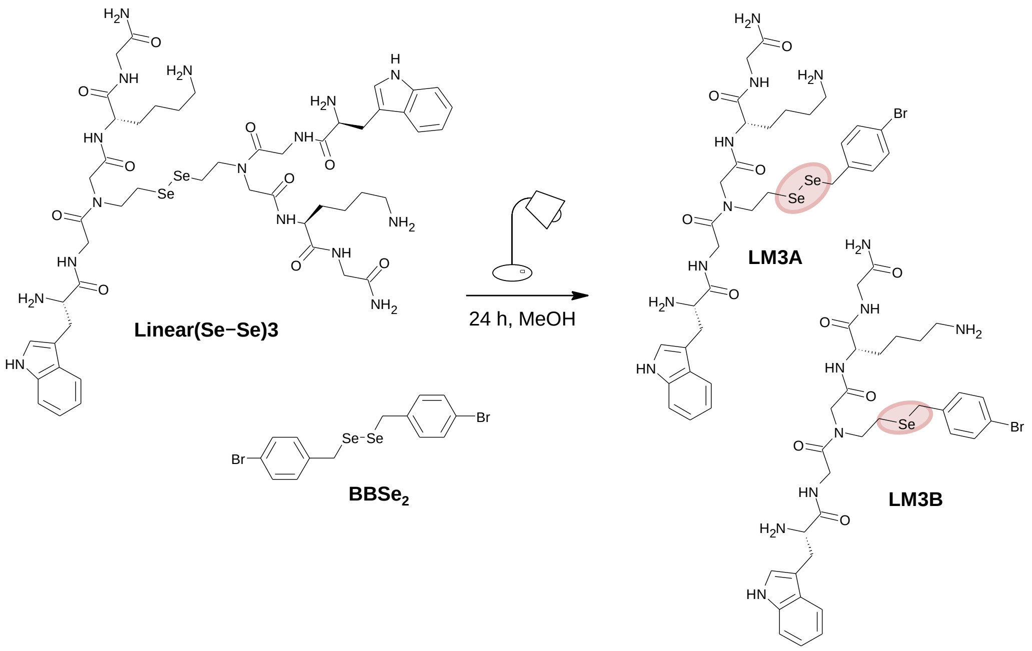


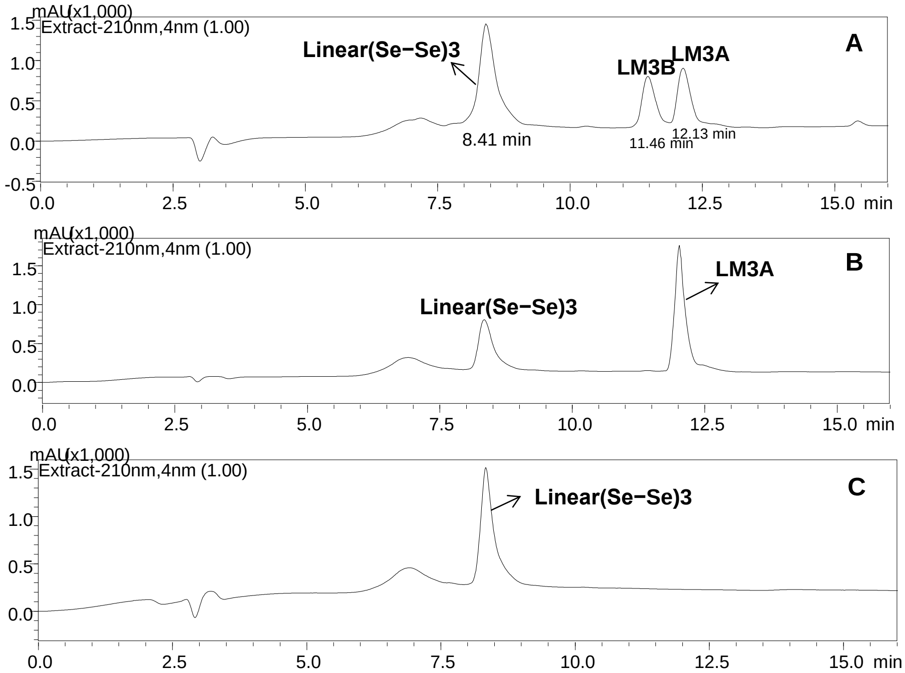


*Figure S47. HPLC chromatograms illustrating the progress of the metathesis reaction between* ***Linear(Se−Se)3*** *and* ***$BBSe_2$****. Chromatograms (B) and (A) were acquired after 1 h and 24 h of irradiation of the sample under visible light, respectively. Chromatogram (C) corresponds to the purified* ***Linear(Se−Se)3****. Conditions: (5 mM)* ***Linear(Se−Se)3****, (5 mM)* ***$BBSe_2$****, methanol and LED lamp 400–700 nm. Retention times (r.t.) of* ***Linear(Se−Se)3****,* ***LM3B*** *and* ***LM3A*** *are 8.41 min, 11.46 min and 12.13 min, respectively.*

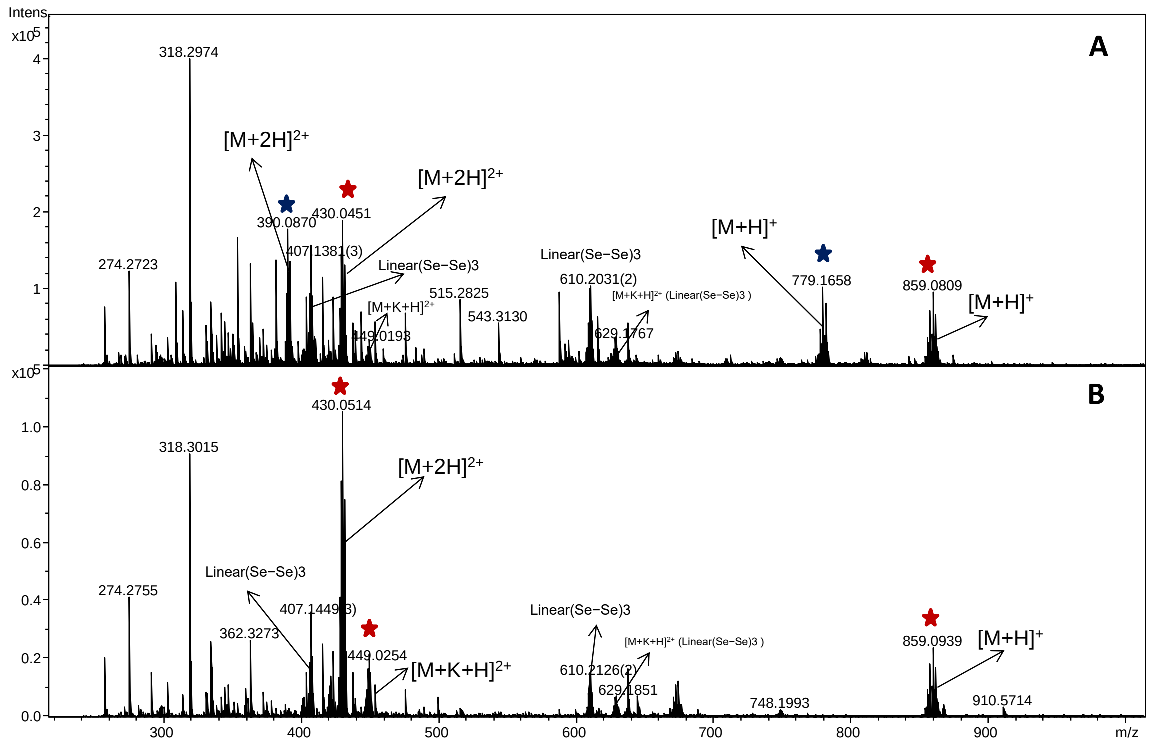


*Figure S48. ESI-qTOF-MS spectra illustrating the progress of the metathesis reaction between **Linear(Se−Se)3** and **BBSe$_2$**. Spectra (B) and (A) were acquired after 1 h and 24 h of irradiation of the sample under visible light, respectively. Conditions: (5 mM) **Linear(Se−Se)3**, (5 mM) **BBSe$_2$**, methanol and LED lamp 400–700 nm. Red and dark-blue stars indicate peaks of **LM3A** and **LM3B**, respectively.*

## 2.4.1.1. LM3A

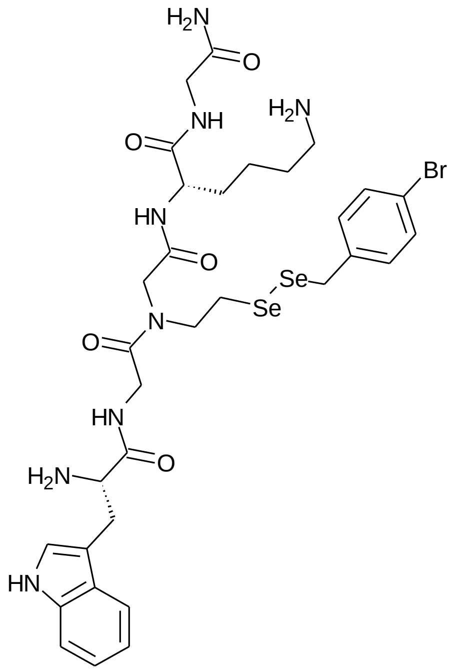

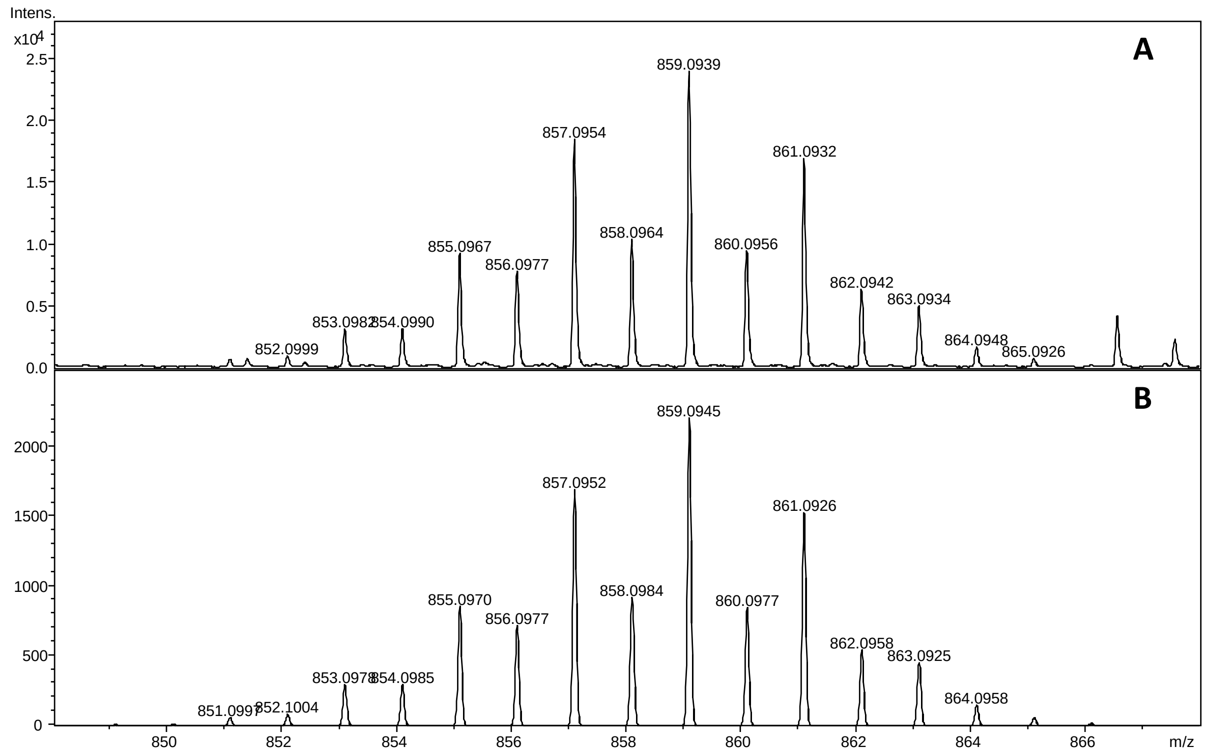


*Figure S49. ESI-qTOF-MS spectrum of **LM3A** ($C_{32}H_{43}BrN_8O_5Se_2$) (A) Isotopic distribution of the relative peak, m/z found $[M+H]^+$: 859.0939 (B) Isotopic distribution of the simulated peak, m/z calculated $[M+H]^+$: 859.0945*

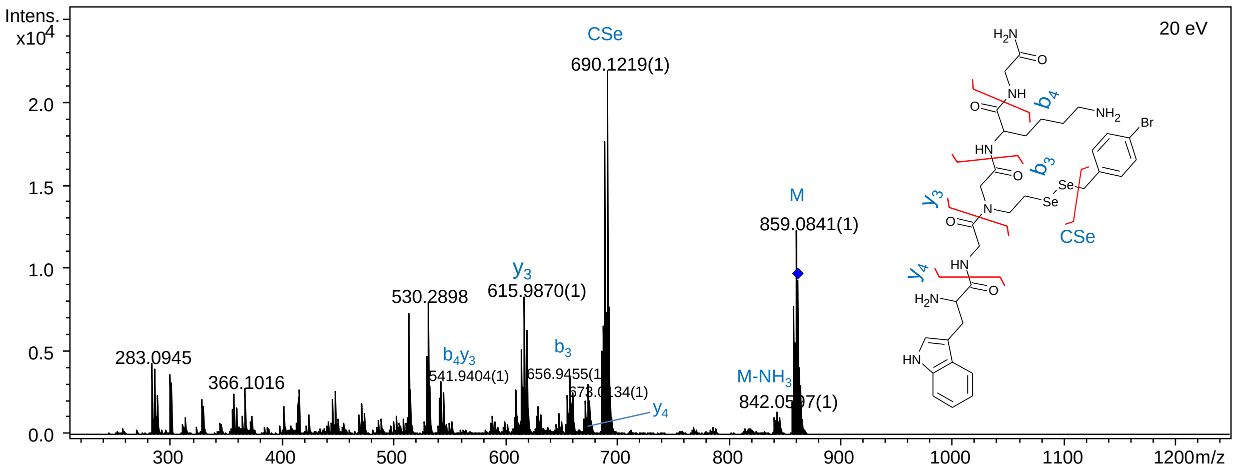


*Figure S50. ESI-qTOF-MS/MS (CE 20 eV) spectrum of **LM3A** ($C_{32}H_{43}BrN_8O_5Se_2$). Precursor ion: m/z $[M+H]^+$: 859.0841 (calculated for M: 859.0945, z=+) The notation "CSe" was used to indicate cleavage of the C−Se bond.*

### 2.4.1.2. LM3B

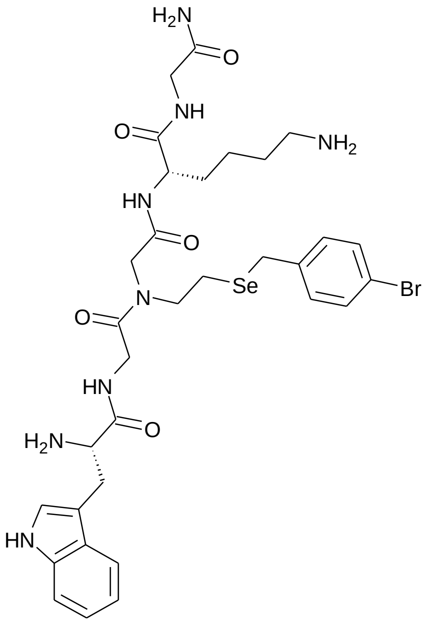


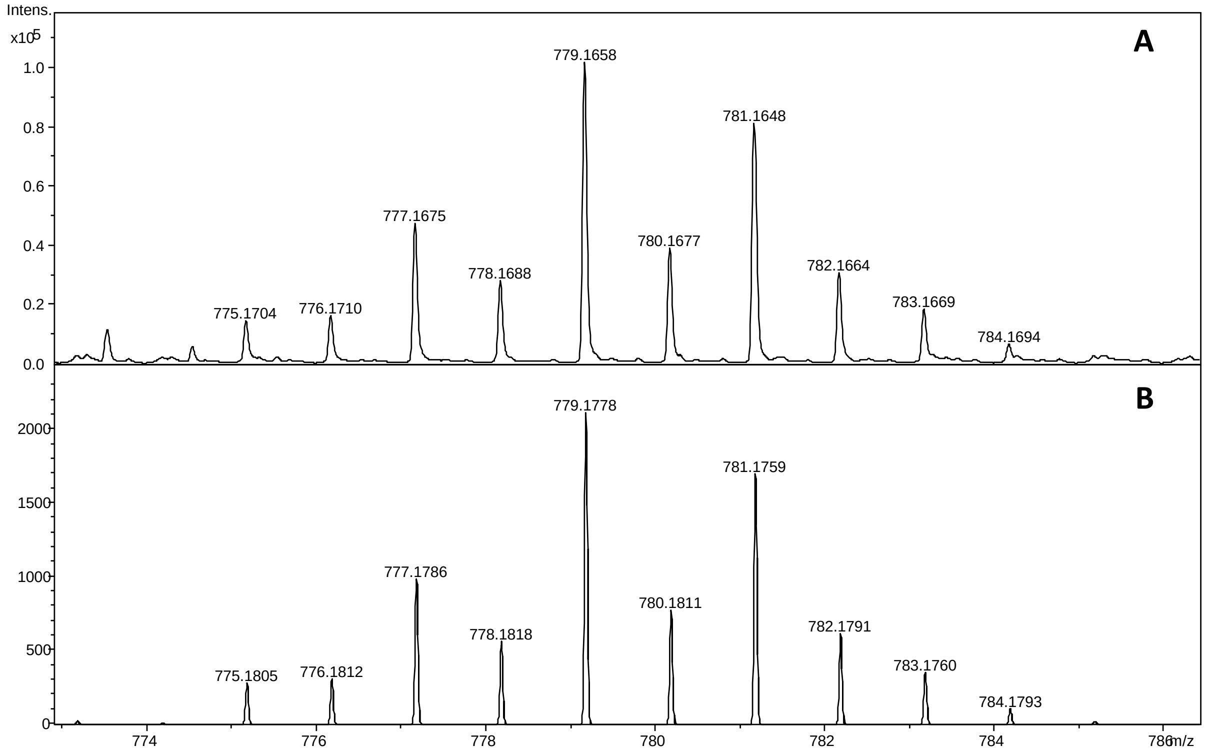


*Figure S51. ESI-qTOF-MS spectrum of **LM3B** ($C_{32}H_{43}BrN_8O_5Se$) (A) Isotopic distribution of the relative peak, m/z found $[M+H]^+$: 779.1658 (B) Isotopic distribution of the simulated peak, m/z calculated $[M+H]^+$: 779.1778*

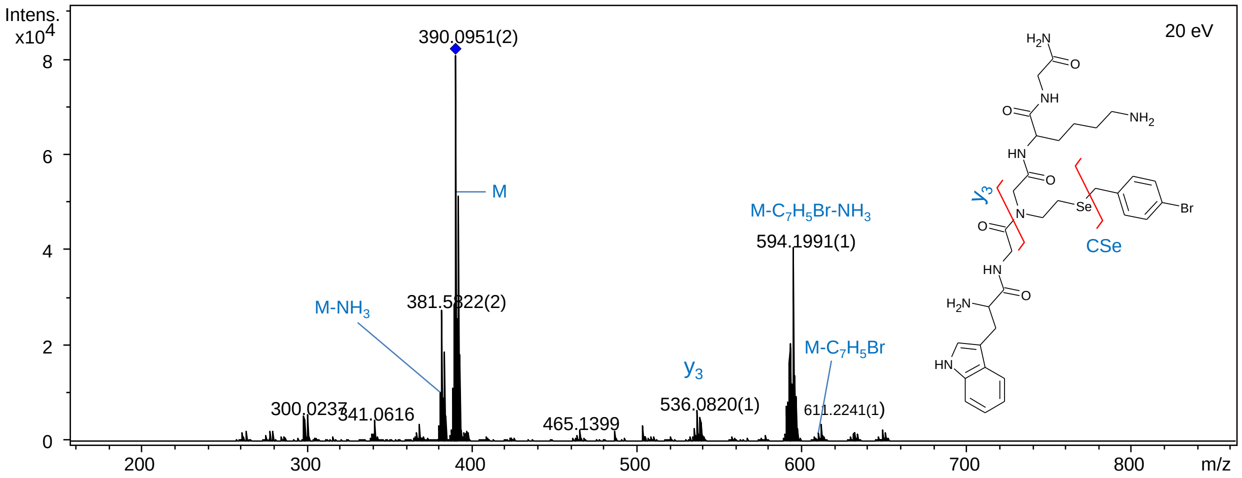


*Figure S52. ESI-qTOF-MS/MS (CE 20 eV) spectrum of **LM3B** ($C_{32}H_{43}BrN_8O_5Se$). Precursor ion: m/z $[M+2H]^{2+}$: 390.0951 (calculated for M: 390.0925, z=2+) The notation "CSe" was used to indicate cleavage of the C−Se bond.*

## 2.5. Cyclo(Se−Se)1

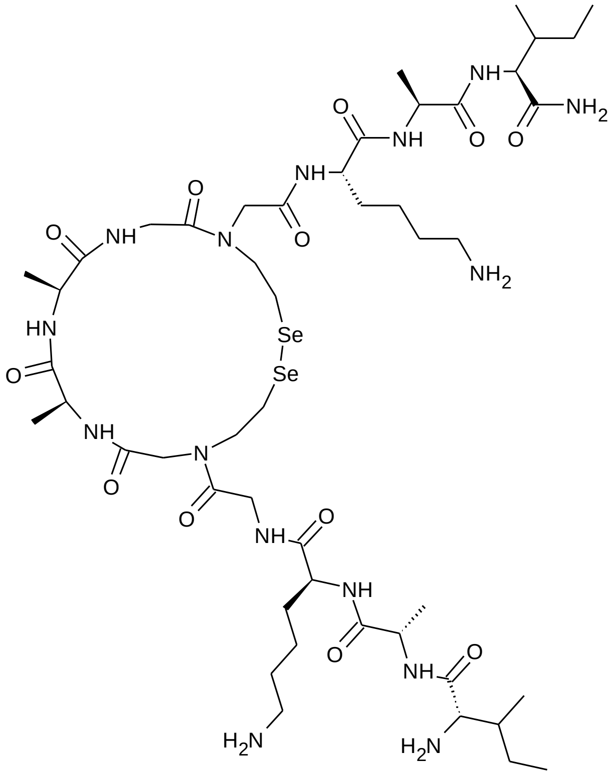

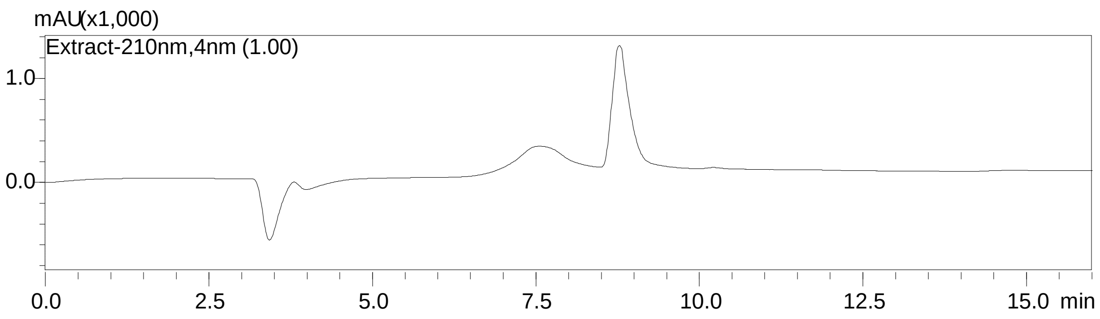

*Figure S53. HPLC chromatogram of **Cyclo(Se−Se)1** (retention time: 8.77 min)*

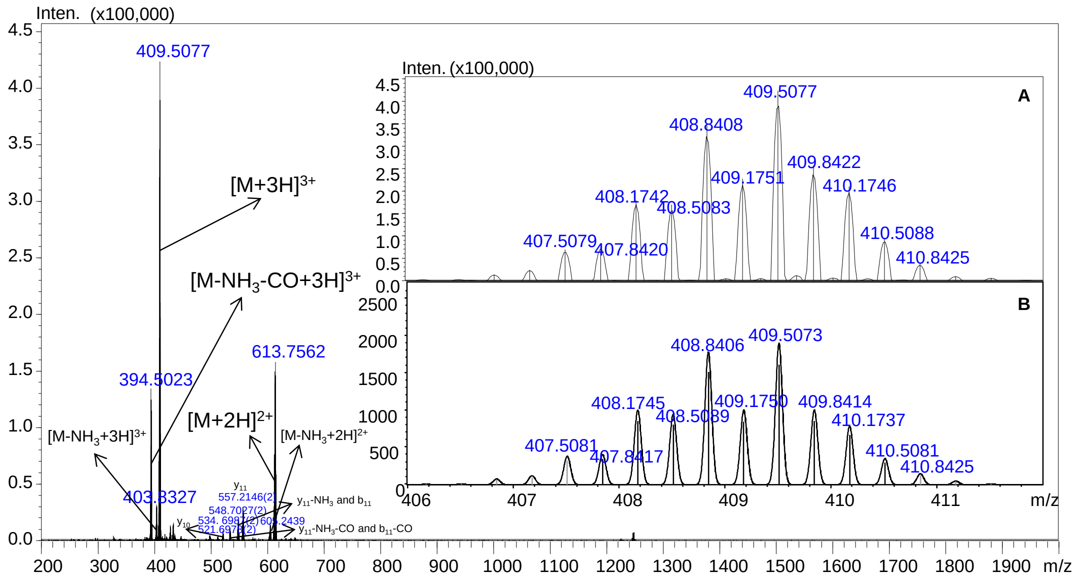

*Figure S54. LC-ESI-IT-TOF-MS spectrum of **Cyclo(Se−Se)1** ($C_{48}H_{87}N_{15}O_{12}Se_2$) (A) Isotopic distribution of the relative peak, m/z found $[M+3H]^{3+}$: 409.5077 (B) Isotopic distribution of the simulated peak, m/z calculated $[M+3H]^{3+}$: 409.5073 (Fragmentation was observed in MS spectrum.)*

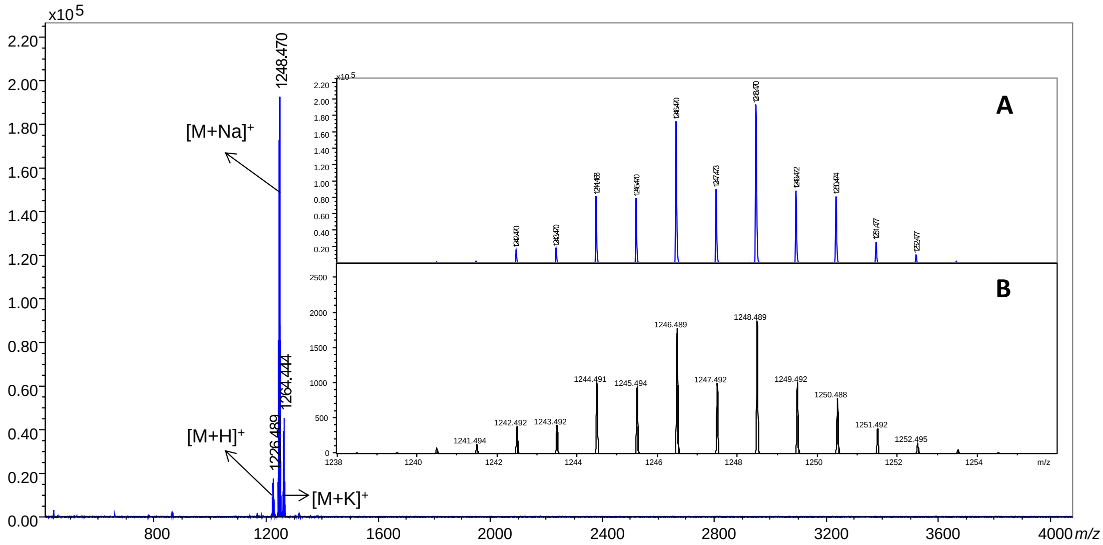

*Figure S55. MALDI-MS spectrum of **Cyclo(Se−Se)1** ($C_{48}H_{87}N_{15}O_{12}Se_2$) (A) Isotopic distribution of the relative peak, m/z found $[M+Na]^+$: 1248.470 (B) Isotopic distribution of the simulated peak, m/z calculated $[M+Na]^+$: 1248.489*

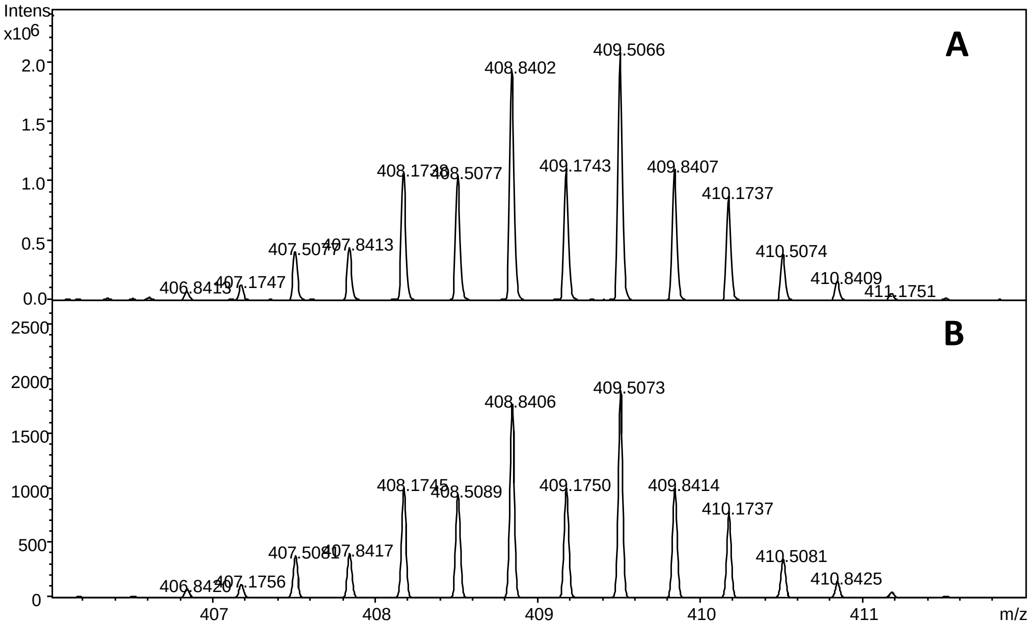


*Figure S56. ESI-qTOF-MS spectrum of **Cyclo(Se−Se)1** ($C_{48}H_{87}N_{15}O_{12}Se_2$) (A) Isotopic distribution of the relative peak, m/z found $[M+3H]^{3+}$: 409.5066 (B) Isotopic distribution of the simulated peak, m/z calculated $[M+3H]^{3+}$: 409.5073*

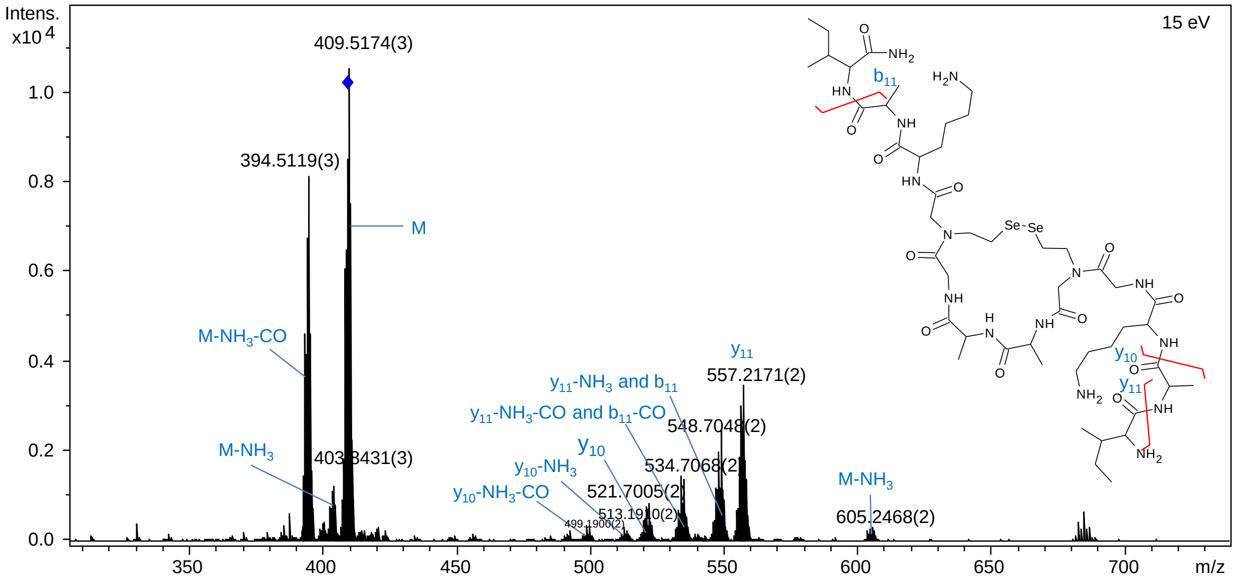


*Figure S57. ESI-qTOF-MS/MS (CE 15 eV) spectrum of **Cyclo(Se−Se)1** ($C_{48}H_{87}N_{15}O_{12}Se_2$). Precursor ion: m/z $[M+3H]^{3+}$: 409.5174 (calculated for M: 409.5073, z=3+)*

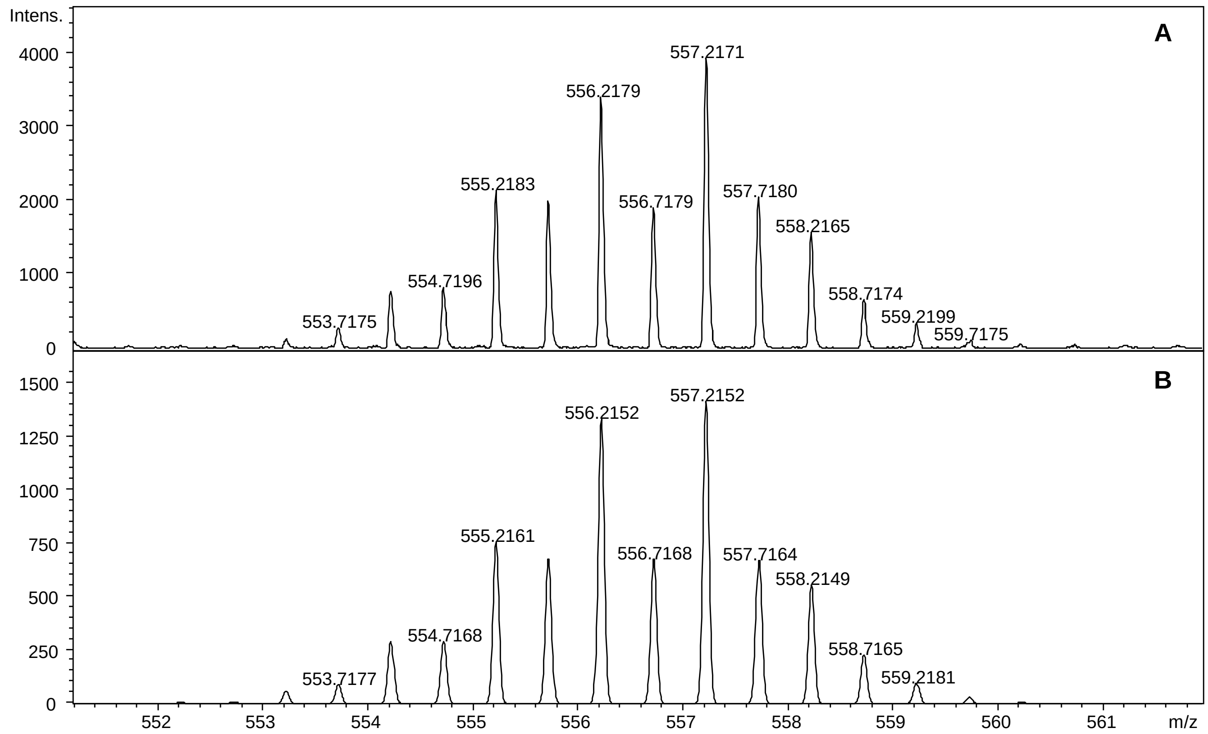


*Figure S58. ESI-qTOF-MS/MS (CE 15 eV) spectrum of **Cyclo(Se−Se)1** ($C_{48}H_{87}N_{15}O_{12}Se_2$). (A) Isotopic distribution of the relative peak that corresponds to the fragment $y_{11}$, m/z found $[M+2H]^{2+}$: 557.2171 (B) Isotopic distribution of the simulated peak, m/z calculated $[M+2H]^{2+}$: 557.2152*

## 2.5.1. Metathesis reaction between Cyclo(Se−Se)1 and BBSe$_2$ under visible light

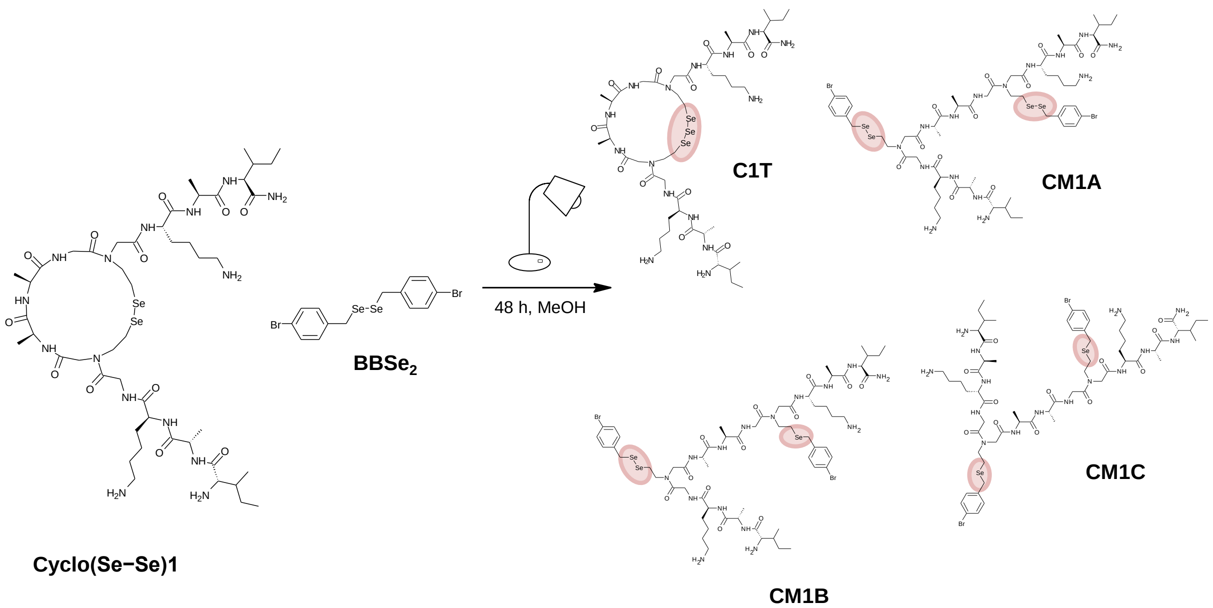

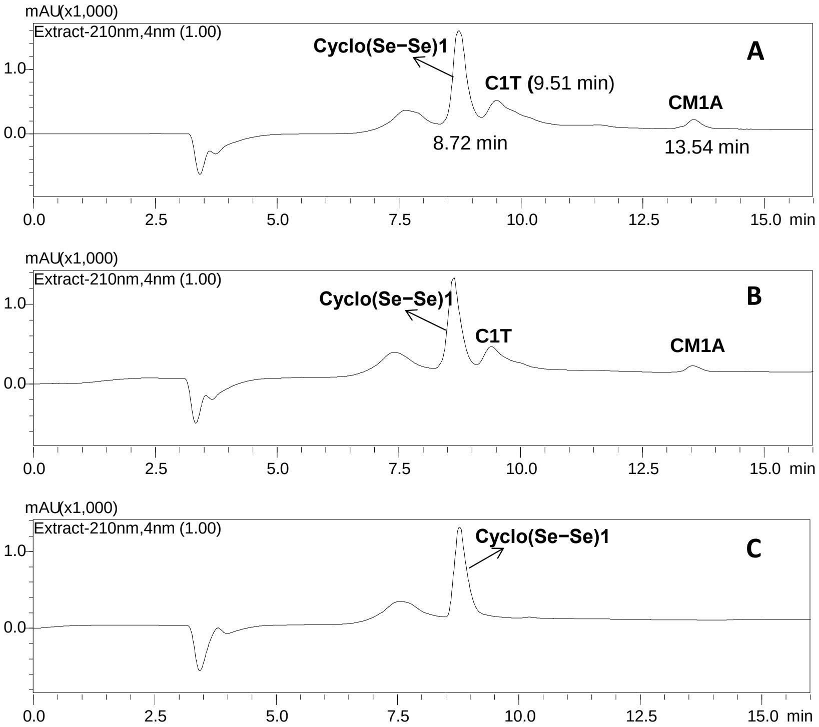


*Figure S59. HPLC chromatograms illustrating the progress of the metathesis reaction between **Cyclo(Se−Se)1** and **BBSe$_2$**. Chromatograms (B) and (A) were acquired after 1 h and 24 h of irradiation of the sample under visible light, respectively. The chromatogram (C) corresponds to the purified **Cyclo(Se−Se)1**. Conditions: (5 mM) **Cyclo(Se−Se)1**, (5 mM) **BBSe$_2$**, methanol and LED lamp 400–700 nm. Retention times (r.t.) of **Cyclo(Se−Se)1**, **C1T** and **CM1A** are 8.72 min, 9.51 min and 13.54 min, respectively.*

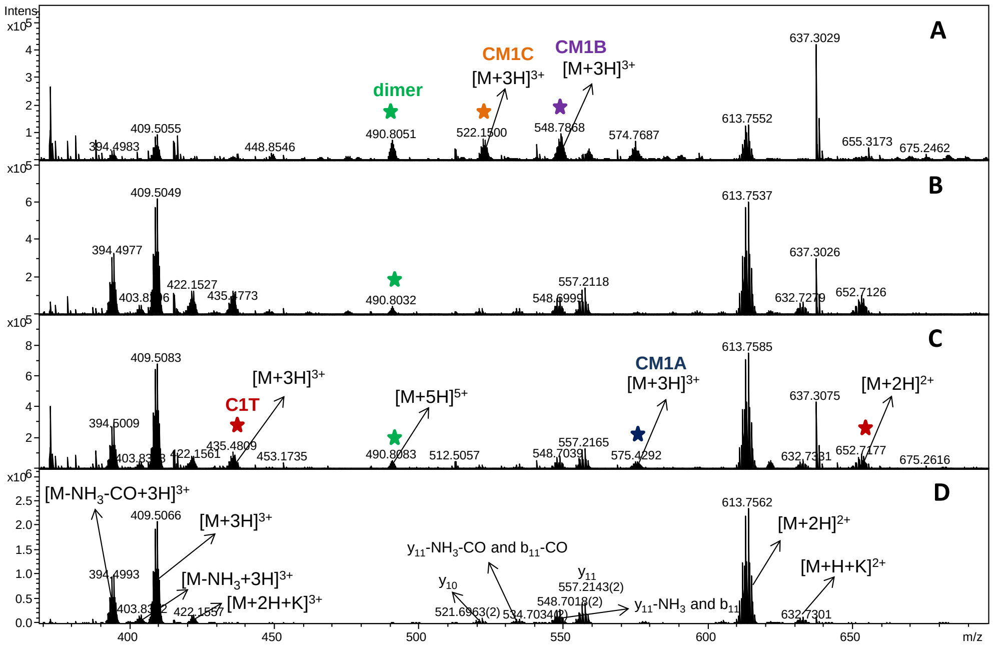


*Figure S60. ESI-qTOF-MS spectra illustrating the progress of the metathesis reaction between **Cyclo(Se−Se)1** and **BBSe$_2$**. Spectra (C), (B) and (A) were acquired after 1 h, 24 h and 48 h of irradiation of the sample under visible light, respectively. The spectrum (D) corresponds to the purified **Cyclo(Se−Se)1**. Conditions: (5 mM) **Cyclo(Se−Se)1**, (5 mM) **BBSe$_2$**, methanol and LED lamp 400–700 nm. Red, green, orange, violet and dark-blue stars indicate peaks of **C1T**, dimer, **CM1C**, **CM1B** and **CM1A**, respectively.*

### 2.5.1.1. CM1A

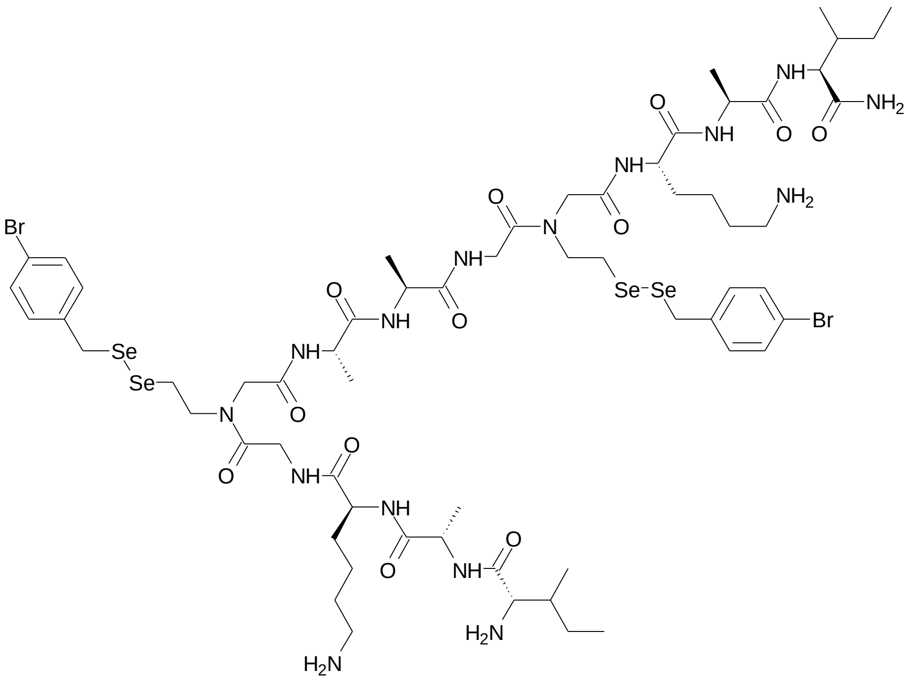

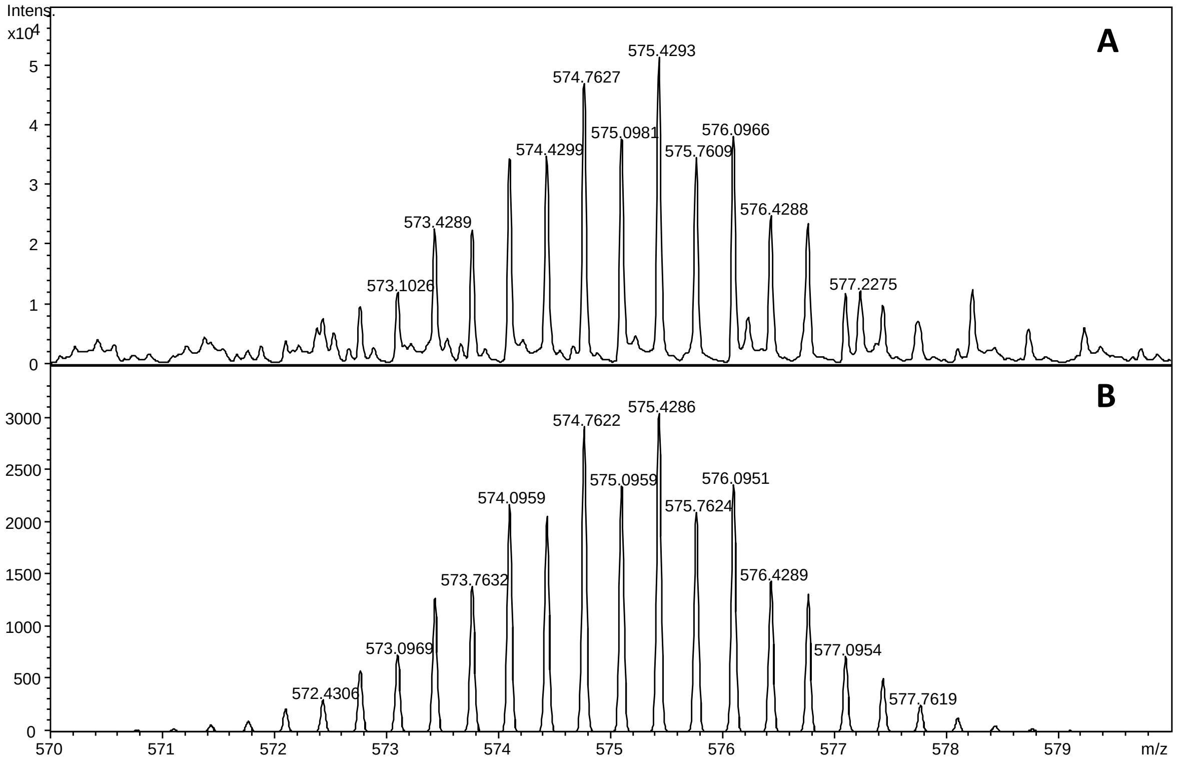


*Figure S61. ESI-qTOF-MS spectrum of **CM1A** ($C_{62}H_{99}Br_2N_{15}O_{12}Se_4$) (A) Isotopic distribution of the relative peak, m/z found $[M+3H]^{3+}$: 575.4293 (B) Isotopic distribution of the simulated peak, m/z calculated $[M+3H]^{3+}$: 575.4286*

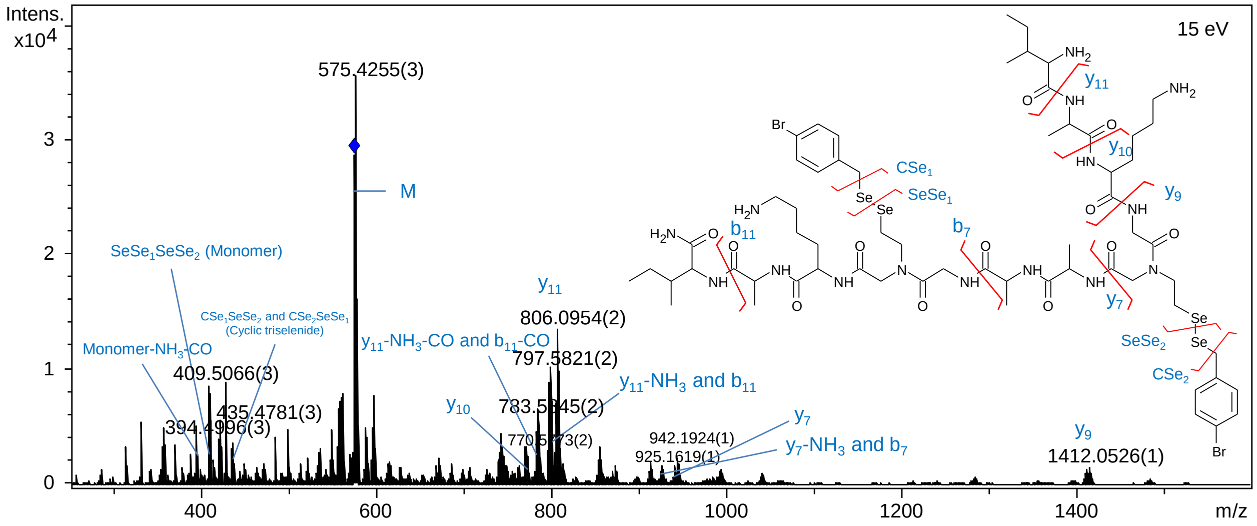


*Figure S62. ESI-qTOF-MS/MS (CE 15 eV) spectrum of **CM1A** ($C_{62}H_{99}Br_2N_{15}O_{12}Se_4$). Precursor ion: m/z $[M+3H]^{3+}$: 575.4255 (calculated for M: 575.4286, z=3+) The notations "CSe and SeSe" were used to indicate cleavage of C−Se and Se−Se bonds, respectively.*

## 2.5.1.2. C1T

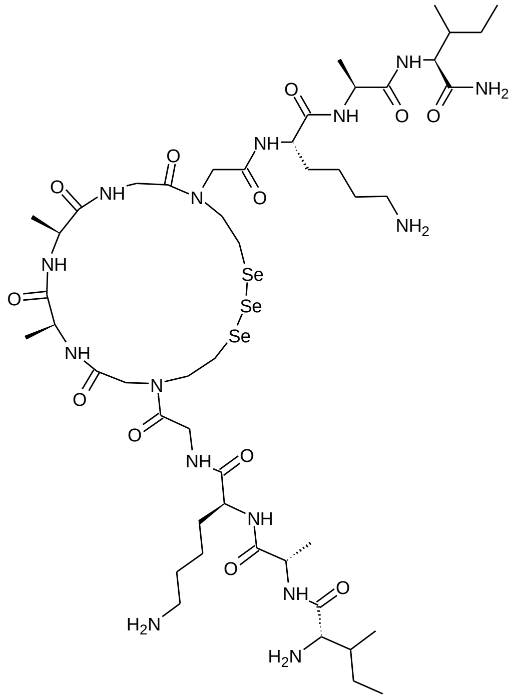

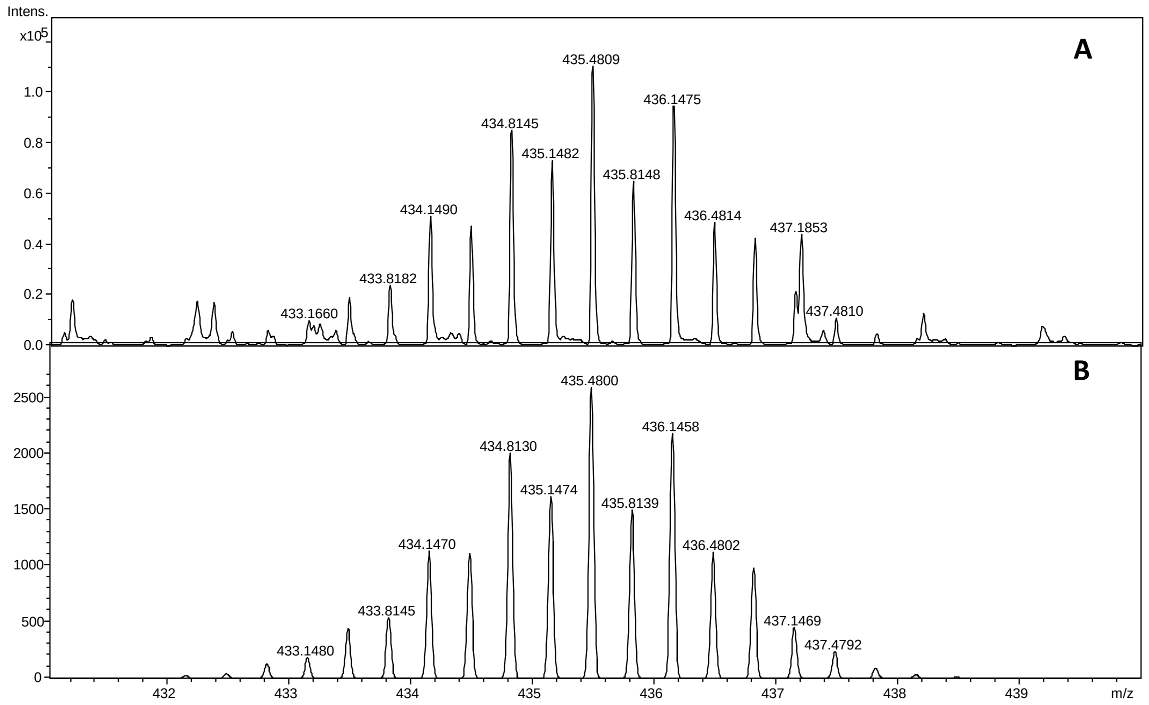


*Figure S63. ESI-qTOF-MS spectrum of **C1T** ($C_{48}H_{87}N_{15}O_{12}Se_3$) (A) Isotopic distribution of the relative peak, m/z found $[M+3H]^{3+}$: 436.1475 (B) Isotopic distribution of the simulated peak, m/z calculated $[M+3H]^{3+}$: 436.1458*

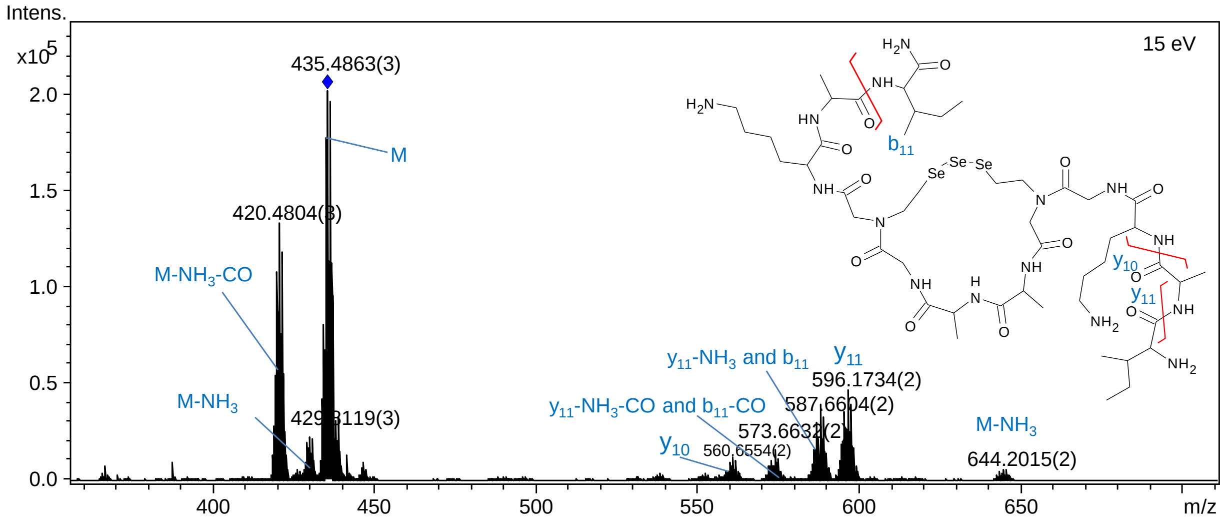


*Figure S64. ESI-qTOF-MS/MS (CE 15 eV) spectrum of **C1T** ($C_{48}H_{87}N_{15}O_{12}Se_3$). Precursor ion: m/z $[M+3H]^{3+}$: 435.4863 (calculated for M: 435.4800, z=3+)*

### *2.5.1.3. Dimer form of Cyclo(Se−Se)1*

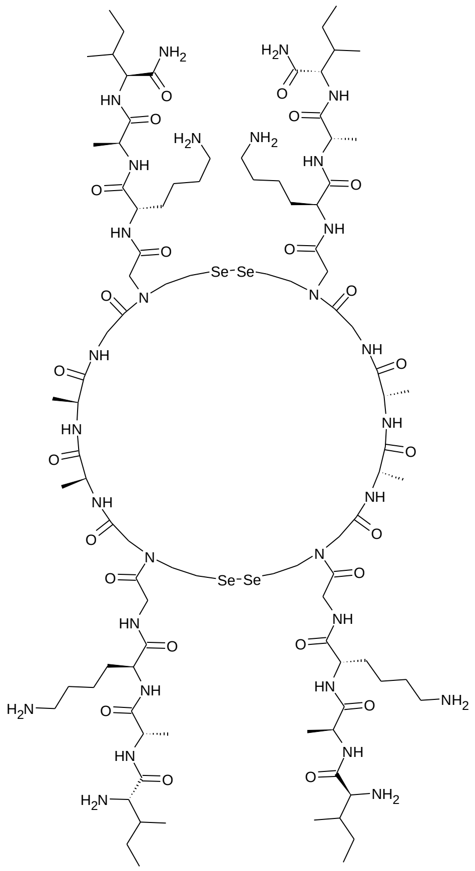

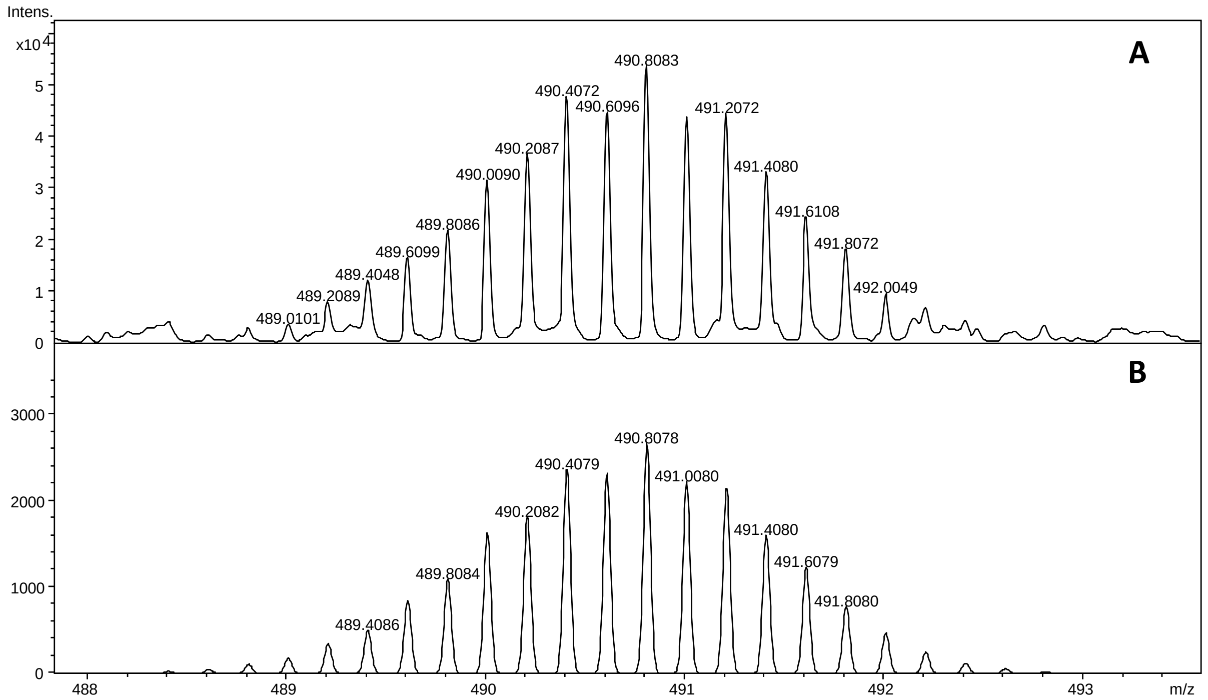


*Figure S65. ESI-qTOF-MS spectrum of the dimer ($C_{96}H_{174}N_{30}O_{24}Se_4$) (A) Isotopic distribution of the relative peak, m/z found $[M+5H]^{5+}$: 491.2072 (B) Isotopic distribution of the simulated peak, m/z calculated $[M+5H]^{5+}$: 491.2079*

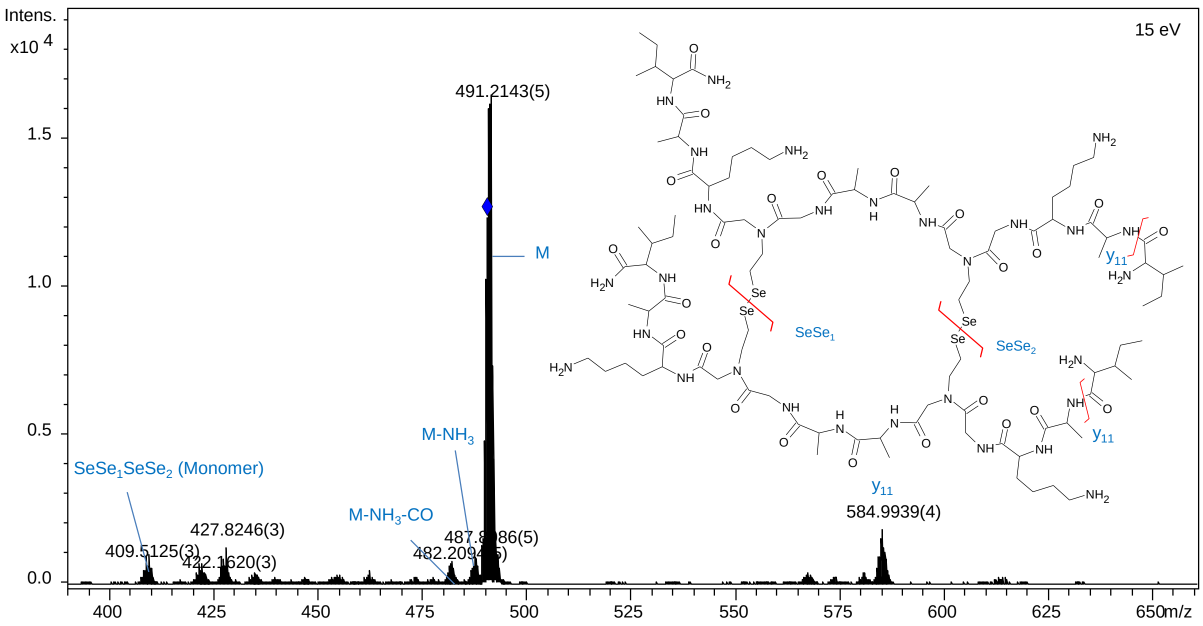


*Figure S66. ESI-qTOF-MS/MS (CE 15 eV) spectrum of the dimer ($C_{96}H_{174}N_{30}O_{24}Se_4$). Precursor ion: m/z $[M+5H]^{5+}$: 491.2143 (calculated for M: 491.2079, z=5+) The notation "SeSe" was used to indicate the cleavage of Se−Se bond.*

## 2.5.1.4. CM1B

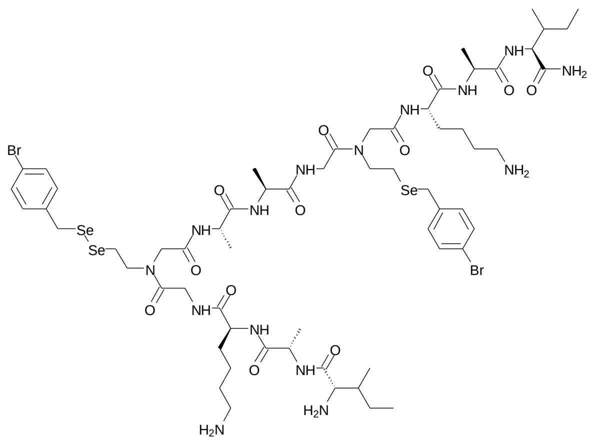

**and/or**

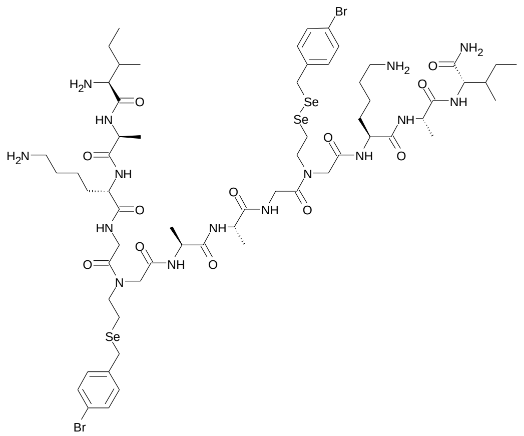

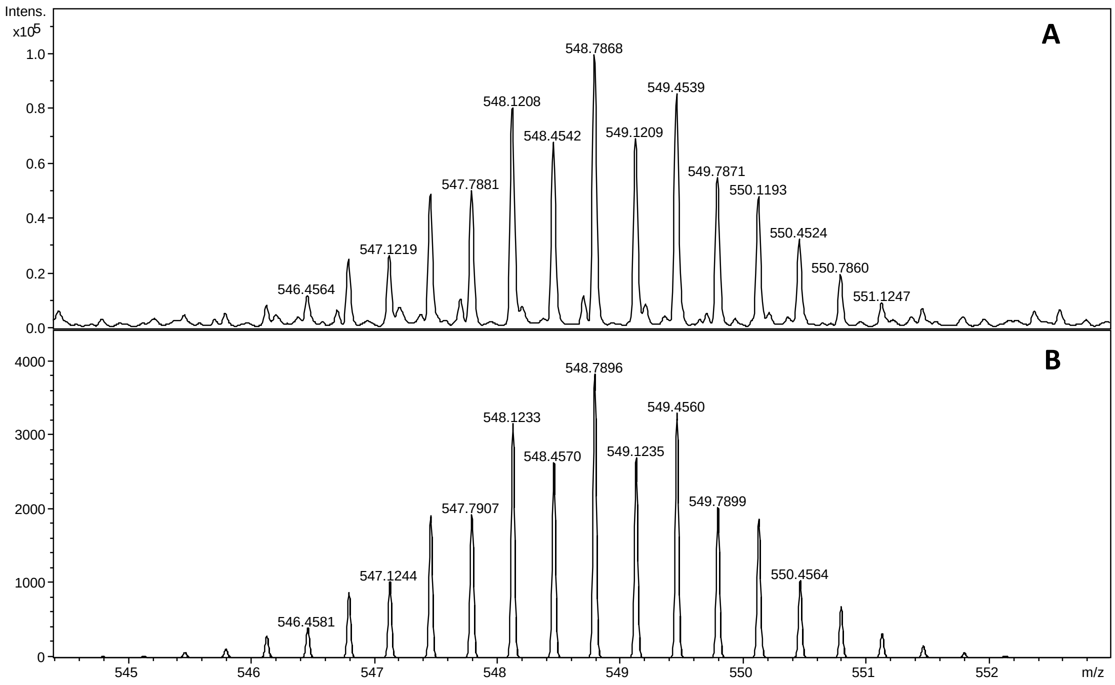


*Figure S67. ESI-qTOF-MS spectrum of **CM1B** ($C_{62}H_{99}Br_2N_{15}O_{12}Se_3$) (A) Isotopic distribution of the relative peak, m/z found $[M+3H]^{3+}$: 548.7868 (B) Isotopic distribution of the simulated peak, m/z calculated $[M+3H]^{3+}$: 548.7896*

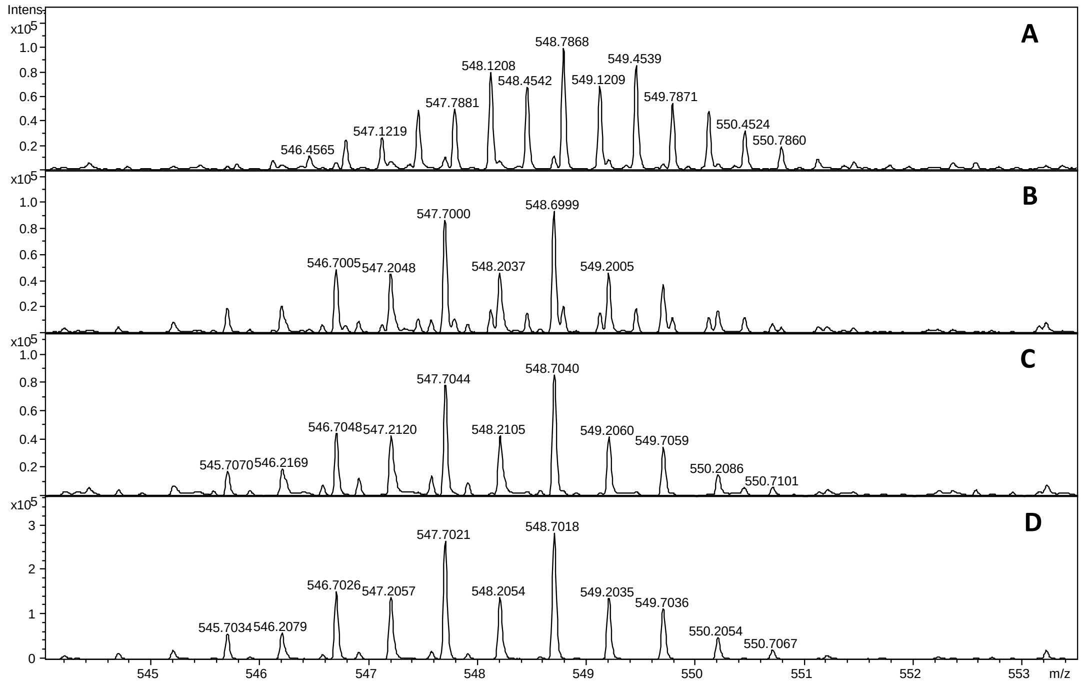


*Figure S68. ESI-qTOF-MS spectra illustrating the isotopic distribution of peaks corresponding to **CM1B** (A) and fragment of **Cyclo(Se−Se)1** "$y_{11}$-$NH_3$ and $b_{11}$" (B, C, D). Spectra A, B and C were obtained after 48 h, 24 h and 1 h of irradiation of the sample containing **Cyclo(Se−Se)1** and **BBSe₂**. Spectrum D corresponds to the purified **Cyclo(Se−Se)1**. This figure was included to show the changes in isotopic distribution.*

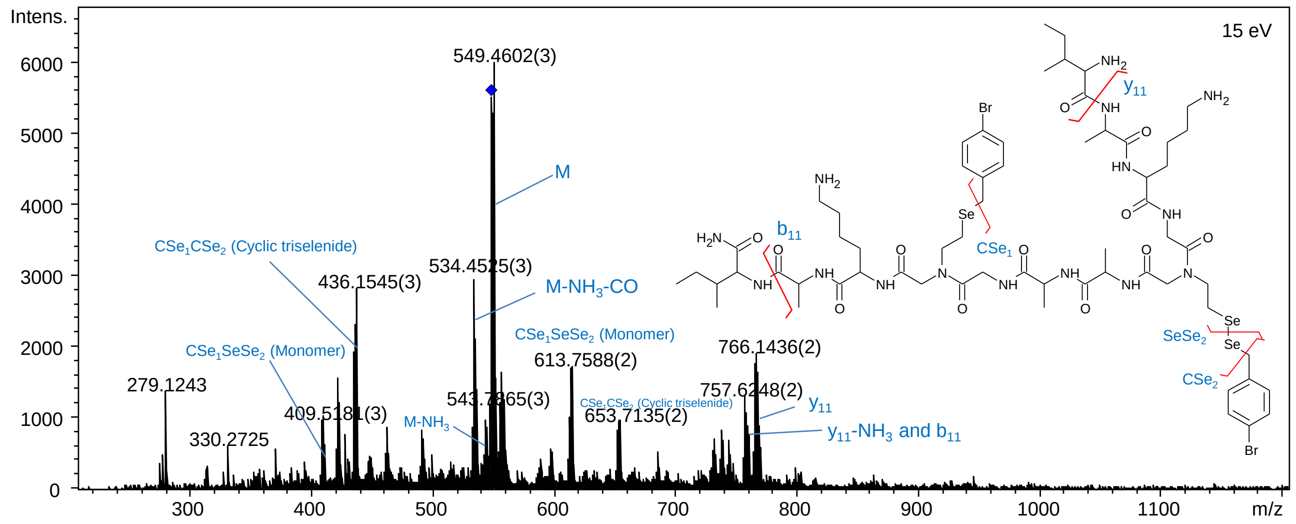


*Figure S69. ESI-qTOF-MS/MS (CE 15 eV) spectrum of **CM1B** ($C_{62}H_{99}Br_2N_{15}O_{12}Se_3$). Precursor ion: m/z $[M+3H]^{3+}$: 549.4602 (calculated for M: 549.4560, z=3+) The notations "CSe and SeSe" were used to indicate cleavage of C−Se and Se−Se bonds, respectively.*

### 2.5.1.5. CM1C

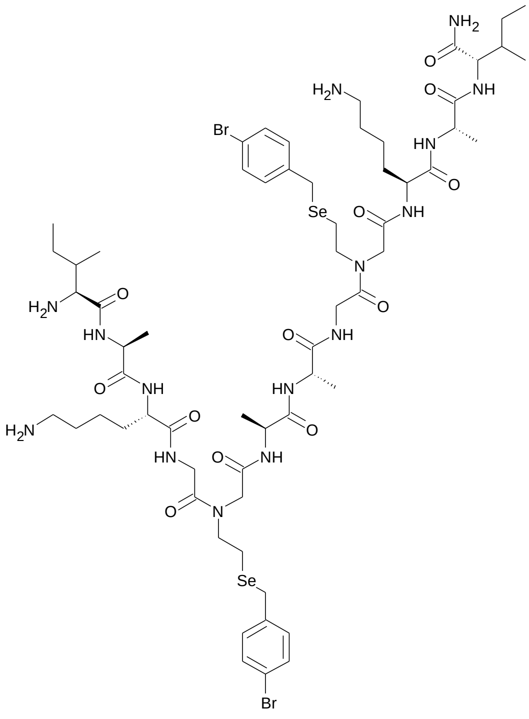

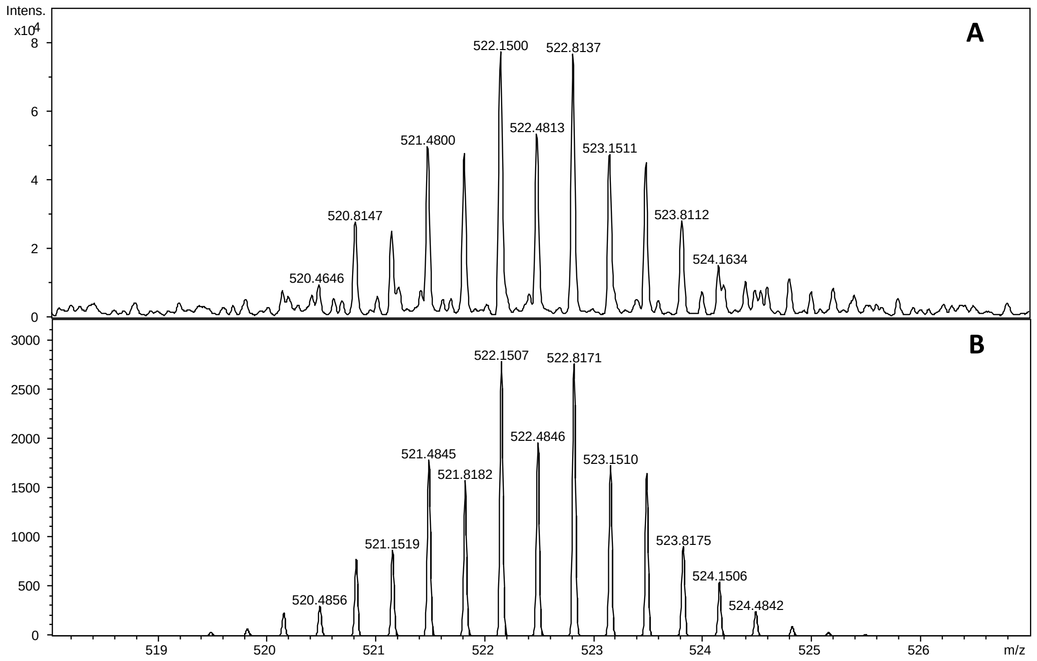


*Figure S70. ESI-qTOF-MS spectrum of* ***CM1C*** *($C_{62}H_{99}Br_2N_{15}O_{12}Se_2$) (A) Isotopic distribution of the relative peak, m/z found $[M+3H]^{3+}$: 522.1500 (B) Isotopic distribution of the simulated peak, m/z calculated $[M+3H]^{3+}$: 522.1507*

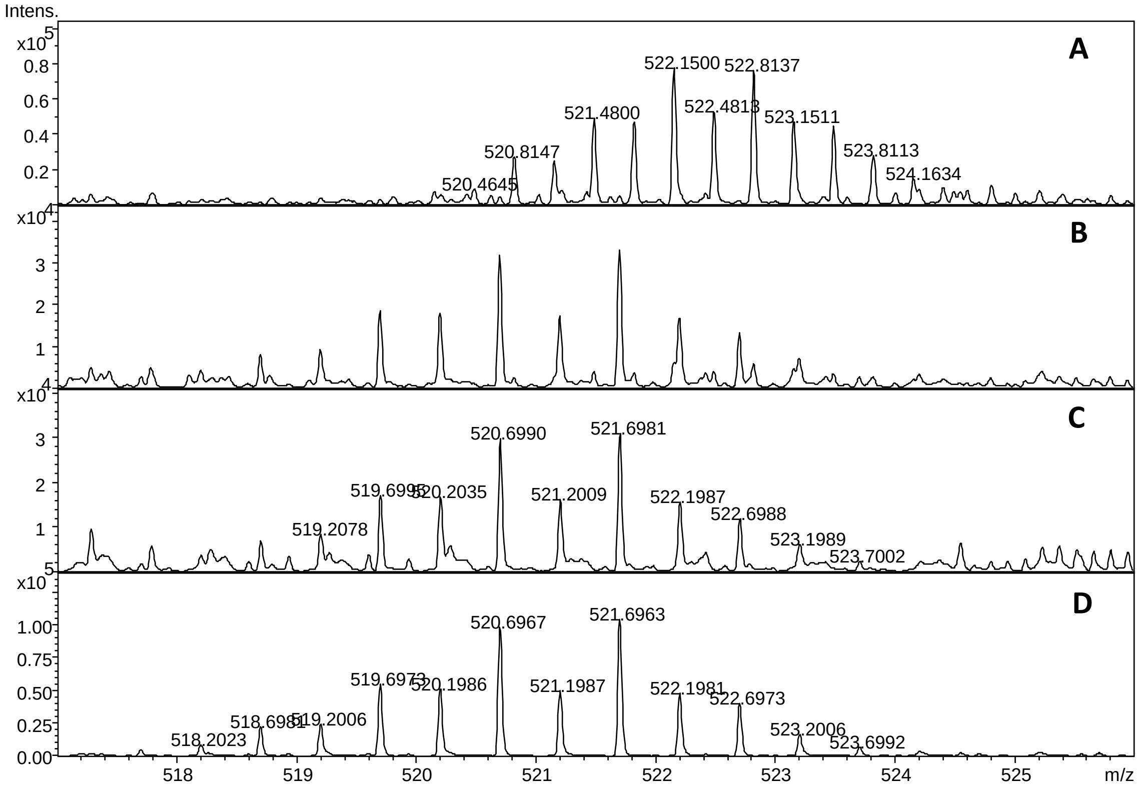


*Figure S71. ESI-qTOF-MS spectra illustrating the isotopic distribution of peaks corresponding to* ***CM1C*** *(A) and fragment of* ***Cyclo(Se−Se)1*** *"$y_{10}$" (B, C, D). Spectra A, B and C were obtained after 48 h, 24 h and 1 h of irradiation of the sample containing* ***Cyclo(Se−Se)1*** *and* ***BBSe₂***. *Spectrum D corresponds to the purified* ***Cyclo(Se−Se)1***. *This figure was included to show the changes in isotopic distribution.*

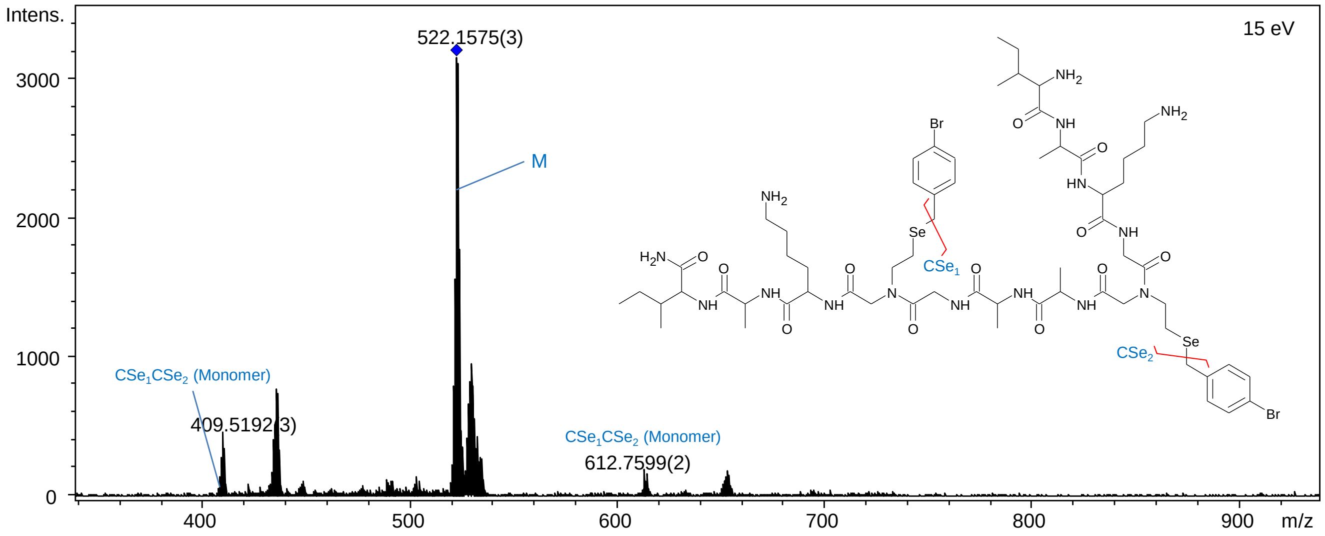


*Figure S72. ESI-qTOF-MS/MS (CE 15 eV) spectrum of **CM1C** ($C_{62}H_{99}Br_2N_{15}O_{12}Se_2$). Precursor ion: m/z $[M+3H]^{3+}$: 522.1575 (calculated for M: 522.1507, z=3+) The notation "CSe" was used to indicate the cleavage of C−Se bond.*

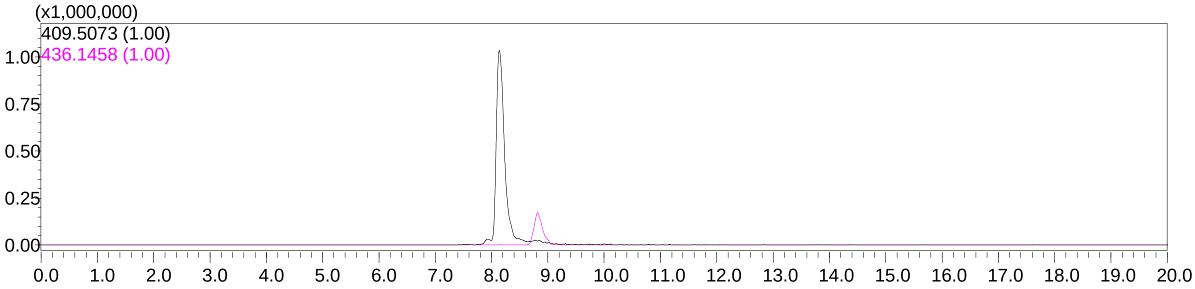


*Figure S73. LC-MS chromatogram, XIC of the **Cyclo(Se−Se)1** (black, retention time: 8.14 min) and XIC of **C1T** (pink, retention time: 8.83 min)*

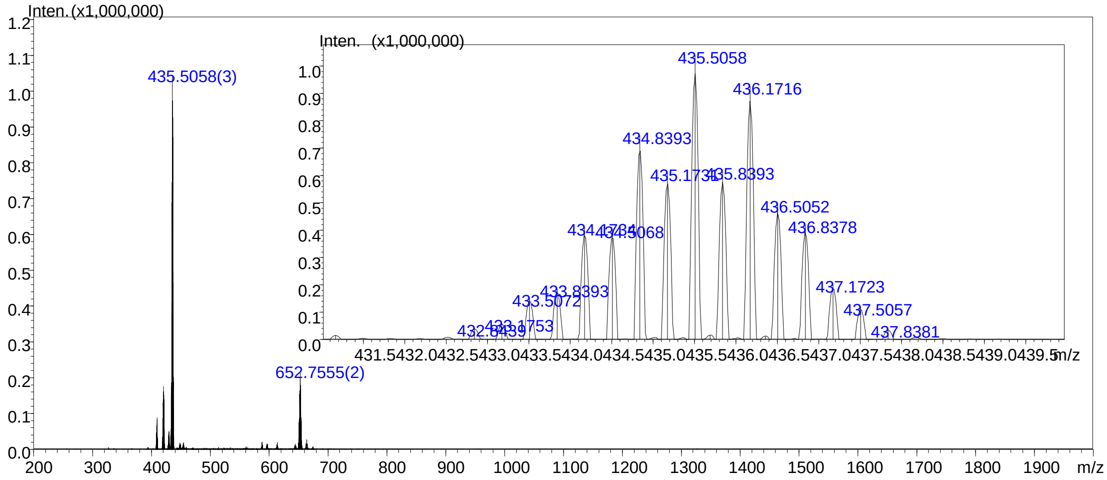


*Figure S74. LC-ESI-IT-TOF-MS spectrum of **C1T***

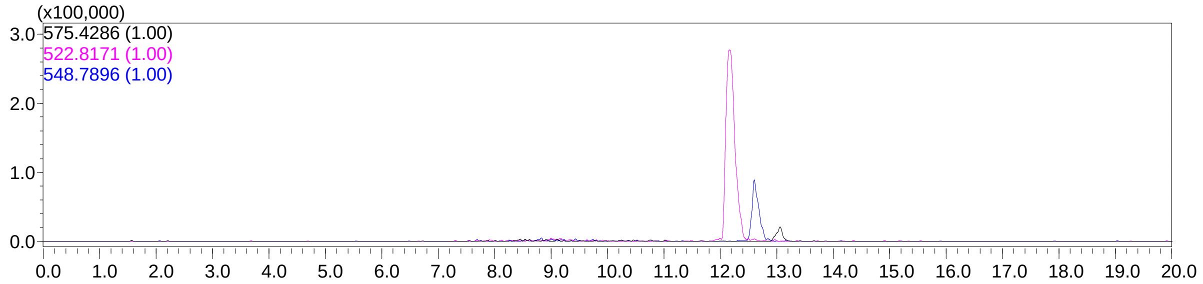


*Figure S75. LC-MS chromatogram, XIC of **CM1C** (pink, retention time: 12.16 min), XIC of **CM1B** (blue, retention time: 12.62 min) and XIC of **CM1A** (black, retention time: 13.07 min)*

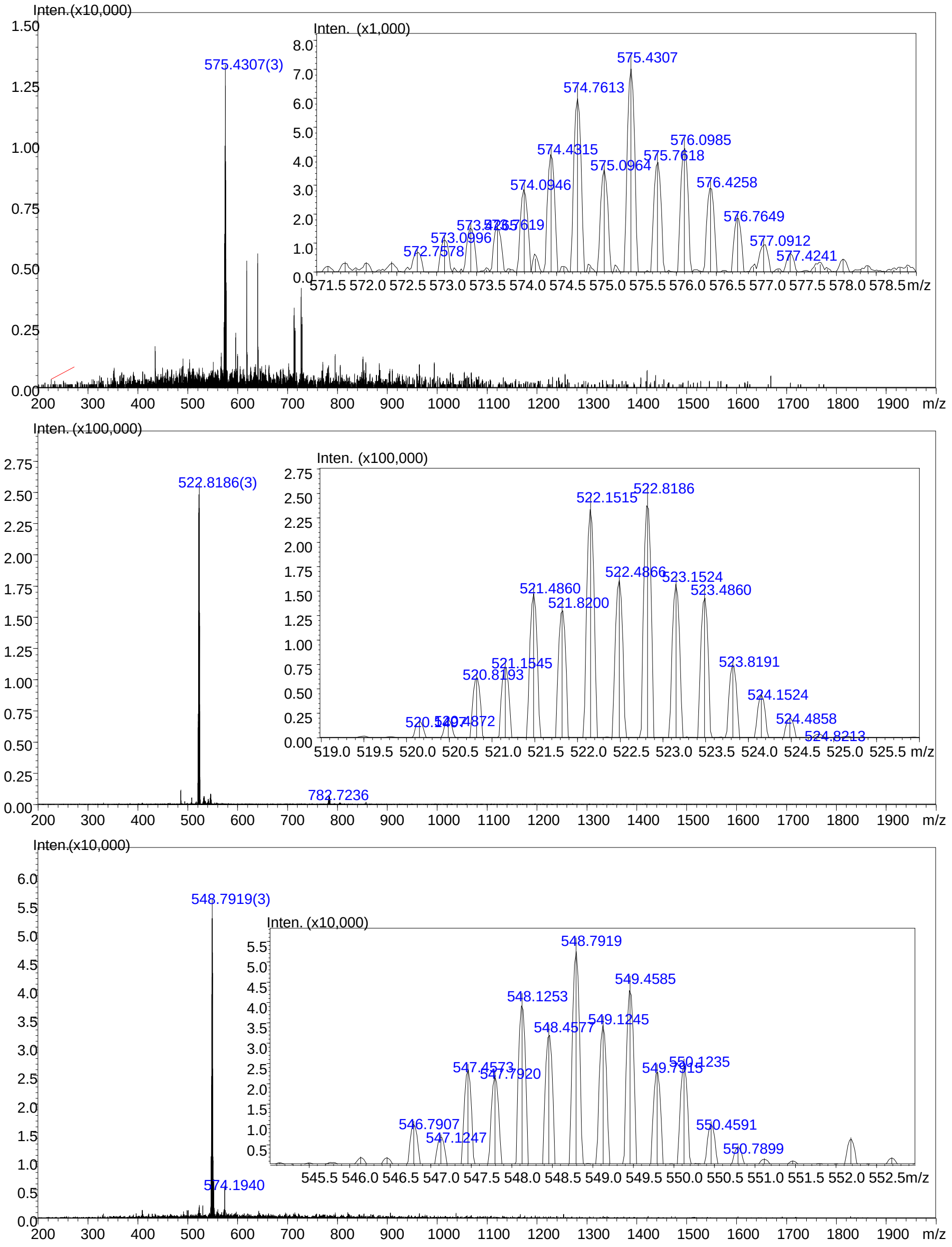


*Figure S76. LC-ESI-IT-TOF-MS spectra of **CM1A** (top), **CM1C** (middle) and **CM1B** (bottom)*

## 2.5.2. Metathesis reaction between Cyclo(Se−Se)1 and BBSe$_2$ under heat

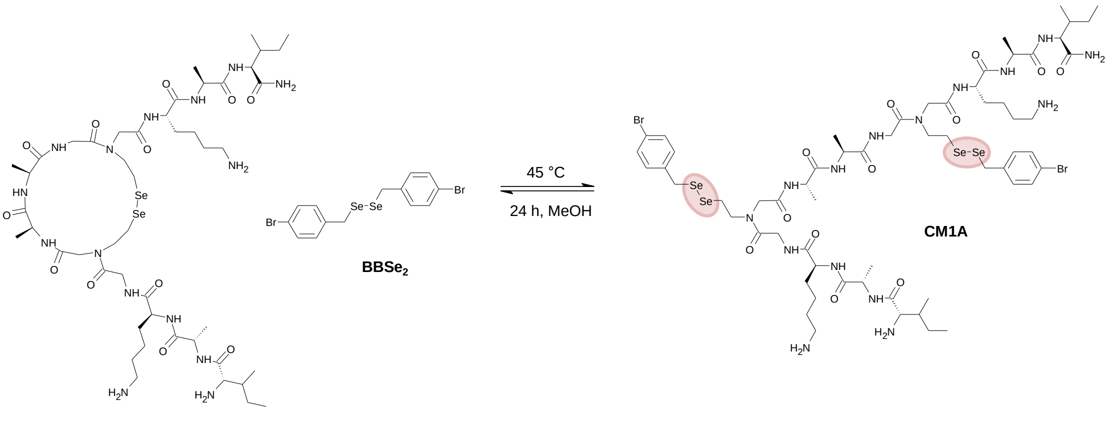


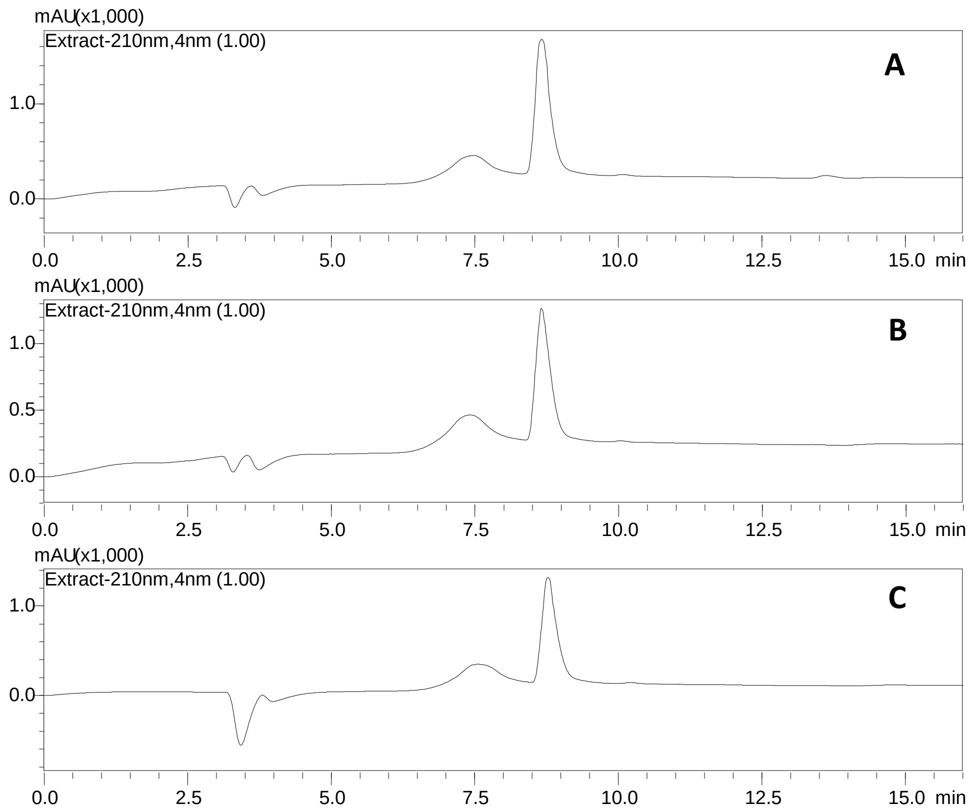


*Figure S77. HPLC chromatograms illustrating the progress of the metathesis reaction between **Cyclo(Se−Se)1** and **BBSe$_2$**. Chromatograms (B) and (A) were acquired after 2 h and 24 h of incubation of the sample in the dark at 45 °C, respectively. The chromatogram (C) corresponds to the purified **Cyclo(Se−Se)1**. Conditions: (5 mM) **Cyclo(Se−Se)1**, (5 mM) **BBSe$_2$**, methanol, dark and 45 °C.*

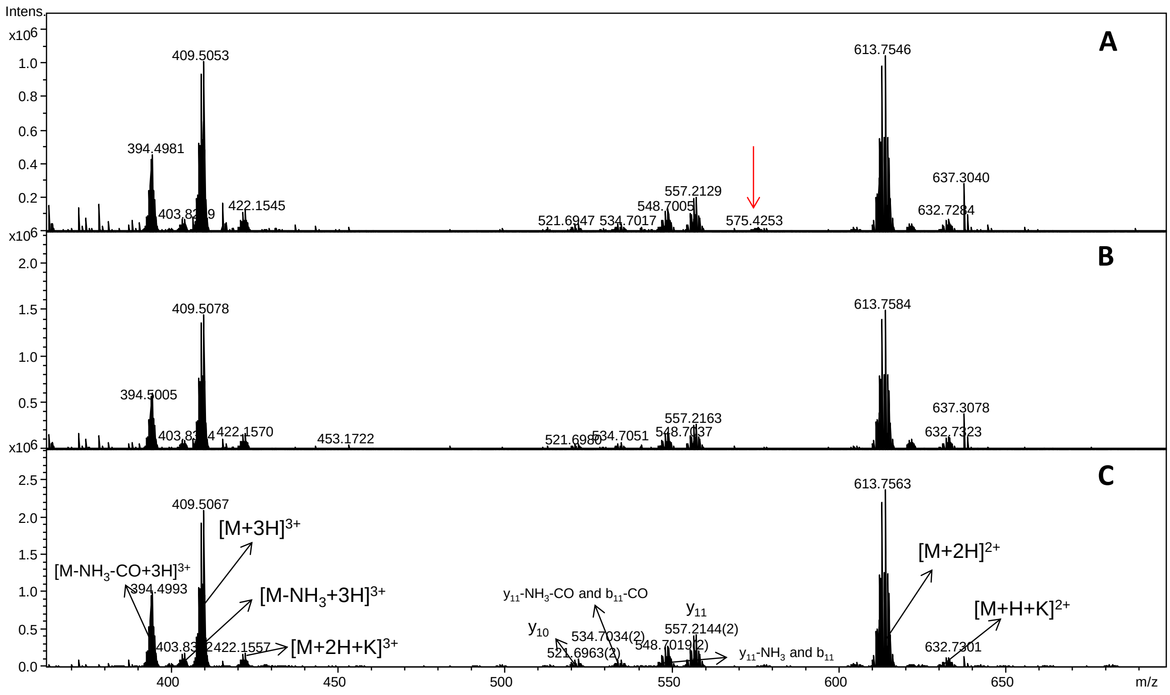


*Figure S78. ESI-qTOF-MS spectra illustrating the progress of the metathesis reaction between* ***Cyclo(Se−Se)1*** *and* ***BBSe₂****. Spectra (B) and (A) were acquired after 2 h and 24 h of incubation of the sample in the dark at 45 °C, respectively. The spectrum (C) corresponds to the purified* ***Cyclo(Se−Se)1****. Conditions: (5 mM)* ***Cyclo(Se−Se)1****, (5 mM)* ***BBSe₂****, methanol, dark and 45 °C.*

## 2.5.3. Metathesis reaction between Cyclo(Se−Se)1 and BBSe$_2$ in the presence of VA-044 under heat

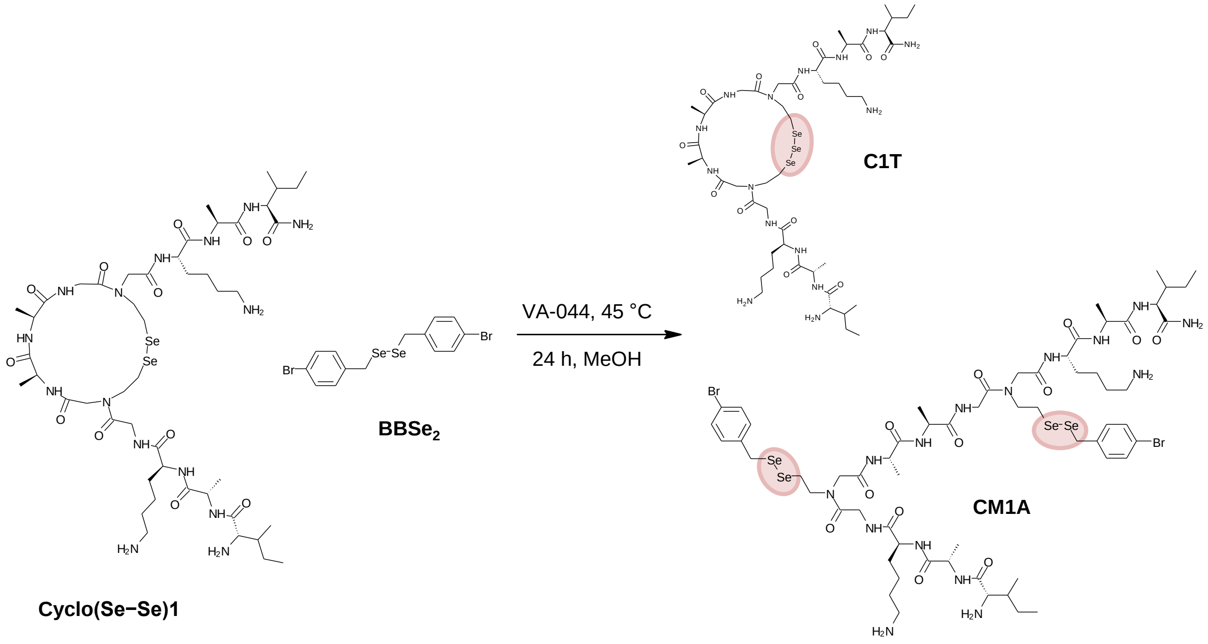

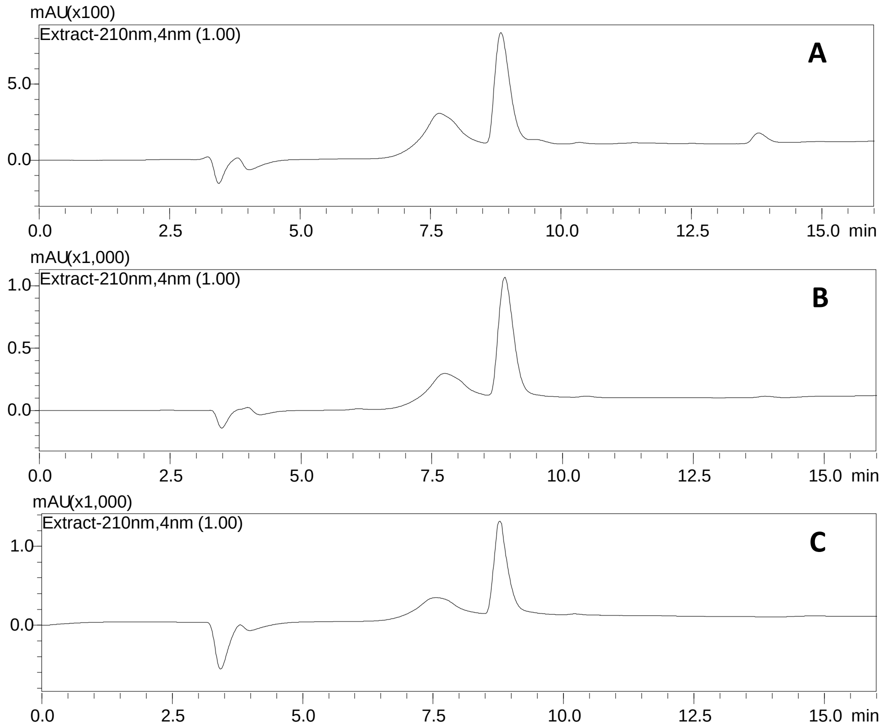


*Figure S79. HPLC chromatograms illustrating the progress of the metathesis reaction between **Cyclo(Se−Se)1** and **BBSe$_2$**. Chromatograms (B) and (A) were acquired after 2 h and 24 h of incubation of the sample in the dark at 45 °C, respectively. The chromatogram (C) corresponds to the purified **Cyclo(Se−Se)1**. Conditions: (5 mM) **Cyclo(Se−Se)1**, (5 mM) **BBSe$_2$**, (0.5 mM) VA-044, methanol, dark and 45 °C. Retention times (r.t.) of **Cyclo(Se−Se)1**, **C1T** and **CM1A** are 8.85 min, 9.60 min and 13.79 min, respectively.*

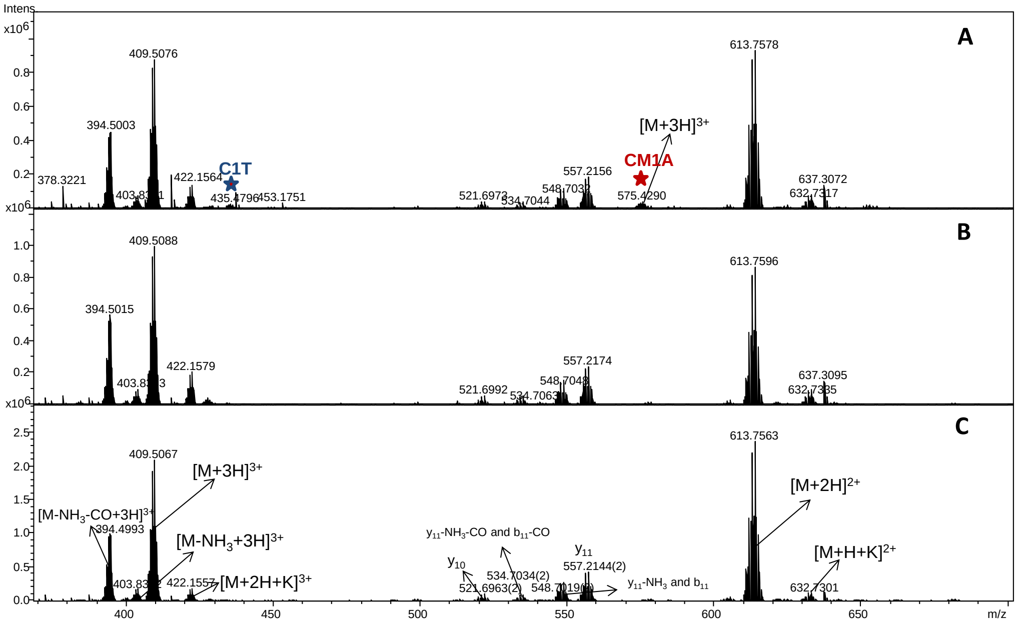


*Figure S80. ESI-qTOF-MS spectra illustrating the progress of the metathesis reaction between **Cyclo(Se−Se)1** and **BBSe$_2$**. Spectra (B) and (A) were acquired after 2 h and 24 h of incubation of the sample in the dark at 45 °C, respectively. The spectrum (C) corresponds to the purified **Cyclo(Se−Se)1**. Conditions: (5 mM) **Cyclo(Se−Se)1**, (5 mM) **BBSe$_2$**, (0.5 mM) VA-044, methanol, dark and 45 °C. The red and dark blue stars indicate the peaks of **CM1A** and **C1T**, respectively.*

## 2.6. Cyclo(Se−Se)2

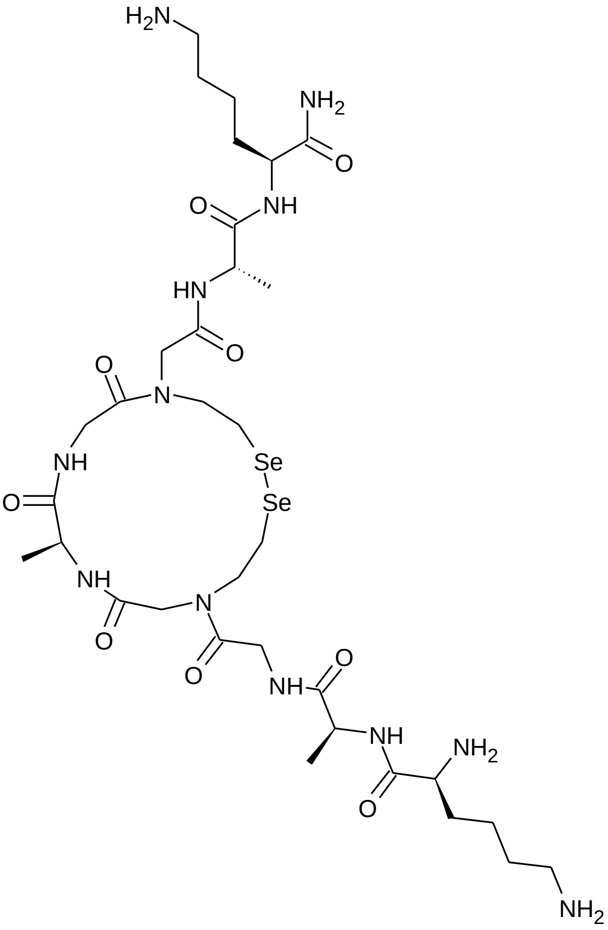


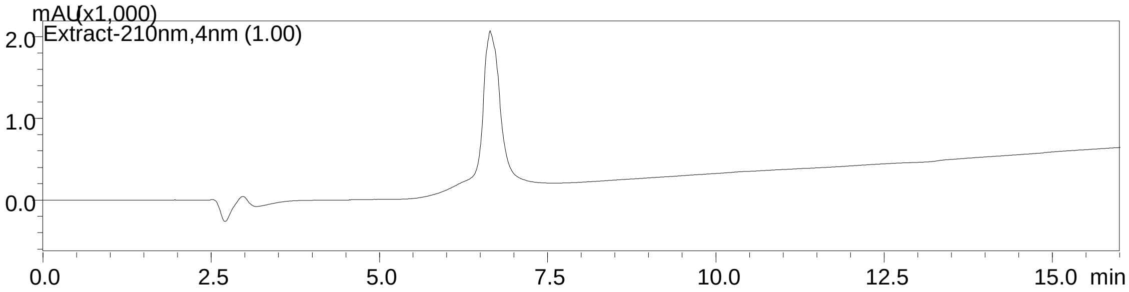


*Figure S81. HPLC chromatogram of **Cyclo(Se−Se)2** (retention time: 6.65 min)*

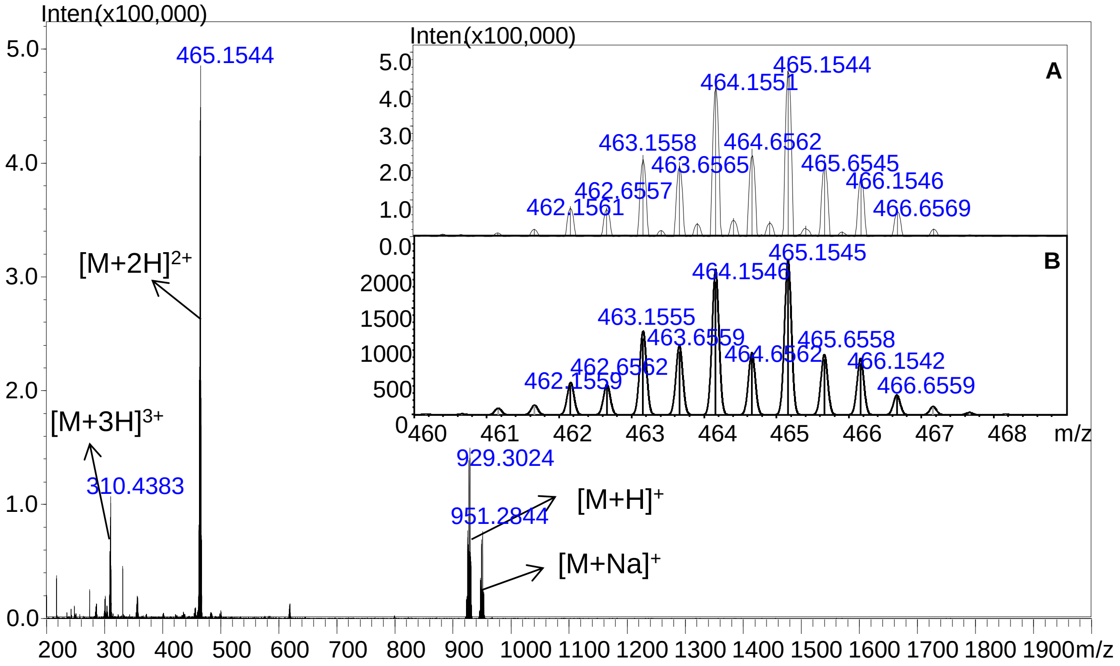


*Figure S82. LC-ESI-IT-TOF-MS spectrum of* ***Cyclo(Se−Se)2*** *($C_{33}H_{60}N_{12}O_9Se_2$) (A) Isotopic distribution of the relative peak, m/z found $[M+2H]^{2+}$: 465.1544 (B) Isotopic distribution of the simulated peak, m/z calculated $[M+2H]^{2+}$: 465.1545*

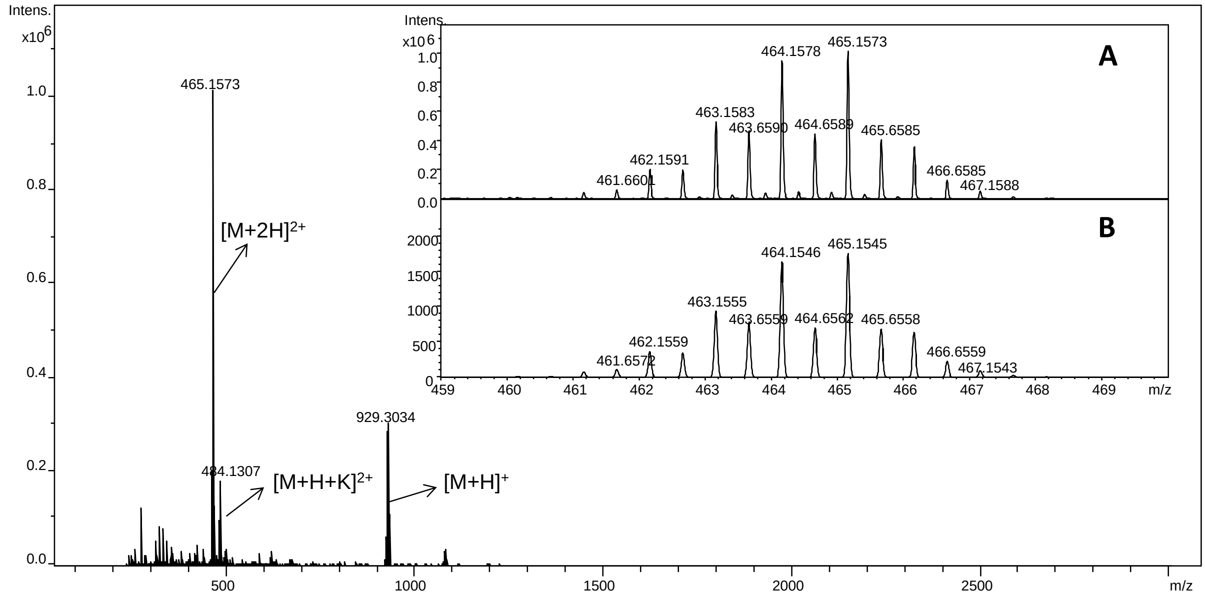


*Figure S83. ESI-qTOF-MS spectrum of* ***Cyclo(Se−Se)2*** *($C_{33}H_{60}N_{12}O_9Se_2$) (A) Isotopic distribution of the relative peak, m/z found $[M+2H]^{2+}$: 465.1573 (B) Isotopic distribution of the simulated peak, m/z calculated $[M+2H]^{2+}$: 465.1545*

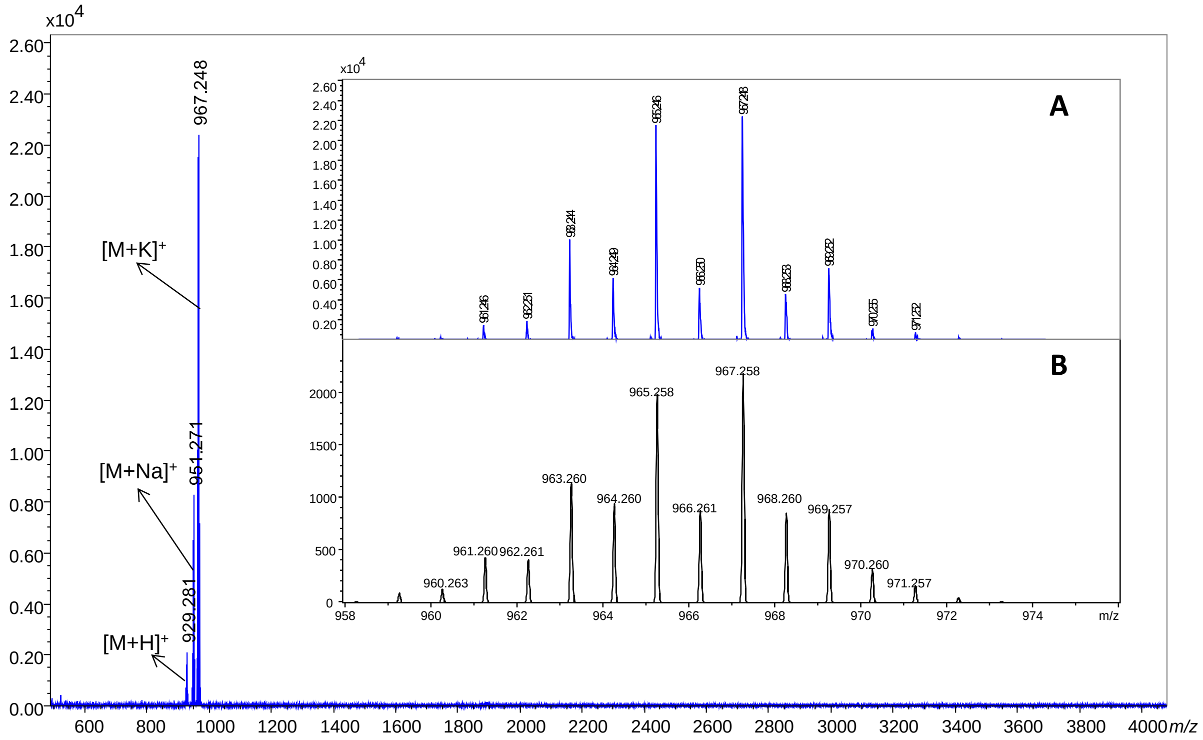


*Figure S84. MALDI-MS spectrum of **Cyclo(Se−Se)2** ($C_{33}H_{60}N_{12}O_9Se_2$) (A) Isotopic distribution of the relative peak, m/z found $[M+K]^+$: 967.248 (B) Isotopic distribution of the simulated peak, m/z calculated $[M+K]^+$: 967.258*

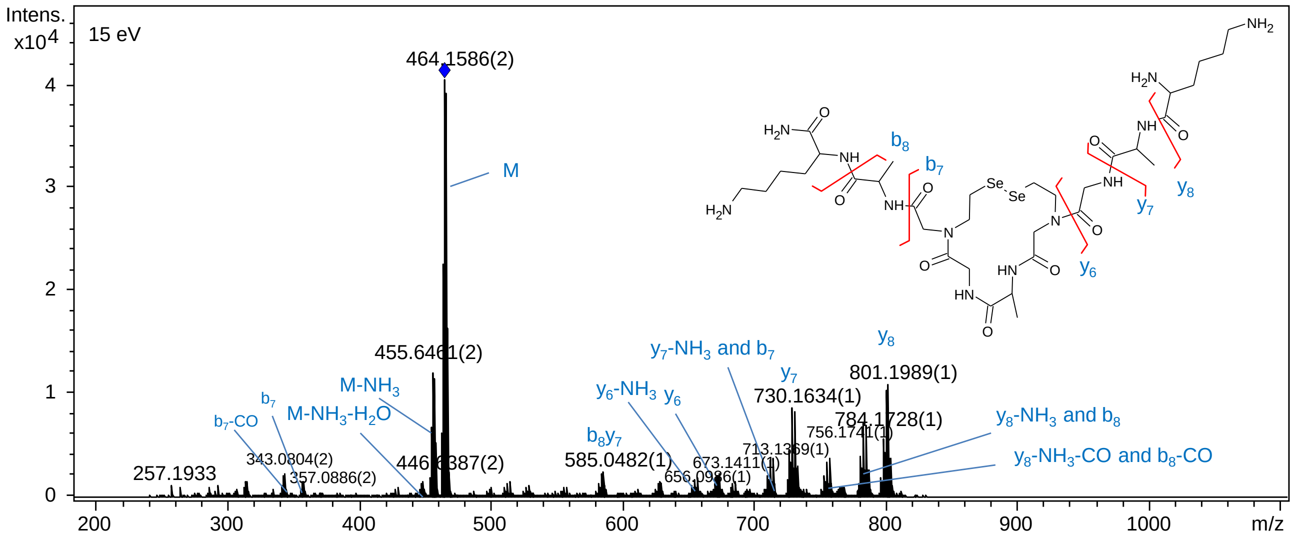


*Figure S85. ESI-qTOF-MS/MS (CE 15 eV) spectrum of **Cyclo(Se−Se)2** ($C_{33}H_{60}N_{12}O_9Se_2$). Precursor ion: m/z $[M+2H]^{2+}$: 464.1586 (calculated for M: 464.1546, z=2+)*

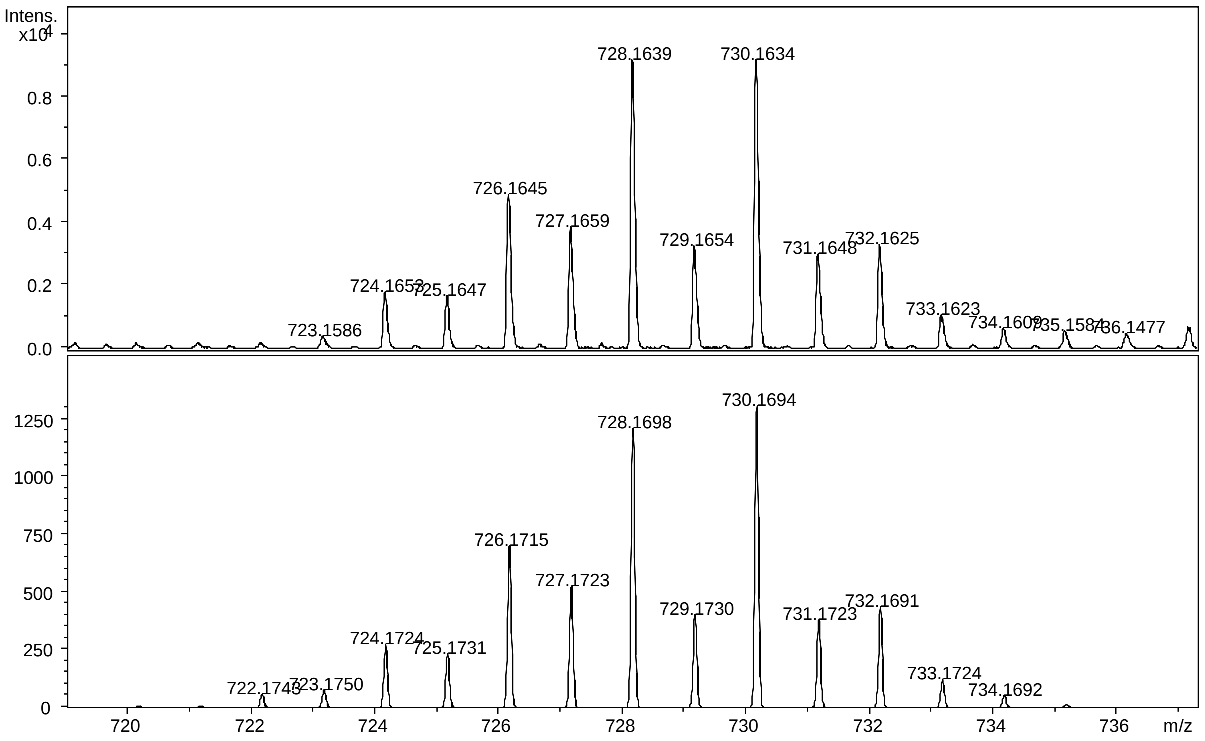


*Figure S86. ESI-qTOF-MS/MS (CE 15 eV) spectrum of* ***Cyclo(Se−Se)2*** *($C_{33}H_{60}N_{12}O_9Se_2$). Isotopic distribution of the relative peak that corresponds to the fragment $y_7$, m/z found $[M+H]^+$: 730.1634 (top) and isotopic distribution of the simulated peak, m/z calculated $[M+H]^+$: 730.1694 (bottom)*

## 2.6.1. Metathesis reaction between Cyclo(Se−Se)2 and BBSe$_2$ under visible light

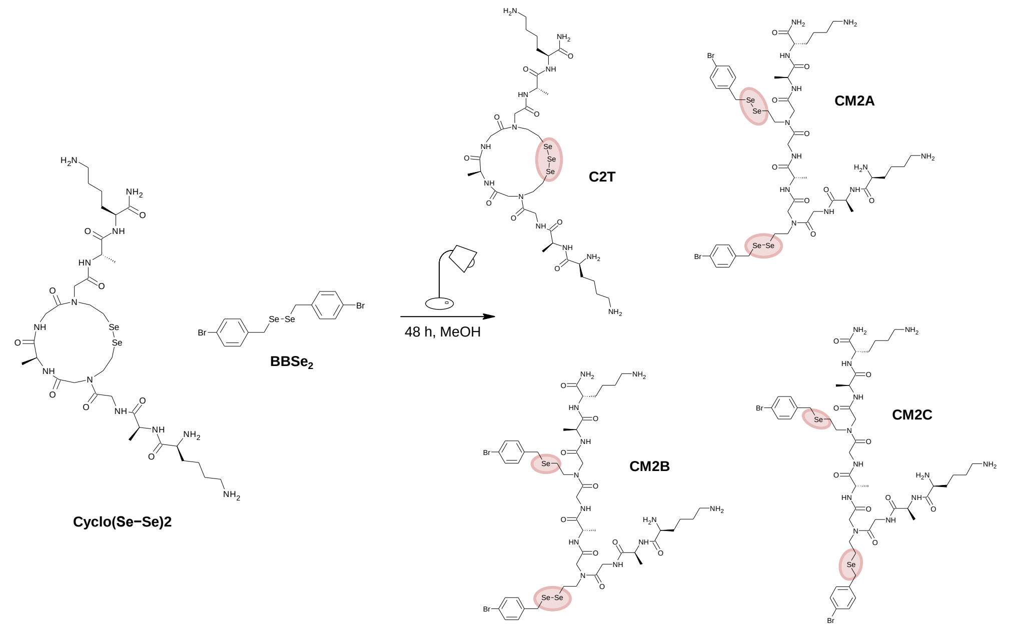

### 2.6.1.1. CM2A

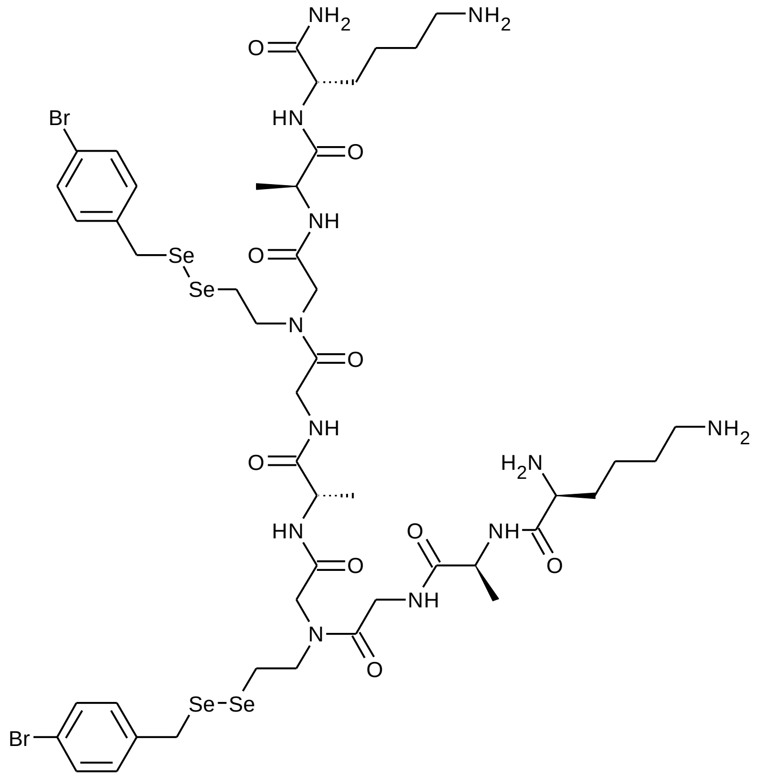


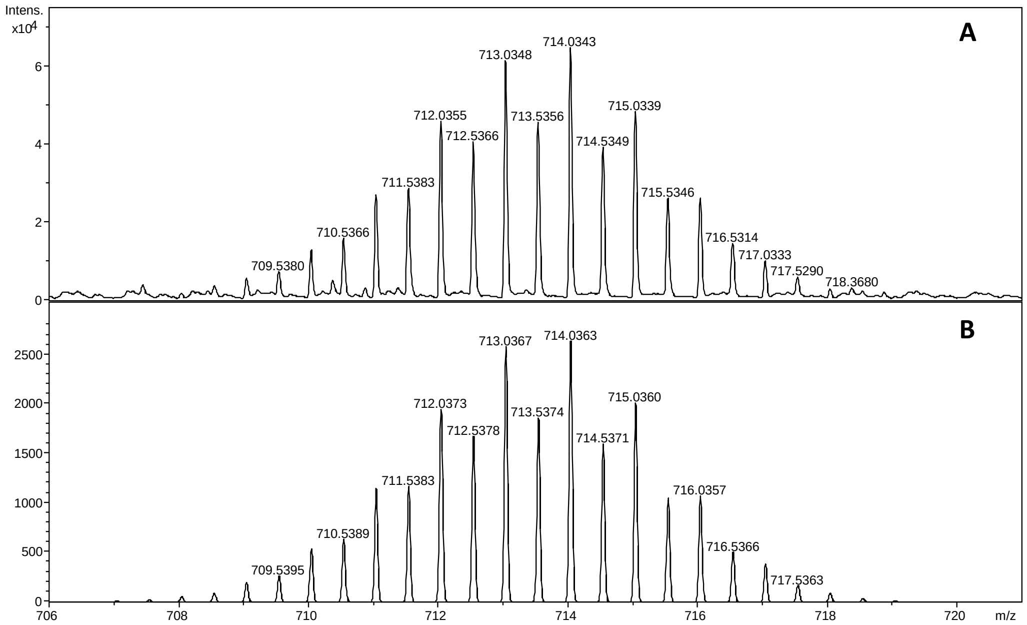


*Figure S87. ESI-qTOF-MS spectrum of **CM2A** ($C_{47}H_{72}Br_2N_{12}O_9Se_4$) (A) Isotopic distribution of the relative peak, m/z found $[M+2H]^{2+}$: 714.0343 (B) Isotopic distribution of the simulated peak, m/z calculated $[M+2H]^{2+}$: 714.0363*

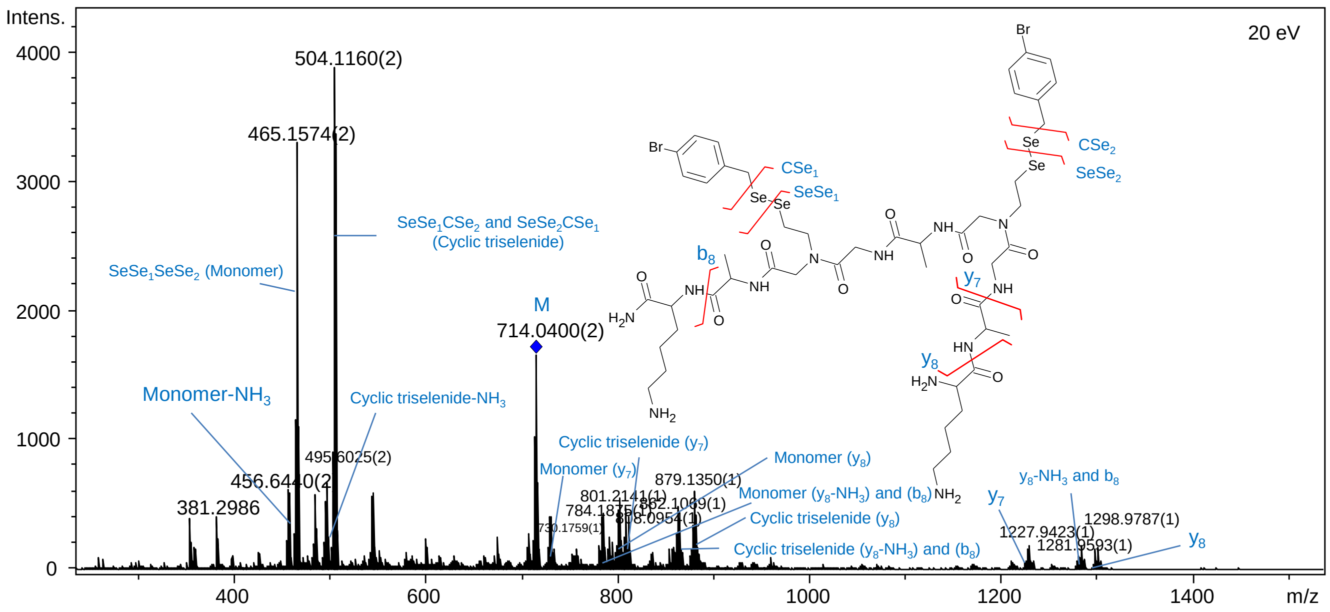


*Figure S88. ESI-qTOF-MS/MS (CE 20 eV) spectrum of **CM2A** ($C_{47}H_{72}Br_2N_{12}O_9Se_4$). Precursor ion: m/z $[M+2H]^{2+}$: 714.0400 (calculated for M: 714.0363, z=2+) The notations "CSe and SeSe" were used to indicate cleavage of C−Se and Se−Se bonds, respectively.*

### 2.6.1.2. CM2B

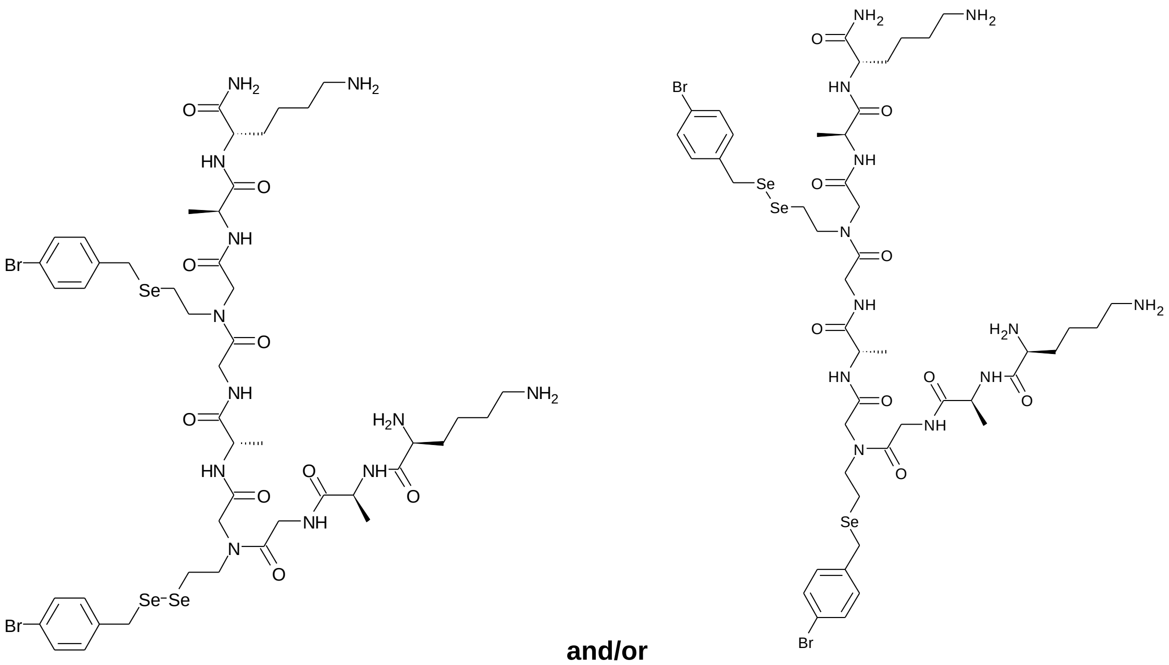

**and/or**

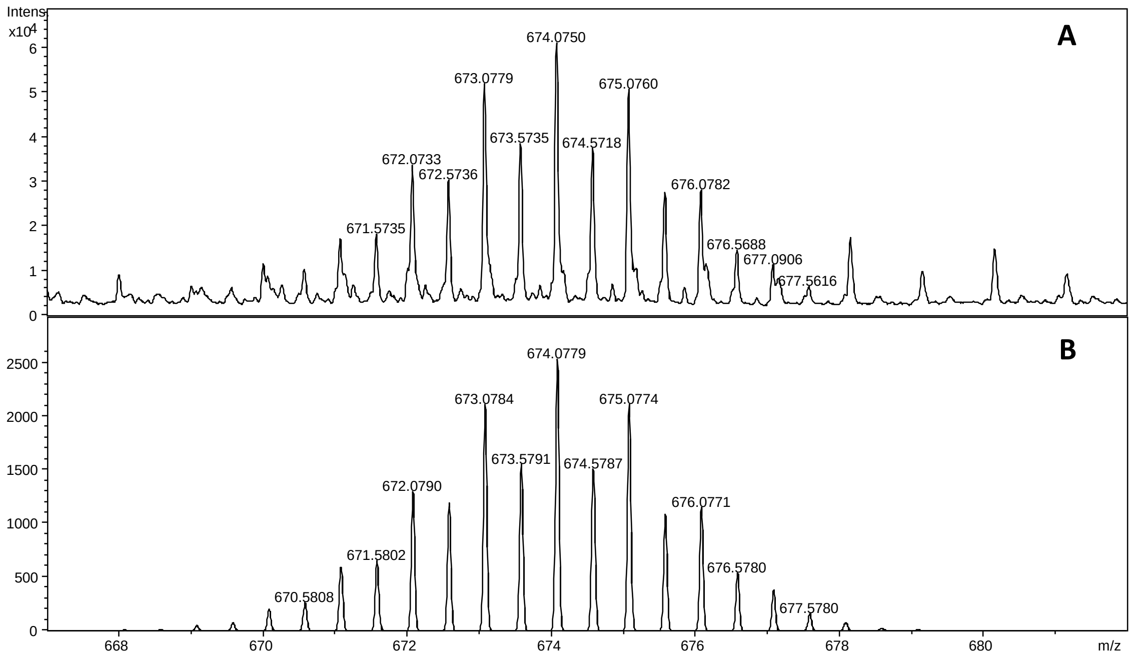


*Figure S89. ESI-qTOF-MS spectrum of **CM2B** ($C_{47}H_{72}Br_2N_{12}O_9Se_3$) (A) Isotopic distribution of the relative peak, m/z found $[M+2H]^{2+}$: 674.0750 (B) Isotopic distribution of the simulated peak, m/z calculated $[M+2H]^{2+}$: 674.0779*

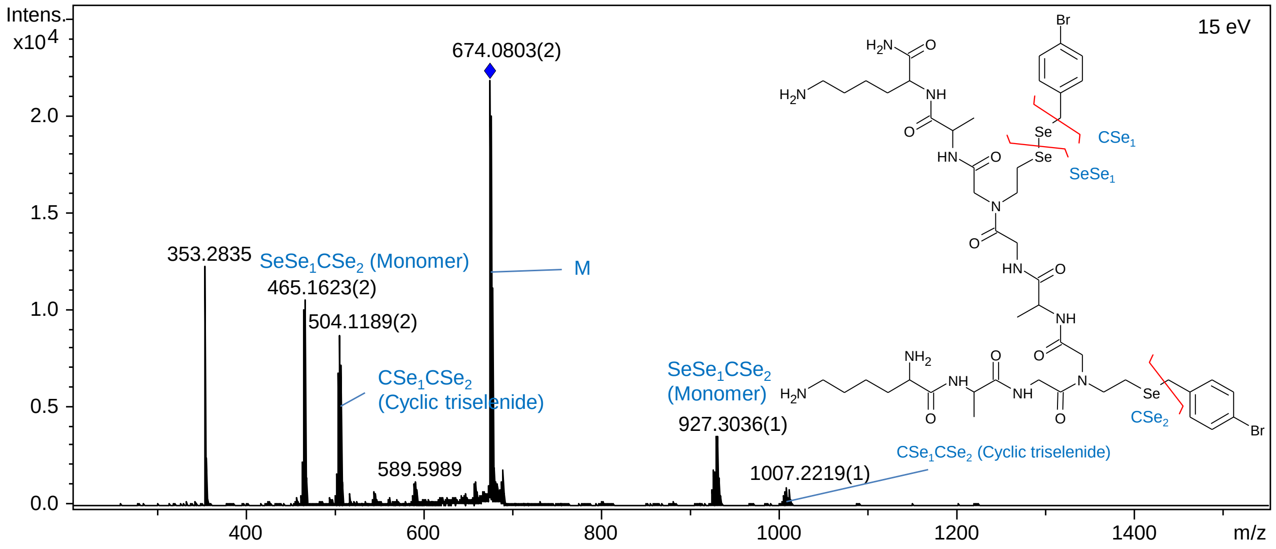


*Figure S90. ESI-qTOF-MS/MS (CE 15 eV) spectrum of **CM2B** ($C_{47}H_{72}Br_2N_{12}O_9Se_3$). Precursor ion: m/z $[M+2H]^{2+}$: 674.0803 (calculated for M: 674.0779, z=2+) The notations "CSe and SeSe" were used to indicate cleavage of C−Se and Se−Se bonds, respectively.*

### 2.6.1.3. CM2C

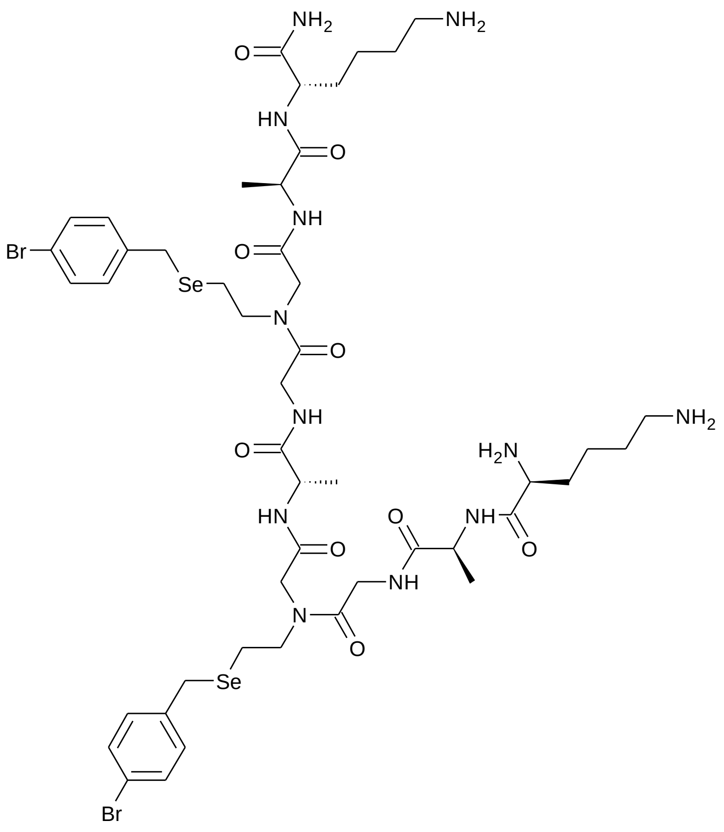


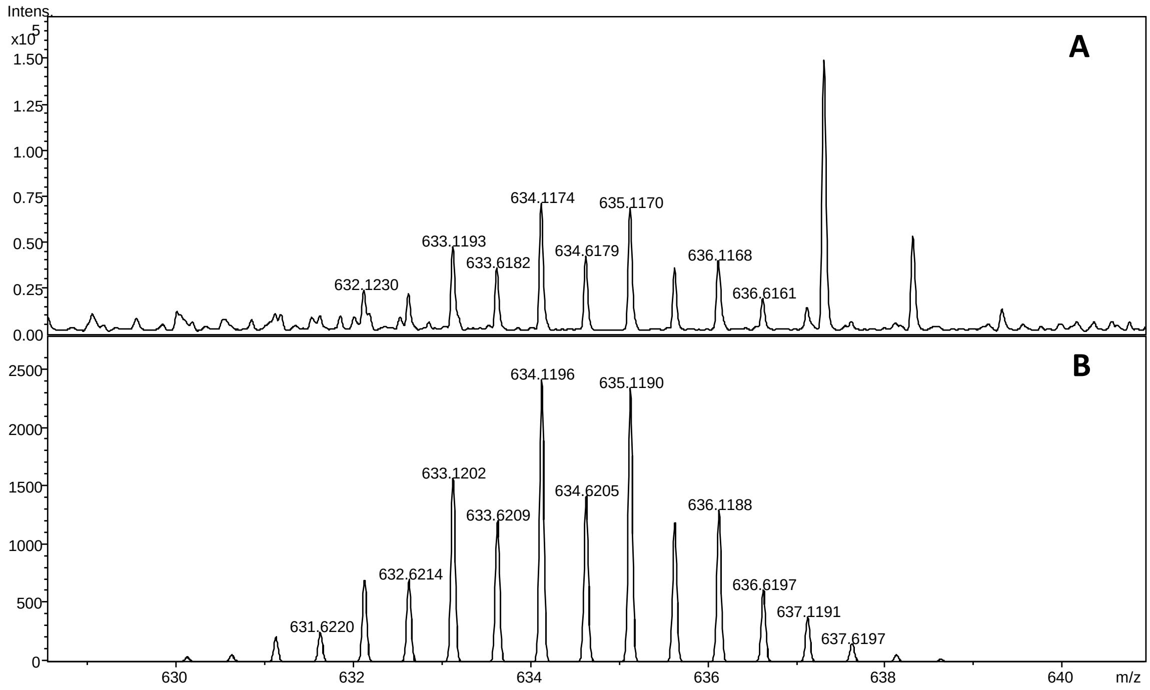


*Figure S91. ESI-qTOF-MS spectrum of **CM2C** ($C_{47}H_{72}Br_2N_{12}O_9Se_2$) (A) Isotopic distribution of the relative peak, m/z found $[M+2H]^{2+}$: 634.1174 (B) Isotopic distribution of the simulated peak, m/z calculated $[M+2H]^{2+}$: 634.1196*

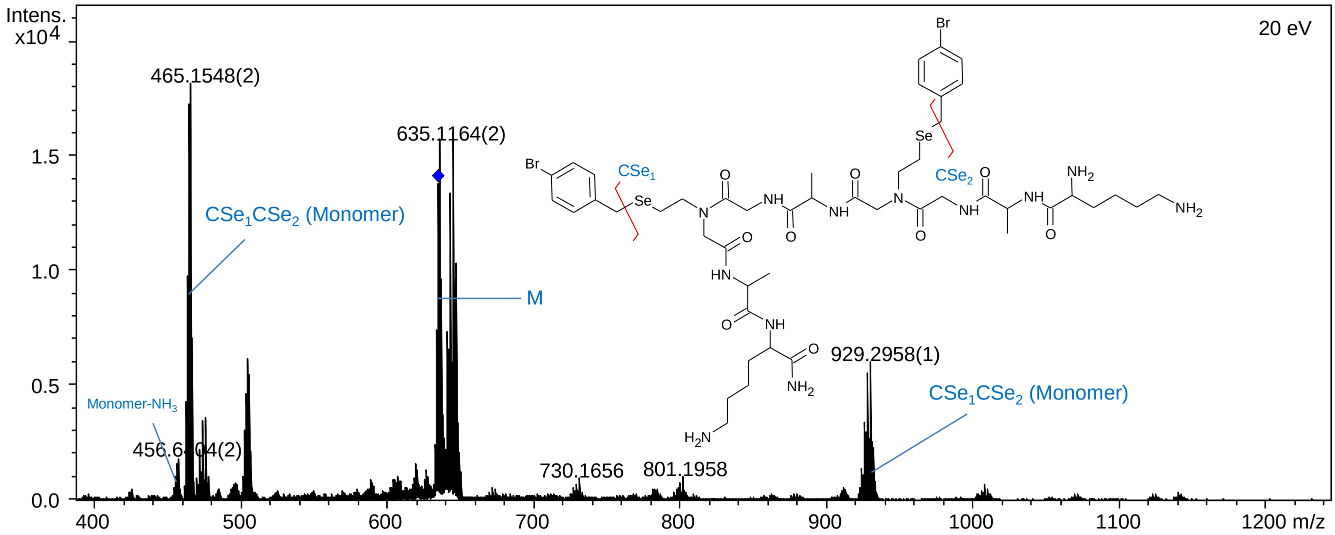


*Figure S92. ESI-qTOF-MS/MS (CE 20 eV) spectrum of* ***CM2C*** *($C_{47}H_{72}Br_2N_{12}O_9Se_2$). Precursor ion: m/z $[M+2H]^{2+}$: 635.1164 (calculated for M: 635.1190, z=2+) The notation "CSe" was used to indicate the cleavage of C−Se bond.*

### *2.6.1.4. C2T*

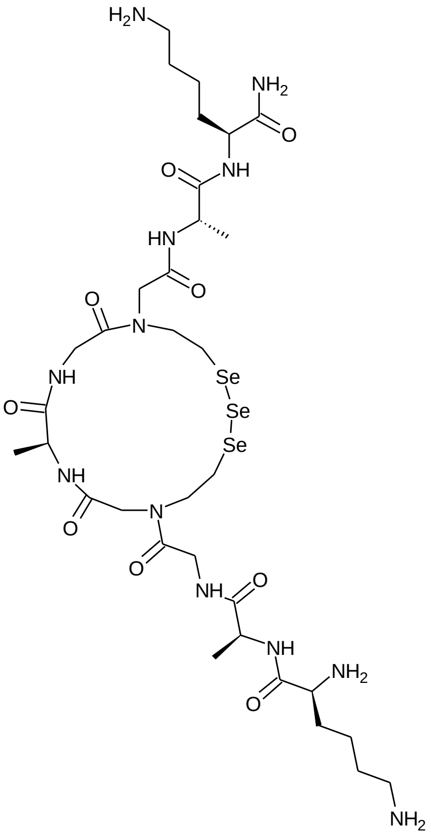

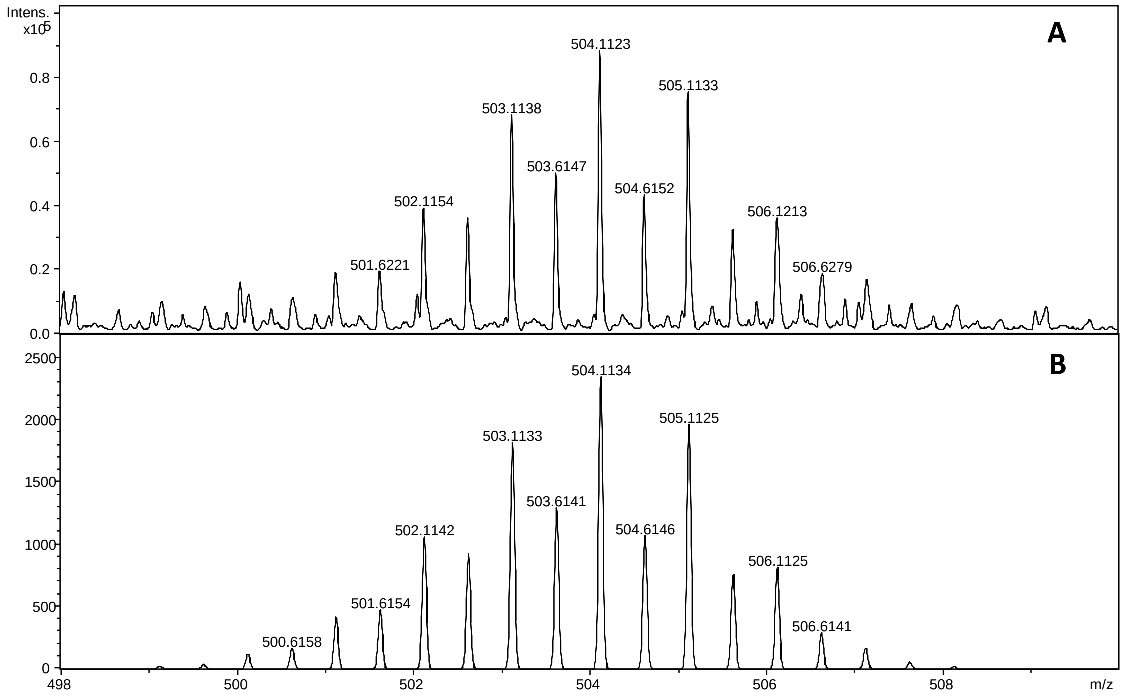


*Figure S93. ESI-qTOF-MS spectrum of **C2T** ($C_{33}H_{60}N_{12}O_9Se_3$) (A) Isotopic distribution of the relative peak, m/z found $[M+2H]^{2+}$: 505.1133 (B) Isotopic distribution of the simulated peak, m/z calculated $[M+2H]^{2+}$: 505.1125*

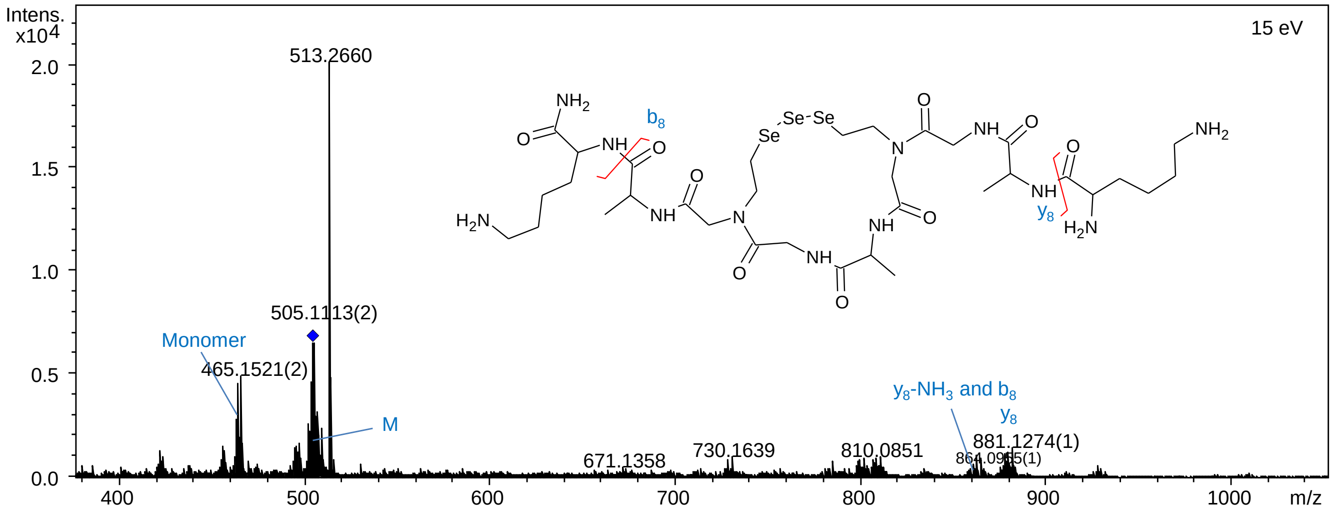


*Figure S94. ESI-qTOF-MS/MS (CE 15 eV) spectrum of **C2T** ($C_{33}H_{60}N_{12}O_9Se_3$). Precursor ion: m/z $[M+2H]^{2+}$: 505.1113 (calculated for M: 505.1125, z=2+)*

### 2.6.1.5. Dimer form of Cyclo(Se−Se)2

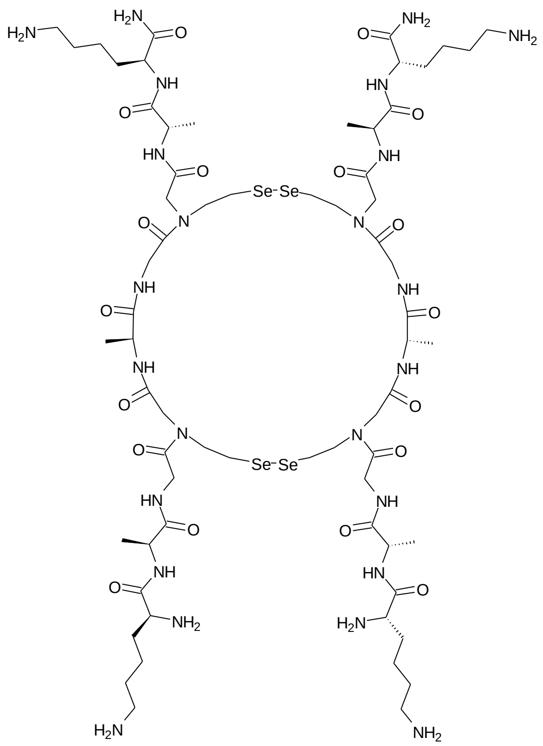

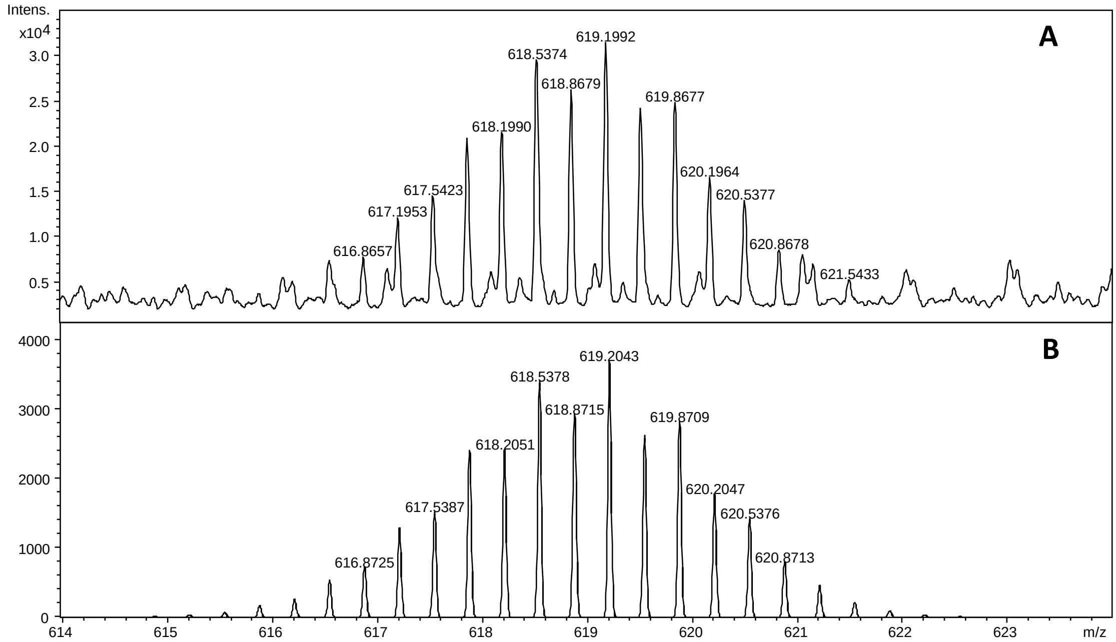


*Figure S95. ESI-qTOF-MS spectrum of the dimer ($C_{66}H_{120}N_{24}O_{18}Se_4$) (A) Isotopic distribution of the relative peak, m/z found $[M+3H]^{3+}$: 619.8677 (B) Isotopic distribution of the simulated peak, m/z calculated $[M+3H]^{3+}$: 619.8709*

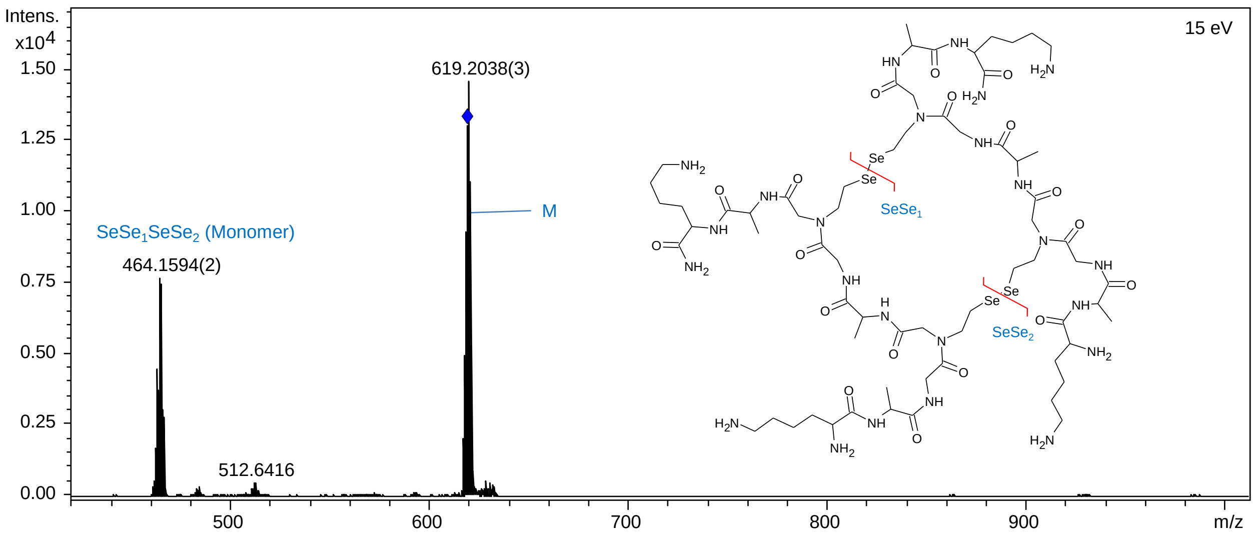


*Figure S96. ESI-qTOF-MS/MS (CE 15 eV) spectrum of the dimer ($C_{66}H_{120}N_{24}O_{18}Se_4$). Precursor ion: m/z $[M+3H]^{3+}$: 619.2038 (calculated for M: 619.2043, z=3+) The notation "SeSe" was used to indicate the cleavage of Se−Se bond.*

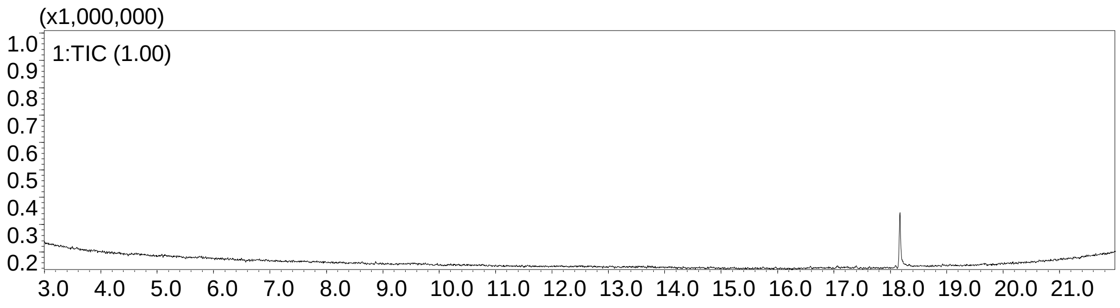


*Figure S97. GC-MS chromatogram acquired after 24 h of exposure of **BBSe$_2$** to visible light*

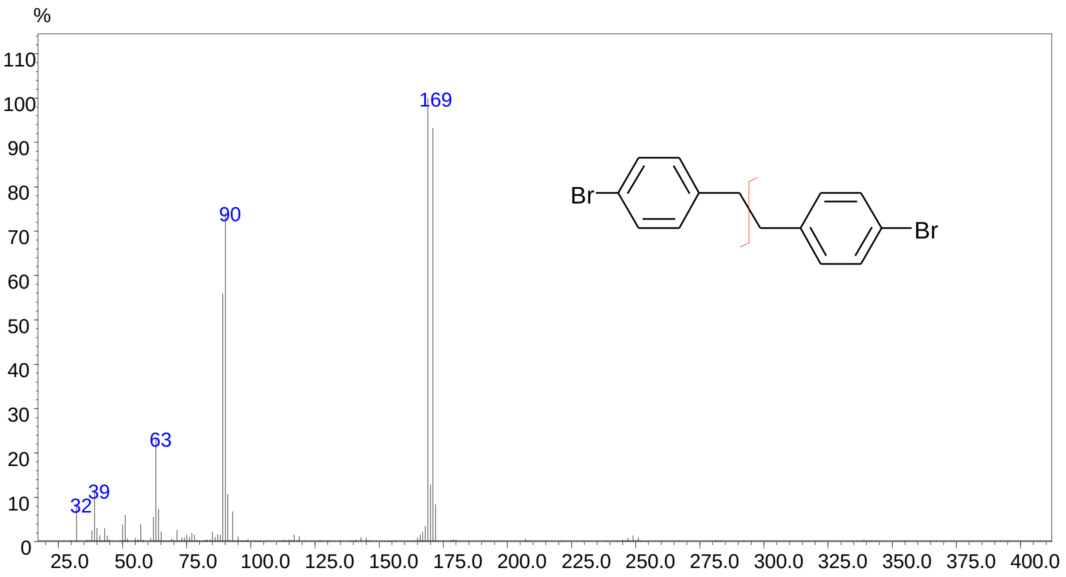


*Figure S98. GC-MS spectrum acquired after 24 h of exposure of **BBSe$_2$** to visible light. (m/z=169 corresponds to the fragment of 1,2-Bis(4-bromophenyl)ethane)*

## 3. Theoretical analyses

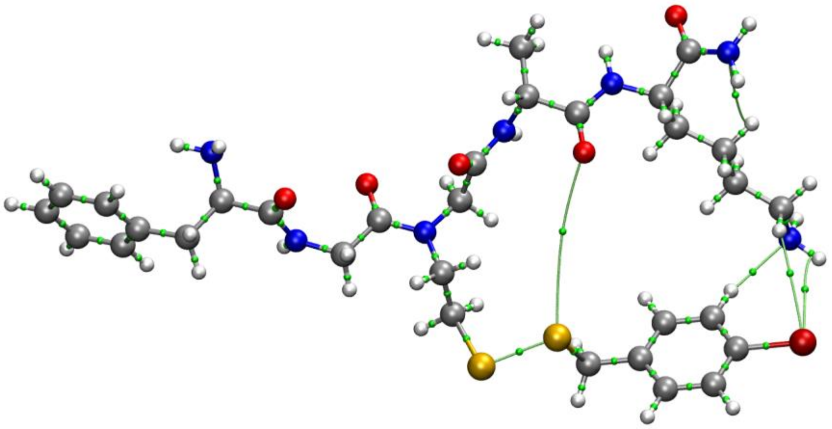

*Figure S99. The linear model of **LM1A***

| System | BCP* | ρ | $\nabla^2\rho$ | H(r) | BDE | compliance constants |
|---|---|---|---|---|---|---|
| **Benzylic C-Se** | BCP11 | 0.1351 | -0.1203 | -0.073 | -41.5989 | 0.547 |
| **Se-Se** | BCP13 | 0.1041 | -0.0332 | -0.045 | -56.5072 | 0.535 |
| **Peptide C-Se** | BCP15 | 0.1429 | -0.1421 | -0.083 | -51.9277 | 0.462 |

*BCP – Bond Critical Point

**BDE – Bond Dissociation Enthalpy

*Table S1. Electronic and thermochemical parameters derived from QTAIM as well as bond dissociation enthalpy (BDE, 298.15K) obtained at the B3LYP-D3BJ/def2-TZVP level of theory (in methanol as a solvent). Electron density (ρ), its Laplacian ($\nabla^2\rho$) and the energy density (H(r)) are given in atomic units, whereas BDE and compliance constants are given in kcal/mol and cm/N, respectively.*

| Products | Bond Critical Point (BCPs) | Interaction type | ρ= e/a0$^{3}$ | $\nabla^2$ρ= e/a0$^{5}$ | Interatomic distance of the non-covalent interaction [Å] | Valence angle [°] |
|---|---|---|---|---|---|---|
| **Product (A)** | BCP20 | N-H…O | 0.0291 | 0.1035 | 1.90 | 152.2 |
| **Product (A)** | BCP145 | N-H…O | 0.0238 | 0.0916 | 2.13 | 110.5 |
| **Product (A)** | BCP64 | C-H…O | 0.0072 | 0.0236 | 2.60 | 165.4 |
| **Product (A)** | BCP75 | C-H…O | 0.0105 | 0.0388 | 2.35 | 170.5 |
| **Product (B)** | BCP24 | N-H…O | 0.0276 | 0.1000 | 1.92 | 152.1 |
| **Product (B)** | BCP112 | N-H…O | 0.0242 | 0.0924 | 2.12 | 110.7 |
| **Product (B)** | BCP38 | C-H…O | 0.0080 | 0.0268 | 2.54 | 166.1 |
| **Product (B)** | BCP58 | C-H…O | 0.0108 | 0.0404 | 2.34 | 172.2 |
| **Product (C)** | BCP44 | N-H…O | 0.0307 | 0.1066 | 1.88 | 152.0 |
| **Product (C)** | BCP103 | N-H…O | 0.0224 | 0.0885 | 2.16 | 109.5 |
| **Product (C)** | BCP72 | C-H…O | 0.0133 | 0.0476 | 2.31 | 148.3 |
| **Product (C)** | BCP76 | C-H…O | 0.0041 | 0.0130 | 2.92 | 155.0 |
| **Product (D)** | BCP67 | N-H…O | 0.0184 | 0.0717 | 2.07 | 154.0 |
| **Product (D)** | BCP127 | N-H…O | 0.0200 | 0.0781 | 2.10 | 131.9 |
| **Product (D)** | BCP64 | O…Se | 0.0093 | 0.0322 | 3.18 | 109.2 |
| **Product (D)** | BCP27 | O…Se | 0.0089 | 0.0292 | 3.24 | 116.3 |
| **Product (D)** | BCP37 | C-H…Se | 0.0070 | 0.0202 | 2.96 | 134.2 |

*Table S2. Geometric parameters obtained at the B3LYP-D3BJ/def2-TZVP level of theory (methanol as a solvent) and electron density (ρ) and its Laplacian ( $\nabla^2$ρ ) in a.u. derived from the QTAIM.*

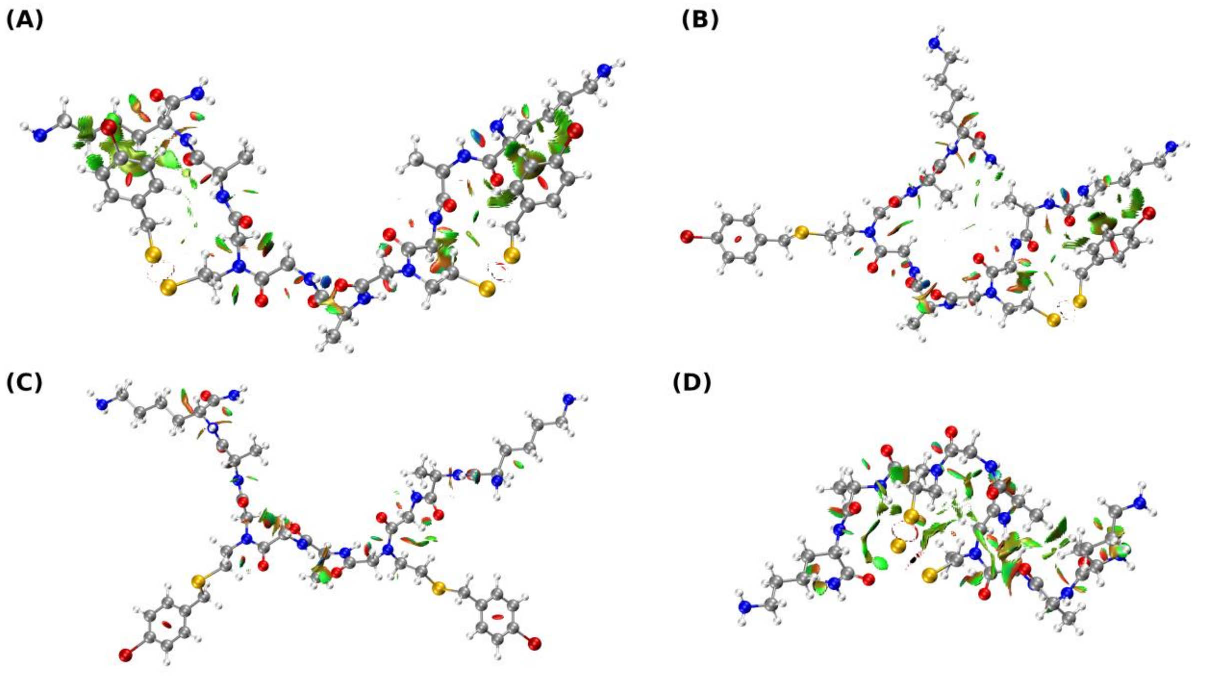


*Figure S100. 3D plots of RDG of the possible products (A, B, C and D) obtained at the B3LYP-D3BJ/def2-TZVP level of theory and with solvent reaction field reproduced by IEF-PCM model and methanol as a solvent. Color coding: gray – carbon, red – oxygen, blue – nitrogen, white – hydrogen, dark yellow - selenium, dark red - bromine.*

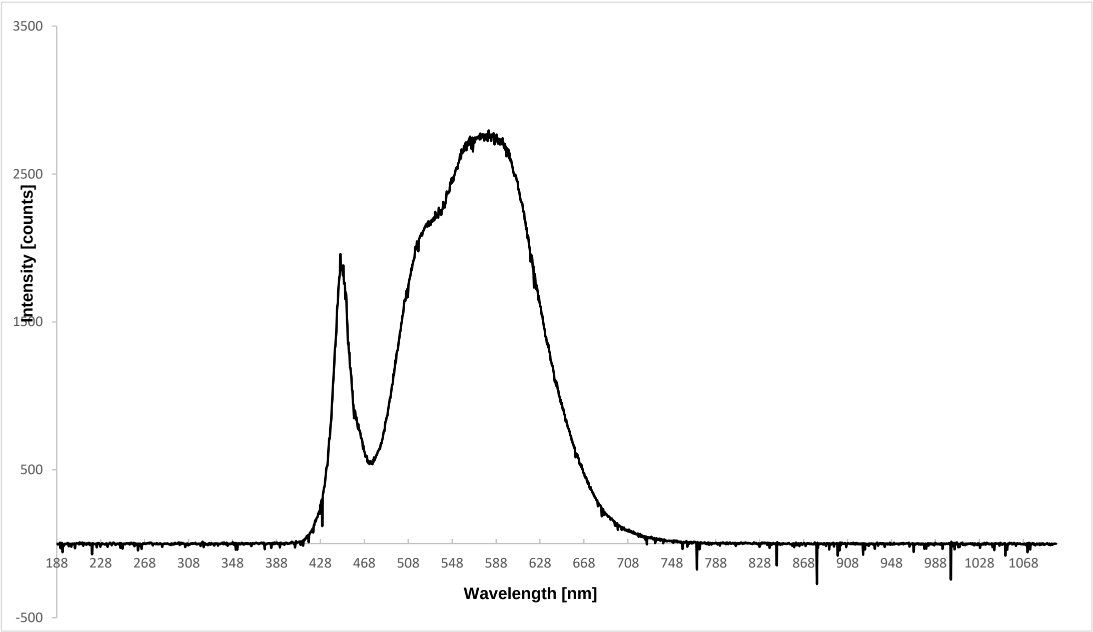


*Figure S101. The measured emission spectrum of the LED lamp.*

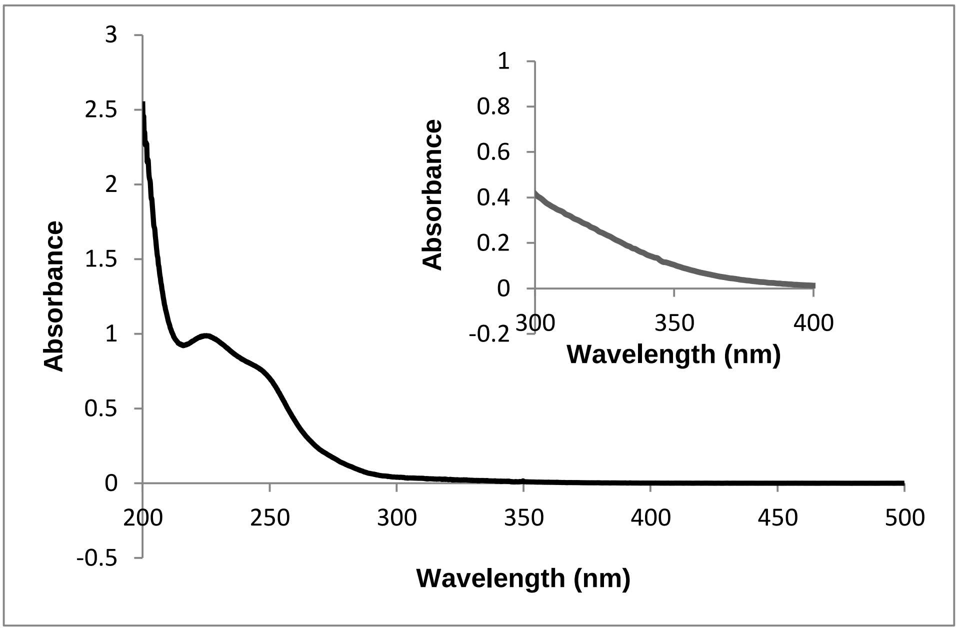


*Figure S102. UV-Vis spectrum of **$BBSe_2$** (the sample was prepared at a concentration of $10^{-4}$ M). The inset shows the absorbance in the range of 300–400 nm (the sample was prepared at a concentration of $10^{-3}$ M).*

**LM1A**

| | | | |
|---|---|---|---|
| C | 10.650621 | -0.514411 | 0.021892 |
| C | 11.621282 | 0.162524 | -0.717171 |
| H | 11.360820 | 0.575056 | -1.684233 |
| C | 12.917048 | 0.307570 | -0.230713 |
| H | 13.656872 | 0.834193 | -0.819988 |
| C | 13.261079 | -0.225025 | 1.005964 |
| H | 14.268777 | -0.115754 | 1.385364 |
| C | 12.301978 | -0.904714 | 1.751783 |
| H | 12.562776 | -1.327235 | 2.713789 |
| C | 11.009670 | -1.046038 | 1.262662 |
| H | 10.268914 | -1.577784 | 1.848319 |
| C | 9.236493 | -0.624838 | -0.474864 |
| C | 8.314634 | 0.456778 | 0.153516 |
| H | 9.204554 | -0.509237 | -1.559878 |
| H | 8.831560 | -1.610267 | -0.233067 |
| C | 6.904533 | 0.245031 | -0.385486 |
| C | 4.740993 | -0.813549 | 0.011244 |
| C | 3.766684 | 0.208483 | 0.610695 |
| H | 4.667130 | -0.779774 | -1.074963 |
| H | 4.481578 | -1.820870 | 0.334405 |
| C | 1.499801 | 0.933047 | 0.942404 |
| C | 1.200480 | 2.042157 | -0.073396 |
| H | 0.564718 | 0.425264 | 1.183994 |
| H | 1.887343 | 1.375803 | 1.857583 |
| C | -0.157195 | 4.079230 | -0.408148 |
| H | 0.055496 | 2.925578 | 1.353972 |
| C | 0.078600 | 5.441129 | 0.232281 |
| H | -0.298915 | 6.243030 | -0.402795 |
| H | -0.397102 | 5.515007 | 1.212506 |
| H | 1.148227 | 5.596017 | 0.366518 |

| | | | |
|---|---|---|---|
| C | -1.618134 | 3.713983 | -0.733515 |
| C | -3.975390 | 4.356581 | -0.599474 |
| H | -2.329278 | 5.481537 | 0.045488 |
| C | -4.678049 | 5.711887 | -0.639003 |
| C | -4.516793 | 3.481786 | 0.548070 |
| H | -4.108260 | 3.829714 | -1.544719 |
| C | -5.918300 | 2.923401 | 0.331206 |
| H | -3.816674 | 2.651876 | 0.658049 |
| H | -4.474929 | 4.062349 | 1.473739 |
| C | -6.334079 | 1.989481 | 1.464006 |
| H | -6.644643 | 3.735597 | 0.245692 |
| H | -5.944913 | 2.376464 | -0.617666 |
| C | -7.691493 | 1.340775 | 1.235959 |
| H | -5.583562 | 1.203275 | 1.586476 |
| H | -6.355209 | 2.548679 | 2.405941 |
| N | 8.704658 | 1.833244 | -0.119832 |
| H | 9.696088 | 1.953109 | 0.056279 |
| H | 8.545301 | 2.029328 | -1.103097 |
| H | 8.313245 | 0.309201 | 1.236049 |
| N | 6.104644 | -0.534868 | 0.371732 |
| O | 6.561591 | 0.702242 | -1.470991 |
| O | 4.177906 | 1.185522 | 1.225004 |
| N | 2.444672 | -0.031850 | 0.432406 |
| C | 1.899720 | -1.126603 | -0.368174 |
| C | 1.484103 | -2.314224 | 0.487879 |
| H | 2.618862 | -1.437451 | -1.120589 |
| H | 1.036864 | -0.739389 | -0.909020 |
| Se | 0.589244 | -3.708561 | -0.591119 |
| H | 2.343472 | -2.814962 | 0.933944 |
| H | 0.801368 | -2.025196 | 1.283186 |
| Se | -1.504938 | -2.716434 | -0.915125 |
| C | -2.500108 | -3.463626 | 0.658218 |
| H | -2.273547 | -4.525746 | 0.667401 |
| H | -2.102350 | -2.989138 | 1.549878 |
| C | -3.956127 | -3.194982 | 0.475033 |
| C | -4.716426 | -3.981920 | -0.394343 |
| H | -4.243438 | -4.791717 | -0.935498 |
| C | -6.071352 | -3.745360 | -0.574544 |
| H | -6.651153 | -4.361903 | -1.246332 |
| C | -6.668832 | -2.707917 | 0.128923 |
| C | -5.946044 | -1.906569 | 0.999050 |
| H | -6.436991 | -1.118119 | 1.555653 |
| C | -4.586520 | -2.153612 | 1.156772 |
| H | -4.012883 | -1.532489 | 1.833595 |
| Br | -8.542826 | -2.382751 | -0.107569 |
| H | 6.404153 | -0.761873 | 1.305570 |
| O | 1.651222 | 2.025172 | -1.209308 |
| N | 0.399805 | 3.015004 | 0.410055 |
| O | -1.879501 | 2.632930 | -1.254245 |
| N | -2.560215 | 4.616598 | -0.418325 |
| O | -4.256423 | 6.656262 | 0.021938 |
| N | -5.779763 | 5.780412 | -1.402109 |
| H | -6.089983 | 5.003833 | -1.959922 |
| H | -6.317413 | 6.632256 | -1.423610 |
| N | -8.033636 | 0.451083 | 2.350885 |
| H | -8.444397 | 2.122433 | 1.063911 |

| | | | |
|---|---|---|---|
| H | -7.655214 | 0.734951 | 0.326840 |
| H | -8.198835 | 0.994049 | 3.191881 |
| H | -8.893266 | -0.048021 | 2.151784 |
| H | 0.370572 | 4.027329 | -1.360433 |

**CM2A**

| | | | |
|---|---|---|---|
| C | -10.598192 | 2.362643 | 2.482262 |
| C | -11.518771 | 1.567339 | 1.816983 |
| H | -12.571806 | 1.806788 | 1.840217 |
| C | -11.063840 | 0.466402 | 1.103413 |
| H | -11.776810 | -0.150522 | 0.571709 |
| C | -9.704698 | 0.158228 | 1.043707 |
| C | -8.805396 | 0.964334 | 1.744740 |
| H | -7.749932 | 0.725332 | 1.728595 |
| C | -9.241370 | 2.069741 | 2.461121 |
| H | -8.536102 | 2.694689 | 2.989829 |
| Br | -11.209281 | 3.902787 | 3.435748 |
| C | -9.223449 | -1.017553 | 0.264146 |
| Se | -8.951084 | -2.537657 | 1.538521 |
| H | -9.943950 | -1.347261 | -0.477647 |
| H | -8.261879 | -0.830826 | -0.206118 |
| Se | -8.188154 | -4.184667 | 0.062045 |
| C | -6.322184 | -3.553788 | -0.201672 |
| C | -5.896319 | -3.763683 | -1.650295 |
| H | -6.325453 | -2.501402 | 0.061312 |
| H | -5.671504 | -4.089961 | 0.482528 |
| N | -4.616588 | -3.118771 | -1.947329 |
| H | -5.802612 | -4.825637 | -1.868282 |
| H | -6.639368 | -3.342241 | -2.325879 |
| C | -4.664374 | -1.760853 | -2.444412 |
| C | -4.780560 | -0.710174 | -1.333237 |
| H | -3.765329 | -1.528611 | -3.012948 |
| H | -5.499495 | -1.673738 | -3.138522 |
| C | -5.582113 | 1.547886 | -0.807370 |
| C | -3.472378 | -3.741830 | -1.560870 |
| C | -2.158042 | -2.990720 | -1.809478 |
| H | -2.066063 | -2.761855 | -2.870876 |
| H | -2.178883 | -2.052261 | -1.257555 |
| N | -1.010503 | -3.751749 | -1.400927 |
| C | -0.449068 | -4.670449 | -2.204240 |
| C | 0.694172 | -5.499810 | -1.599026 |
| C | 1.581694 | -4.110304 | 0.300449 |
| C | 0.138429 | -6.652937 | -0.759634 |
| H | -0.546061 | -7.243696 | -1.368143 |
| H | 0.955443 | -7.295381 | -0.431245 |
| H | -0.395113 | -6.286323 | 0.113727 |
| O | 0.539861 | -4.068524 | 0.957862 |
| O | -0.817726 | -4.883883 | -3.356275 |
| O | -3.482530 | -4.852157 | -1.045048 |
| C | 2.834904 | -3.446818 | 0.862896 |
| C | 5.146747 | -4.140419 | 0.177705 |
| C | 6.111648 | -3.453996 | 1.142092 |
| H | 4.819755 | -5.101055 | 0.571172 |
| H | 5.628496 | -4.350324 | -0.774887 |
| Se | 7.919031 | -4.256686 | 0.971009 |

| | | | |
|---|---|---|---|
| H | 6.217178 | -2.402899 | 0.885585 |
| H | 5.786468 | -3.541113 | 2.175494 |
| Se | 9.173858 | -2.572397 | 1.999083 |
| C | 9.180249 | -1.195713 | 0.544185 |
| H | 9.628303 | -1.656259 | -0.330500 |
| H | 8.142367 | -0.940716 | 0.348792 |
| C | 9.961207 | -0.023037 | 1.030520 |
| C | 11.261197 | 0.208215 | 0.580896 |
| H | 11.709332 | -0.471193 | -0.132592 |
| C | 11.982517 | 1.315304 | 1.009851 |
| H | 12.978872 | 1.498604 | 0.634518 |
| C | 11.399147 | 2.186108 | 1.918586 |
| C | 10.119677 | 1.965083 | 2.409959 |
| H | 9.681081 | 2.647020 | 3.124330 |
| C | 9.406437 | 0.863949 | 1.956977 |
| H | 8.399987 | 0.698265 | 2.319356 |
| N | 3.953801 | -3.322504 | -0.056962 |
| C | 3.942139 | -2.260743 | -0.912627 |
| C | 5.162041 | -2.072967 | -1.824611 |
| C | 5.711924 | 0.233037 | -1.350191 |
| C | 5.673916 | 1.675246 | -1.870496 |
| C | 8.045700 | 2.170153 | -1.549706 |
| C | 9.055627 | 3.076671 | -0.844868 |
| O | 2.978841 | -1.512305 | -0.992641 |
| N | 5.311991 | -0.701357 | -2.233620 |
| O | 6.033977 | -0.048224 | -0.200656 |
| C | 4.334095 | 2.310181 | -1.503182 |
| N | 6.755344 | 2.452247 | -1.316540 |
| O | 8.411920 | 1.250893 | -2.282098 |
| N | 8.403809 | 3.928114 | 0.153661 |
| C | 9.813281 | 3.916146 | -1.878006 |
| C | 11.097695 | 4.527619 | -1.323281 |
| H | 10.058389 | 3.273087 | -2.724664 |
| H | 9.148043 | 4.701584 | -2.251816 |
| C | 11.847189 | 5.368444 | -2.351250 |
| H | 10.880329 | 5.149214 | -0.449160 |
| H | 11.745138 | 3.721540 | -0.965524 |
| C | 13.136740 | 5.959188 | -1.798508 |
| H | 12.081389 | 4.749739 | -3.225103 |
| H | 11.207188 | 6.181447 | -2.707054 |
| N | 13.821870 | 6.770362 | -2.812961 |
| H | 14.667671 | 7.171639 | -2.422584 |
| H | 14.122061 | 6.174842 | -3.578473 |
| H | 12.902674 | 6.609698 | -0.951339 |
| H | 13.762873 | 5.144839 | -1.406953 |
| Br | 12.364034 | 3.735579 | 2.484335 |
| H | -0.720185 | -3.734358 | -0.428243 |
| H | 2.530696 | -2.465871 | 1.222764 |
| H | 3.149150 | -4.036439 | 1.723242 |
| H | 6.080098 | -2.393966 | -1.342288 |
| H | 5.018842 | -2.692546 | -2.710273 |
| H | 4.997505 | -0.423314 | -3.147529 |
| H | 4.208283 | 2.332111 | -0.419813 |
| H | 4.286984 | 3.330158 | -1.884961 |
| H | 3.512198 | 1.740777 | -1.934251 |
| H | 5.781447 | 1.648310 | -2.957516 |

| | | | |
|---|---|---|---|
| H | 6.578156 | 3.212401 | -0.673720 |
| H | 8.931822 | 4.778244 | 0.301168 |
| H | 8.355607 | 3.456720 | 1.048534 |
| H | 9.772524 | 2.388735 | -0.389475 |
| O | -4.274038 | -0.882876 | -0.229902 |
| C | -4.298084 | 2.385046 | -0.741927 |
| H | -4.402364 | 3.200842 | -0.026665 |
| H | -3.471543 | 1.752749 | -0.422826 |
| H | -4.067312 | 2.801590 | -1.723013 |
| H | -5.823689 | 1.184959 | 0.192562 |
| C | -8.372736 | 4.161560 | -0.812330 |
| C | -6.741371 | 2.387674 | -1.346081 |
| O | -7.114478 | 2.290677 | -2.509930 |
| N | -7.264938 | 3.270527 | -0.475906 |
| C | -9.684545 | 3.428428 | -1.113773 |
| C | -10.916390 | 4.238152 | -0.719280 |
| H | -9.722224 | 3.173491 | -2.172109 |
| H | -9.679506 | 2.493328 | -0.553967 |
| C | -12.217674 | 3.537545 | -1.096243 |
| H | -10.895921 | 4.407167 | 0.361810 |
| H | -10.877566 | 5.221532 | -1.192509 |
| C | -13.444317 | 4.218384 | -0.508175 |
| H | -12.309438 | 3.502823 | -2.187633 |
| H | -12.193568 | 2.500485 | -0.753198 |
| C | -7.983940 | 5.149832 | -1.930590 |
| H | -8.520266 | 4.776162 | 0.079013 |
| O | -8.796551 | 5.531297 | -2.761135 |
| N | -6.718661 | 5.608531 | -1.884203 |
| H | -6.049598 | 5.252129 | -1.225070 |
| H | -6.408347 | 6.264220 | -2.581983 |
| N | -14.671505 | 3.499399 | -0.874086 |
| H | -15.478381 | 3.959208 | -0.466305 |
| H | -14.803445 | 3.540983 | -1.879879 |
| H | -13.367488 | 4.215969 | 0.582566 |
| H | -13.455814 | 5.272383 | -0.820890 |
| H | -6.965527 | 3.236443 | 0.484514 |
| N | -5.440312 | 0.403790 | -1.684026 |
| H | -5.873274 | 0.493061 | -2.592808 |
| N | 1.705606 | -4.711856 | -0.889397 |
| H | 1.211254 | -5.916141 | -2.460201 |
| H | 2.602734 | -4.627147 | -1.339729 |

**CM2B**

| | | | |
|---|---|---|---|
| Se | -9.961745 | -0.418851 | 0.874768 |
| C | -8.266548 | -0.391957 | -0.135517 |
| C | -7.092187 | -0.289755 | 0.827023 |
| H | -8.202886 | -1.311059 | -0.714165 |
| H | -8.296155 | 0.459989 | -0.813122 |
| N | -5.818638 | -0.248633 | 0.111668 |
| H | -7.162629 | 0.611123 | 1.435220 |
| H | -7.075261 | -1.148567 | 1.493995 |
| C | -5.281452 | 1.045178 | -0.260545 |
| C | -4.193480 | 1.531076 | 0.704121 |
| H | -4.915992 | 1.039590 | -1.286527 |
| H | -6.092958 | 1.772008 | -0.229376 |

| | | | |
|---|---|---|---|
| C | -2.132583 | 2.858547 | 0.871922 |
| C | -5.158213 | -1.411047 | -0.122959 |
| C | -3.780142 | -1.278713 | -0.777537 |
| H | -3.884580 | -0.826104 | -1.762837 |
| H | -3.153737 | -0.619606 | -0.175142 |
| N | -3.119043 | -2.542356 | -0.935895 |
| C | -3.026610 | -3.167574 | -2.121865 |
| C | -2.271051 | -4.506211 | -2.117153 |
| C | -0.759740 | -4.378937 | -0.110596 |
| C | -3.167518 | -5.643577 | -1.623728 |
| H | -4.067726 | -5.681133 | -2.237266 |
| H | -2.642229 | -6.593833 | -1.719683 |
| H | -3.454471 | -5.503375 | -0.584925 |
| O | -1.649330 | -4.334568 | 0.740067 |
| O | -3.511844 | -2.736847 | -3.164722 |
| O | -5.619926 | -2.504737 | 0.178545 |
| C | 0.700718 | -4.388380 | 0.331380 |
| C | 2.705436 | -4.829780 | -1.122168 |
| C | 3.919925 | -4.778924 | -0.198742 |
| H | 2.286117 | -5.833449 | -1.161168 |
| H | 2.979681 | -4.574574 | -2.143496 |
| Se | 5.495417 | -5.559977 | -1.119469 |
| H | 4.176795 | -3.747615 | 0.029865 |
| H | 3.749739 | -5.322141 | 0.727241 |
| Se | 7.193640 | -4.685993 | 0.229670 |
| C | 7.219921 | -2.790323 | -0.416495 |
| H | 7.413456 | -2.818887 | -1.484003 |
| H | 6.233709 | -2.379321 | -0.220113 |
| C | 8.285379 | -2.066776 | 0.333560 |
| C | 9.502789 | -1.755719 | -0.271947 |
| H | 9.677268 | -2.042059 | -1.301076 |
| C | 10.486521 | -1.054093 | 0.413692 |
| H | 11.414713 | -0.794384 | -0.074337 |
| C | 10.253250 | -0.683225 | 1.729869 |
| C | 9.065869 | -1.009096 | 2.371393 |
| H | 8.902418 | -0.723487 | 3.400780 |
| C | 8.085979 | -1.692913 | 1.665305 |
| H | 7.148333 | -1.928887 | 2.151986 |
| N | 1.649188 | -3.921130 | -0.668694 |
| C | 1.679309 | -2.577211 | -0.893768 |
| C | 2.733558 | -2.037026 | -1.868498 |
| C | 3.795103 | -0.359169 | -0.487641 |
| C | 3.935171 | 1.140854 | -0.201440 |
| C | 6.372877 | 1.229220 | -0.383661 |
| C | 7.671016 | 1.606673 | 0.331959 |
| O | 0.863784 | -1.826856 | -0.376005 |
| N | 3.067588 | -0.666902 | -1.577831 |
| O | 4.262921 | -1.217521 | 0.252855 |
| C | 2.873052 | 1.553724 | 0.814827 |
| N | 5.247196 | 1.450249 | 0.311280 |
| O | 6.380374 | 0.757160 | -1.520349 |
| N | 7.428262 | 1.947070 | 1.736414 |
| C | 8.358592 | 2.757855 | -0.408101 |
| C | 9.824716 | 2.926366 | -0.016541 |
| H | 8.292804 | 2.559177 | -1.478894 |
| H | 7.804568 | 3.683089 | -0.217433 |

| | | | |
|---|---|---|---|
| C | 10.511610 | 4.066082 | -0.761377 |
| H | 9.916835 | 3.101972 | 1.059805 |
| H | 10.351636 | 1.988229 | -0.214816 |
| C | 11.978080 | 4.215218 | -0.381176 |
| H | 10.436607 | 3.891506 | -1.840781 |
| H | 9.994505 | 5.009296 | -0.561097 |
| N | 12.600999 | 5.326662 | -1.111133 |
| H | 13.571307 | 5.424004 | -0.832146 |
| H | 12.615125 | 5.115705 | -2.104145 |
| H | 12.054485 | 4.431683 | 0.687920 |
| H | 12.488375 | 3.255467 | -0.545673 |
| Br | 11.579168 | 0.322006 | 2.669650 |
| H | -2.772917 | -3.027815 | -0.114855 |
| H | 0.754168 | -3.777694 | 1.230725 |
| H | 0.956190 | -5.412761 | 0.600602 |
| H | 3.643777 | -2.628142 | -1.849746 |
| H | 2.320745 | -2.094271 | -2.875982 |
| H | 2.651718 | 0.067743 | -2.124452 |
| H | 2.990284 | 0.988163 | 1.739708 |
| H | 2.967702 | 2.615729 | 1.044365 |
| H | 1.876674 | 1.363861 | 0.420988 |
| H | 3.781638 | 1.684834 | -1.137024 |
| H | 5.370229 | 1.829855 | 1.240508 |
| H | 8.126893 | 2.594954 | 2.075799 |
| H | 7.488300 | 1.122116 | 2.320219 |
| H | 8.308045 | 0.724324 | 0.231628 |
| O | -4.217394 | 1.245167 | 1.895707 |
| C | -1.058936 | 1.806584 | 1.180626 |
| H | -0.251537 | 2.236301 | 1.772885 |
| H | -1.503896 | 0.990588 | 1.746834 |
| H | -0.645231 | 1.407700 | 0.253872 |
| H | -2.510227 | 3.273394 | 1.807892 |
| C | -0.160598 | 6.015007 | -0.012876 |
| C | -1.553932 | 3.980165 | 0.006786 |
| O | -1.770523 | 4.032400 | -1.198905 |
| N | -0.751437 | 4.851831 | 0.646817 |
| C | -1.191872 | 7.087347 | -0.375880 |
| C | -0.595934 | 8.489158 | -0.476597 |
| H | -1.679310 | 6.816329 | -1.312360 |
| H | -1.957904 | 7.079492 | 0.403030 |
| C | -1.639624 | 9.538916 | -0.845417 |
| H | -0.139106 | 8.753991 | 0.483578 |
| H | 0.204400 | 8.490878 | -1.218621 |
| C | -1.061886 | 10.944753 | -0.927340 |
| H | -2.086962 | 9.279380 | -1.811707 |
| H | -2.453218 | 9.531492 | -0.113414 |
| C | 0.711323 | 5.592322 | -1.212518 |
| H | 0.534173 | 6.436265 | 0.717899 |
| O | 0.730353 | 6.227278 | -2.256519 |
| N | 1.502564 | 4.523407 | -0.993352 |
| H | 1.421899 | 3.972609 | -0.157389 |
| H | 2.092258 | 4.188271 | -1.737032 |
| N | -2.101662 | 11.921547 | -1.276622 |
| H | -1.703530 | 12.853798 | -1.311835 |
| H | -2.447445 | 11.729556 | -2.211741 |
| H | -0.652550 | 11.225766 | 0.046970 |

| | | | |
|---|---|---|---|
| H | -0.220849 | 10.944092 | -1.635261 |
| H | -0.699960 | 4.810618 | 1.651950 |
| N | -3.243320 | 2.289385 | 0.136429 |
| H | -3.251419 | 2.482581 | -0.855502 |
| N | -0.977413 | -4.447286 | -1.431786 |
| H | -2.031867 | -4.685020 | -3.162734 |
| H | -0.162013 | -4.396690 | -2.021320 |
| C | -11.159461 | -0.581033 | -0.704698 |
| H | -10.964690 | 0.278760 | -1.340992 |
| H | -10.877078 | -1.492906 | -1.225026 |
| C | -12.579440 | -0.621101 | -0.245362 |
| C | -13.311177 | 0.556628 | -0.088833 |
| H | -12.850353 | 1.508273 | -0.322131 |
| C | -14.625377 | 0.531098 | 0.359892 |
| H | -15.183825 | 1.449224 | 0.472624 |
| C | -15.208330 | -0.692060 | 0.658263 |
| C | -14.506959 | -1.880505 | 0.515021 |
| H | -14.973861 | -2.826754 | 0.747702 |
| C | -13.193743 | -1.834899 | 0.064929 |
| H | -12.640811 | -2.758976 | -0.047734 |
| Br | -17.019589 | -0.741310 | 1.276337 |

**CM2C**

| | | | |
|---|---|---|---|
| Se | 7.988160 | 2.994925 | 1.029242 |
| C | 6.318780 | 1.993207 | 0.707860 |
| C | 6.201387 | 0.843376 | 1.697958 |
| H | 5.482734 | 2.679007 | 0.826535 |
| H | 6.343311 | 1.628095 | -0.317780 |
| N | 4.978581 | 0.075293 | 1.470197 |
| H | 7.048352 | 0.163376 | 1.605089 |
| H | 6.178092 | 1.222871 | 2.716562 |
| C | 5.057203 | -1.040684 | 0.553889 |
| C | 5.290639 | -2.376718 | 1.265776 |
| H | 4.147141 | -1.103875 | -0.043034 |
| H | 5.870493 | -0.854004 | -0.146757 |
| C | 5.827319 | -4.744631 | 0.902555 |
| C | 3.865721 | 0.414554 | 2.160588 |
| C | 2.605976 | -0.442580 | 2.015603 |
| H | 2.757767 | -1.356582 | 1.453901 |
| H | 2.304305 | -0.712770 | 3.025646 |
| N | 1.533919 | 0.318735 | 1.406308 |
| C | 1.420150 | 0.434247 | 0.074852 |
| C | 0.306274 | 1.357941 | -0.440781 |
| C | -1.404053 | 1.412091 | 1.402361 |
| C | 0.740029 | 2.823562 | -0.353284 |
| H | 1.686688 | 2.951564 | -0.878313 |
| H | -0.009137 | 3.457072 | -0.827880 |
| H | 0.865453 | 3.139286 | 0.679654 |
| O | -0.664365 | 1.852804 | 2.284699 |
| O | 2.175350 | -0.120126 | -0.722871 |
| O | 3.827804 | 1.396473 | 2.899824 |
| C | -2.870244 | 1.155351 | 1.733732 |
| C | -4.457987 | 1.737908 | -0.095483 |
| C | -5.788279 | 2.042231 | 0.580708 |
| H | -3.835655 | 2.631478 | -0.136206 |

| | | | |
|---|---|---|---|
| H | -4.606580 | 1.421947 | -1.125434 |
| Se | -6.799231 | 3.360739 | -0.482289 |
| H | -6.395116 | 1.141917 | 0.660193 |
| H | -5.634473 | 2.451861 | 1.577325 |
| N | -3.704983 | 0.711484 | 0.631815 |
| C | -3.906441 | -0.627792 | 0.473256 |
| C | -4.843296 | -1.066554 | -0.660015 |
| C | -6.309466 | -2.615850 | 0.479567 |
| C | -6.628633 | -4.092394 | 0.747277 |
| C | -8.977550 | -4.075573 | 0.071999 |
| C | -10.418497 | -4.292083 | 0.537307 |
| O | -3.333755 | -1.455693 | 1.167010 |
| N | -5.352131 | -2.394522 | -0.443789 |
| O | -6.856443 | -1.705619 | 1.090622 |
| C | -5.809271 | -4.578494 | 1.940942 |
| N | -8.036407 | -4.274053 | 1.008190 |
| O | -8.718043 | -3.779791 | -1.093775 |
| N | -10.520005 | -4.197851 | 1.994793 |
| C | -10.887564 | -5.666058 | 0.042535 |
| C | -12.389005 | -5.901811 | 0.189087 |
| H | -10.614729 | -5.752794 | -1.011047 |
| H | -10.332570 | -6.439008 | 0.583578 |
| C | -12.821295 | -7.254183 | -0.371014 |
| H | -12.686176 | -5.848842 | 1.241270 |
| H | -12.929075 | -5.100452 | -0.325357 |
| C | -14.315762 | -7.505767 | -0.228258 |
| H | -12.547732 | -7.310488 | -1.430682 |
| H | -12.276288 | -8.056715 | 0.134892 |
| N | -14.680902 | -8.819529 | -0.773458 |
| H | -15.675333 | -8.979903 | -0.654535 |
| H | -14.513455 | -8.830493 | -1.774782 |
| H | -14.585256 | -7.496284 | 0.831430 |
| H | -14.864192 | -6.678805 | -0.701440 |
| H | 0.924271 | 0.886986 | 1.988028 |
| H | -2.887320 | 0.409103 | 2.525484 |
| H | -3.268476 | 2.083323 | 2.142235 |
| H | -5.684382 | -0.387783 | -0.764108 |
| H | -4.282029 | -1.048070 | -1.594748 |
| H | -4.876128 | -3.175251 | -0.862990 |
| H | -6.064504 | -4.007458 | 2.834836 |
| H | -6.009576 | -5.633663 | 2.126842 |
| H | -4.744876 | -4.455509 | 1.747291 |
| H | -6.364513 | -4.669772 | -0.141120 |
| H | -8.372533 | -4.438447 | 1.947357 |
| H | -11.374683 | -4.627757 | 2.323826 |
| H | -10.544675 | -3.229627 | 2.293250 |
| H | -11.007487 | -3.524790 | 0.021120 |
| O | 5.143596 | -2.505139 | 2.476041 |
| C | 4.497053 | -5.479390 | 1.114138 |
| H | 4.662552 | -6.477943 | 1.518262 |
| H | 3.885255 | -4.919510 | 1.819484 |
| H | 3.958206 | -5.565510 | 0.169837 |
| H | 6.374090 | -4.713823 | 1.846480 |
| C | 8.110455 | -7.408123 | -0.618924 |
| C | 6.674742 | -5.456389 | -0.153284 |
| O | 6.775147 | -5.020949 | -1.295051 |

| | | | |
|---|---|---|---|
| N | 7.239553 | -6.612780 | 0.243977 |
| C | 9.463530 | -6.741752 | -0.883928 |
| C | 10.570438 | -7.735276 | -1.227392 |
| H | 9.354038 | -6.008014 | -1.682475 |
| H | 9.740335 | -6.196820 | 0.021650 |
| C | 11.906856 | -7.047648 | -1.489256 |
| H | 10.684335 | -8.445019 | -0.400254 |
| H | 10.277710 | -8.315834 | -2.104004 |
| C | 13.025212 | -8.028804 | -1.810864 |
| H | 11.794999 | -6.346907 | -2.324545 |
| H | 12.197378 | -6.451044 | -0.618941 |
| C | 7.381299 | -7.822266 | -1.913292 |
| H | 8.284336 | -8.337901 | -0.071511 |
| O | 7.943934 | -7.836553 | -2.998518 |
| N | 6.110582 | -8.237928 | -1.743716 |
| H | 5.640734 | -8.150214 | -0.859600 |
| H | 5.571860 | -8.506947 | -2.550310 |
| N | 14.292625 | -7.323947 | -2.043593 |
| H | 15.029340 | -7.992054 | -2.242868 |
| H | 14.210565 | -6.742606 | -2.871945 |
| H | 13.169109 | -8.704995 | -0.963584 |
| H | 12.719395 | -8.651367 | -2.663734 |
| H | 7.196075 | -6.860852 | 1.219239 |
| N | 5.627258 | -3.385335 | 0.445756 |
| H | 5.775097 | -3.229251 | -0.541838 |
| N | -1.009726 | 1.116385 | 0.157129 |
| H | 0.197097 | 1.090456 | -1.488820 |
| H | -1.711917 | 0.728185 | -0.451979 |
| C | 7.746951 | 4.303737 | -0.448852 |
| H | 7.676388 | 3.732001 | -1.370996 |
| H | 6.807835 | 4.819955 | -0.265555 |
| C | 8.903654 | 5.247486 | -0.468126 |
| C | 10.051110 | 4.953686 | -1.205730 |
| H | 10.095881 | 4.040204 | -1.785269 |
| C | 11.140286 | 5.815514 | -1.212800 |
| H | 12.021586 | 5.580020 | -1.791882 |
| C | 11.076045 | 6.983360 | -0.466531 |
| C | 9.950667 | 7.302912 | 0.279283 |
| H | 9.912326 | 8.217272 | 0.853719 |
| C | 8.871404 | 6.428631 | 0.273955 |
| H | 7.990687 | 6.671928 | 0.854829 |
| Br | 12.569676 | 8.181013 | -0.469695 |
| C | -8.453790 | 3.304034 | 0.620539 |
| H | -8.786731 | 2.269180 | 0.634723 |
| H | -8.179011 | 3.614978 | 1.625310 |
| C | -9.477261 | 4.209197 | 0.018863 |
| C | -9.544924 | 5.553059 | 0.387869 |
| H | -8.860296 | 5.939047 | 1.132618 |
| C | -10.479659 | 6.407641 | -0.182391 |
| H | -10.526223 | 7.445459 | 0.114658 |
| C | -11.352721 | 5.905611 | -1.136616 |
| C | -11.309264 | 4.574737 | -1.526555 |
| H | -11.997354 | 4.195724 | -2.268435 |
| C | -10.367513 | 3.735475 | -0.945522 |
| H | -10.327737 | 2.696173 | -1.246509 |
| Br | -12.647441 | 7.072342 | -1.928390 |

**C2T**

| | | | |
|---|---|---|---|
| C | -6.354981 | -0.892109 | -0.212912 |
| C | -5.767077 | -2.001340 | -1.100216 |
| N | -6.201255 | 0.365634 | -0.933585 |
| C | -5.618860 | 1.458030 | -0.401112 |
| O | -5.177085 | 1.501479 | 0.741388 |
| C | -5.466939 | 2.675494 | -1.330779 |
| C | -6.354946 | 2.710645 | -2.567933 |
| H | -6.216199 | 3.660920 | -3.081971 |
| H | -6.102665 | 1.916732 | -3.274365 |
| H | -7.406939 | 2.621362 | -2.295803 |
| N | -4.059452 | 2.764876 | -1.688820 |
| H | -5.670015 | 3.542707 | -0.705445 |
| C | -3.233979 | 3.735330 | -1.243865 |
| O | -3.590603 | 4.664270 | -0.531481 |
| C | -1.785396 | 3.595534 | -1.706928 |
| N | -0.846519 | 3.600691 | -0.600417 |
| H | -1.642716 | 2.681800 | -2.285548 |
| H | -1.558461 | 4.449750 | -2.340665 |
| C | -0.042953 | 4.680626 | -0.424965 |
| N | 1.057898 | -0.867189 | -2.556920 |
| C | -0.113927 | -1.148698 | -1.718314 |
| C | 0.289758 | -1.295034 | -0.256646 |
| H | -0.829987 | -0.340063 | -1.845925 |
| H | -0.575595 | -2.068982 | -2.068917 |
| Se | -1.061641 | -2.173262 | 0.870444 |
| H | 1.148744 | -1.959337 | -0.169823 |
| H | 0.543549 | -0.342521 | 0.202636 |
| Se | -2.772117 | -0.566642 | 1.011479 |
| Se | -1.896072 | 0.972586 | 2.590344 |
| C | -1.807827 | 2.574308 | 1.442835 |
| C | -0.845098 | 2.425991 | 0.273754 |
| H | -2.821082 | 2.784981 | 1.118948 |
| H | -1.487595 | 3.365518 | 2.121372 |
| H | 0.156654 | 2.224248 | 0.638378 |
| H | -1.141916 | 1.572751 | -0.334654 |
| C | 1.866099 | -1.920980 | -2.847567 |
| C | 3.187635 | -1.633367 | -3.572287 |
| N | 4.222529 | -2.500521 | -3.060457 |
| H | 3.058786 | -1.835371 | -4.635026 |
| H | 3.494113 | -0.595927 | -3.463737 |
| C | 4.497379 | -2.523348 | -1.745769 |
| C | 5.449103 | -3.621791 | -1.252124 |
| C | 6.216382 | -4.381239 | -2.334839 |
| H | 6.847828 | -5.131426 | -1.860825 |
| H | 5.538637 | -4.908159 | -3.008018 |
| H | 6.852721 | -3.710331 | -2.910441 |
| N | 6.340834 | -3.097746 | -0.230951 |
| C | 7.139604 | -2.028875 | -0.423614 |
| O | 7.130206 | -1.381403 | -1.467160 |
| C | 8.043572 | -1.655664 | 0.761964 |
| C | 7.253081 | -0.768868 | 1.739282 |
| C | 7.752972 | -0.788982 | 3.187656 |
| H | 7.245198 | 0.248319 | 1.343668 |

| | | | |
|---|---|---|---|
| H | 6.212531 | -1.098934 | 1.744153 |
| C | 9.158130 | -0.242052 | 3.445327 |
| H | 7.038076 | -0.222644 | 3.793813 |
| H | 7.705520 | -1.820337 | 3.550533 |
| C | 9.311665 | 1.237863 | 3.125992 |
| H | 9.407273 | -0.394684 | 4.499618 |
| H | 9.885008 | -0.805303 | 2.856411 |
| N | 10.674264 | 1.694760 | 3.439473 |
| N | 9.273498 | -1.002508 | 0.328003 |
| H | -5.700164 | -0.829127 | 0.652375 |
| H | -6.576843 | 0.423628 | -1.866039 |
| H | 4.648495 | -3.166594 | -3.678653 |
| H | 8.319920 | -2.579385 | 1.278627 |
| H | 9.817315 | -1.640381 | -0.244015 |
| H | 9.029121 | -0.226284 | -0.279668 |
| H | 9.042840 | 1.416365 | 2.078370 |
| H | 8.613599 | 1.817188 | 3.737674 |
| H | 11.335008 | 1.207584 | 2.841568 |
| H | 10.768627 | 2.679085 | 3.212512 |
| O | -4.594616 | -2.338213 | -0.992834 |
| N | -6.592509 | -2.524395 | -2.026347 |
| H | -7.571019 | -2.298129 | -2.065767 |
| H | -6.247201 | -3.242270 | -2.643021 |
| H | -3.669866 | 2.022457 | -2.248478 |
| O | -0.132234 | 5.695985 | -1.108008 |
| O | 1.581369 | -3.064329 | -2.519660 |
| O | 3.949325 | -1.756640 | -0.956091 |
| H | 4.787601 | -4.324934 | -0.740992 |
| H | 6.406379 | -3.602473 | 0.637024 |
| C | 1.092837 | 4.561469 | 0.592446 |
| N | 2.165183 | 3.789566 | -0.010179 |
| H | 1.422950 | 5.575076 | 0.813290 |
| H | 0.803289 | 4.080389 | 1.522683 |
| C | 2.690445 | 2.670431 | 0.529140 |
| O | 2.438247 | 2.267532 | 1.658348 |
| C | -7.791097 | -1.111159 | 0.262703 |
| C | -7.952061 | -2.366899 | 1.115172 |
| H | -8.072729 | -0.231441 | 0.846143 |
| H | -8.479347 | -1.137390 | -0.588447 |
| C | -9.383857 | -2.564315 | 1.603327 |
| H | -7.642852 | -3.247201 | 0.543607 |
| H | -7.275816 | -2.303026 | 1.973290 |
| C | -9.549677 | -3.814795 | 2.455286 |
| H | -9.692828 | -1.689056 | 2.185701 |
| H | -10.062553 | -2.625644 | 0.747378 |
| N | -10.943847 | -3.973328 | 2.887785 |
| H | -11.042282 | -4.821828 | 3.434682 |
| H | -11.196420 | -3.210103 | 3.507847 |
| H | -9.280472 | -4.694058 | 1.863690 |
| H | -8.842865 | -3.770750 | 3.295990 |
| H | 2.330562 | 3.920390 | -1.000664 |
| C | 3.613651 | 1.906670 | -0.441483 |
| C | 4.685825 | 1.131179 | 0.294076 |
| H | 5.315090 | 0.591675 | -0.410696 |
| H | 5.305730 | 1.819351 | 0.867396 |
| H | 4.243526 | 0.414436 | 0.983060 |

| | | | |
|---|---|---|---|
| N | 2.781144 | 1.012439 | -1.249719 |
| H | 4.072771 | 2.628061 | -1.119009 |
| C | 2.074875 | 1.466272 | -2.294831 |
| O | 2.058664 | 2.646427 | -2.648545 |
| C | 1.244584 | 0.466246 | -3.097055 |
| H | 0.266045 | 0.926587 | -3.222417 |
| H | 1.677327 | 0.421744 | -4.095576 |
| H | 2.877703 | 0.013585 | -1.091933 |
